# Unraveling the coastal marine plastisphere archaeome

**DOI:** 10.1038/s41467-026-76782-z

**Published:** 2026-08-14

**Authors:** Changchao Li, Yijing Wang, Albert Shing King Zhou, Xi Pan, Yanjie Zhu, Xiaohua Zhang, Tian Chen, Anqi Xiong, Yuen-Wa Ho, Jian Liu, Zhichao Zhou, Jie Wang, Tanveer M. Adyel, James Kar-Hei Fang, Michael S. Bank, Matthias C. Rillig, Ling N. Jin

**Affiliations:** 1 Department of Civil and Environmental Engineering, The Hong Kong Polytechnic University, Hong Kong, China; 2 PolyU-BGI Joint Research Centre for Genomics and Synthetic Biology in Global Ocean Resources, The Hong Kong Polytechnic University, Hong Kong, China; 3 Environment Research Institute, Shandong University, Qingdao, China; 4 Institute of Biology, Freie Universität Berlin, Berlin, Germany; 5 Concord Academy, Concord, Massachusetts, USA; 6 Department of Food Science and Nutrition, The Hong Kong Polytechnic University, Hong Kong, China; 7 Institute for Advanced Study, Shenzhen University, Shenzhen, China; 8 College of Resources and Environmental Sciences, China Agricultural University, Beijing, China; 9 Centre for Nature Positive Solutions, Department of Biology, School of Science, RMIT University, Melbourne, Australia; 10 Research Institute for Future Food and Research Centre for Nature-based Urban Infrastructure Solutions, The Hong Kong Polytechnic University, Hong Kong, China; 11 State Key Laboratory of Marine Environmental Health, City University of Hong Kong, Hong Kong, China; 12 Institute of Marine Research, Bergen, Norway; 13 University of Massachusetts Amherst, Amherst, Massachusetts, USA; 14 Department of Health Technology and Informatics and Mental Health Research Centre, The Hong Kong Polytechnic University, Hong Kong, China

**Keywords:** Environmental impact, Microbial ecology, Community ecology, Water microbiology, Metagenomics

## Abstract

Plastic pollution has created an expanding anthropogenic microbial niche, the plastisphere, raising questions about microbial ecology and associated impacts. Archaea, the third domain of life with fundamental ecological and evolutionary significance, remain poorly understood in this habitat. Here, using paired plastic debris and bulk-water samples from coastal marine ecosystems, key archaeal habitats increasingly threatened by plastic pollution, we characterize the plastisphere archaeome through archaeal amplicon sequencing and metagenomics. We show that the archaeome is significantly reshaped in the plastisphere, exhibiting higher taxonomic diversity, greater community heterogeneity, and selective enrichment of Euryarchaeota and Crenarchaeota. Archaeal genes involved in methane, nitrogen, and sulfur cycling are enriched in the plastisphere. Taxonomic and functional divergence between the plastisphere and bulk water increases with anthropogenic chemical stress. These findings suggest that plastic pollution could alter marine archaeal diversity, biogeography, and biogeochemical potential, extending understanding of plastisphere impacts to the archaeal domain.

## Abstract (web summary)

Plastic pollution creates new habitats for microbes in the ocean, but little is known about how it affects archaea. Here, the authors characterized the plastisphere archaeome and the associated potential ecological impacts from bulk water samples paired with microplastics.

## Introduction

Growing plastic pollution exacerbates the impacts of all planetary boundaries and challenges efforts to achieve a sustainable future^1–6^. More than 400 million tons of plastic waste are produced each year, and this number continues to grow^7^. By 2050, the amount of plastic waste accumulated in the environment is projected to double compared to current levels^6^.

As one of the most representative anthropogenic novel entities, plastics are characterized by properties such as persistence, organic composition, hydrophobicity, buoyancy, and a wide range of polymer types, shapes and sizes^8–11^. These distinctive properties create a unique niche for microorganisms in the environment, giving rise to a novel microbial ecosystem with plastic as the central substrate, termed the “plastisphere”^12–15^. This new microbial habitat has several notable characteristics: (i) A vast and expanding surface area due to the huge stock of plastic waste in the environment, increasing inputs, and its poor degradability^15^; (ii) Massive microbial colonization, because most microorganisms prefer living attached to surfaces rather than pelagic^15^. The hydrophobic organic surface of plastics, along with organic matter adsorbed from the surrounding environment and leached from the plastic itself, provides abundant carbon and nutrients for microbes^13,15–17^; (iii) Access to larger life forms, as plastic debris has been found to infiltrate virtually all kinds of larger organisms, including humans and even plants^1,18–22^; (iv) Long-distance dispersal of microorganisms, as plastic debris is highly mobile and can carry attached microbes over long distances and across ecosystems^15,23–25^.

Understanding the microbiology of this novel habitat is crucial to further understand how human activities are altering the natural microbial world, and what these changes mean for ecosystems and human well-being^15,26–28^. Research in this emerging field has intensified in recent years, yielding foundational knowledge. For example, our previous studies revealed that the plastisphere selectively assembles distinct bacterial, fungal, and algal communities, altering their diversity and functional profiles, and enriching pathogens, harmful genes, and microbes involved in organic compound degradation^29–32^. Other studies further suggest that the plastisphere can support viral persistence^33^, facilitate the dissemination of antimicrobial resistance^28,34–36^, and, through bacteria–virus interactions, affect ecosystem carbon sequestration^37^. Together, these findings highlight the potentially severe impacts of the plastisphere microbiome on ecosystem and human health^15,28,38^. We therefore emphasize the importance of recognizing indirect and hidden microbiological impacts of plastic pollution in order to develop effective, adaptive management strategies^15,23,26^.

Although archaea were formally recognized only a few decades ago, this third domain of life is recognized to be of important ecological and evolutionary significance^39–43^. It constitutes a significant fraction of global microbial biomass, plays a key role in mediating global biogeochemical cycles and climate change^39–42^, and has been implicated in the origin of eukaryotic life^44,45^. Furthermore, archaea are also important components of host-associated microbiomes (including those of humans), where they interact with other microorganisms and may influence host health^43,46^. Although some previous plastisphere studies have detected archaea^47,48^, a systematic understanding is still lacking regarding which archaeal taxa colonize the plastisphere, how archaeal communities respond to the plastisphere’s unique conditions, and what ecological impacts may be associated with these changes.

Coastal ecosystems, as critical interfaces between the land and open ocean, harbor high degrees of biodiversity, support billions of people through food provision, ecosystem services and coastal protection, and regulate global elemental cycles and climate^41,49,50^. Furthermore, coastal ecosystems represent important habitats for archaea, where they contribute substantially to key ecological functions such as nutrient cycling, organic matter transformation, and greenhouse gas regulation^41,51^. However, these ecosystems are now facing critical and growing threats from plastic pollution^20,22,38,49,52–55^.

Therefore, to address the knowledge gap in archaeal plastisphere ecology, we collected paired plastic debris and adjacent surface-water samples from diverse coastal marine environments and performed both amplicon and metagenomic analyses. Our goal was to characterize archaeal community patterns and their ecological implications in the coastal plastisphere, thereby advancing understanding of archaeal ecology in this anthropogenic habitat and the potential threats posed by plastic pollution. Our discoveries serve to advance the achievement of United Nations Sustainable Development Goals 12 (Responsible consumption and production), 6.6 (protect aquatic ecosystems), and 14.1 (reduce marine pollution). To capture robust, generalizable patterns, sampling was conducted in two seasons (dry and wet), across two regions (subtropical Hong Kong and temperate Qingdao, China), and across various coastal habitat types in each region (beaches, urban river estuaries, mariculture sites, ports, marine reserves, and near wastewater outfalls), encompassing a wide range of environmental conditions and human impacts, and yielding a total of 94 sample pairs (plastisphere versus bulk water) (Supplementary Fig. S1). By sampling directly in the field rather than relying on laboratory or field incubations, we aimed to capture authentic in situ plastisphere patterns that reflected real-world complexity and the high mobility of plastic debris.

## Results and discussion

### The restructured archaeome in the plastisphere

Metagenomic read classification revealed that, across all samples, bacteria dominated the coastal marine microbiome, accounting for ~98–99% of the combined bacterial, archaeal, and fungal reads, whereas archaea and fungi each constituted only ~1% and ~0.2%, respectively (Supplementary Notes S1 and S2 and Figs. S2 and S3). The relatively low archaeal abundance may partly reflect underrepresentation of archaeal genomes in current reference databases, which can lead to underestimation in shotgun-based classifications. Nevertheless, low relative abundance does not necessarily imply low ecological relevance^41,56,57^. We therefore further examined whether plastisphere-associated archaea exhibit distinct taxonomic, assembly, and functional patterns.

**Fig. 1:**
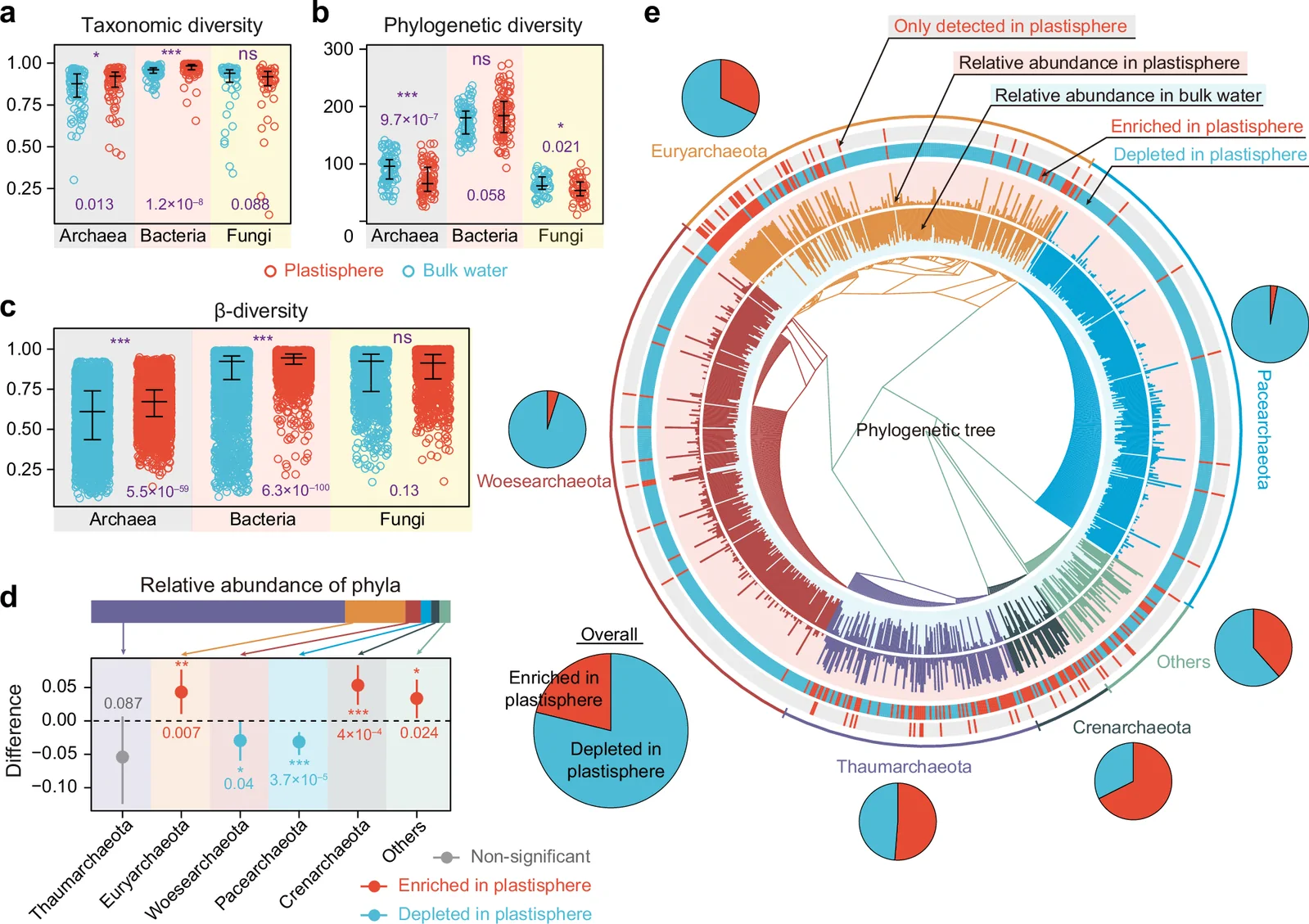
Characteristics of the plastisphere archaeal community. **a**, **b** Changes in archaeal *α*-diversity, including taxonomic diversity quantified by Simpson index **a** and Faith’s phylogenetic diversity **b** between the plastisphere and bulk water. **c** Differences in *β*-diversity based on Bray–Curtis dissimilarities between the plastisphere and bulk water. In **a**–**c** centre bars show medians and error bars show interquartile ranges; significance was assessed using two-sided Wilcoxon rank-sum tests. **d** Relative abundances of main archaeal phyla and their shifts in the plastisphere compared with bulk water. Data are presented as Hodges–Lehmann estimates with 95% confidence intervals and assessed using two-sided Wilcoxon rank-sum tests. **e** Changes in the relative abundance of individual archaeal ASVs in the plastisphere compared to bulk water. The innermost ring shows the phylogenetic relationships among ASVs. Only ASVs with significant differences based on two-sided Wilcoxon rank-sum tests with Holm–Bonferroni adjustment (adjusted *P* < 0.05) and an average read count ≥8 were included. The inner and outer bars of the second ring represent the normalized relative abundances of ASVs in bulk water and the plastisphere, respectively. Colors in the first and second rings denote phylum-level classification. The third ring indicates ASVs enriched (red) or depleted (blue) in the plastisphere, while the fourth ring highlights ASVs detected exclusively in the plastisphere. Pie charts summarize the total abundance of enriched and depleted ASVs in the plastisphere. *n* = 88, 92, and 51 per group for archaeal, bacterial, and fungal analyses, respectively. ns = non-significant, *P < 0.05, **P < 0.01, ***P < 0.001. Exact *P* values are also shown in the figure for statistical comparisons.

Significant differences were observed in archaeal community structure between the plastisphere and surrounding bulk water (Fig. 1; Supplementary Fig. S4). First, archaeal taxonomic α-diversity (Simpson index) was significantly higher in the plastisphere than in bulk water, mirroring patterns observed for bacteria (Fig. 1a). This increase in diversity is likely driven by the unique habitat properties of the plastisphere. Plastic surfaces provide an enriched “resource archipelago” of organic carbon and nutrients that offers microbes refuge, especially under harsh marine conditions^13,15–17,30,58,59^. Weathering creates cracks and pits on plastic surfaces^5^, further enhancing the plastisphere’s protective role. Additionally, due to its mobility and habitat stability, the plastisphere can transport microorganisms over long distances and across ecosystems^24,25,29^; thus, some potentially non-native amplicon sequence variants (ASVs) were observed in the plastisphere (Fig. 1e). These properties of the plastisphere could explain the increased taxonomic diversity of archaea and bacteria (Fig. 1a). In contrast, fungal taxonomic diversity did not increase in the plastisphere (Fig. 1a). This may be because fungi are relatively large organisms for which small plastic crevices provide little refuge; furthermore, fungi typically colonize by extending mycelia, a strategy constrained by the limited surface area of individual plastic particles.

Although taxonomic diversity (reflecting the number and relative distribution of taxa) increased, archaeal phylogenetic diversity (reflecting the evolutionary breadth of the community) decreased in the plastisphere, with a similar pattern also observed for fungi (Fig. 1b). Bacterial phylogenetic diversity also did not show a significant increase in the plastisphere (Fig. 1b). These results suggest that plastisphere habitats selectively recruit phylogenetically related microorganisms that share certain functional traits—for example, the ability to degrade hydrophobic compounds, tolerate low-oxygen conditions, or form biofilms^13,14,26,29^. Such selective filtering could explain the narrowing of phylogenetic breadth despite higher species counts in plastisphere communities. Likewise, archaeal taxonomic β-diversity, based on Bray–Curtis dissimilarity and reflecting community compositional heterogeneity, was greater in the plastisphere (Fig. 1c). This elevated β-diversity could result from high microscale habitat heterogeneity and/or strong dispersal limitation among isolated plastic “islands,” as discussed in the assembly mechanism section below.

The dominant archaeal phyla in the plastisphere were Thaumarchaeota, Euryarchaeota, Woesearchaeota, Pacearchaeota, and Crenarchaeota, together accounting for >97% of all sequences identified as archaea (Fig. 1d). Plastisphere communities showed significant shifts in the relative abundances of these phyla: Euryarchaeota and Crenarchaeota were enriched in the plastisphere (*P* < 0.01), whereas Thaumarchaeota (*P* = 0.087), Woesearchaeota (*P* < 0.05), and Pacearchaeota (*P* < 0.001) were depleted (Fig. 1d; Supplementary Figs. S5 and S6). At finer taxonomic resolution, most ASVs that changed significantly were less abundant in the plastisphere than in bulk water (Fig. 1e). A notable exception was within the Crenarchaeota: many Crenarchaeotal ASVs increased in the plastisphere (Fig. 1e). Crenarchaeota, characterized by their chemoautotrophic metabolism and substantial contributions to marine sediment organic matter^60^, thus appear to benefit disproportionately from the plastisphere habitat. Some ASVs were detected only in plastisphere samples and not in bulk water (Fig. 1e), highlighting the plastisphere’s capacity to harbor archaeal taxa that are not observed in the surrounding water or occur there at abundances below detection.

Using random forest modeling, we identified several archaeal taxa as biomarkers that reliably distinguish plastisphere samples from bulk water (Supplementary Note S3 and Fig. S7). Notably, the ammonia-oxidizing archaeal genus *Nitrososphaera* (phylum Crenarchaeota)^61,62^ emerged as the top biomarker, contributing ~16% of the model’s predictive power and showing strong enrichment in the plastisphere (Supplementary Fig. S7). Genome-resolved metagenomic analysis further supported this pattern: among 23 recovered archaeal metagenome-assembled genomes (MAGs), all five plastisphere-derived MAGs were affiliated with Nitrososphaerales, whereas Poseidoniales MAGs were recovered exclusively from bulk water (Supplementary Note S4 and Figs. S8 and S9). The enrichment of Nitrososphaerales in the plastisphere is consistent with observations from other systems, where *Candidatus* Nitrosocosmicus progressively increased on coastal plastic biofilms during a 39-month colonization experiment^62^ and Nitrososphaeraceae were enriched in the benthic plastisphere of aquaculture ponds^63^, suggesting that the plastisphere may recurrently favor AOA across aquatic systems.

To assess whether these compositional patterns reflect a plastic-specific archaeal signal rather than a general consequence of surface attachment, we performed a meta-analysis of published metagenomic datasets that included both plastisphere and non-plastic solid surfaces. Across studies, plastisphere archaeal communities remained compositionally distinct from those on other solid substrates (Stouffer weighted *Z*-test: *P* < 0.01; Supplementary Notes S5 and S6 and Figs. S10a and S11a), indicating that the observed archaeal community patterns are not merely a generic biofilm effect.

As plastic waste continues to inundate marine environments, the prevalence and concentration of plastisphere-associated archaea will likely increase. This expansion of archaeal biofilms, characterized by higher species richness, greater community heterogeneity, and a distinct compositional profile, could redistribute archaea species and alter overall archaeal diversity and community structure in marine ecosystems. Such shifts may disrupt existing biogeographic patterns, energy flow, and trophic transfer dynamics, potentially triggering cascading ecological effects.

### Biogeochemical cycling implications of the plastisphere archaeome

**Fig. 2:**
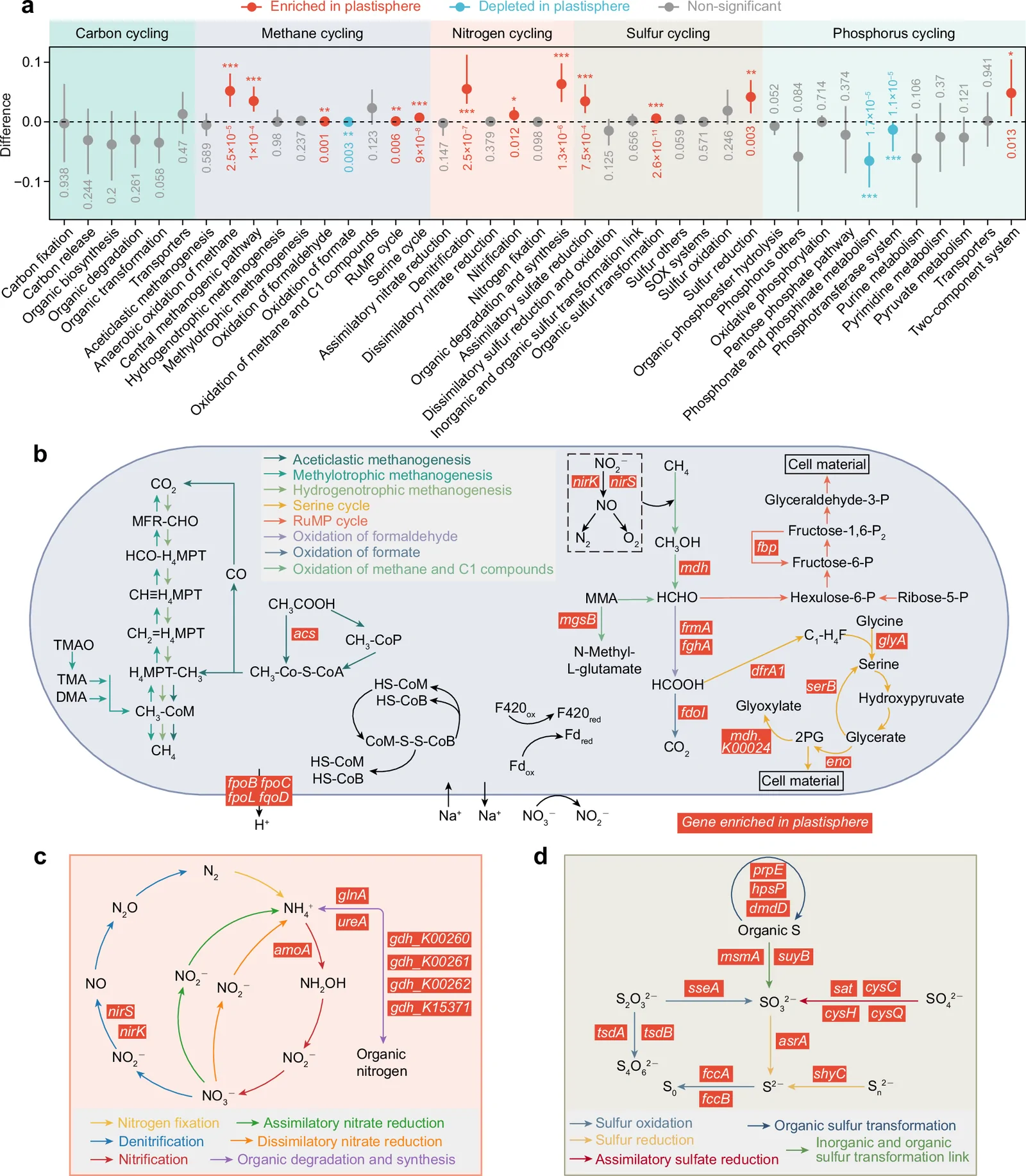
Functional potential of the plastisphere archaeome. **a** Shifts in archaeal functional potential in the plastisphere. Metagenomic assessment of functions associated with carbon, methane, nitrogen, sulfur, and phosphorus cycling. RuMP = ribulose monophosphate, SOX = sulfur oxidation. Points show Hodges–Lehmann estimates and error bars show 95% confidence intervals; significance was assessed using two-sided Wilcoxon rank-sum tests. *n* = 82 per group. *P < 0.05, **P < 0.01, ***P < 0.001. Exact *P* values are also shown in the figure for statistical comparisons. **b**–**d** Detected plastisphere archaeal pathways involved in the transformation of methane **b**, nitrogen **c**, and sulfur **d**. Only genes enriched in the plastisphere are shown (highlighted with red background). For sulfur cycling, only pathways containing genes enriched in the plastisphere are illustrated. See Supplementary Figs. S13–S17 for all detected functional genes and their shifts. TMAO = trimethylamine-N-oxide, TMA = trimethylamine, DMA = dimethylamine, MMA = methylamine, MFR = methanofuran, MPT = methanopterin, Co = Coenzyme, 2PG = 2-phosphoglycerate.

To investigate functional implications of these community shifts, we assessed archaeal biogeochemical potential via metagenomics. The archaeal functional gene profile was significantly altered in the plastisphere relative to bulk water, with distinct differences in functional genes related to methane (CH₄), nitrogen (N), sulfur (S), and phosphorus (P) cycling (Fig. 2a; Supplementary Figs. S12 to S17). This suggests that the plastisphere creates distinct niches associated with archaeal metabolic potential across multiple elemental cycles. We focus here on functions that were enriched in the plastisphere, rather than those reduced, because the plastisphere, its inhabiting microbiome, and the associated ecological impacts are anthropogenic additions to natural ecosystems in which they would otherwise not occur. Notably, many archaeal pathways for CH₄, N, and S cycling were significantly enriched in plastisphere communities (Fig. 2a). Given the global ubiquity of plastics, their continued accumulation, and their capacity to host substantial microbial biomass^15^, these functional enrichments warrant close attention.

For methane cycling, multiple pathways were significantly enriched in plastisphere metagenomes (Fig. 2a, b; Supplementary Fig. S14). These included archaeal pathways for anaerobic oxidation of methane (AOM), the main methanogenesis route (CO₂ reduction to CH₄), oxidation of formaldehyde, and one-carbon assimilation via the serine and ribulose monophosphate (RuMP) cycles. In contrast, the pathway for formate oxidation (a methane-precursor utilization) was less abundant in the plastisphere. Together, these results suggest that plastisphere archaea are equipped to both produce and consume methane. A likely mechanism is that plastisphere biofilms create localized low-oxygen microenvironments relative to the surrounding water column^64,65^. Such conditions could allow methanogenic archaea and anaerobic methanotrophs performing AOM to coexist in close proximity within the biofilm. Accordingly, the concurrent enrichment of genes associated with methanogenesis and AOM suggests the potential for coupled methane production and oxidation in plastisphere biofilms, with possible implications for local CH_4_ dynamics. Ecologically, dispersed plastic debris may therefore represent hotspots of methane turnover in coastal waters, although the magnitude and direction of any net effect remain uncertain. Related studies in soils have linked plastics to elevated CH₄ emissions via microbial shifts^66,67^, highlighting the need to test whether similar outcomes occur in marine plastisphere systems and to quantify the contribution of the plastisphere archaeome.

For nitrogen cycling, genes for both nitrification (e.g., ammonia oxidation to nitrite) and denitrification (stepwise reduction of nitrate/nitrite to N₂O and N₂), as well as genes related to organic nitrogen degradation and synthesis, were enriched in the plastisphere (Fig. 2a, c; Supplementary Fig. S15). This pattern suggests enhanced potential for coupled nitrification–denitrification in the plastisphere archaeome. Plastisphere biofilms likely develop steep oxygen and nutrient gradients^65^, allowing ammonia-oxidizing archaea to inhabit oxygenated outer layers of the biofilm, where they convert ammonia to nitrite^68^. These may then diffuse into deeper low-O_2_ zones that support denitrification^64^. In addition, plastics tend to adsorb organic matter from surrounding media^16,17,59^, providing abundant substrate for nitrogen-transforming microorganisms. Weathered plastic surfaces also acquire negative charges that attract positively charged ions like NH_4_^+^^69^, potentially concentrating ammonia and further benefiting nitrifiers. These features are consistent with previous reports that the plastisphere can function as a nitrification hotspot and may also favor denitrification and N_2_O production^62,64,65,70^. Our genome-resolved analyses further supported this pattern: Nitrososphaerales MAGs were enriched in the plastisphere and carried key nitrogen-cycling genes, including complete *amoABC*, *ureABC*, and *nirK*, consistent with the potential for ammonia oxidation and related nitrogen transformations, including AOA-associated nitrite reduction, in plastisphere biofilms (Supplementary Note S4 and Figs. S8 and S9). Overall, the enrichment of both nitrification- and denitrification-related genes suggests that floating debris may harbor enhanced potential for nitrogen transformations, with possible implications for nitrogen removal, N_2_O-related processes, water quality, the global nitrogen balance, and climate change^38^.

For sulfur cycling, plastisphere archaeal communities exhibited significant enrichment of dissimilatory sulfur reduction pathways, as well as assimilatory sulfate reduction (conversion of sulfate to sulfide for amino acid biosynthesis) and pathways for metabolizing organic sulfur compounds (Fig. 2a, d; Supplementary Fig. S16). The elevated abundance of sulfur reduction genes further indicates that plastisphere biofilms may create localized low-oxygen microenvironments that promote reductive sulfur metabolism, enabling archaea to exploit redox niches that are not available in the surrounding water column. Enrichment of assimilatory sulfate reduction genes further indicates an increased biosynthetic demand, suggesting elevated potential for sulfur assimilation into amino acids and cofactors by plastisphere archaea. In parallel, the enrichment of organic sulfur transformation pathways suggests the potential for plastisphere archaea to degrade organosulfur molecules (e.g., dimethylsulfoniopropionate, DMSP) that may concentrate in the plastisphere. Notably, aquatic plastisphere biofilms have been shown to release dimethyl sulfide (DMS), a volatile breakdown product of DMSP^71,72^. DMS contributes to atmospheric sulfate aerosol formation, which influences cloud properties and climate^73^. Together, these findings suggest enhanced sulfur-cycling potential in the plastisphere. Ecologically, such enhancement may affect both nutrient dynamics and trophic interactions. By assimilating sulfate and transforming organosulfur compounds, plastisphere archaea may alter sulfur partitioning within the plastisphere relative to the surrounding water column. Additionally, DMS associated with plastic biofilms can act as an infochemical: organisms such as seabirds and copepods are attracted by the DMS signal to plastic debris, increasing exposure through mistaken ingestion and thereby potentially generating broader ecosystem impacts^71,74^. In summary, the plastisphere archaeome exhibits enriched sulfur-cycling potential, with possible implications for local sulfur availability, sulfur-mediated ecological interactions, and associated trophic processes.

Consistent with the taxonomic meta-analysis above, a parallel analysis further showed that, despite considerable heterogeneity in study design, combined evidence supported the distinctiveness of plastisphere archaeal functional gene profiles relative to non-plastic substrates (Stouffer weighted *Z*-test: *Z* = 2.35, *P* < 0.01; Supplementary Notes S5 and S6 and Figs. S10b and S11b), suggesting that these functional patterns likewise reflect substrate-specific archaeal responses rather than a general surface-associated biofilm effect.

Overall, plastisphere archaeal communities exhibit enhanced biogeochemical functional potential. Although plastic debris is an inert material, it serves as an important substrate for microbial processes with the potential to accelerate greenhouse gas emissions and modify nutrient cycling pathways. When scaled up to countless plastic particles across large spatial extents, these effects could potentially influence carbon sequestration rates, nitrogen removal dynamics, and nutrient availability in coastal waters. As plastic pollution continues to pervade marine habitats, these functional shifts may accumulate and lead to significant ecological impacts^38,75^. It will be crucial to incorporate the role of the plastisphere into models of coastal biogeochemical cycling, ecosystem health, and climate change mitigation^15,29,55,64,75,76^.

### Defining assembly mechanisms of the plastisphere archaeome

**Fig. 3:**
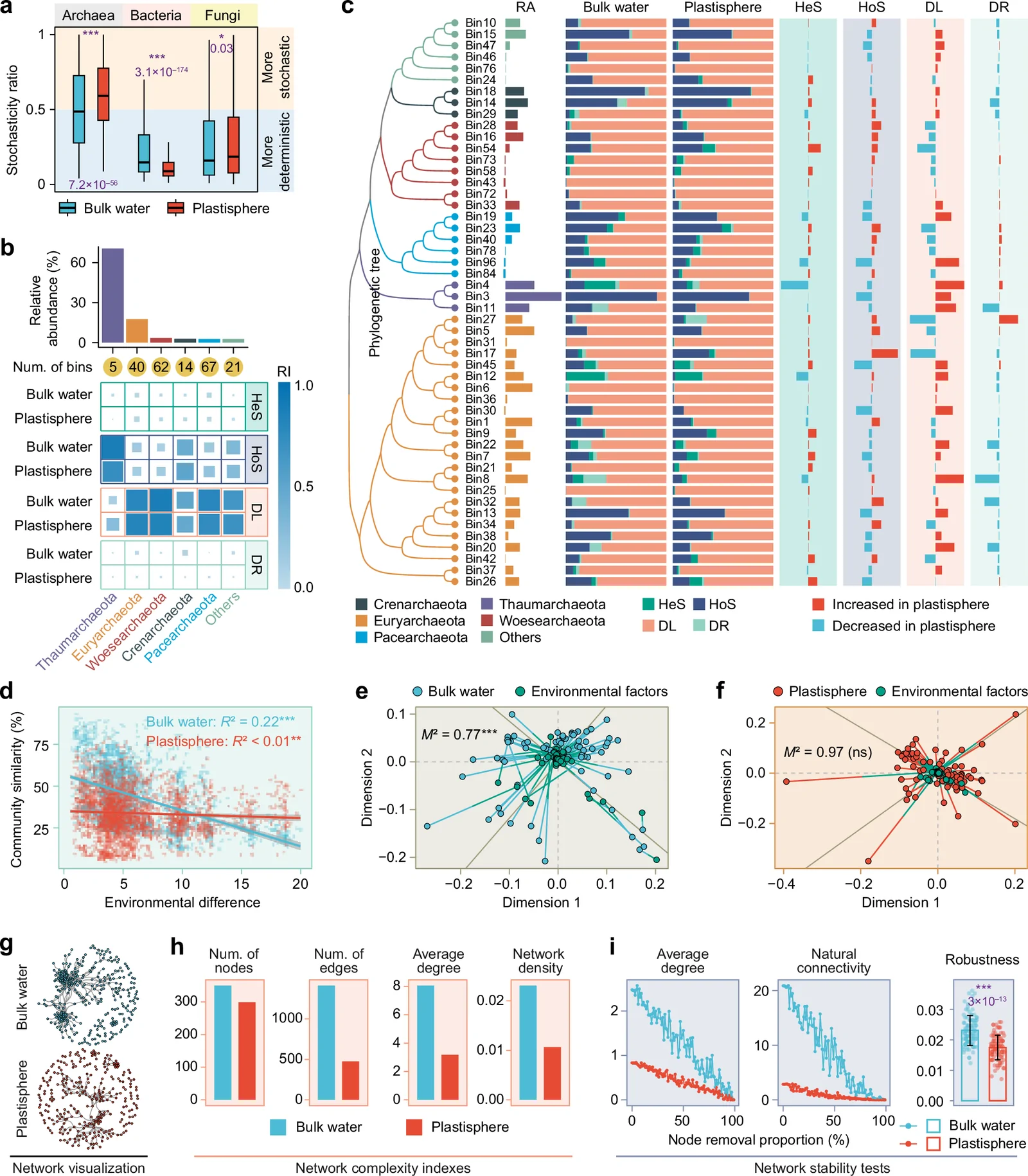
Archaeal community assembly mechanisms in the plastisphere. **a** Stochasticity ratio of archaeal, bacterial and fungal communities in the plastisphere and bulk water. Values > 0.5 indicate stochastic dominance and values < 0.5 indicate deterministic dominance. Box plots show medians, interquartile ranges, and whiskers extending to 1.5 × interquartile range; significance was assessed using two-sided Wilcoxon rank-sum tests. **b** Relative importance of assembly processes across major archaeal phyla, assessed by iCAMP. Bars show phylum-level contributions to overall community assembly; numbers indicate phylogenetic bins assigned to each phylum based on the top member lineage. The heatmap shows process contributions within each phylum and habitat. HeS, heterogeneous selection; HoS, homogeneous selection; DL, dispersal limitation; DR, drift; HD, homogenizing dispersal; RI, relative importance. HD was zero and is not shown. **c** Assembly mechanisms across phylogenetic bins, determined by iCAMP. The leftmost column shows the phylogenetic tree of the top member lineage in each bin. The second column shows relative abundance (RA; ln-transformed ‰) of each bin. Two sets of stacked bar charts (columns 3 and 4) show the relative importance of different processes for each bin in bulk water and plastisphere, respectively. Columns 5–8 indicate processes increased (red) or decreased (blue) in the plastisphere. The 50 most abundant bins are shown, covering >96% of sequences. **d** Relationship between archaeal community similarity (1 − Bray–Curtis distance) and environmental difference (Euclidean distance), with linear regression fits and 95% confidence intervals. **e**, **f** Procrustes analyses relating archaeal community composition to environmental factors in bulk water **e** and the plastisphere **f**. *M*² indicates the sum of squared residuals after optimal alignment; smaller values indicate stronger correspondence. **g** Archaeal co-occurrence networks. Nodes represent ASVs, and edges represent significant co-occurrences. **h** Network complexity metrics. **i** Network stability tests based on random node removal. For robustness, hollow bars show means, error bars show SD, and points show 100 simulations; comparisons used two-sided Wilcoxon rank-sum tests. *n* = 88, 92 and 51 per group for archaeal, bacterial and fungal analyses, respectively. ns, non-significant; *P < 0.05, **P < 0.01, ***P < 0.001. Exact *P* values are shown in the figure.

To disentangle archaeal community assembly in the plastisphere, we first calculated the stochasticity ratio and found striking domain-level contrasts: archaeal assemblages were far more stochastic than those of bacteria or fungi (Fig. 3a). Chance-driven processes such as drift (random changes in community composition) and dispersal limitation (restricted arrival of taxa to local habitats) dominated archaeal composition, while bacteria and fungi were more strongly shaped by environmental filtering and biotic interactions. Archaea generally occur at relatively low abundance in coastal marine environments, comprising ~1% of total metagenomic reads from bacteria, fungi, and archaea combined in this study (Supplementary Fig. S2), and such low-abundance taxa are more susceptible to stochastic colonization and extinction events. Moreover, many archaeal taxa (e.g., Thaumarchaeota and Euryarchaeota) are generalists with broad physiological tolerances (to oligotrophic conditions, variable salinity, pH, etc.)^40,77,78^. This generalist lifestyle reduces the strength of environmental filtering, further favoring stochastic over deterministic processes in archaeal community assembly.

Within archaea, stochastic processes were even more prominent in the plastisphere than in bulk water (Fig. 3a). For archaea, the plastisphere behaves like countless small, fragmented, and isolated islands that amplify dispersal limitation and drift^29^, so the presence or absence of particular archaeal taxa often depends on random chance of arrival and establishment. In this sense, each plastic fragment can be viewed as an isolated habitat patch colonized by whichever archaeal cells happen to reach it, thereby contributing to high between-particle heterogeneity and weaker deterministic filtering.

Despite facing the same isolated island situation, bacteria showed the opposite trend: the assembly in the plastisphere was more deterministic than in bulk water (Fig. 3a). Several factors could contribute to this divergence. First, bacteria are typically more abundant and diverse than archaea in coastal environments, ensuring more continuous dispersal and enabling selective pressures to act effectively, thereby reducing stochastic effects. Second, plastisphere conditions strongly favor deterministic bacterial filtering by consistently selecting for specialists such as biofilm-forming heterotrophs and hydrocarbon degraders^14,29^. Furthermore, the heterogeneity introduced by plastic properties, such as polymer type, additive, and weathering status, imposes strong selective pressure on bacteria, leading to more structured community assembly^79–81^. By contrast, the broad physiological tolerance of archaea reduces the selective impact of such heterogeneity.

Our phylogenetic bin-based iCAMP analysis supports these interpretations. We found that heterogeneous selection, in which different local environments favor different taxa, was minimal for archaeal communities in both plastisphere and bulk water (Fig. 3b, c). Instead, homogeneous selection, in which similar environmental conditions repeatedly favor similar taxa, influenced the most abundant archaeal taxa (e.g., bin3, dominated by Thaumarchaeota, which accounted for >66% of relative abundance) (Fig. 3b, c). Most other archaeal taxa, however, were primarily governed by dispersal limitation (Fig. 3b, c). Importantly, the relative importance of dispersal limitation was significantly greater in the plastisphere archaeal community than in bulk water, further supporting our idea that plastic particles act as island-like habitats for archaeal colonization.

Such enhanced dispersal limitation inherently increases stochastic assembly and can even decouple plastisphere archaeal communities from broader environmental gradients^56,57^. Indeed, plastisphere archaeal community similarity was found to be much less correlated with external physicochemical conditions than bulk-water communities (Fig. 3d). Consistently, Procrustes analysis revealed no significant alignment between plastisphere archaeal community composition and measured environmental variables (Fig. 3f), while bulk-water assemblages showed significant correlations (Fig. 3e).

Biotic interactions among plastisphere archaea also appeared weaker, further underscoring the predominance of neutral processes (Fig. 3g–i). Co-occurrence network analysis showed that the plastisphere archaeal network was simpler and less robust than that of bulk water, with fewer nodes, fewer edges, and lower connectivity (Fig. 3g–i). This suggests that plastisphere archaea form relatively sparse, low-interaction communities. Low network complexity implies limited inter-species cooperation or competition, consistent with communities shaped primarily by stochastic drift rather than tightly organized niches.

Taken together, our results demonstrate that plastisphere archaeal assembly is dominated by stochastic processes, in contrast to the deterministic filtering seen in bacteria and fungi. The island-like nature of plastic debris magnifies random colonization, decoupling archaeal communities from environmental gradients. As a result, plastisphere archaea form simplified, weakly connected networks, underscoring their distinct and chance-driven assembly dynamics.

### Environmental correlates of plastisphere archaeal variation and ecological implications

Although stochastic processes dominated plastisphere archaeal community assembly, this does not preclude environmental variables from being associated with archaeal taxonomic and functional variation. We therefore examined which measured environmental factors were correlated with archaeal community composition and functional profiles, with the aim of identifying environmental correlates and potential modulators of archaeal variation.

**Fig. 4:**
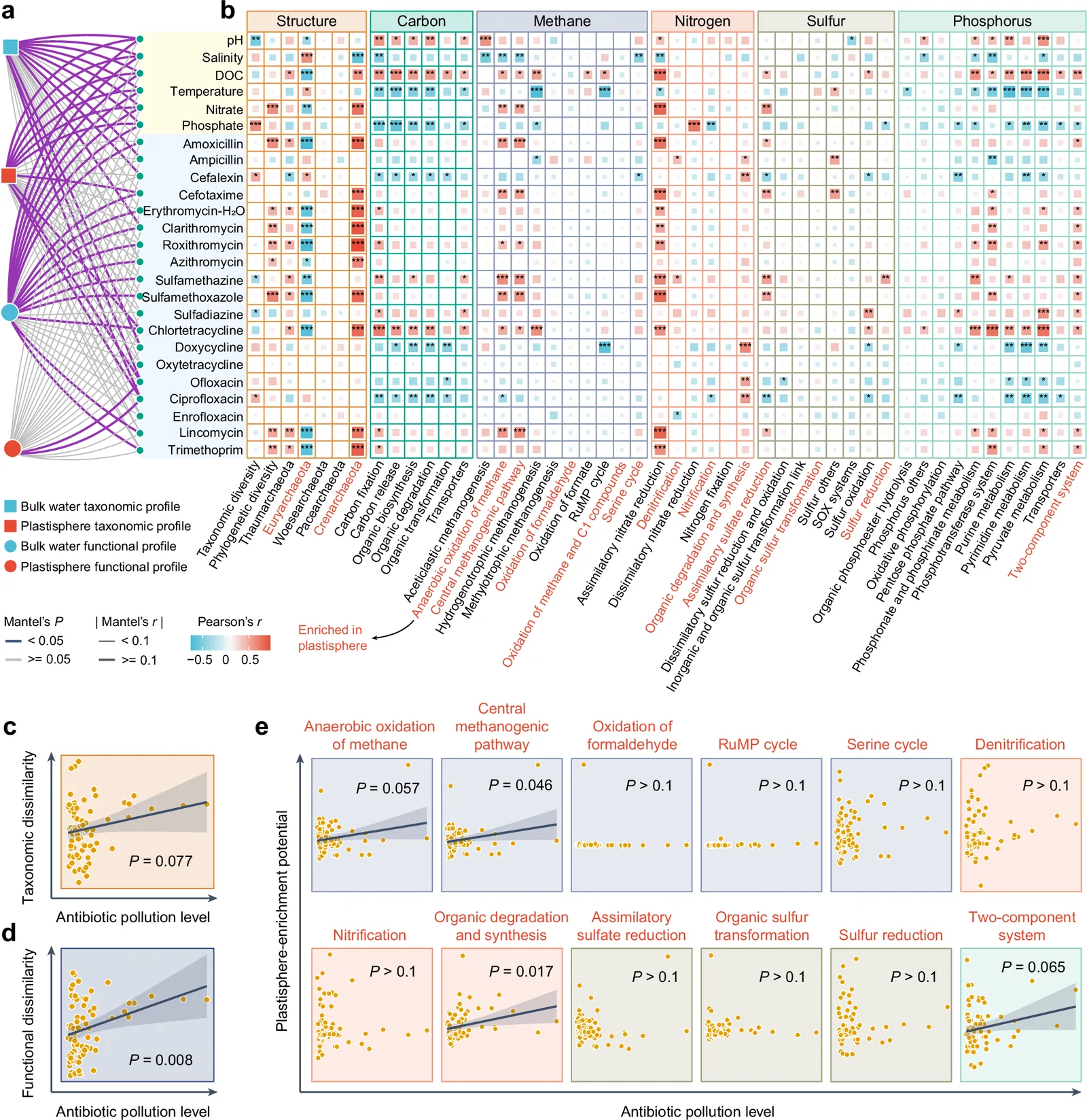
Environmental drivers of archaeal community structure and function. **a** Relationships between environmental variables and archaeal community (taxonomic composition and functional profile) in the plastisphere and bulk water, revealed by Mantel tests. Edge width denotes Mantel’s *r* value and edge color indicates statistical significance. **b** Correlations between environmental variables and the differences in archaeal community metrics between the plastisphere and bulk water (plastisphere value – bulk water value). Cell color corresponds to Pearson’s *r* value; significance was assessed using two-sided Pearson correlation tests. DOC = dissolved organic carbon. See Supplementary Information for correlations between environmental variables and archaeal community metrics in the plastisphere (Supplementary Fig. S18) and bulk water (Supplementary Fig. S19). **c**–**e** Relationships between archaeal taxonomic dissimilarity (Bray–Curtis dissimilarity between plastisphere and bulk water) and antibiotic pollution level (indexed as the sum of all measured antibiotics, each standardized 0–1) **c**, between functional dissimilarity and antibiotic pollution level **d**, and between the enrichment potential of plastisphere-enriched functional pathways (plastisphere value – bulk water value) and antibiotic pollution level **e**. Lines and shaded bands show linear regression fits with 95% confidence intervals; significance was assessed using two-sided tests of regression slopes. Only when *P* < 0.1 are the linear regression trendline and confidence interval displayed. *P < 0.05, **P < 0.01, ***P < 0.001.

Mantel tests revealed that several environmental gradients—including dissolved organic carbon (DOC), nitrate, phosphate, pH, salinity, temperature, and certain antibiotics—were significantly correlated with archaeal community composition in both plastisphere and bulk water (Fig. 4a). However, bulk water archaeal communities showed significant associations with a wider array of antibiotic variables than plastisphere communities did (Fig. 4a). We found a similar pattern for archaeal functional potential: in bulk water, most measured environmental factors were significantly associated with the overall archaeal functional gene profile (Fig. 4a). By contrast, the archaeal functional profile in the plastisphere was significantly correlated with only two antibiotic concentrations and showed no detectable relationships with the other 23 environmental parameters (Fig. 4a).

The plastisphere’s functional composition thus appears less sensitive to environmental variation, suggesting a relatively stable, and potentially functionally redundant, metabolic repertoire within plastisphere communities, as well as a possible buffering effect of the plastisphere microenvironment for microorganisms under harsh and fluctuating environmental conditions. Such stability may be due to high functional redundancy among plastisphere archaea, meaning multiple taxa perform overlapping metabolic roles that buffer community function against changing conditions^82^. This observation complements our finding that plastisphere archaea exhibit higher taxonomic diversity but lower phylogenetic diversity (Fig. 1a, b), consistent with a functionally redundant community composed of relatively close relatives.

To further probe the influence of anthropogenic stress, we examined how differences between plastisphere and bulk-water archaeal communities relate to pollution indicators (Fig. 4b; Supplementary Figs. S18 and S19). We found that the magnitude of difference between plastisphere and bulk communities in several metrics—specifically archaeal α-diversity, the relative abundance of Euryarchaeota (enriched in the plastisphere), and the abundances of key metabolic pathways (CH₄, N, and P cycling)—tended to be greater in more polluted waters (i.e., those with higher DOC and higher concentrations of multiple antibiotics) (Fig. 4b). Consistently, linear regression analyses showed that both the taxonomic dissimilarity (Fig. 4c) and functional dissimilarity (Fig. 4d) between plastisphere and bulk communities increased with antibiotic pollution level. Notably, several plastisphere-enriched archaeal functional pathways, such as anaerobic oxidation of methane, the core methanogenic pathway, and organic nitrogen degradation/synthesis, appeared to be enhanced under high levels of antibiotic pollution (Fig. 4e).

These findings indicate that under higher environmental stress, plastisphere archaeal communities deviate more strongly from those in bulk water. One plausible explanation is that the plastisphere provides a sheltered microhabitat in which resident microorganisms are buffered, to some extent, from external chemical stress, whereas bulk-water archaeal communities are more directly exposed to surrounding conditions^23^. Under this scenario, increasing stress would be expected to reshape bulk-water communities more strongly than plastisphere communities, thereby amplifying the taxonomic and functional divergence between them. Overall, increasing anthropogenic chemical stressors appear to be associated with more distinct plastisphere archaeal assemblages and greater plastisphere–bulk-water divergence in functional potential. These results suggest that, where chemical pollution co-occurs with plastic debris, the plastisphere may become an increasingly distinct and ecologically important microbial habitat.

### Final remarks

The plastisphere represents a novel human-made habitat within the ocean microbiome that may redistribute microbial taxa and alter biogeochemical functional potential in marine systems. Recognizing these shifts is important for predicting the broader environmental impacts of plastic debris beyond the well-known direct hazards to wildlife. Here, we show that plastics host a restructured archaeal community characterized by higher taxonomic diversity and greater heterogeneity than the surrounding seawater. Correspondingly, archaeal functional gene profiles differ between the plastisphere and bulk water, with enrichment of pathways related to CH_4_, N, and S cycling in the plastisphere, highlighting the potential for plastic pollution to influence biogeochemical cycling^38,83^ and climate-related processes^84^ from an archaeal perspective. As plastic waste continues to accumulate globally, the plastisphere will expand accordingly, leading to greater prevalence and density of archaeal communities in marine ecosystems, and thereby amplifying their ecological impacts.

We also found that stochastic processes predominantly govern archaeal community assembly in the plastisphere, underscoring the complexity of predicting these communities and the difficulty of mitigating their impacts through environmental management alone. Therefore, effectively addressing the impacts of plastic pollution will require upstream solutions such as reducing plastic production, improving recycling and reuse^85^, advancing waste treatment technologies, and actively cleaning up existing plastic waste^6^. It is also important to identify the locations and time periods that should be prioritized for waste control and management, namely those in which plastic pollution is exerting particularly strong ecological impacts. An ambitious global plastic treaty could also help mitigate these impacts^6,15,86^. Beyond the context of plastic pollution, these results help clarify how microorganisms respond to the expansion of anthropogenic microbial habitats in the Anthropocene^27^.

Nevertheless, because our analyses are based on taxonomic composition and metagenomic functional inference, direct links to viability, in situ activity, and realized biogeochemical rates remain to be established. In addition, the ecological implications highlighted here are based on relative comparisons rather than direct measurements of impact magnitude. For example, we still do not know to what extent plastic pollution contributes to microbial redistribution and invasion in the ocean, nor what magnitude of greenhouse-gas-related effects may be associated with plastisphere archaea. Further research is needed to directly link these microbial shifts to real-world ecological and health outcomes of plastic pollution, and to quantitatively assess the full risks of the plastisphere. Addressing these questions will require cross-disciplinary collaboration among researchers in environmental microbiology, environmental impact assessment, public health, and data science. Moreover, our study only considered surface waters and significant knowledge gaps remain regarding plastisphere dynamics throughout the water column and deep ocean ecosystems. Future work should also investigate the ecological mechanisms underlying plastisphere archaeal community assembly, including the roles of biofilm succession, substrate heterogeneity, and dispersal dynamics, to advance predictive understanding of how plastic pollution reshapes microbial diversity and function in marine ecosystems.

## Methods

### Sample collection

We collected paired plastic debris and corresponding surface water samples along the coasts of Hong Kong and Qingdao, China, in August 2021 and February 2022 (Supplementary Fig. S1). Floating plastic debris was collected using a Manta trawl (333 μm mesh) towed behind a boat. After approximately 30 minutes of trawling, all visible plastic pieces were picked out with sterilized tweezers from the material gathered in the net’s collection cup and stored in sterile tubes. At least three replicate trawls were conducted at each site. Two liters of surface seawater were collected alongside each plastic sample as the corresponding bulk water sample. All samples were immediately stored on ice in the field, transported to the lab on the same day, and kept at 4 °C until further processing. In total, we obtained 94 plastic debris samples and corresponding paired 94 bulk water samples.

### Determination of plastic identity

All collected debris particles were visually identifiable as plastic (Supplementary Fig. S20). To confirm their identity, we randomly selected 62 particles for polymer analysis using optical photothermal infrared (O-PTIR) spectroscopy. Each particle was mounted on a glass slide without cleaning or chemical treatment, and an IR spectrum was obtained. The spectra were matched against the KnowItAll (Wiley) spectral library to identify polymer types. All tested particles were confirmed to be plastic, each with a minimum match score >70% (Supplementary Fig. S21 and Table S1).

### Detection of environmental parameters

Water temperature, pH, and salinity were measured in situ using a YSI multi-parameter probe. One liter of each water sample was filtered through a 0.45 μm membrane for subsequent analyses of nutrients and organic carbon. Dissolved organic carbon (DOC) was measured with an Elementar Acquray TOC analyzer. Nitrate and phosphate concentrations were determined with a Skalar San++ continuous flow analyzer. Nineteen types of antibiotics were measured (as indicators of human-derived chemical pollution) following established protocols^87^. These antibiotics included amoxicillin, ampicillin, azithromycin, cefalexin, cefotaxime, chlortetracycline, ciprofloxacin, clarithromycin, doxycycline, enrofloxacin, erythromycin-H₂O, lincomycin, ofloxacin, oxytetracycline, roxithromycin, sulfamethazine, sulfadiazine, sulfamethoxazole, and trimethoprim (Supplementary Table S2).

### DNA extraction, amplification, and sequencing

For each plastic sample, we extracted DNA from the combined plastic debris (pooling all plastic pieces in that sample) rather than from individual particles, in order to capture a general community profile. This approach aligns with the “microplastome” framework we previously proposed^88^. We adapted a membrane-filtration DNA extraction method^89^: briefly, each plastic debris sample was sonicated multiple times in sterile 1× PBS on ice to dislodge microbes from surfaces and pores, and the PBS wash was filtered through a 0.22 μm membrane to collect the cells. For each water sample, 1 L of water was filtered through a 0.22 μm membrane to collect cells. DNA from all membranes (plastisphere and water samples) was extracted using the QIAamp DNA Mini Kit (QIAGEN) according to the manufacturer’s instructions. DNA extracts were stored at –80 °C until use.

### Amplicon sequencing and processing

Archaeal, bacterial, and fungal communities were profiled based on amplicon sequencing. For archaea, we amplified the 16S rRNA gene using archaea-specific primers Arch519F/Arch915R, which are suitable for capturing archaeal diversity even when archaea occur at low relative abundance^90^. For bacteria, the 16S rRNA gene (V3–V4 region) was amplified with primers 341F/806R, and for fungi, the ITS2 region with primers ITS3-2024F/ITS4-2409R. PCR products were purified with Agencourt AMPure XP beads and eluted in buffer. Sequencing libraries were quality-checked on an Agilent 2100 Bioanalyzer, then paired-end sequenced (2 × 300 bp) on a DNBSEQ-2000 platform.

Raw amplicon sequences were processed using an open-source pipeline (EasyAmplicon)^91,92^ based on USEARCH/VSEARCH. Briefly, paired-end reads were merged and quality-filtered (with VSEARCH’s --fastq_mergepairs and --fastx_filter), then dereplicated (derep_fulllength). Denoising was performed with the UNOISE3 algorithm in USEARCH to generate ASVs. An ASV table (read counts per ASV per sample) was created by mapping the reads back to the ASVs using VSEARCH (usearch_global). Taxonomy was assigned to archaeal and bacterial ASVs using the RDP classifier, and to fungal ASVs using the UNITE database.

For internal consistency with the marker-gene analysis, archaeal taxonomy from amplicon data is reported using the SILVA/RDP framework, whereas genome-based GTDB taxonomy is used separately for MAG analyses. These two frameworks use different classification boundaries and nomenclature, reflecting distinct underlying methodologies: rRNA-based marker-gene classification versus genome phylogeny-based taxonomy. Consequently, some archaeal higher-level names differ between the amplicon and MAG analyses and cannot be reconciled by simple one-to-one name replacement. For example, many lineages historically assigned to Thaumarchaeota in rRNA-based frameworks correspond broadly to Nitrososphaeria within Thermoproteota in GTDB; Crenarchaeota-related rRNA lineages are also reorganized within Thermoproteota-related genome-based taxonomy; traditional Euryarchaeota *sensu lato* has been split across multiple GTDB phyla/lineages, including Thermoplasmatota/Poseidoniales (Marine Group II) and other groups; and Woesearchaeota and Pacearchaeota correspond to DPANN/Nanobdellati-related lineages with revised boundaries in genome-based taxonomy. We therefore retained SILVA/RDP names for amplicon-derived ASV analyses and used GTDB names for genome-resolved MAG analyses, while providing key cross-references between the two frameworks where relevant.

To normalize sequencing depth across samples, we rarefied the archaeal dataset to 8,000 reads per sample, the bacterial dataset to 30,000 reads, and the fungal dataset to 5,700 reads. Samples with fewer reads than these thresholds were removed. Additionally, any sample lacking its paired plastic or water counterpart was removed to maintain a 1:1 plastisphere–bulk pairing. This yielded 88 paired plastisphere and water samples for archaea (176 samples total), 92 pairs for bacteria, and 51 pairs for fungi. According to rarefaction curves, our sequencing depth and sample counts were sufficient to capture the dominant microbial diversity in both plastisphere and bulk communities (Supplementary Fig. S22).

To contextualize methodological influences on archaeal detection in 16S-based surveys, we also re-examined a previously published bacterial-focused amplicon dataset from our group^30^; the corresponding methods and results are provided in Supplementary Note S2 and Fig. S3.

### Metagenomics and functional gene identification

For shotgun metagenomics, we constructed low-input libraries from 25 to 100 ng of DNA per sample. Several samples failed library construction due to insufficient DNA, but all remaining samples were sequenced on the DNBSEQ-2000 platform (150 bp paired-end), generating ≥10 Gb of data per sample. We excluded any metagenomes that lacked corresponding amplicon data or a paired sample. This yielded 82 paired plastisphere and water metagenomes (164 total metagenomic samples).

Raw reads were processed with the EasyMetagenome pipeline^93^. Reads were quality-controlled with fastp v0.23.2 (default parameters) and assembled using MEGAHIT v1.2.9. Open reading frames (ORFs) were predicted on assembled contigs using Prodigal v2.6.3. The ORFs were clustered at 95% identity (≥ 90% coverage) with CD-HIT v4.8.1 to construct a non-redundant gene catalog. Quality-filtered reads were mapped back to the gene catalog, and gene abundances were calculated as transcripts per million (TPM) using Salmon v1.10.1.

To provide cross-domain context for archaeal analyses, we quantified the relative contributions of archaea, bacteria, and fungi across all metagenomic samples. Reads were classified against a custom database integrating Genome Taxonomy Database (GTDB) release 207 species-representative genomes for bacterial and archaeal classification, and the RefSeq-based PlusPF database for fungal classification. Domain-level read counts were extracted and normalized to calculate relative proportions across the three domains. Detailed methods and results are provided in Supplementary Note S1.

Taxonomic annotation for each gene in the catalog was determined using GTDB via MMseqs2. From the full gene catalog (and corresponding abundance matrix), we extracted all archaeal-affiliated sequences to create an archaeal-only gene catalog and abundance table. The abundance of each archaeal gene in a sample was then normalized to the total archaeal gene abundance of that sample (yielding relative abundances of each archaeal gene). We next mapped the archaeal gene catalog against five curated databases of biogeochemical cycling genes—CCycDB^94^, MCycDB^95^, NCycDB^96^, SCycDB^97^, and PCycDB^98^ (for carbon, methane, nitrogen, sulfur, and phosphorus cycling, respectively)—using DIAMOND v2.1.6 with an e-value threshold of 1 × 10^−5^.

### Archaeal MAG characterization

Metagenomic contigs from all metagenomic samples were assembled and binned using metaWRAP v1.3.2^99^. Resulting bins were dereplicated using dRep v3.6.2 with an average nucleotide identity threshold of 95% (-sa 0.95) and a minimum coverage of 30% (-nc 0.30), while retaining bins with completeness ≥50% and contamination ≤10% (-comp 50 -con 10). Dereplicated MAGs were taxonomically classified using GTDB-Tk v2.1.0 against the GTDB database, based on 120 bacterial marker genes (bac120) and 53 archaeal marker genes (ar53). A total of 23 archaeal MAGs were recovered, affiliated with two orders: Nitrososphaerales (*n* = 11) and Poseidoniales (*n* = 12).

A phylogenetic tree of the 23 archaeal MAGs was constructed using the multiple-sequence alignment of ar53 marker genes generated by GTDB-Tk. Protein-coding genes were predicted from each archaeal MAG using Prodigal v2.6.3 in metagenomic mode (-p meta). Predicted protein sequences were searched against NCycDB^96^ using DIAMOND blastp with the following parameters: e-value ≤ 1 × 10^−5^, amino acid identity ≥40%, and query coverage ≥70%.

To quantify the abundance of each archaeal MAG across samples, protein-coding gene sequences from all 23 archaeal MAGs were pooled and indexed using Salmon v1.10.1. Quality-filtered reads from each sample were mapped to the index using salmon quant. TPM values of all genes within each MAG were summed to generate per-MAG abundance estimates for each sample.

### Metagenomic sample collection and processing for comparison between plastic and other solid surfaces

To assess whether the plastisphere represents an archaeal habitat distinct from other solid surfaces, rather than reflecting a general feature of surface-attached biofilms, we performed a meta-analysis of publicly available marine metagenomic datasets. Potentially relevant studies were identified through a Web of Science search in November 2025 using the query: ALL = (microplastic OR microplastics OR plastic OR plastics OR plastisphere) AND ALL = (metagenomic OR metagenomics OR metagenome OR shotgun sequencing OR whole genome OR whole-genome sequencing). Search results were screened manually to identify studies meeting the following criteria: (i) inclusion of plastisphere samples together with other solid-surface samples; (ii) sampling from marine environments; and (iii) availability of raw metagenomic sequencing data. After screening, 99 metagenomic samples from seven studies were retained (Supplementary Tables S3 and S4). These datasets were processed using the same analytical workflow as described above, including quality control, assembly, taxonomic annotation against GTDB, and functional gene annotation and quantification using CCycDB^94^, MCycDB^95^, NCycDB^96^, SCycDB^97^, and PCycDB^98^.

### Statistical analyses

To test whether plastisphere archaeal communities and functional profiles differ from those in the surrounding environment, we performed adjusted principal coordinates analyses (aPCoA) on Bray–Curtis dissimilarity matrices for community composition (ASV level) and for functional gene profiles, using the aPCoA R package. Statistical significance of community differences was assessed via PERMANOVA. Taxonomic α-diversity was quantified with the Simpson index, and phylogenetic α-diversity with Faith’s phylogenetic diversity (using the vegan and picante R packages, respectively). To evaluate β-diversity (community heterogeneity), we compared Bray–Curtis dissimilarities among samples within each group (plastisphere vs. bulk water). We constructed random forest models at multiple taxonomic levels (phylum, class, order, family, genus) using the randomForest R package to identify biomarkers distinguishing plastisphere vs. bulk-water archaeal communities (Supplementary Note S3 and Table S5).

To infer community assembly mechanisms underlying the unique plastisphere archaeal community, we applied two null-model approaches: the modified stochasticity ratio (MST)^100^ and a phylogenetic-bin-based null model (iCAMP)^101^, using the NST^100^ and iCAMP^101^ R packages, respectively. Both approaches rely on generating a null expectation by randomizing community composition while preserving species richness and total abundance^100,101^. Then the observed community patterns were compared to the null expectations: communities more similar or more dissimilar than the null expectation were attributed to deterministic processes, whereas those within null expectations were attributed to stochastic processes. The MST summarizes this balance, with values < 0.5 indicating deterministic dominance and >0.5 indicating stochastic dominance. The iCAMP analysis provides a more detailed breakdown of five specific assembly processes (homogeneous selection, heterogeneous selection, dispersal limitation, homogenizing dispersal, and drift). The 9744 observed ASVs were divided into 209 phylogenetic bins according to a phylogenetic signal threshold (0.2) and minimal bin size (12). The relative importance of five assembly processes was calculated, and the dominant taxa contributing to assembly processes were evaluated in each bin. Homogeneous and heterogeneous selection are categorized as deterministic processes, while the other three are stochastic. We quantified the relative importance of these processes in governing archaeal community assembly in the plastisphere versus bulk water.

To evaluate how strongly environmental divergence drives community divergence in the plastisphere and bulk water, we performed linear regression analyses relating archaeal community similarity (1 – Bray-Curtis distance) to differences in environmental conditions (Euclidean distance of environmental variables) for plastisphere and bulk samples separately. Procrustes analysis was used to further assess the relationship between the environmental variables and archaeal community in bulk water and in the plastisphere.

To assess the influence of species interactions on community structure, we constructed archaeal co-occurrence networks for the plastisphere and bulk water. ASVs present in fewer than 10% of samples were excluded to minimize false-positive links. Spearman correlations between ASVs (*ρ* > 0.6, *P* < 0.05) were considered significant co-occurrence relationships and were included as edges in the networks (visualized using the igraph R package). We calculated network topological parameters (number of nodes, number of edges, average degree, and network density) using the ggClusterNet package. To compare network stability, we performed simulated node removal experiments: we randomly removed an increasing proportion of nodes (from 0% to 100%) and recalculated the average degree and natural connectivity after each removal, comparing the robustness of plastisphere vs. bulk networks. We also measured robustness as the resistance to removing 50% of the nodes (averaged over 100 random repetitions for each network).

To explore potential environmental drivers of the plastisphere archaeome, we used Mantel tests to examine the relationships between environmental variables and archaeal community attributes (taxonomic composition and functional profile) in the plastisphere and bulk water. We also calculated Pearson correlations to examine how environmental variables correlated with various archaeal community metrics (α-diversity, major phylum abundances, and biogeochemical pathway gene abundances). The correlation results were visualized as heatmaps for the plastisphere and bulk communities. To evaluate the impact of pollution on archaeal community and functional divergence between the plastisphere and bulk water, we calculated an antibiotic pollution index for each site by summing the concentrations of the 19 antibiotics (each concentration standardized from 0 to 1). Linear regressions were then conducted to relate antibiotic pollution level to archaeal taxonomic differences (Bray–Curtis dissimilarity between plastisphere and bulk water), functional differences (Bray–Curtis dissimilarity between plastisphere and bulk water), and the enrichment of plastisphere-enriched functional pathways (plastisphere value minus bulk water value).

To assess whether plastisphere archaeal communities were compositionally and functionally distinct from those on other solid surfaces, we analyzed published marine metagenomic datasets containing both plastisphere and non-plastic surface samples. Within each study, Bray–Curtis dissimilarity was used to compare taxonomic composition and archaeal functional gene profiles, and significance was evaluated using permutation tests. Evidence across studies was then combined using Stouffer’s weighted *Z*-method. Full analytical details are provided in the Supplementary Notes S5 and S6.

### Reporting summary

Further information on research design is available in the Nature Portfolio Reporting Summary linked to this article.

## Supplementary information

Supplementary Information

Reporting Summary

Transparent Peer Review file

## Source data

Source Data

## Data Availability

Amplicon sequencing data have been deposited in National Center for Biotechnology Information (NCBI) under BioProject IDs PRJNA1307274 (archaea), PRJNA1307280 (bacteria), and PRJNA1307281 (fungi). Shotgun metagenomic sequences are available under BioProject ID PRJNA1307277. Source data are provided with this paper.

## Code availability

R scripts for statistical analyses are available at GitHub https://github.com/Changchao-Li/plastisphere_archaea^102^. The EasyAmplicon pipeline for the front-end processing of amplicon sequences can be found at https://github.com/YongxinLiu/EasyAmplicon. The EasyMetagenome pipeline for the front-end processing of metagenomic sequences can be found at https://github.com/YongxinLiu/EasyMetagenome.
