# Supplementary material for: Unraveling the coastal marine plastisphere archaeome
Source: Nat Commun. 2026 Aug 14;17:9780. doi: 10.1038/s41467-026-76782-z (PMC13572444; doi:10.1038/s41467-026-76782-z)
Supplement: Supplementary file 1 [file 41467_2026_76782_MOESM1_ESM.pdf]

Supplementary Information for

Unraveling the coastal marine plastisphere archaeome

Changchao Li[1,2,#,*], Yijing Wang[3,4,#], Albert Shing King Zhou[5], Xi Pan[1], Yanjie Zhu[4], Xiaohua Zhang[1], Tian Chen[1], Anqi Xiong[1], Yuen-Wa Ho[6], Jian Liu[3], Zhichao Zhou[7], Jie Wang[8], Tanveer M. Adyel[9], James Kar-Hei Fang[2,6,10,11*], Michael S. Bank[12,13], Matthias C. Rillig[4], Ling N. Jin[1,10,11,14,*]

*** Correspondences:**

C. Li (changchao.li@polyu.edu.hk);
J.K.H. Fang (james.fang@polyu.edu.hk);
L.N. Jin (ling.jin@polyu.edu.hk)

This PDF file includes:

- Supplementary Notes S1 to S6
- Supplementary Figures S1 to S22
- Supplementary Tables S1 to S5

Supplementary Notes

Note S1 Relative abundance of archaea, bacteria, and fungi in the plastisphere

To contextualize the ecological significance of archaea within the broader microbial community, we quantified the relative abundances of the three domains—archaea, bacteria, and fungi—across all plastisphere and seawater samples using a dual-database Kraken2 classification strategy. For bacterial and archaeal classification, a custom database was constructed using GTDB release 207 representative genomes, comprising 62,291 bacterial and 3,412 archaeal species-representative genomes selected from 311,480 bacterial and 6,062 archaeal total genomes. For fungal classification, the Kraken2 PlusPF database, which incorporates RefSeq fungal reference genomes, was used. All metagenomic reads were classified independently against both databases in paired-end mode, and reads assigned to non-target groups were discarded.

Domain-level relative abundances were calculated as follows. For each sample, bacterial and archaeal read counts were extracted from the GTDB-based Kraken2 report at the domain rank, whereas fungal read counts were extracted from the PlusPF-based report at the kingdom rank. To account for differences in total read counts between the two databases, the proportion of each domain relative to the total reads in its respective database was first calculated, and the three proportions were then normalized to sum to 100%.

As expected, bacteria dominated both seawater and plastisphere communities, accounting for 98.96% (96.32%–99.91%) and 98.66% (89.21%–99.94%) of the combined three-domain reads, respectively (Supplementary Fig. S2). Archaea and fungi each represented minor but consistent fractions of the total community (Supplementary Fig. S2). Archaea comprised 0.94% (0.07%–3.61%) of reads in bulk water and 1.03% (0.03%–10.08%) in plastisphere communities, whereas fungi accounted for 0.10% (0.02%–0.56%) and 0.31% (0.02%–2.70%), respectively (Supplementary Fig. S2). The relatively low archaeal read proportions may also partly reflect the still less complete representation of archaeal genomes in current reference databases compared with bacteria, which can lead to systematic underestimation of archaeal reads in shotgun-based classification. The use of a GTDB-based database, which provides substantially broader archaeal genome coverage than standard NCBI RefSeq-based Kraken2 databases (3,412 versus approximately 800–1,000 archaeal representative genomes), was intended to mitigate this bias and improve archaeal abundance estimates.

These data confirm that, although archaea constitute a numerically minor fraction of the total microbial community, they are consistently present across all samples and habitats. In microbial ecology, low relative abundance does not necessarily imply low ecological importance. For example,

marine ammonia-oxidizing Thaumarchaeota drive a substantial fraction of global oceanic nitrification despite comprising only a minor fraction of total prokaryotic cells[1, 2], and the "rare biosphere" concept recognizes that low-abundance taxa can maintain important ecosystem functions and resilience[3-6]. The domain-level abundance data are therefore provided here as contextual background rather than as a direct measure of ecological importance. The primary analyses in the main text, including archaeal community structure, diversity, assembly, and functional potential, are based on archaeal-targeted amplicon sequencing and archaeal-specific metagenomic analyses designed to resolve archaeal ecology at appropriate resolution.

Note S2 Recovery of archaeal reads from bacterial-targeted 16S amplicon data

To further illustrate that the limited representation of archaea in the plastisphere literature may partly reflect methodological constraints rather than true ecological absence, we re-examined a previously published dataset from our group[7] in which 16S rRNA gene amplicon sequencing had been performed using the 515F/806R primer pair with a bacterial focus. When archaeal reads were retained rather than discarded during taxonomic assignment, archaeal sequences were recovered at detectable proportions across all samples.

Notably, the plastisphere group showed a higher archaeal proportion (~3.7%) than the seawater group (Supplementary Fig. S3), consistent with the view that plastic surfaces can selectively concentrate archaeal taxa. These results are presented here only as a methodological illustration and do not constitute new findings of the present study. Instead, they support the interpretation that the apparent scarcity of archaea in many 16S-based plastisphere surveys may partly reflect under-detection arising from primer bias and downstream analytical choices, rather than true ecological absence. By contrast, archaeal reads were not recovered from the bacterial-targeted 341F/806R amplicon dataset generated in the present study, which is also consistent with the known primer-dependent variation in archaeal coverage.

Note S3 Archaeal biomarkers of the plastisphere

To identify archaeal features that best distinguish the plastisphere from bulk water, we applied random-forest machine-learning models to archaeal community data at multiple taxonomic levels, including phylum, class, order, family, and genus, using the randomForest package in R.

Among these models, the genus-level model showed the highest predictive performance, explaining 85% of the variation between plastisphere and bulk-water archaeal communities (Supplementary Table S5), further supporting the marked compositional differentiation of the plastisphere from the surrounding natural habitat. Model performance and feature importance were evaluated using five-fold cross-validation with five repeated runs. We then selected the 20 archaeal taxa that contributed most strongly to model accuracy as plastisphere biomarkers (Supplementary Fig. S7a).

These biomarker taxa belonged to five phyla. Of the 20 biomarkers, 11 were enriched in the plastisphere, namely *Nitrososphaera*, *Halococcus*, *Fervidicoccus*, *Halogranum*, *Methanolobus*, *Methanogenium*, *Halovivax*, *Halomarina*, *Natrialba*, *Halostagnicola*, and *Natrinema*, whereas 9 were depleted, namely Woesearchaeota Incertae Sedis AR18, Pacearchaeota Incertae Sedis AR13, *Nitrosopumilus*, *Methanospirillum*, Woesearchaeota Incertae Sedis AR15, *Methanocorpusculum*, Woesearchaeota Incertae Sedis AR20, Woesearchaeota Incertae Sedis AR16, and *Methanoplanus* (Supplementary Fig. S7b).

Notably, *Nitrososphaera* emerged as the single most important biomarker, consistent with the genome-resolved metagenomic results showing enrichment of Nitrososphaerales MAGs in the plastisphere (Supplementary Figs. S8 and S9). Together, these results indicate that ammonia-oxidizing Nitrososphaerales-related archaea are a prominent and recurrent signature of the plastisphere archaeome.

Note S4 Recovery and characterization of archaeal metagenome-assembled genomes

Using genome-resolved metagenomic binning, we reconstructed 23 non-redundant archaeal metagenome-assembled genomes (MAGs) that met at least medium-quality draft criteria (completeness ≥50%, contamination ≤10%; Supplementary Fig. S8)[8]. Taxonomic classification within the GTDB framework indicated two dominant archaeal lineages: Nitrososphaerales (n = 11; phylum Thermoproteota, class Nitrososphaeria) and Poseidoniales (n = 12; phylum Thermoplasmatota, class Poseidoniia). Notably, all five plastisphere-derived MAGs were affiliated with Nitrososphaerales, whereas all Poseidoniales MAGs were recovered exclusively from bulk-water metagenomes (Supplementary Fig. S8). The plastisphere signal was robust to dereplication strategy, as dereplication restricted to plastisphere-derived bins recovered the same five Nitrososphaerales MAGs. Despite comparable sequencing depth across habitats, fewer archaeal MAGs were recovered from plastisphere samples, consistent with the greater complexity and strain heterogeneity expected in surface-attached biofilms, which can reduce assembly contiguity and genome recovery[9]. This pattern is also consistent with the higher archaeal taxonomic diversity observed in the amplicon data (Fig. 1 in the main text).

Because Nitrososphaerales/Nitrososphaeria comprise ammonia-oxidizing archaea (AOA), typically characterized by genes encoding ammonia monooxygenase[10], we evaluated the nitrogen-cycling potential of recovered MAGs by annotating predicted proteins against NCycDB[11]. Among the five plastisphere-derived Nitrososphaerales MAGs, two encoded the complete ammonia monooxygenase gene set (*amoA*, *amoB*, *amoC*), supporting genomic potential for archaeal ammonia oxidation (Supplementary Fig. S8). One of these MAGs additionally contained a complete urease operon (*ureA*, *ureB*, *ureC*) (Supplementary Fig. S8), suggesting the potential to hydrolyze urea and thereby generate ammonia for nitrification[12]. Given that urea is widely available in marine systems and can support AOA growth and activity[13], this genomic configuration is consistent with the possibility that plastisphere-associated AOA exploit dissolved organic nitrogen substrates within the biofilm microenvironment.

We further detected *nirK* (copper-containing nitrite reductase) in most plastisphere Nitrososphaerales MAGs (Supplementary Fig. S8). In AOA, *nirK* has been shown to generate nitric oxide (NO) from nitrite and/or hydroxylamine-derived intermediates and is considered central to archaeal nitrogen metabolism[14]. NO-related reactions in AOA have also been linked to nitrous oxide (N_2O) formation under low-oxygen conditions[15]. Although gene presence alone does not demonstrate in situ activity, these marker genes suggest that plastisphere-associated AOA may contribute to localized nitrification and NO/N_2O-related transformations within redox-stratified plastisphere biofilms.

In contrast, bulk-water Poseidoniales MAGs lacked *amo* genes (Supplementary Fig. S8), consistent with genome-resolved studies describing Poseidoniales (Marine Group II archaea) as predominantly aerobic (photo)heterotrophs involved in dissolved and particulate organic matter turnover, with additional lineage-specific traits including transport and degradation of organic substrates and, in some taxa, nitrate-reduction capacity[16].

Finally, metagenomic read recruitment to MAG-predicted genes showed that Nitrososphaerales MAGs were significantly more abundant in plastisphere samples than in bulk water, whereas Poseidoniales MAGs showed the opposite pattern (Wilcoxon rank-sum tests, $P < 0.001$; Supplementary Fig. S9).

Collectively, the selective recovery and enrichment of AOA-affiliated MAGs in the plastisphere, together with their encoded potential for ammonia oxidation, urea utilization, and NO-related nitrite reduction, provide genome-resolved support for our gene-centric inference that the plastisphere may act as a localized hotspot for archaeal nitrogen transformations in coastal marine ecosystems.

Note S5 Plastisphere as an archaeal habitat distinct from other solid surfaces

To determine whether the distinctive archaeal assemblages observed on plastics represent a genuine substrate-specific signal rather than a general biofilm effect, we compared plastisphere archaeal communities with those on other solid surfaces, including wood, ceramic, metal, and cardboard, across five independent studies with sufficient replication, using both taxonomic composition and functional gene profiles as response variables.

For taxonomic composition (Supplementary Fig. S10a), four of the five studies showed greater between-group than within-group Bray–Curtis dissimilarity, indicating that plastisphere archaeal communities were generally more dissimilar from other-surface communities than they were internally variable. Two studies, wos_2 and wos_134, reached individual significance, whereas the others showed positive but non-significant trends, likely owing to limited within-study sample sizes. When combined across all five studies, Stouffer's weighted Z-test yielded $Z = 2.385$, $P < 0.01$, confirming consistent compositional separation of plastisphere archaeal communities from those on other solid surfaces across independent datasets.

A parallel pattern was observed for functional gene profiles (Supplementary Fig. S10b). Again, four of the five studies showed greater between-group than within-group Bray–Curtis dissimilarity, indicating that plastisphere samples harbored archaeal functional profiles distinct from those on other solid substrates. The combined Stouffer Z-test yielded $Z = 2.351$, $P < 0.01$, indicating that the functional distinctiveness of plastisphere archaeal communities was as consistent and statistically robust as their compositional distinctiveness.

Together, these results demonstrate that plastisphere archaeal communities differ from those on other solid surfaces at both taxonomic and functional levels, providing evidence that plastic substrates constitute a genuinely distinct archaeal habitat rather than simply another attachment surface. The convergence of compositional and functional signals across independent studies with diverse reference surfaces further supports the robustness of this conclusion.

Note S6 Archaeome dissimilarity patterns across polymer types and substrate types

To evaluate whether polymer type structures plastisphere archaeal communities, we compared three categories of Bray–Curtis dissimilarity across four independent studies that included multiple polymer types and non-plastic reference substrates with known polymer identity (wos_50, wos_85, wos_2, and wos_134): within-polymer, between-polymer, and plastic-versus-non-plastic dissimilarity. Analyses were conducted for both archaeal taxonomic composition and functional gene profiles (Supplementary Fig. S11).

Overall, results were inconsistent across studies. In some datasets, plastic-versus-non-plastic dissimilarity exceeded between-polymer dissimilarity, suggesting that substrate identity explained more variation than polymer chemistry. In others, this pattern was not observed, and similar inconsistency was found for functional gene profiles. We attribute this lack of consistency primarily to the limited size and scope of the available datasets, including low replication per polymer type, narrow polymer diversity, sparse non-plastic reference groups, and the restricted ecological representativeness of incubation-based experiments.

These results indicate that currently available public datasets are insufficient to robustly disentangle polymer-type effects from broader substrate-identity effects in plastisphere archaeal communities. In contrast, the present study was designed to resolve the broader plastisphere signal under field conditions using a much larger set of paired plastisphere and ambient seawater samples across diverse coastal environments. We therefore interpret the consistent taxonomic and functional separation observed in our dataset as evidence of a generalizable plastisphere-specific archaeal signal, while recognizing that polymer-specific effects remain an important question for future work.

Supplementary Figures

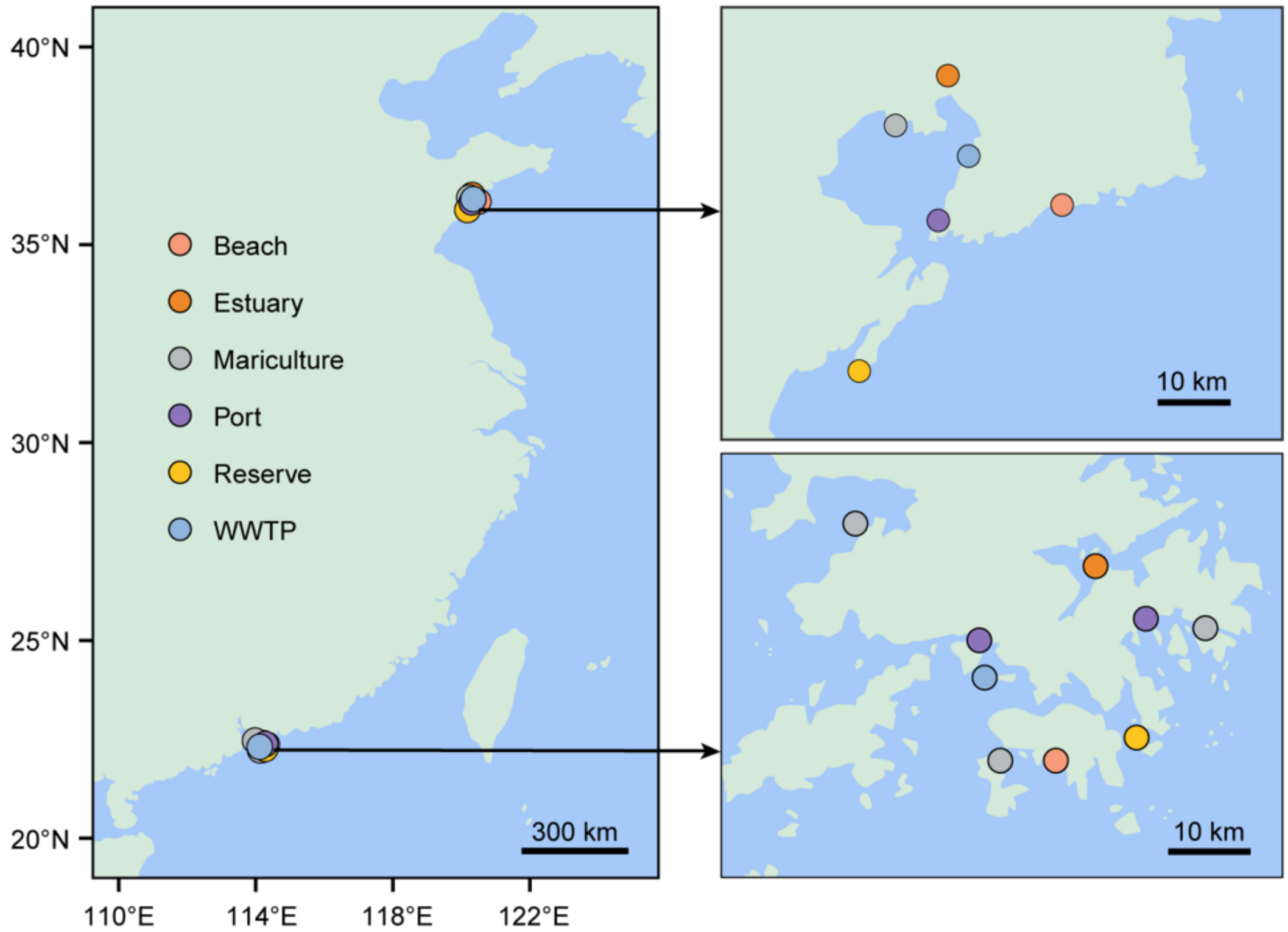

Fig. S1 Geographical locations of sampling sites. A total of 94 sample pairs (plastic debris and bulk water) were collected from beach, estuary, mariculture, port, reserve and wastewater treatment plants (WWTP) in Qingdao and Hong Kong coasts, encompassing a wide range of environmental conditions and human impacts. The map was created by the authors in R using ggplot2 and sf.

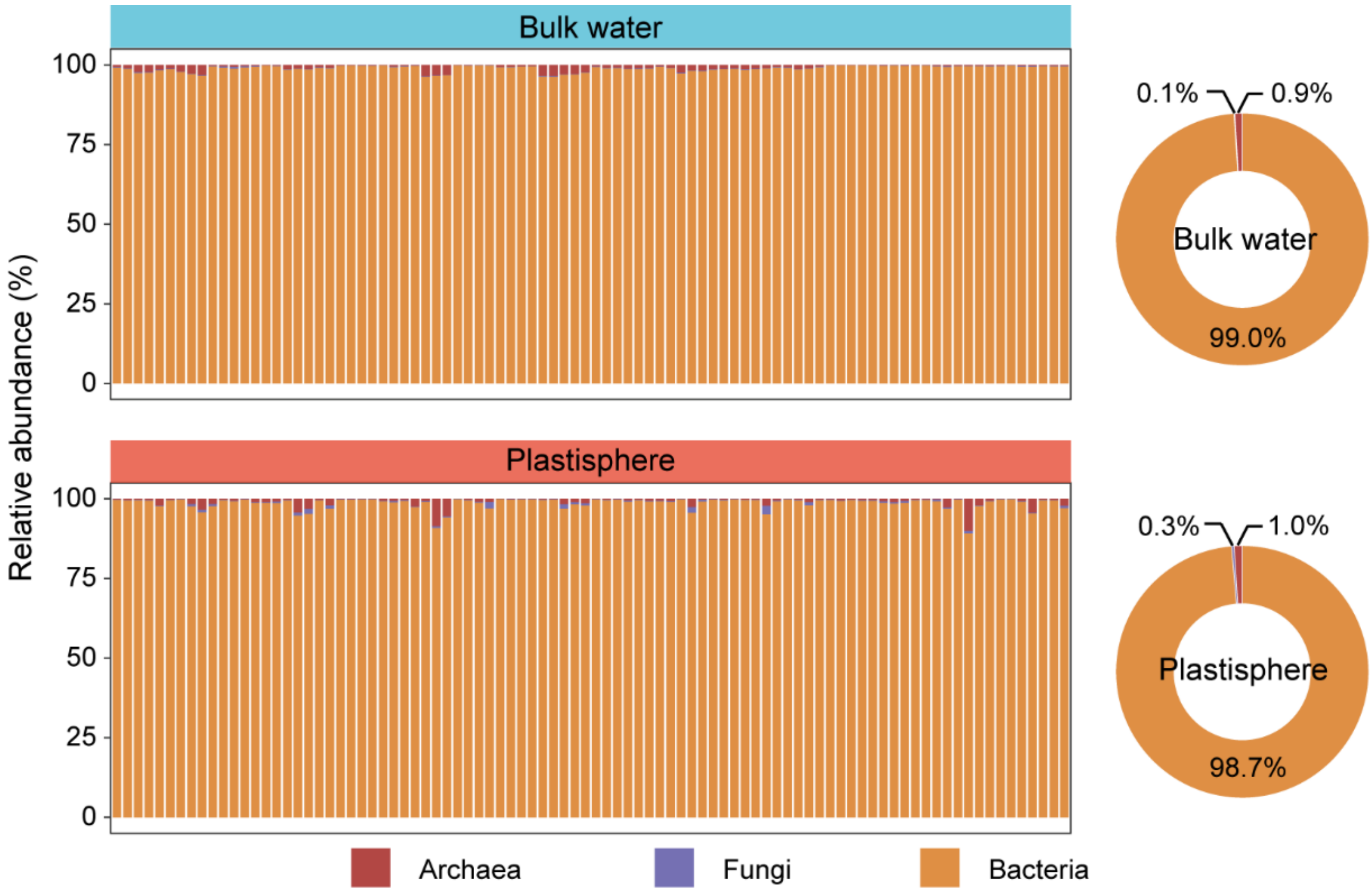

Fig. S2 Domain-level relative abundances of archaea, bacteria, and fungi in plastisphere and bulk-water communities. Stacked bar plots and pie charts show the relative proportions of archaea, bacteria, and fungi in plastisphere and seawater samples. Details of the classification strategy and normalization procedure are provided in Supplementary Note S1. n = 82 per group.

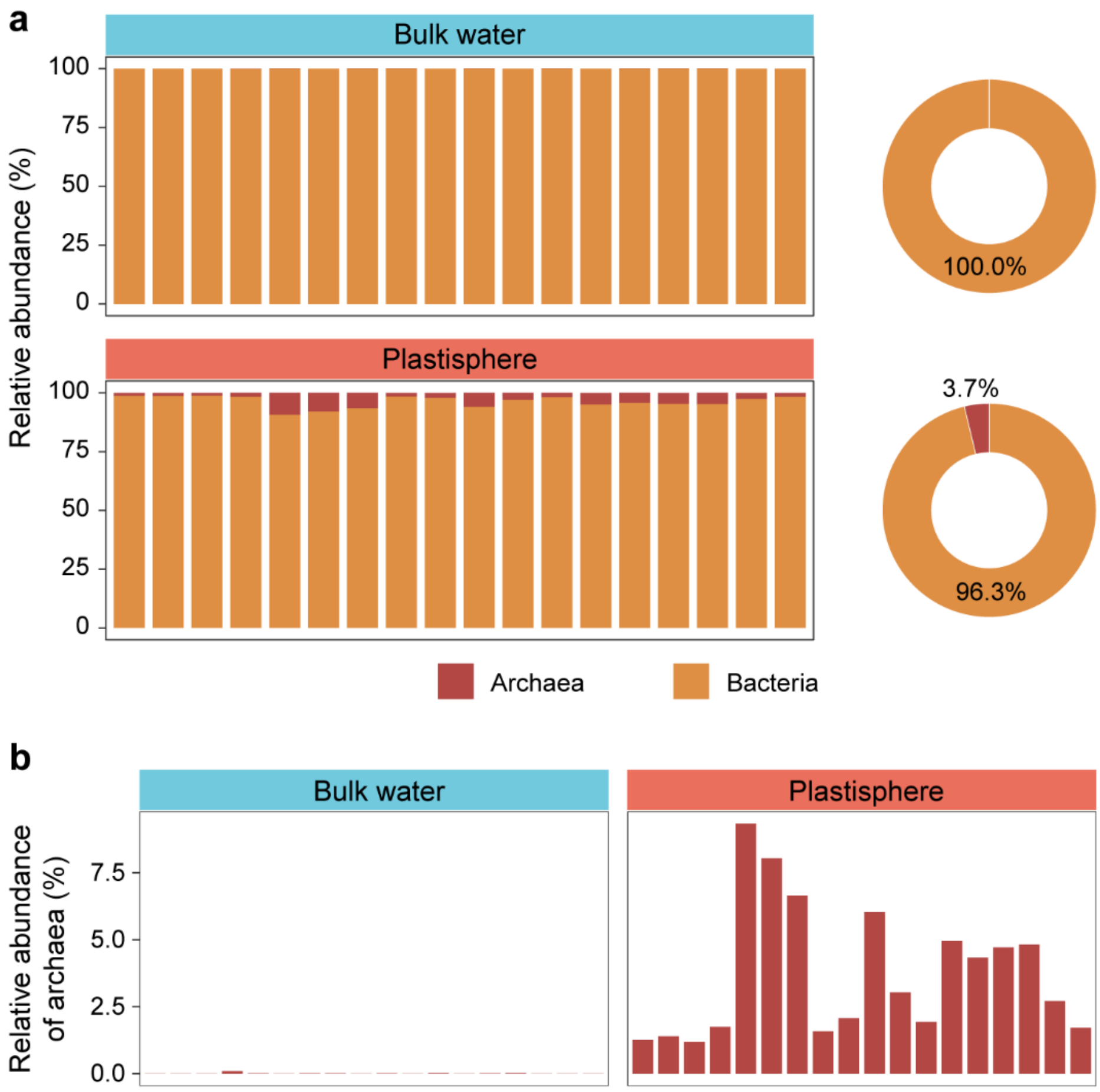

Fig. S3 Recovery of archaeal reads from a bacterial-targeted 16S amplicon dataset. a, Stacked bar plots and pie charts showing the relative proportions of archaea and bacteria recovered from a previously published 16S amplicon dataset generated with the 515F/806R primer pair[7] when archaeal reads were retained during taxonomic assignment. **b,** Relative abundance of archaeal reads in plastisphere and seawater samples from the same dataset. These data are provided as a methodological illustration showing that archaeal sequences can be recovered from bacterial-targeted amplicon surveys at detectable levels, particularly in plastisphere communities, and do not represent new results of the present study. *n* = 18 per group.

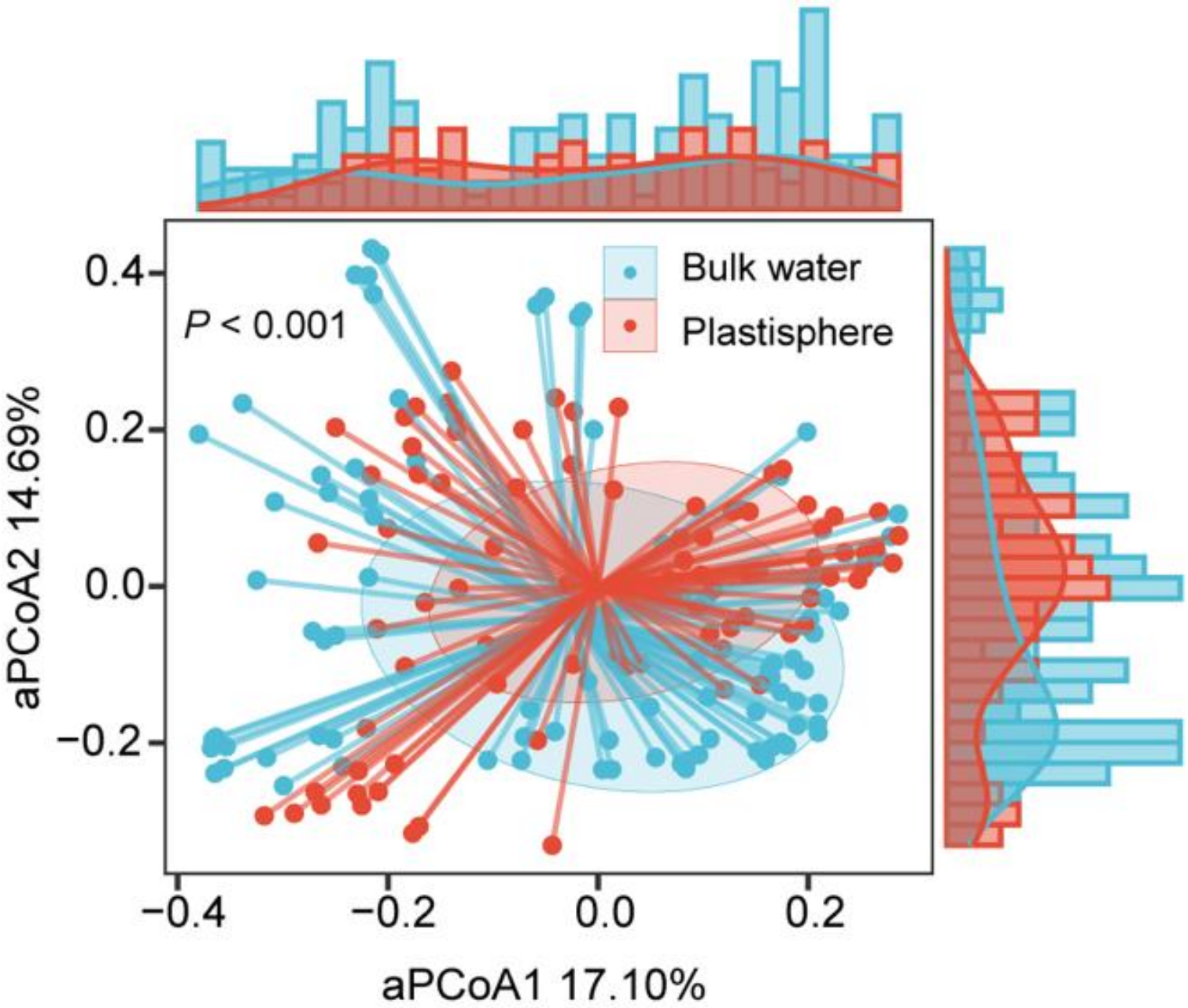

Fig. S4 Differences in archaeal community structure between the plastisphere and bulk water. Adjusted principal coordinates analysis (aPCoA) and permutational multivariate analysis of variance (PERMANOVA) show that archaeal community composition differs significantly between the plastisphere and bulk water. n = 88 per group.

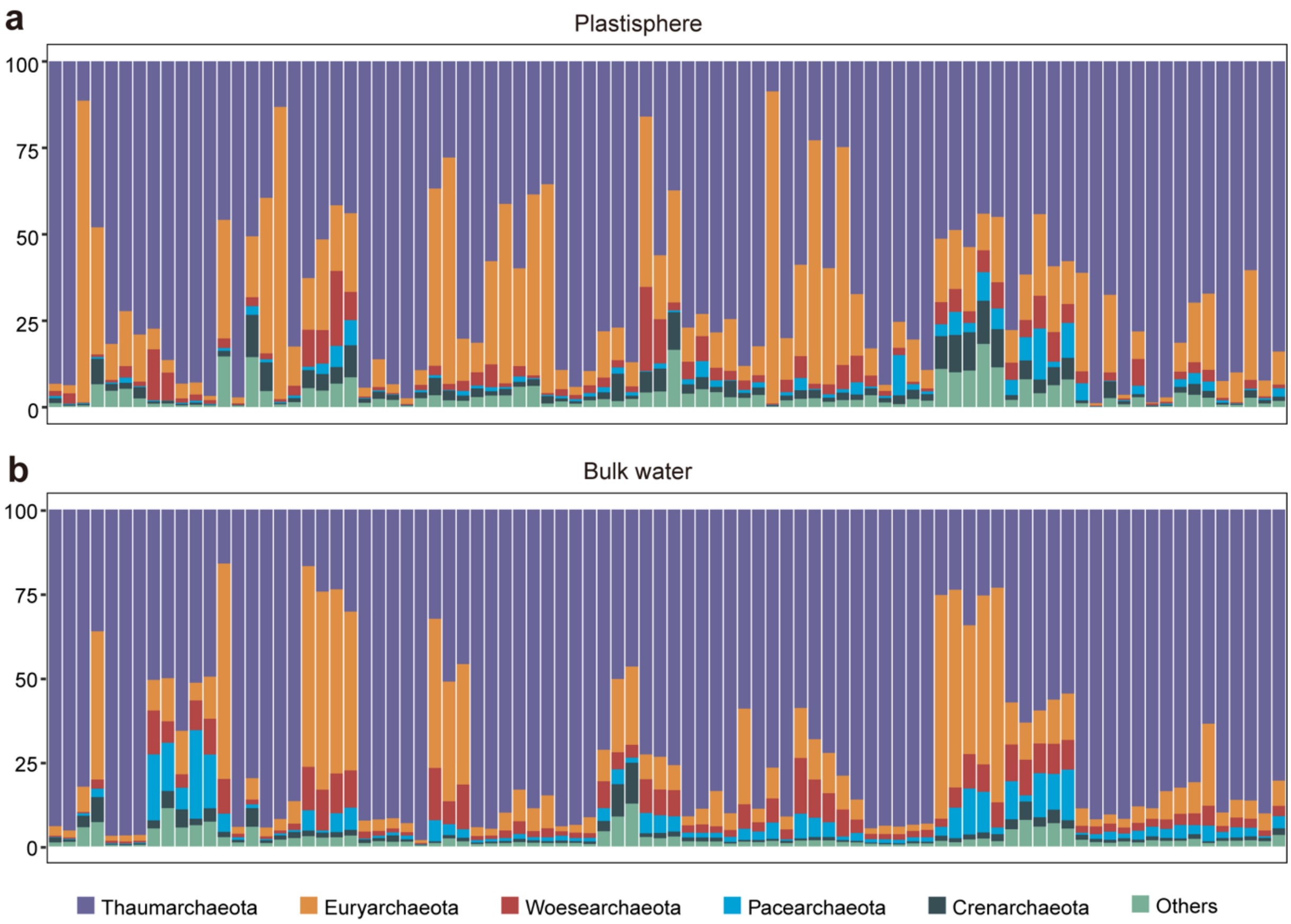

Fig. S5 Phylum-level composition of archaeal communities in the plastisphere and bulk water. Stacked bars show the relative abundances of major archaeal phyla in individual plastisphere (**a**) and bulk-water (**b**) samples. n = 88 per group.

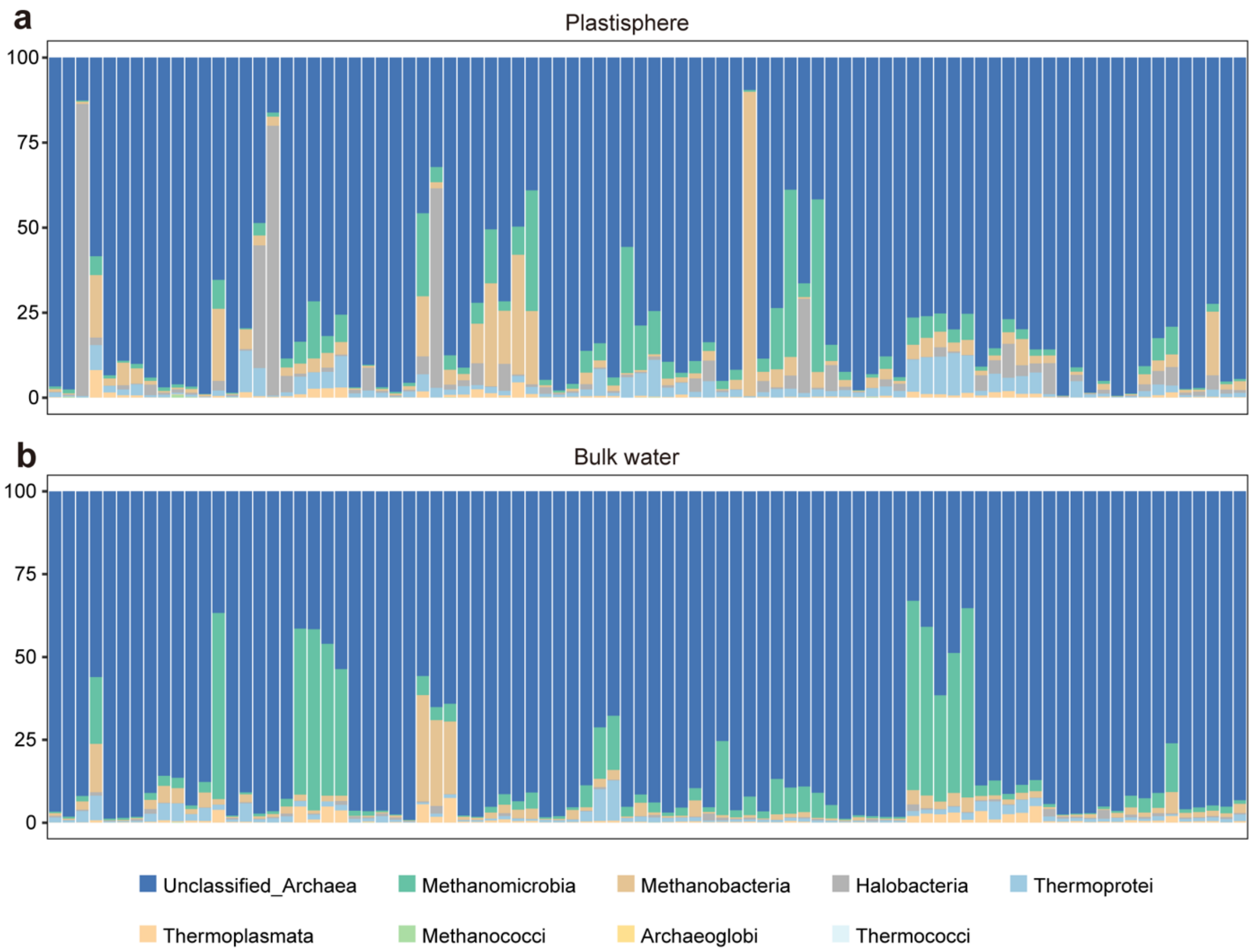

Fig. S6 Class-level composition of archaeal communities in the plastisphere and bulk water. Stacked bars show the relative abundances of major archaeal classes in individual plastisphere (**a**) and bulk-water (**b**) samples. n = 88 per group.

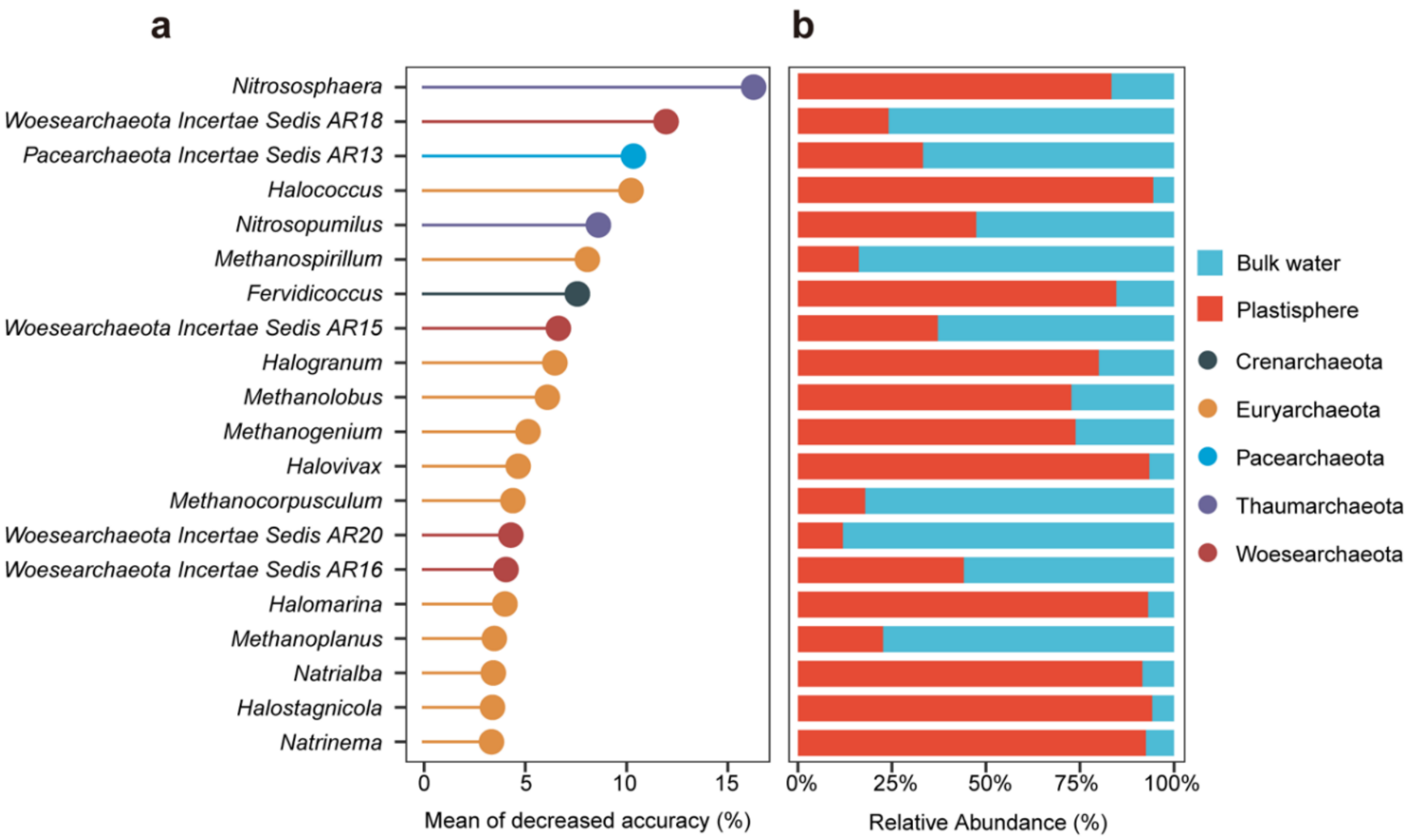

Fig. S7 Plastisphere biomarkers based on a random-forest model. a, The top 20 archaeal genera contributing most to the accuracy of the random-forest classification model for distinguishing the plastisphere from bulk water were identified as plastisphere biomarkers in the coastal ecosystem. The biomarker taxa are listed in descending order of importance to the accuracy of the model. **b,** Relative proportions of mean abundance of the biomarker taxa in the plastisphere and bulk water. n = 88 per group.

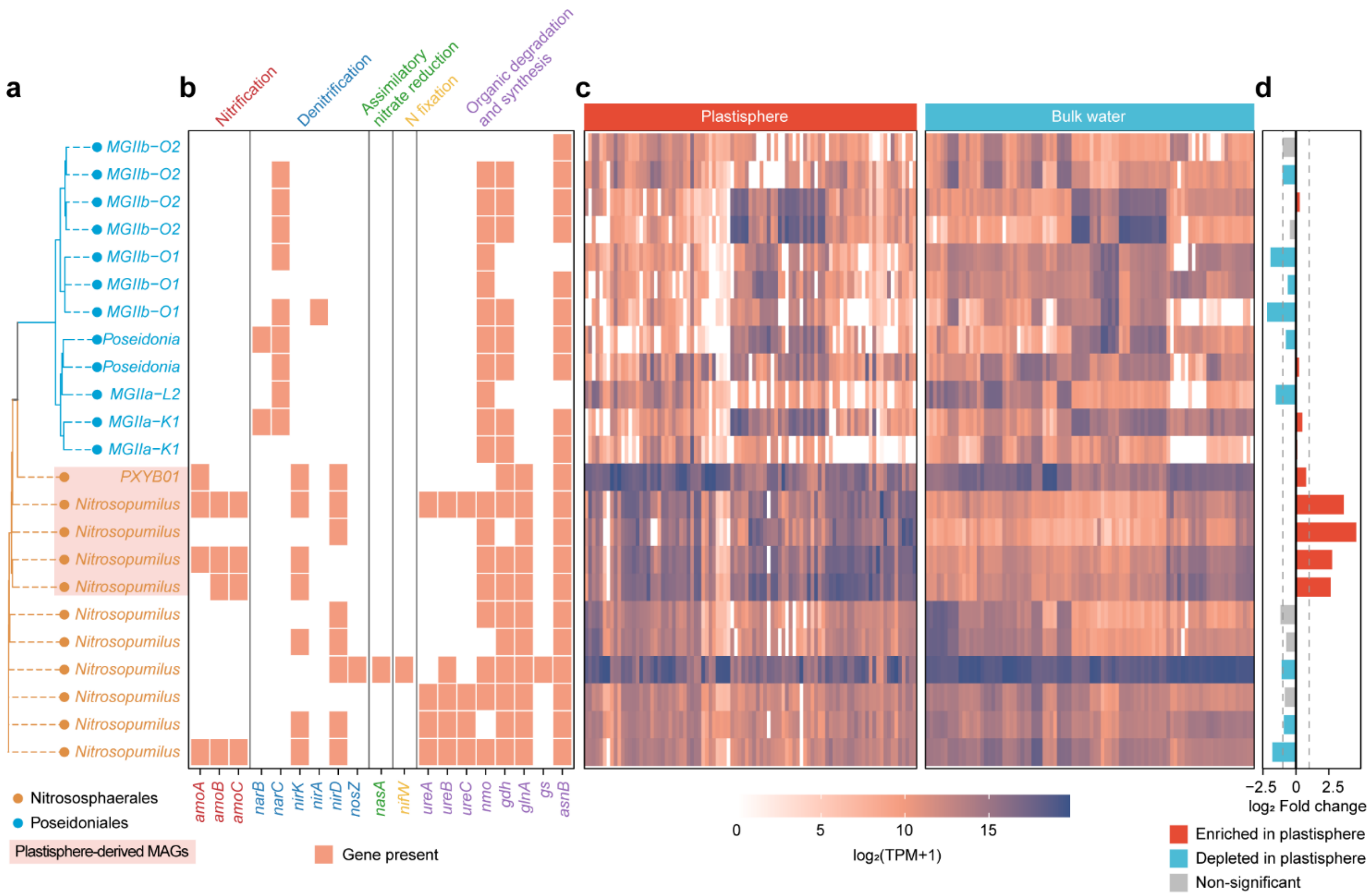

Fig. S8 Genome-resolved characterization of archaeal MAGs recovered from plastisphere and bulk-water communities. a, Maximum-likelihood phylogenetic tree of 23 archaeal MAGs inferred from ar53 marker genes. Tips are coloured by taxonomic order (orange, Nitrososphaerales; blue, Poseidoniales); plastisphere-derived MAGs are highlighted with a pink background. **b,** Presence of nitrogen-cycling genes across all 23 MAGs, grouped by functional process according to NCycDB classification. **c,** Heat map of MAG relative abundance (log_2-transformed TPM) across plastisphere and bulk water samples. **d,** Differential abundance of each MAG between plastisphere and bulk water. Red and blue bars indicate significant enrichment in plastisphere and bulk water, respectively; grey bars indicate no significant difference (Wilcoxon rank-sum test, *P* < 0.05).

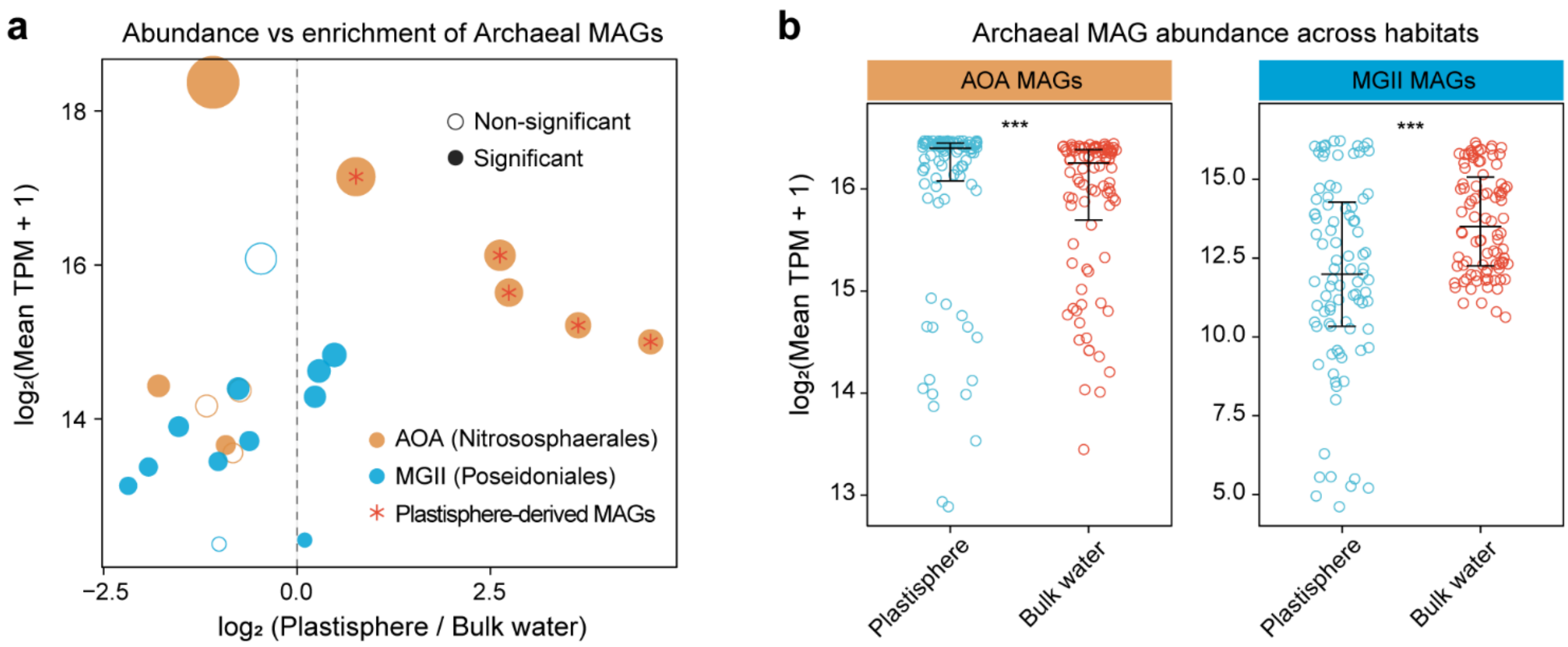

Fig. S9 Differential abundance of archaeal MAGs between plastisphere and bulk water. a, Bubble plot showing the relationship between differential abundance and mean abundance for each MAG. Bubble size represents mean total TPM, and colour indicates taxonomic affiliation (orange, Nitrososphaerales; blue, Poseidoniales). Plastisphere-derived MAGs are marked with asterisks. Filled and open symbols indicate statistically significant and non-significant differences, respectively (Wilcoxon rank-sum test, $P < 0.05$). **b,** Mean abundance of Nitrososphaerales and Poseidoniales MAGs in plastisphere and bulk-water samples. Significance was assessed using Wilcoxon rank-sum tests ($n = 82$ per group; *** $P < 0.001$).

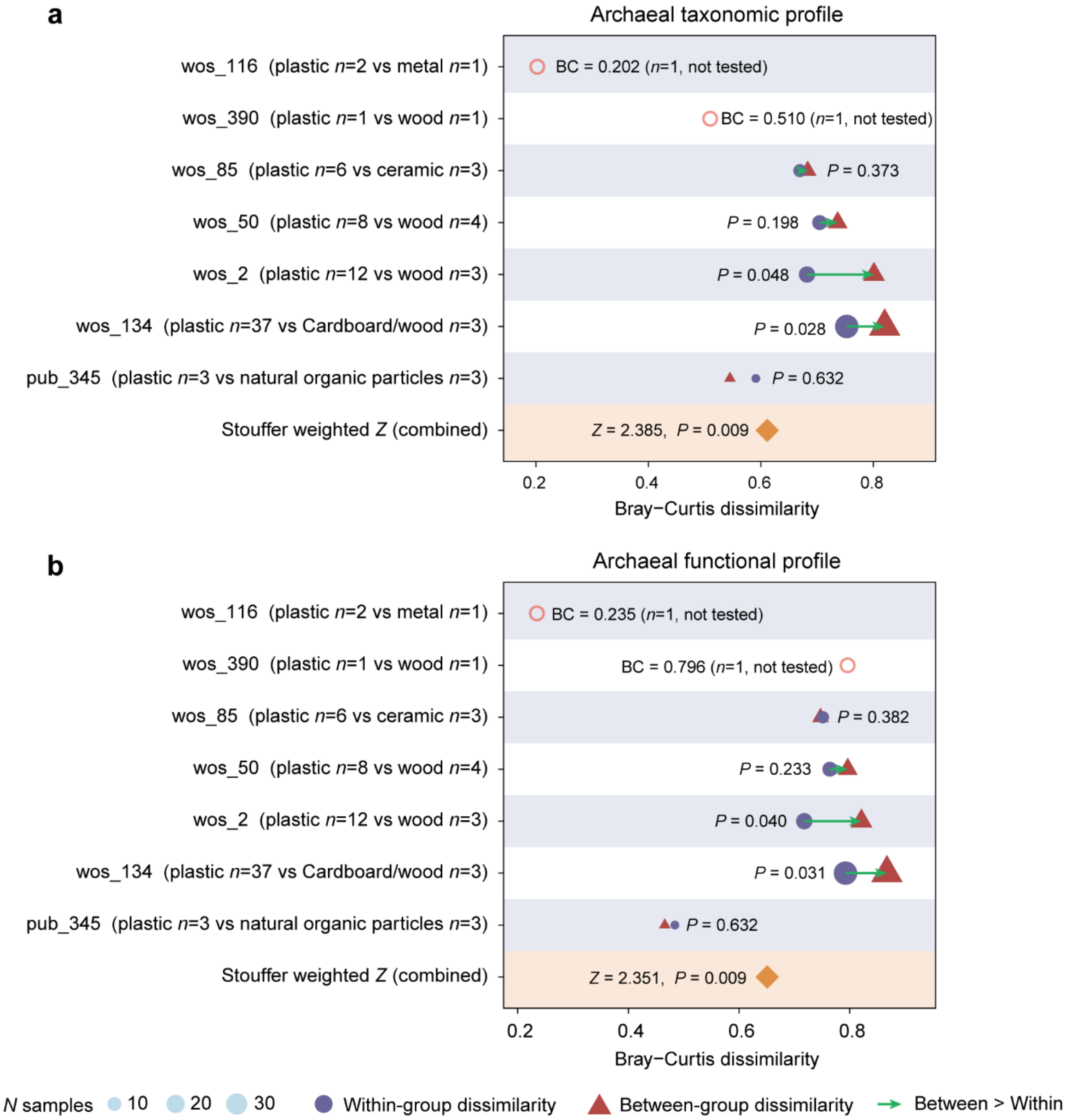

Fig. S10 Plastisphere archaeal communities are compositionally and functionally distinct from those on other solid surfaces. a, Taxonomic composition. **b,** Functional gene profiles. In both panels, red triangles indicate mean between-group Bray–Curtis dissimilarity (plastisphere versus other solid surfaces), and blue circles indicate mean within-group Bray–Curtis dissimilarity. Green arrows indicate studies in which between-group dissimilarity exceeded within-group dissimilarity. Point size is proportional to total sample size. For wos_116 and wos_390, within-group dissimilarity could not be calculated because of insufficient replication, and only between-group dissimilarity is shown descriptively (open circles). Statistical significance within each study was assessed using permutation tests, with the number of permutations limited to the total number of possible label arrangements when this was <999. Combined evidence across the five testable studies was evaluated using Stouffer's weighted *Z*-method, weighting each study by the square root of total sample size.

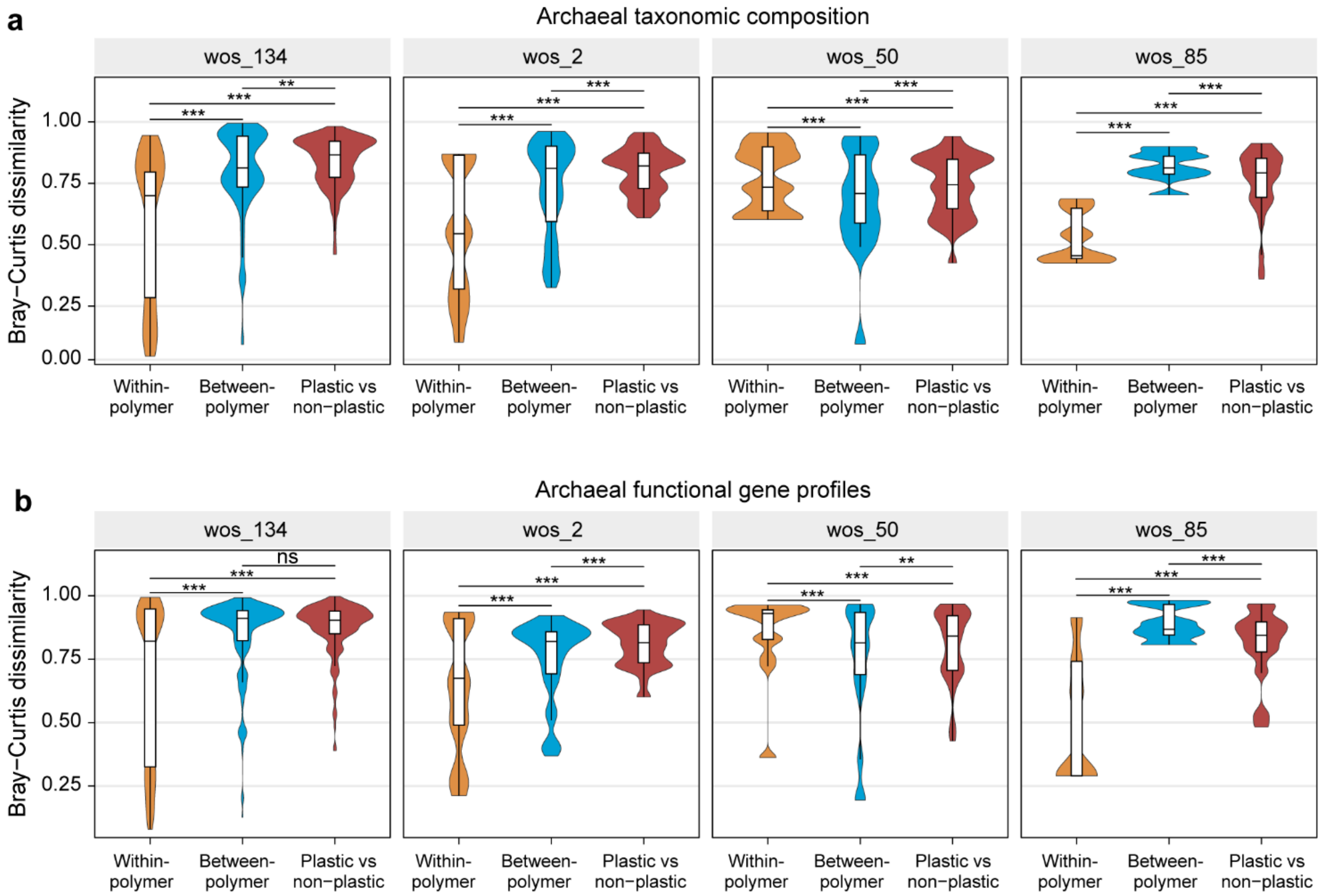

Fig. S11 Comparison of archaeal community dissimilarity across polymer types and substrate types. a, Taxonomic composition based on species-level GTDB annotation. **b,** Functional gene profiles based on combined archaeal carbon, nitrogen, phosphorus, sulfur, and methane cycling genes. In each panel, three categories of pairwise Bray–Curtis dissimilarity are shown across four independent studies: within-polymer, between-polymer, and plastic-versus-non-plastic. Each distribution comprises 999 bootstrap-sampled pairwise distances to ensure equal sample sizes across categories despite group imbalance. The horizontal line within each box indicates the median. Statistical significance was assessed using Wilcoxon rank-sum tests (*** $P < 0.001$, ** $P < 0.01$, ns = non-significant).

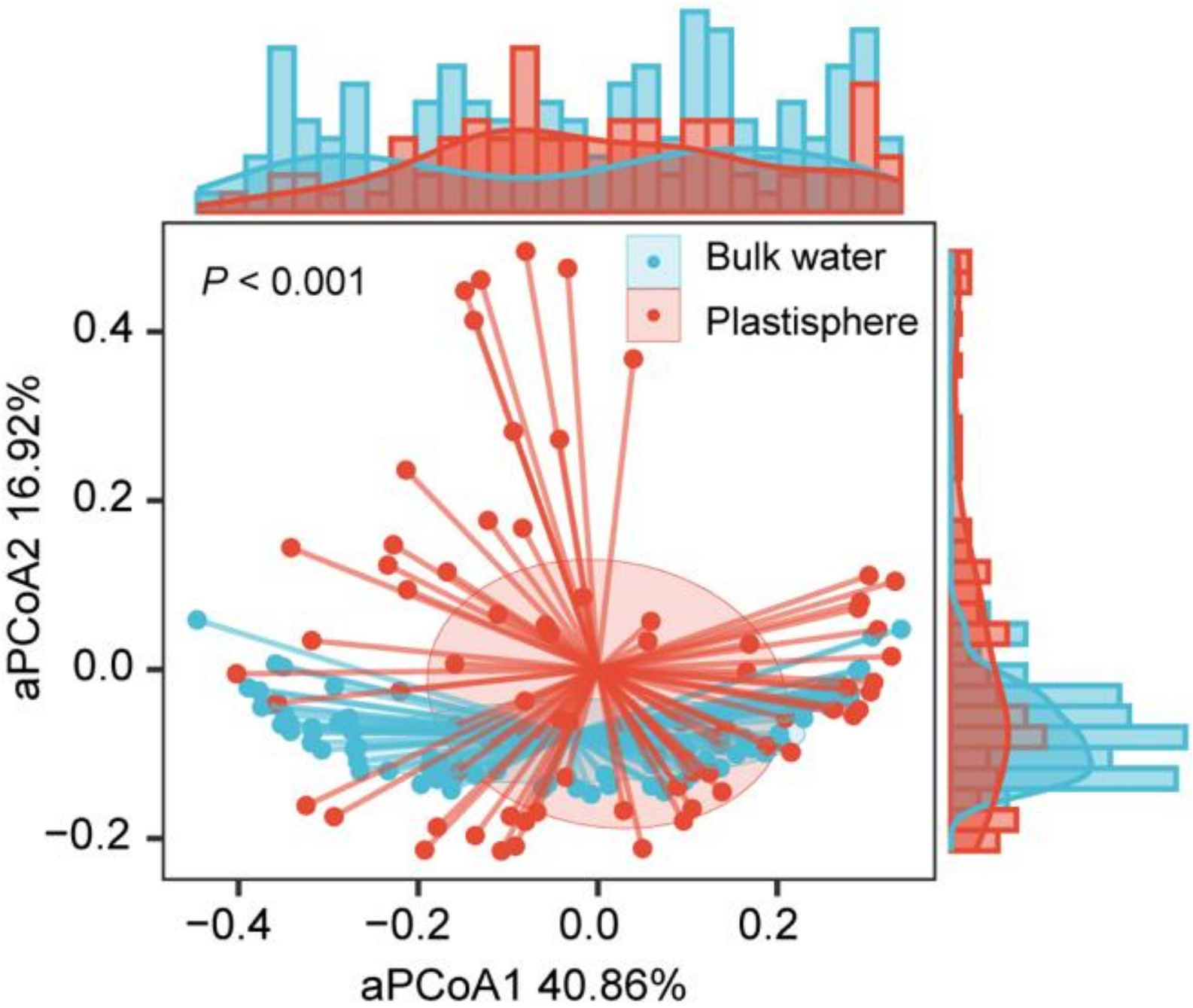

Fig. S12 Differences in archaeal functional profiles between the plastisphere and bulk water. Adjusted principal coordinates analysis (aPCoA) and permutational multivariate analysis of variance (PERMANOVA) show that archaeal functional profiles differ significantly between the plastisphere and bulk water. n = 82 per group.

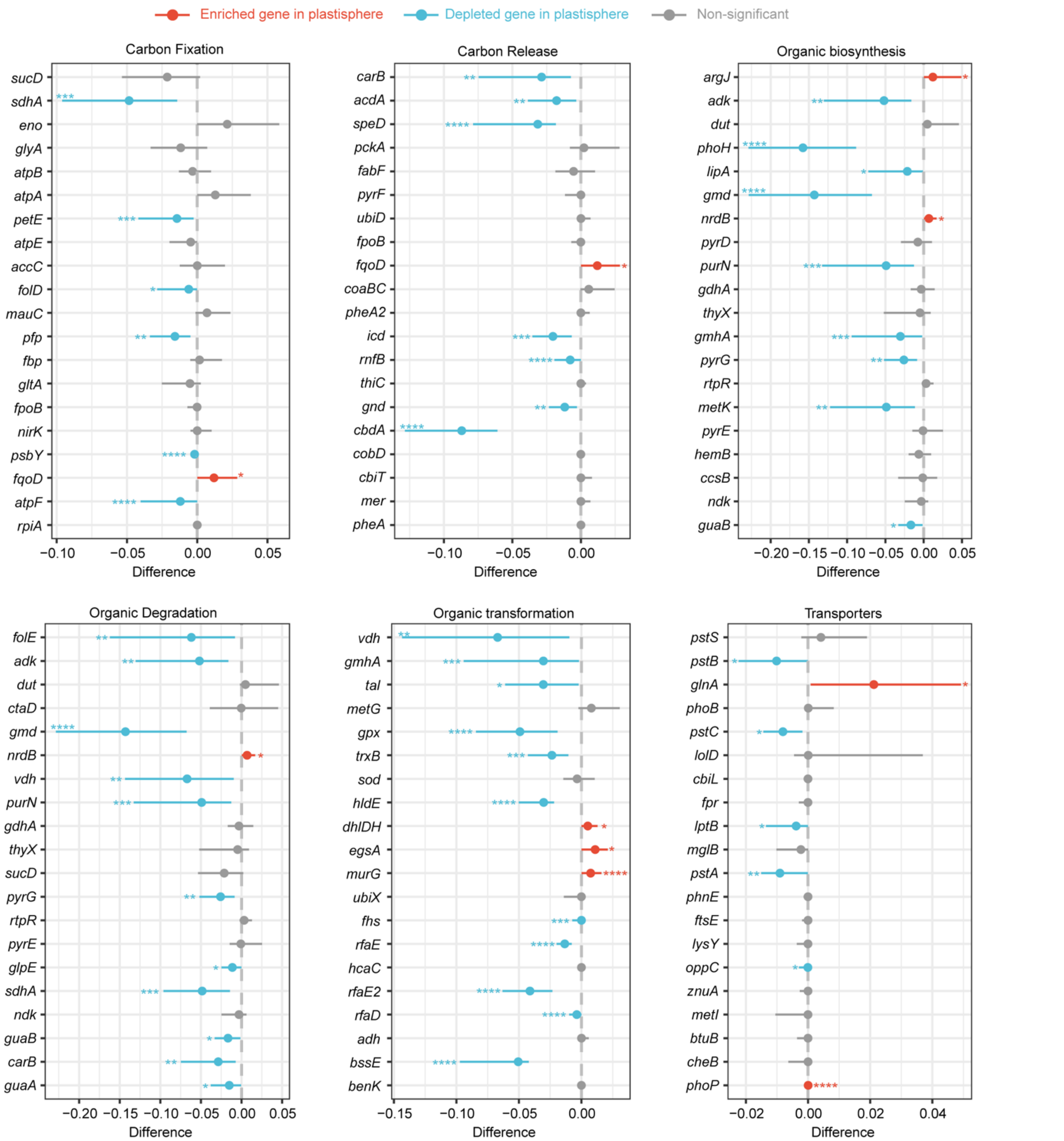

Fig. S13 Shifts in archaeal functional genes associated with carbon cycling in the plastisphere. Metagenomic-based assessment of functional genes associated with carbon cycling, including the pathways of carbon fixation, carbon release, carbon biosynthesis, carbon degradation, organic transformation, and transporters. Only the top 20 most abundant genes in each pathway are shown. Data are shown as Hodges–Lehmann estimates of effect size with 95% confidence intervals. n = 82 per group. *P < 0.05, **P < 0.01, ***P < 0.001, ****P < 0.0001.

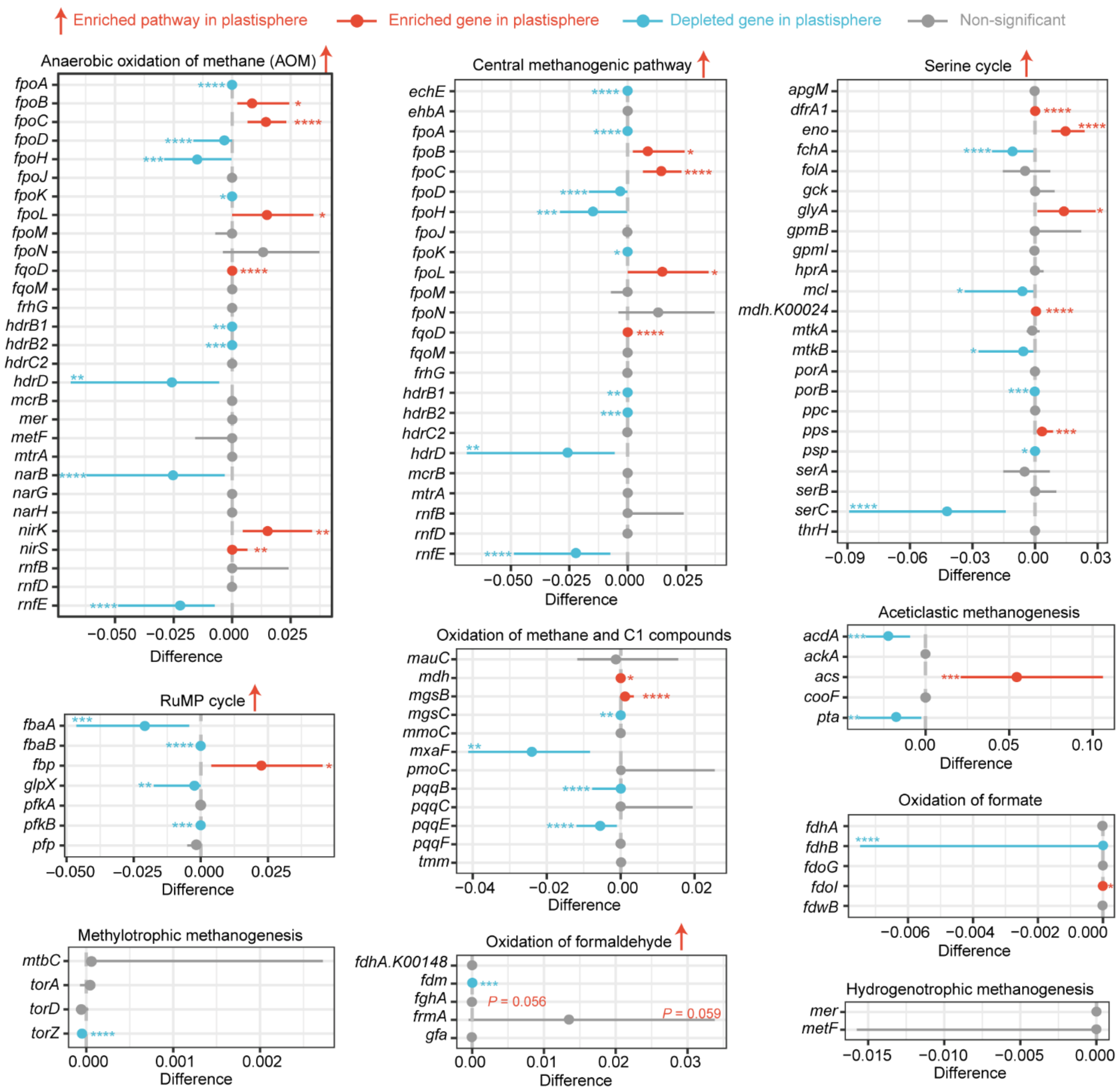

Fig. S14 Shifts in archaeal functional genes associated with methane cycling in the plastisphere. Metagenomic-based assessment of functional genes associated with methane cycling, including the pathways of aceticlastic methanogenesis, anaerobic oxidation of methane, central methanogenic pathway, hydrogenotrophic methanogenesis, methylotrophic methanogenesis, oxidation of formaldehyde, oxidation of formate, oxidation of methane and C1 compounds, ribulose monophosphate (RuMP) cycle, and serine cycle. Data are shown as Hodges–Lehmann estimates of effect size with 95% confidence intervals. n = 82 per group. *$P < 0.05$, **$P < 0.01$, ***$P < 0.001$, ****$P < 0.0001$.

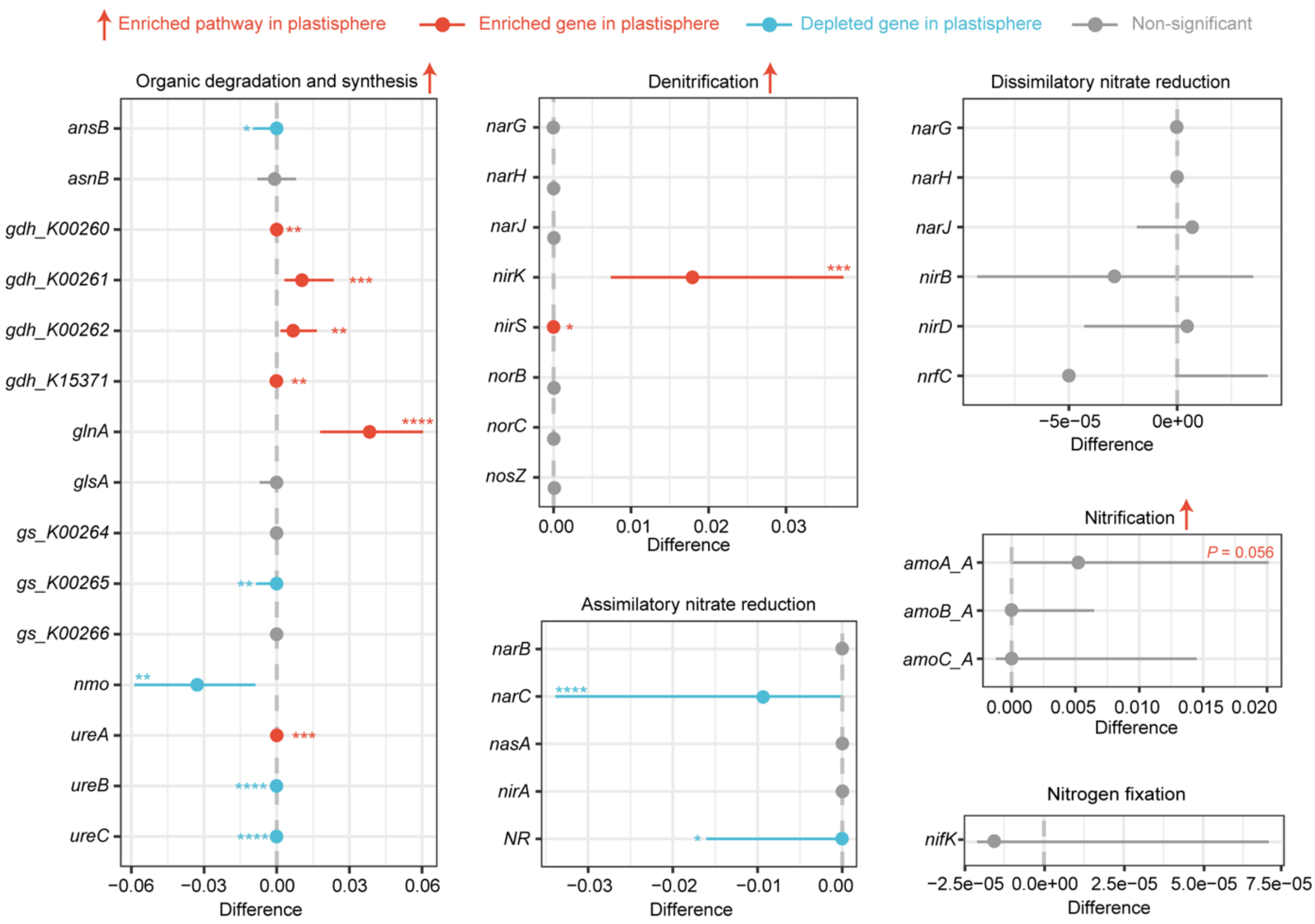

Fig. S15 Shifts in archaeal functional genes associated with nitrogen cycling in the plastisphere. Metagenomic-based assessment of functional genes associated with nitrogen cycling, including the pathways of assimilatory nitrate reduction, denitrification, dissimilatory nitrate reduction, nitrification, nitrogen fixation, and organic degradation and synthesis. Data are shown as Hodges–Lehmann estimates of effect size with 95% confidence intervals. n = 82 per group. *P < 0.05, **P < 0.01, ***P < 0.001, ****P < 0.0001.

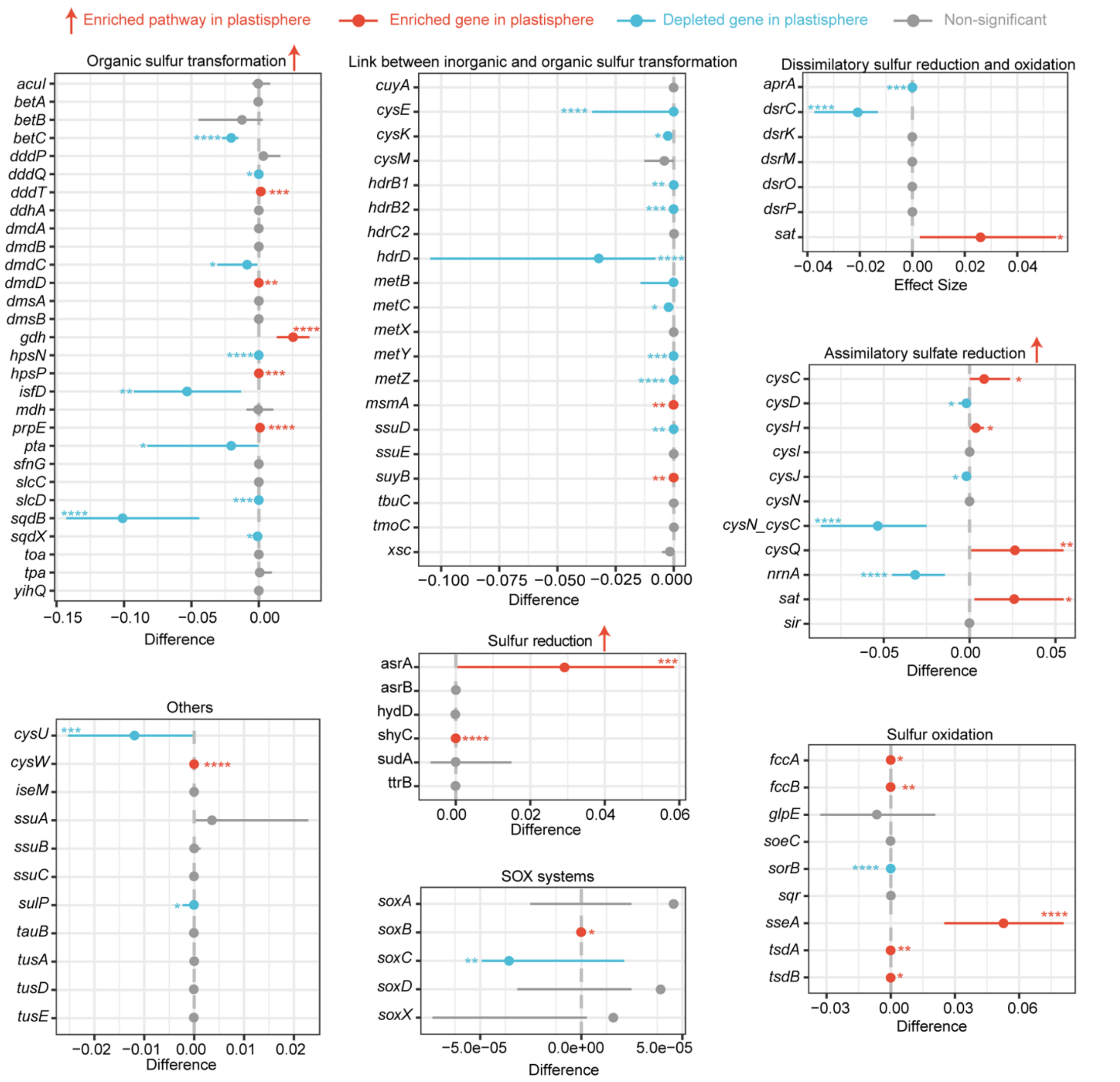

Fig. S16 Shifts in archaeal functional genes associated with sulfur cycling in the plastisphere. Metagenomic-based assessment of functional genes associated with sulfur cycling, including the pathways of assimilatory sulfate reduction, dissimilatory sulfur reduction and oxidation, link between inorganic and organic sulfur transformation, organic sulfur transformation, sulfur oxidation (SOX) systems, sulfur oxidation, sulfur reduction, and others. Data are shown as Hodges–Lehmann estimates of effect size with 95% confidence intervals. n = 82 per group. *P < 0.05, **P < 0.01, ***P < 0.001, ****P < 0.0001.

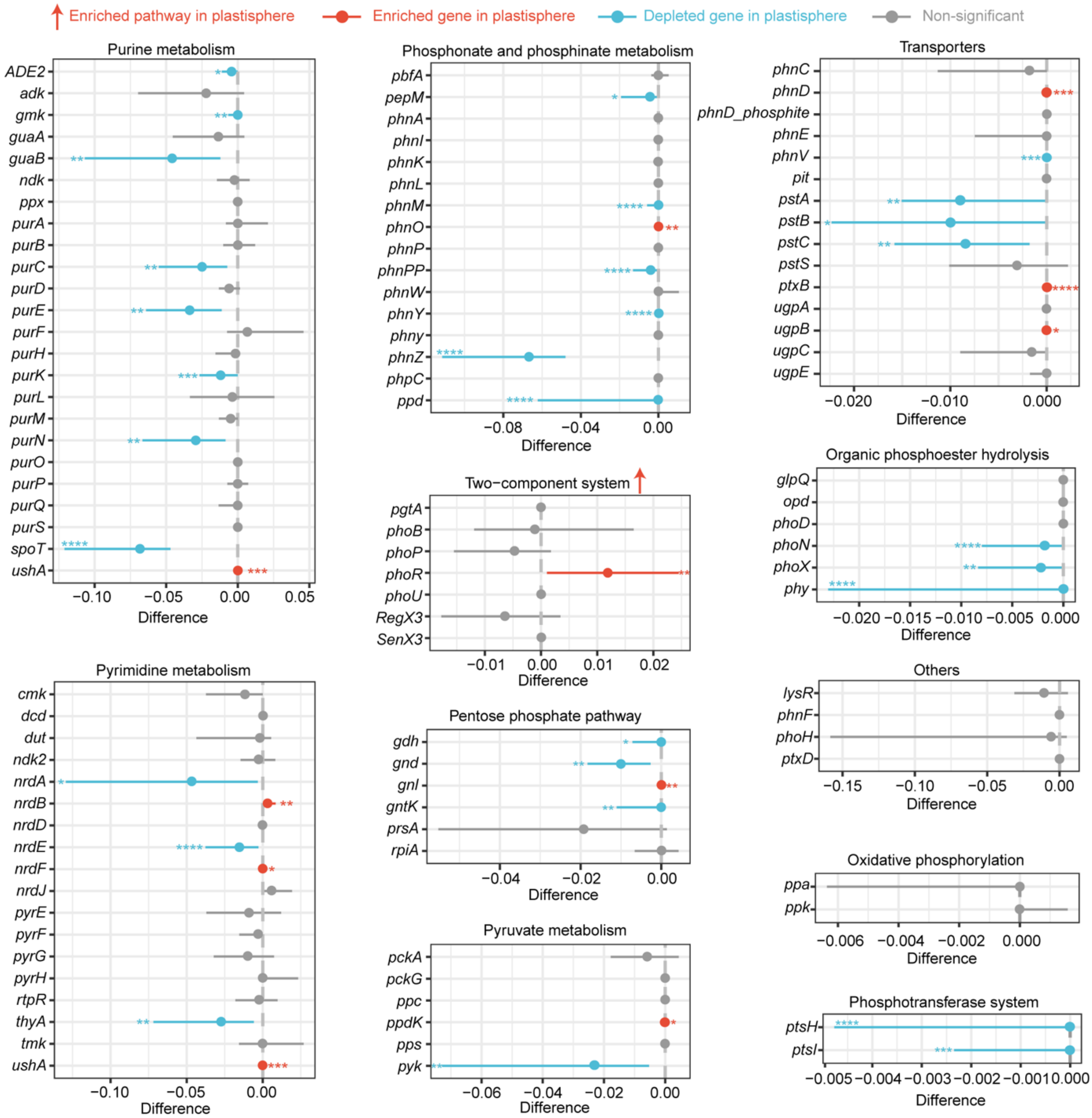

Fig. S17 Shifts in archaeal functional genes associated with phosphorus cycling in the plastisphere. Metagenomic-based assessment of functional genes associated with phosphorus cycling, including the pathways of organic phosphoester hydrolysis, oxidative phosphorylation, pentose phosphate pathway, phosphonate and phosphinate metabolism, phosphotransferase system, purine metabolism, pyrimidine metabolism, pyruvate metabolism, transporters, two-component system, and others. Data are shown as Hodges–Lehmann estimates of effect size with 95% confidence intervals. n = 82 per group. *P < 0.05, **P < 0.01, ***P < 0.001, ****P < 0.0001.

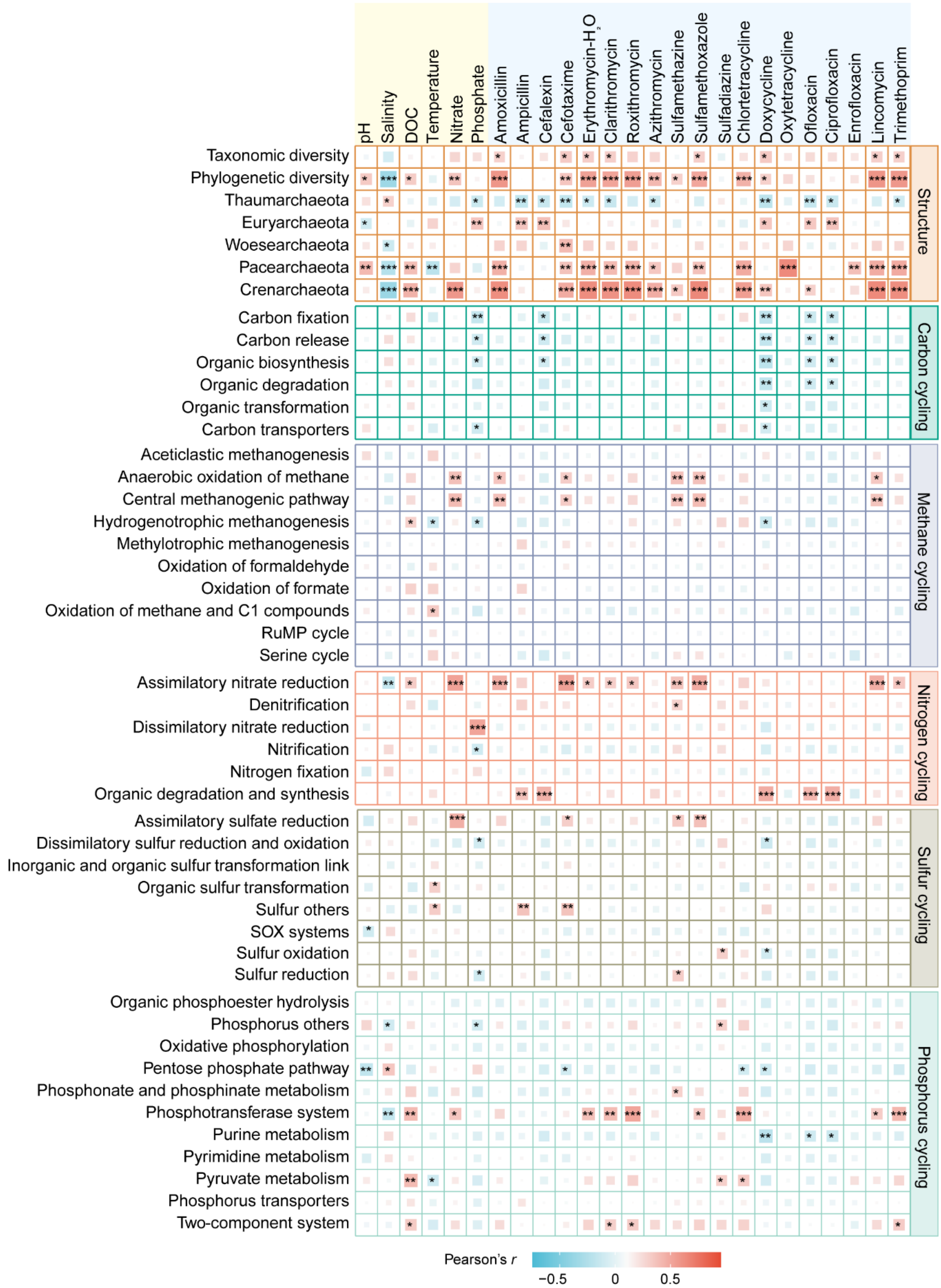

Fig. S18 Correlations between environmental variables and archaeal community metrics in the plastisphere. The color filled in cells corresponds to Pearson's *r* value. RuMP = ribulose monophosphate, SOX = sulfur oxidation, DOC = dissolved organic carbon. $n = 74$. $^*P < 0.05$, $^{**}P < 0.01$, $^{***}P < 0.001$.

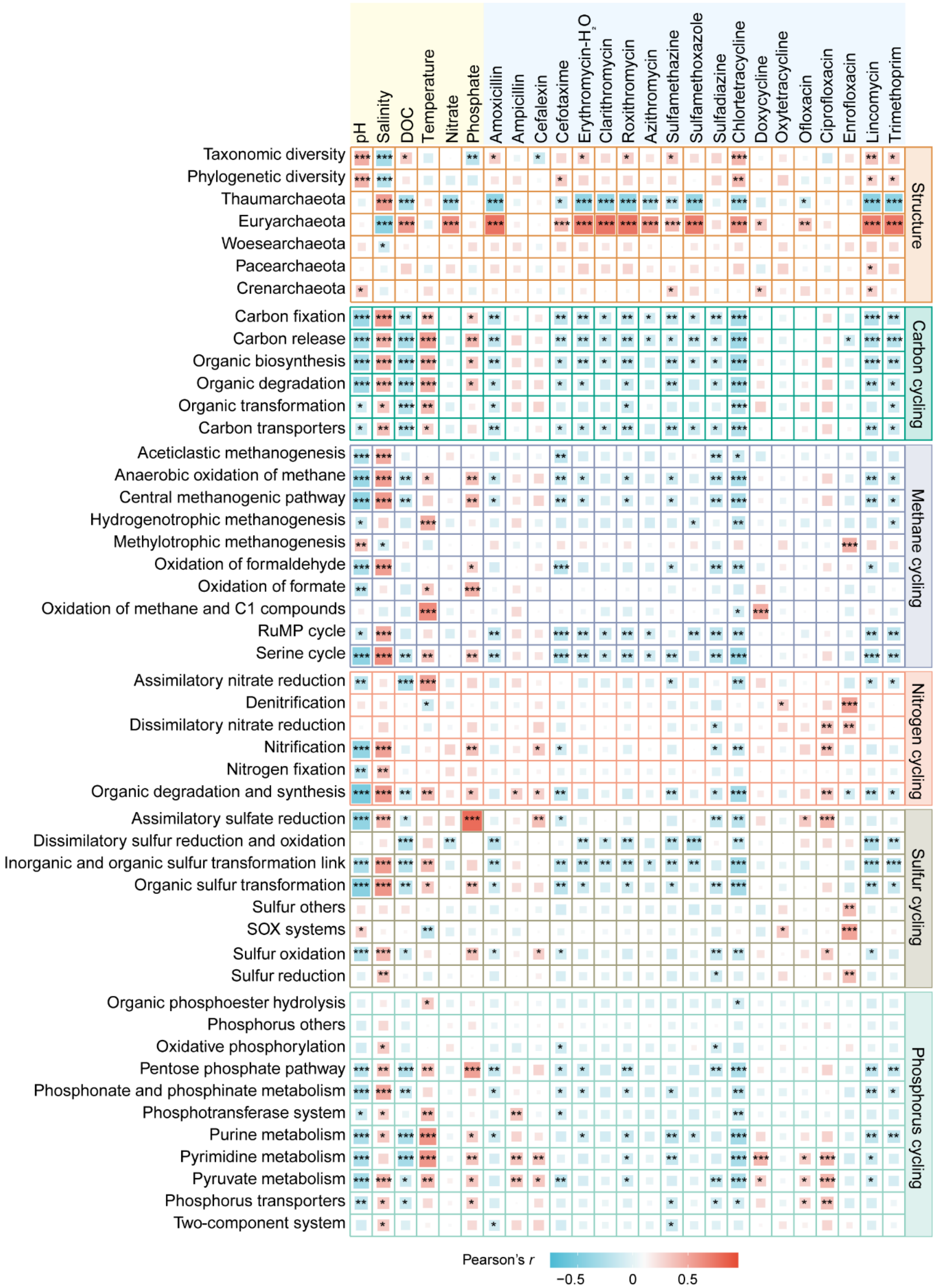

Fig. S19 Correlations between environmental variables and archaeal community metrics in bulk water. The color filled in cells corresponds to Pearson's *r* value. RuMP = ribulose monophosphate, SOX = sulfur oxidation, DOC = dissolved organic carbon. n = 74. *P < 0.05, **P < 0.01, ***P < 0.001.

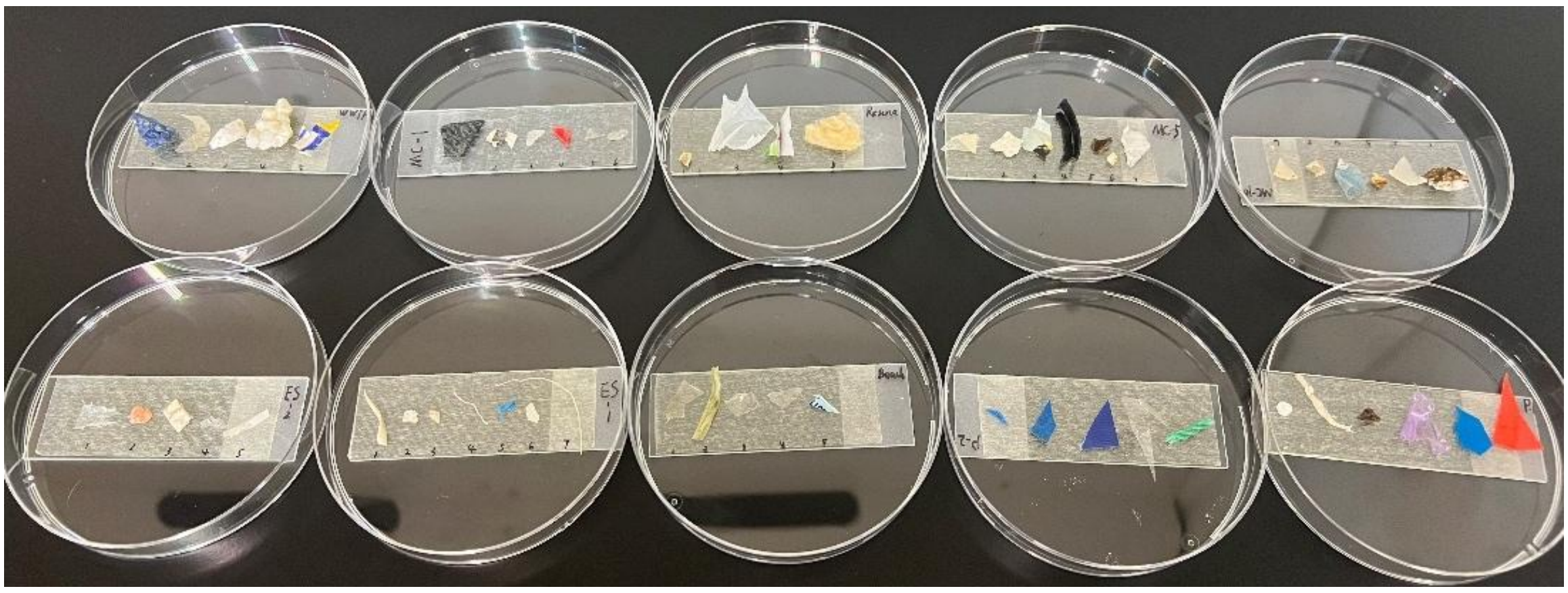

Fig. S20 Representative plastic debris collected during field sampling. The photograph shows examples of the colors, shapes, and sizes of the collected plastic particles.

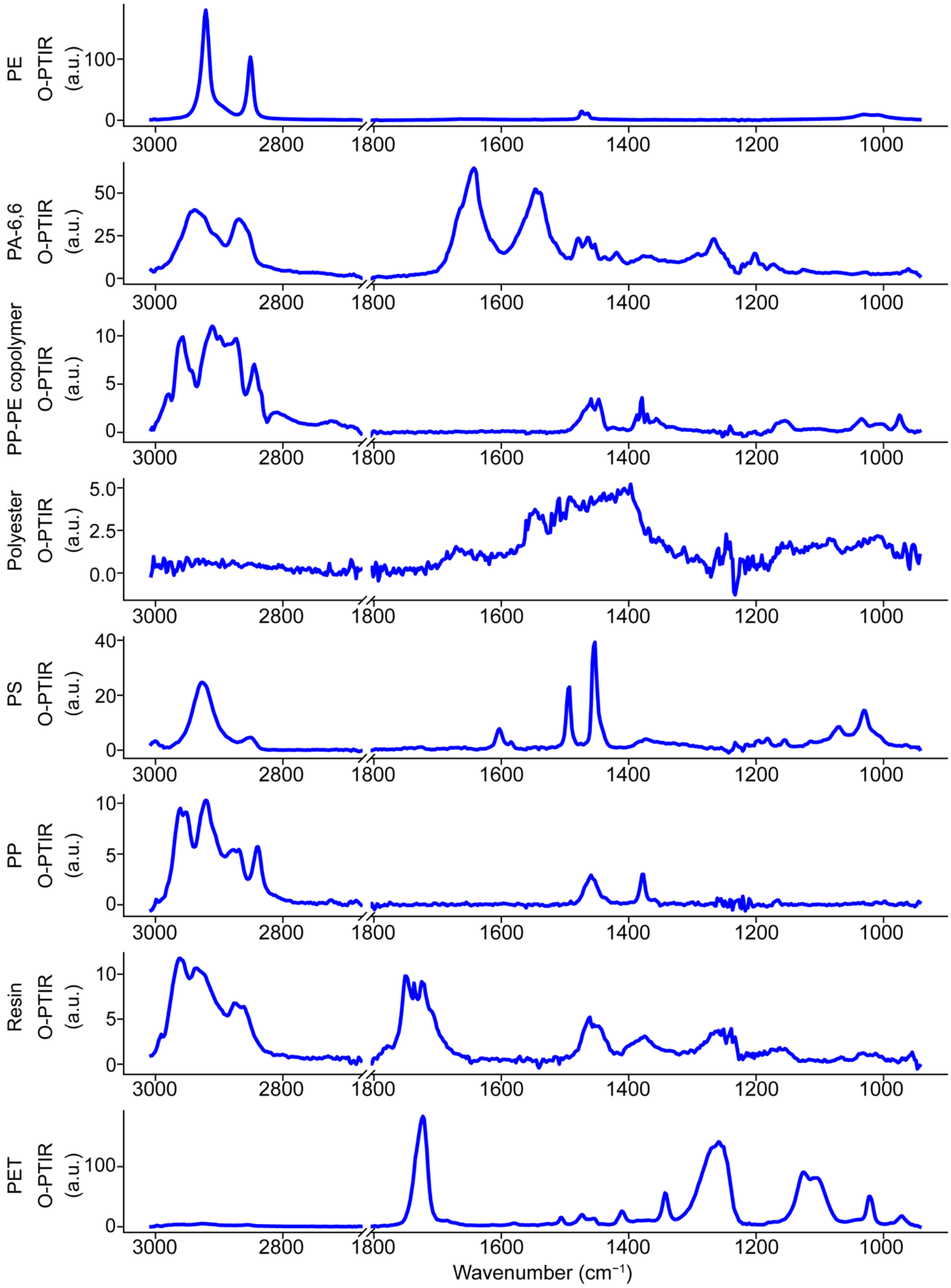

Fig. S21 Optical photothermal infrared (O-PTIR) spectroscopy of typical polymers. PE = polyethylene, PA-6,6 = polyamide 6,6, PP-PE copolymer = polypropylene–co-polyethylene copolymer, PS = polystyrene, PP = polypropylene, PET = polyethylene terephthalate.

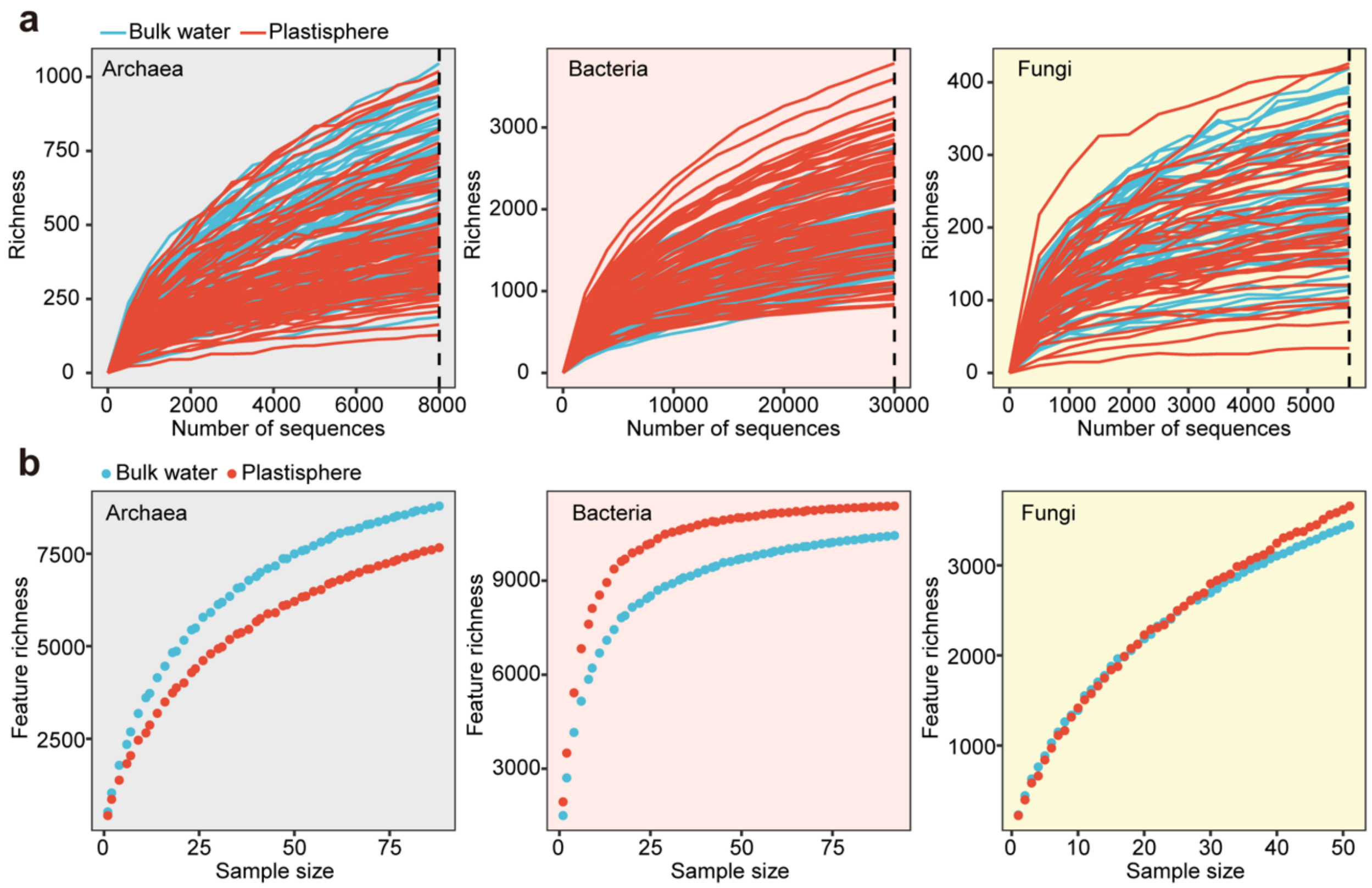

Fig. S22 Rarefaction curves. a, The number of archaeal, bacterial and fungal ASVs with the increasing number of sequences (the sequencing depth). **b,** Accumulation curves of the number of archaeal, bacterial and fungal ASVs with an increasing number of samples (the sample size). These curves flatten out at the final sequence depth (8,000 for archaea, 30,000 for bacteria, and 5,700 for fungi) and sample size (*n* = 88 per archaeal group, *n* = 92 per bacterial group and *n* = 51 per fungal group) chosen for this study, indicating that these sequencing depths and sample sizes captured most microbial members in the plastisphere and bulk water and were sufficient for the comparative analyses.

Supplementary Tables

Table S1 Polymer identification of plastic debris samples by O-PTIR spectroscopy.

Coastal ecosystems	Particle ID	Polymer	Hit quality index (HQI)
Beach	A1	Polypropylenes	86.63
Beach	A2	Ethylene propylene copolymer	81.24
Beach	A3	Resin	81.22
Beach	A4	Polypropylenes	82.93
Beach	A5	Polypropylenes	91.8
Estuary	A1	Polypropylene-co-ethylene	92.07
Estuary	A2	Polyethylenes	86.42
Estuary	A3	Polypropylene isotactic	90.85
Estuary	A4	Polyethylene, chlorinated	73.38
Estuary	A5	Low-molecular polyethylene	91.42
Estuary	A6	Ethylene copolymer	87.78
Estuary	A7	Polyethylenes	90.04
Estuary	B1	Polypropylenes	85.46
Estuary	B2	Polyethylenes	90.72
Estuary	B3	Polyethylenes	81.21
Estuary	B4	Polyethylenes	90.47
Estuary	B5	Polyethylene Terephthalate	90.05
Mariculture	A1	Ethylene-propylene copolymer	93.53
Mariculture	A2	Propylene-ethylene copolymer	83.88
Mariculture	A3	Polyethylene-co-Propylene copolymer	89.68
Mariculture	A4	Polyethylene Terephthalate	82.12
Mariculture	A5	Polyethylenes	81.71
Mariculture	A6	Polypropylenes	84.54
Mariculture	A7	Polypropylene	79.74
Mariculture	B1	Polystyrene	74.07
Mariculture	B2	Polyethylenes	90.9
Mariculture	B3	Polyester urethane	74.77
Mariculture	B4	Polypropylenes	90.83
Mariculture	B5	Polypropylenes	85.62
Mariculture	B6	Polyethylenes	82.21
Mariculture	C1	Ethylene-propylene copolymer	93.78
Mariculture	C2	Polypropylenes	82
Mariculture	C3	Polypropylenes	79.7
Mariculture	C4	Butadiene rubber	84.82
Mariculture	C5	Polyester	81.05
Mariculture	C6	Polystyrene	72.75
Mariculture	C7	Polyethylene-co-Propylene copolymer	92.72
Mariculture	D1	Polypropylenes	89.8
Mariculture	D2	Polypropylenes	80

Mariculture	D3	Polystyrenes	81.09
Mariculture	D4	Polypropylenes	93.12
Mariculture	D5	Polyethylene Terephthalate	70.28
Port	A1	Polystyrene	79.85
Port	A2	Polypropylenes	86.96
Port	A4	Polyethylenes	88.67
Port	A5	Polyethylenes	85.68
Port	A6	Ethylene-Propylene copolymer	82.67
Port	B1	Polyethylenes	92.28
Port	B2	Polypropylenes	88.33
Port	B3	Polyethylene Terephthalate	91.33
Port	B4	Polyethylenes	76.74
Port	B5	Polyethylene-co-Propylene copolymer	81.87
Reserve	A1	Propylene-ethylene copolymer	74.43
Reserve	A2	Low-molecular polyethylene	91.82
Reserve	A3	Polyethylenes	82.9
Reserve	A4	Polyamide-6,6	91.6
Reserve	A5	Polyethylene terephthalate	76.65
WWTP	A1	Propylene-ethylene copolymer	85.7
WWTP	A2	Polypropylenes	72.89
WWTP	A3	Polypropylene-co-ethylene	94.18
WWTP	A4	Polystyrene	72.73
WWTP	A5	Polypropylenes	78.85

Table S2 Antibiotic concentrations detected at the sampling sites. Concentrations are shown in ng L^{-1}. AMX = Amoxicillin, AMP = Ampicillin, CFX = Cefalexin, CTX = Cefotaxime, ERY = Erythromycin-H_2O, CLR = Clarithromycin, ROX = Roxithromycin, AZI = Azithromycin, SMZ = Sulfamethazine, SMX = Sulfamethoxazole, SDZ = Sulfadiazine, CTC = Chlortetracycline, DOX = Doxycycline, OTC = Oxytetracycline, OFL = Ofloxacin, CIP = Ciprofloxacin, ENR = Enrofloxacin, LIN = Lincomycin, TMP = Trimethoprim.

Sample ID	AMX	AMP	CFX	CTX	ERY	CLR	ROX	AZI	SMZ	SMX	SDZ	CTC	DOX	OTC	OFL	CIP	ENR	LIN	TMP
HKSumMc1	0	0.278	0.913	0.260	1.329	0.030	0	0	0	0.159	0.346	2.072	0.556	0	0.146	0.682	0	0	0
HKSumPort1	0	0	0	0.393	4.639	0.567	0.059	0.728	0	2.096	0.985	2.858	0.890	0	2.321	0.624	0	0.529	0.799
HKSumWWTP2	0	0	4.360	0.205	10.180	10.300	0.116	7.275	0	4.959	3.269	2.628	1.480	0	146.109	29.662	0	0.098	4.589
HKWinBeach2	0	0	0.871	0	1.296	0.331	0.015	0.855	0	3.463	0.458	2.653	0.743	0	0.996	0.739	0.123	0	0.555
HKWinBeach3	0	0	0	0.213	1.618	0.285	0.015	0	0	3.472	0.432	1.965	0.612	0	1.127	1.618	0	0	0.827
HKWinBeach4	0	0	0.526	0.510	3.271	0.358	0	0.762	0	3.807	0.952	2.114	0.770	0	0.866	0	0	0	0.669
HKWinEs1	0.265	0.274	1.086	0	1.258	0.551	0.061	0.368	0	1.574	1.881	1.353	0.429	0	0.614	1.532	0	0	0.380
HKWinEs2	0	0	0.888	0	3.073	0.405	0.045	0.375	0	1.491	3.771	1.167	0.470	0	0.676	1.173	0	0	0.358
HKWinEs3	0.369	0.419	1.197	0.124	1.500	0.351	0.023	0.444	0	1.760	0.865	1.204	0.454	0	0.547	2.018	0	0	0.168
HKWinEs4	0.631	0	1.545	0.070	1.810	0.395	0.024	0.433	0	1.539	0.748	1.323	0.258	0.190	0.508	1.111	0	0	0.453
HKWinEs5	0	0.270	1.272	0	1.479	0.422	0.031	0.751	0	1.665	0.984	1.746	0.584	0	0.433	1.281	0	0	0.287
HKWinMc10	0	0	0	0	0.264	0.064	0	0.138	0	4.754	0.532	1.438	0.413	0	0.157	0.526	0	0	0.409
HKWinMc11	0	0	1.785	0.402	1.267	0.648	0.021	1.203	0	3.196	1.026	2.147	0.692	0	1.284	1.018	0	0.491	0.756
HKWinMc12	0	0	3.742	0.170	2.631	0.819	0.025	1.549	0	3.798	0.915	1.968	0.568	0	1.619	0.648	0	0.120	0.663
HKWinMc13	0	0	2.378	0.245	1.406	0.639	0	1.165	0	3.210	0.738	1.459	0.439	0	2.087	1.037	0	0.030	0.717
HKWinMc15	0	0	1.423	0.427	1.367	0.716	0.055	1.404	0	3.188	0.630	1.676	0.730	0.169	2.869	1.818	0	0	0.830
HKWinMc6	0	0	0.556	0	2.273	0.067	0	0	0	3.798	0.530	1.328	0.559	0	0.217	0	0	0	0.376
HKWinMc7	0	0	0.732	0.144	1.112	0.036	0	0.142	0	4.674	0.432	1.466	0.727	0	0	0	0	0	0.148
HKWinMc8	0.273	0	0	0.071	0.399	0.049	0.014	0	0	5.363	0.564	1.605	0.452	0	0	0	0	0	0.332
HKWinMc9	0	0	0	0.338	0.245	0.061	0	0	0	5.103	0.582	1.687	0.408	0	0	0.572	0	0	0.260
HKWinMR1	0	0	0	0.105	0.596	0.039	0.023	0	0	4.964	0.619	1.145	0.296	0	0	1.494	0	0.144	0.428
HKWinPort1	0	0	4.447	0.088	1.356	1.022	0.063	0.751	0	2.374	0.943	1.557	0.431	0	1.563	2.439	0.123	0.093	0.669
HKWinPort2	0.560	0	1.332	0	1.528	1.329	0.029	0.578	0	3.539	0.939	0.860	0.482	0	1.362	0.378	0	0	1.022
HKWinPort3	0	0	1.742	0.129	2.163	1.411	0.028	1.026	0	3.798	0.735	1.332	0.562	0	1.887	0.980	0	0	0.838
HKWinPort5	0	0	0.648	0.196	3.357	1.252	0.042	0.558	0	3.273	0.248	1.266	0.545	0	1.524	1.305	0.125	0	0.537
HKWinWWTP1	1.039	0.373	16.544	0	6.209	4.735	0.147	4.796	0	3.294	0.477	3.724	0.805	0	17.078	9.470	0	0.068	1.734
HKWinWWTP2	0	0.326	11.451	0.334	1.861	5.073	0.081	4.686	0	2.893	0.715	1.820	1.141	0.344	28.836	17.166	0.105	0.191	1.226
HKWinWWTP3	0.818	0.604	54.558	0.432	5.096	12.083	0.159	9.748	0	0.993	1.131	1.499	2.233	0.418	68.783	23.466	0	0.690	5.516
HKWinWWTP4	0.418	0.651	50.770	0.689	6.857	14.213	0.251	10.752	0	0.848	0.800	1.162	2.345	0.497	96.365	43.089	0	0.753	5.040
QDSumBeach3	0	0.344	2.233	0.680	1.513	0.165	0.095	0.324	0	1.584	1.550	2.815	0.777	0.170	2.081	2.285	0	0	2.022
QDSumBeach4	0	0	0	0.250	1.410	0.159	0.093	0.256	0	2.628	1.195	1.816	0.679	0	1.337	0.973	0	0	2.686
QDSumBeach5	0	0	0	0	1.428	0.163	0.156	1.075	0	0.530	3.741	2.536	1.070	0.182	2.149	0	0	0	1.963
QDSumEs1	4.044	0	2.384	0.141	6.382	0.895	1.094	1.651	0	3.828	1.873	3.857	1.325	0	5.012	1.115	0.182	6.950	3.730
QDSumEs2	4.809	0.424	3.177	0.422	14.408	1.529	1.423	1.679	0.315	8.006	2.830	4.633	1.602	0.223	5.083	0	0	9.007	3.448
QDSumEs3	2.694	0	3.605	0.361	5.732	1.151	1.036	2.157	0.241	7.332	2.283	3.811	1.565	0	5.334	0	0	6.421	3.087
QDSumMc1	0.704	0.465	0	1.684	5.099	0.323	0.233	0.906	0	3.687	2.226	3.413	1.199	0	1.173	1.311	0	1.392	1.067
QDSumMc2	0	0.300	3.491	0.917	7.227	0.440	0.181	1.280	0	3.786	2.071	4.148	1.466	0	0.522	0.446	0	1.163	2.154
QDSumMc4	0.298	0	1.411	0.405	5.424	0.278	0.233	0.725	0	3.414	1.609	3.438	1.304	0	1.621	0.487	0	0.944	2.518
QDSumMR1	0.279	0.273	1.241	0.336	3.184	0.124	0.058	0.774	0	0.539	1.583	2.811	0.661	0	0.870	0	0.102	0	0.607
QDSumMR2	0.497	0	1.771	0	1.260	0.146	0.129	0.796	0	0.904	1.455	2.700	0.855	0	2.113	3.147	0	0	0.273

QDSumMR3	0	0	3.222	0.714	4.639	0.117	0.070	0.399	0	0.624	1.314	3.354	1.051	0	1.694	1.057	0	0	0.357
QDSumPort1	0	0	0.656	0.433	3.720	0.284	0.178	1.820	0	0.371	2.147	1.952	0.747	0	2.227	1.975	0	0	0
QDSumPort2	0.266	0	0.872	0.559	2.084	0.484	0.234	1.149	0	0.340	10.759	2.353	0.925	0	3.703	1.546	0	0.200	0.107
QDSumPort3	0	0	0	0.262	3.002	0.404	0.193	0.893	0	0	1.245	2.299	0.649	0	1.959	1.052	0	0	0
QDSumPort4	0	0.272	0.611	0.222	3.613	0.573	0.324	1.533	0	0.735	1.823	2.103	0.867	0	3.936	0.565	0	0	0.806
QDSumPort5	0	0.266	0	0.717	3.900	0.410	0.272	0.776	0	1.101	5.253	2.917	0.951	0	1.703	0.545	0	0	0
QDSumWWTP2	0.560	0.520	2.211	1.230	1.845	1.091	0.555	1.458	0	1.036	9.504	2.734	0.803	0	13.403	1.081	0.108	0.559	1.067
QDSumWWTP3	0	0	1.481	0.878	3.703	1.041	0.643	1.704	0	0.767	1.328	1.968	0.894	0	9.936	1.218	0	0.166	0
QDSumWWTP5	0	0	0.990	0.266	2.931	0.435	0.231	0.660	0	1.015	1.548	3.241	0.586	0	4.829	0.500	0	0	0
QDWinBeach1	0	0	3.355	0.445	1.385	0.357	0.213	2.030	0	1.155	1.300	6.750	0.460	0	0.915	1.200	0.178	0.353	0.372
QDWinEs1	18.300	0	3.015	0.800	38.150	38.200	55.000	11.200	0.228	12.000	2.180	16.300	1.215	0.472	5.150	1.245	0	9.900	23.150
QDWinEs2	21.500	0.448	3.480	1.120	77.500	56.500	63.000	38.800	0.401	19.900	7.150	24.600	0.940	0.955	48.350	2.435	0.469	19.200	20.150
QDWinEs3	3.270	0	3.155	0.800	29.900	22.850	38.400	6.500	0	10.700	6.750	25.500	0.805	0.385	11.050	1.780	0.176	9.350	11.350
QDWinEs4	22.700	0.335	4.200	2.740	37.150	29.150	41.950	7.700	0.454	21.850	5.000	19.550	1.140	0.345	27.000	2.105	0.351	19.250	17.950
QDWinEs5	19.350	0	3.640	1.000	17.650	19.350	29.250	7.400	0	11.250	2.015	13.200	1.220	0.318	21.850	1.900	0.925	8.700	11.900
QDWinMc1	0	0	1.605	0.204	4.595	1.790	0.975	1.480	0	1.305	0.222	7.100	0.545	0	0.243	1.570	0.540	1.420	9.950
QDWinMc2	0	0	3.900	0.091	4.130	1.295	0.535	0.980	0	1.105	0.560	4.835	0.520	5.550	9.350	5.450	5.150	1.195	0.885
QDWinMc3	0	0	3.155	0.385	8.950	1.680	0.825	1.600	0	1.185	0.421	7.000	0.505	74.500	0.520	3.330	2.245	1.435	1.075
QDWinMc4	0	0	1.770	0.545	4.820	1.210	0.915	1.485	0	2.270	0.471	5.700	0.459	4.775	0.430	2.970	1.080	2.255	1.025
QDWinMc5	0	0	1.560	0.425	4.040	1.355	2.300	0.845	0	1.580	0.468	6.750	0.382	1.595	0	1.735	0.241	1.255	7.700
QDWinMR1	0	0	3.165	1.060	3.770	0.122	0.175	2.910	0	1.025	0.254	7.350	0.386	0	0.376	1.505	0.192	0.530	0.815
QDWinMR2	0	0	1.885	0.755	3.020	0.077	0.083	1.350	0	0.945	0.316	7.750	0.439	0	0.505	0.495	0.131	0.374	0.655
QDWinMR3	0	0	2.230	0.715	1.435	0.074	0.155	1.380	0	0.925	0.615	4.355	0.378	0	0.880	0.640	5.700	0.344	0.505
QDWinMR4	0	0	1.460	0.354	2.565	0.068	0.232	1.815	0	0.985	15.400	7.700	0.387	0.305	1.255	0.810	0.373	0.266	2.825
QDWinPort1	0	0	3.140	0.640	1.605	0.284	0.384	1.385	0	1.020	10.900	8.150	0.425	0.264	5.050	1.245	0.183	0.385	0.860
QDWinPort2	0	0	0.810	0.675	0.940	0.316	0.620	2.485	0	1.820	9.850	7.850	0.403	0.460	9.250	0.715	0.735	0.476	2.745
QDWinPort3	0	0	0.520	0.900	2.395	0.369	0.194	2.335	0	1.170	11.150	5.300	0.488	0.312	1.570	1.205	0.150	0.510	0.525
QDWinPort4	0	0	0.725	0.720	1.635	0.450	0.424	2.630	0	1.345	11.900	7.200	0.371	1.350	3.800	1.135	0.525	0.380	7.850
QDWinPort5	0	0	1.365	1.040	1.400	0.605	0.990	2.670	0	0.610	0.885	5.700	0.497	0.166	4.840	0.885	0.192	0.392	0.615
QDWinWWTP1	0	0	2.675	0.257	4.205	1.805	1.190	2.405	0	2.125	0.464	8.150	0.890	0	0.825	0.835	0.250	1.065	1.155
QDWinWWTP2	0	0	1.550	0.128	1.665	1.480	1.230	2.270	0.478	2.090	0.730	12.500	0.800	0.162	0.895	1.945	0.187	1.005	1.050
QDWinWWTP3	0	0	5.800	0.149	3.685	1.460	1.175	2.640	0	2.470	1.190	9.100	0.650	0	0.575	0	0	1.595	1.080
QDWinWWTP4	0	0	3.065	0.092	1.855	1.415	0.880	2.330	0.260	2.430	1.300	7.900	0.487	0	1.865	0.585	0.393	1.260	1.050
QDWinWWTP5	0	0	2.155	0.201	3.335	1.210	1.005	2.865	0.375	2.445	1.265	8.250	0.409	0.299	0.825	0.965	0.167	1.620	1.025

Table S3 Published marine metagenomic datasets included in the meta-analysis. ENA = European Nucleotide Archive; NCBI = National Center for Biotechnology Information

Study ID	Accession number	Sample type	Source
wos_50	ENA: PRJEB37023	plastic, wood	Ref 17
wos_85	ENA: ERP017130	plastic, seawater, ceramic	Ref 18
wos_2	ENA: ERP124866	plastic, wood	Ref 19
wos_116	NCBI: PRJNA917516	plastic, freshwater, seawater, metal	Ref 20
wos_134	NCBI: PRJNA777294	plastic, cardboard, wood	Ref 21
wos_390	NCBI: PRJNA981336	plastisphere, wood	Ref 22
pub_345	NCBI: PRJNA1021400	plastisphere, seawater, natural organic particles	Ref 23

Table S4 Sample information for the meta-analysis.

Sample ID	Study ID	Surface type	Latitude	Longitude	Polymer type
ERR3986765	wos_50	wood	54.146	11.843	-
ERR3986938	wos_50	wood	54.146	11.843	-
ERR3987103	wos_50	wood	54.146	11.843	-
ERR3987193	wos_50	wood	54.146	11.843	-
ERR3987194	wos_50	plastic	54.146	11.843	PE
ERR3987232	wos_50	plastic	54.146	11.843	PE
ERR3987233	wos_50	plastic	54.146	11.843	PE
ERR3987274	wos_50	plastic	54.146	11.843	PE
ERR3987275	wos_50	plastic	54.146	11.843	PS
ERR3987276	wos_50	plastic	54.146	11.843	PS
ERR3987277	wos_50	plastic	54.146	11.843	PS
ERR3987278	wos_50	plastic	54.146	11.843	PS
ERR1995156	wos_85	plastic	27.544	-97.285	PHA
ERR1995157	wos_85	plastic	27.544	-97.285	PHA
ERR1995158	wos_85	plastic	27.544	-97.285	PHA
ERR1995198	wos_85	ceramic	27.544	-97.285	ceramic
ERR1995199	wos_85	ceramic	27.544	-97.285	ceramic
ERR1995200	wos_85	ceramic	27.544	-97.285	ceramic
ERR1995201	wos_85	plastic	27.544	-97.285	PET
ERR1995202	wos_85	plastic	27.544	-97.285	PET
ERR1995203	wos_85	plastic	27.544	-97.285	PET
ERR1995204	wos_85	seawater	27.544	-97.285	-
ERR1995205	wos_85	seawater	27.544	-97.285	-
ERR1995206	wos_85	seawater	27.544	-97.285	-
ERR4801148	wos_2	plastic	-33.142	151.537	PP
ERR4801149	wos_2	plastic	-33.142	151.537	PCL
ERR4801150	wos_2	plastic	-32.998	151.586	PCL
ERR4801151	wos_2	plastic	-33.084	151.522	PCL
ERR4801152	wos_2	wood	-33.142	151.537	-
ERR4801153	wos_2	wood	-32.998	151.586	-
ERR4801154	wos_2	wood	-33.084	151.522	-
ERR4801155	wos_2	seawater	-33.142	151.537	-
ERR4801156	wos_2	plastic	-32.998	151.586	PP
ERR4801157	wos_2	plastic	-33.084	151.522	PP
ERR4801158	wos_2	plastic	-33.142	151.537	PS
ERR4801159	wos_2	plastic	-32.998	151.586	PS
ERR4801160	wos_2	plastic	-33.084	151.522	PS
ERR4801161	wos_2	plastic	-33.142	151.537	PVC
ERR4801162	wos_2	plastic	-32.998	151.586	PVC
ERR4801163	wos_2	plastic	-33.084	151.522	PVC
SRR17458603	wos_134	plastic	27.06	-79.95	PS

SRR17458604	wos_134	seawater	33.76	-76.55	-
SRR17458605	wos_134	plastic	31.18	-79.5	PE
SRR17458606	wos_134	plastic	25.86	-68.99	HDPE
SRR17458607	wos_134	plastic	31.18	-79.5	PP
SRR17458608	wos_134	plastic	26.73	-79.89	PP
SRR17458609	wos_134	plastic	26.73	-79.89	PE
SRR17458610	wos_134	plastic	38.58	-76.42	PHA
SRR17458611	wos_134	plastic	37.89	-76.13	PHA
SRR17458612	wos_134	plastic	25.89	-69.88	PE
SRR17458613	wos_134	plastic	26.73	-79.89	PE
SRR17458614	wos_134	plastic	37.89	-76.17	PP
SRR17458615	wos_134	plastic	22.56	-63.73	PHA
SRR17458616	wos_134	plastic	22.56	-63.73	PS
SRR17458617	wos_134	plastic	22.56	-63.73	HDPE
SRR17458618	wos_134	wood	37.89	-76.17	-
SRR17458619	wos_134	plastic	22.56	-63.73	HDPE
SRR17458620	wos_134	plastic	22.56	-63.73	HDPE
SRR17458621	wos_134	plastic	22.56	-63.73	HDPE
SRR17458622	wos_134	plastic	22.56	-63.73	HDPE
SRR17458623	wos_134	plastic	31.18	-79.5	PE
SRR17458624	wos_134	seawater	36.9	-75.93	-
SRR17458625	wos_134	Cardboard	37.89	-76.17	-
SRR17458626	wos_134	plastic	22.56	-63.73	HDPE
SRR17458627	wos_134	plastic	24.43	-65.55	HDPE
SRR17458628	wos_134	wood	37.89	-76.17	-
SRR17458629	wos_134	plastic	36.9	-75.93	PHA
SRR17458630	wos_134	plastic	24.95	-66.35	PP
SRR17458631	wos_134	plastic	36.1	-75.55	PHA
SRR17458632	wos_134	plastic	24.95	-66.35	PE
SRR17458633	wos_134	seawater	31.16	-79.52	-
SRR17458634	wos_134	plastic	25.89	-69.88	PE
SRR17458635	wos_134	plastic	22.56	-63.73	HDPE
SRR17458636	wos_134	plastic	22.56	-63.73	HDPE
SRR17458637	wos_134	plastic	36.04	-75.52	PP
SRR17458638	wos_134	plastic	36.04	-75.52	PMMA
SRR17458639	wos_134	plastic	22.56	-63.73	HDPE
SRR17458640	wos_134	plastic	33.69	-76.62	PE
SRR17458641	wos_134	seawater	27.06	-79.95	-
SRR17458642	wos_134	plastic	33.69	-76.62	PE
SRR17458643	wos_134	plastic	33.45	-76.89	PE
SRR17458644	wos_134	plastic	33.45	-76.89	PE
SRR17458645	wos_134	plastic	25.89	-69.88	PE
SRR17458646	wos_134	plastic	30.69	-79.78	PE
SRR23008602	wos_116	seawater	38.97	117.83	-

SRR23008603	wos_116	metal	38.97	117.83	-
SRR23008604	wos_116	plastic	38.97	117.83	PLA
SRR23008605	wos_116	plastic	38.97	117.83	PET
SRR24907496	wos_390	plastic	32.826	34.956	-
SRR24907497	wos_390	wood	32.826	34.956	-
SRR26188895	pub_345	Natural organic particles	43.966	9.782	-
SRR26188896	pub_345	Natural organic particles	43.956	10.158	-
SRR26188897	pub_345	Natural organic particles	44.056	9.823	-
SRR26188898	pub_345	seawater	43.966	9.782	-
SRR26188899	pub_345	seawater	43.956	10.158	-
SRR26188900	pub_345	seawater	44.055	9.823	-
SRR26188901	pub_345	plastic	43.966	9.782	-
SRR26188902	pub_345	plastic	43.956	10.158	-
SRR26188903	pub_345	plastic	44.055	9.823	-

Table S5 Performance of random-forest models at different taxonomic levels.

Taxonomy	Accuracy	Feature number	Error rate
phylum	67.61%	8	32.39%
class	75.00%	8	25.00%
order	78.98%	19	21.02%
family	80.68%	24	19.32%
genus	85.23%	64	14.77%

References

1. Wang, B., Qin, W., Ren, Y., Zhou, X., Jung, M.-Y., Han, P., Eloe-Fadrosh, E.A., Li, M., Zheng, Y., Lu, L. et al. (2019). Expansion of Thaumarchaeota habitat range is correlated with horizontal transfer of ATPase operons. ISME J. *13*, 3067-3079.

2. Borrel, G., Brugère, J.-F., Gribaldo, S., Schmitz, R.A. and Moissl-Eichinger, C. (2020). The host-associated archaeome. Nat. Rev. Microbiol. *18*, 622-636.

3. Lynch, M.D.J. and Neufeld, J.D. (2015). Ecology and exploration of the rare biosphere. Nat. Rev. Microbiol. *13*, 217-229.

4. Mouillot, D., Bellwood, D.R., Baraloto, C., Chave, J., Galzin, R., Harmelin-Vivien, M., Kulbicki, M., Lavergne, S., Lavorel, S., Mouquet, N. et al. (2013). Rare species support vulnerable functions in high-diversity ecosystems. PLoS Biol. *11*, e1001569.

5. Lennon, J.T. and Jones, S.E. (2011). Microbial seed banks: the ecological and evolutionary implications of dormancy. Nat. Rev. Microbiol. *9*, 119-130.

6. Li, C., Jin, L., Zhang, C., Li, S., Zhou, T., Hua, Z., Wang, L., Ji, S., Wang, Y., Gan, Y. et al. (2023). Destabilized microbial networks with distinct performances of abundant and rare biospheres in maintaining networks under increasing salinity stress. iMeta *2*, e79.

7. Li, C., Wang, L., Ji, S., Chang, M., Wang, L., Gan, Y. and Liu, J. (2021). The ecology of the plastisphere: Microbial composition, function, assembly, and network in the freshwater and seawater ecosystems. Water Research *202*, 117428.

8. Bowers, R.M., Kyrpides, N.C., Stepanauskas, R., Harmon-Smith, M., Doud, D., Reddy, T.B.K., Schulz, F., Jarett, J., Rivers, A.R., Eloe-Fadrosh, E.A. et al. (2017). Minimum information about a single amplified genome (MISAG) and a metagenome-assembled genome (MIMAG) of bacteria and archaea. Nature Biotechnology *35*, 725-731.

9. Rocha, U., Kasmanas, J.C., Toscan, R., Sanches, D.S., Magnusdottir, S. and Saraiva, J.P. (2024). Simulation of 69 microbial communities indicates sequencing depth and false positives are major drivers of bias in prokaryotic metagenome-assembled genome recovery. PLoS Comput. Biol. *20*, e1012530.

10. Hatzenpichler, R. (2012). Diversity, physiology, and niche differentiation of ammonia-oxidizing archaea. Appl. Environ. Microbiol. *78*, 7501-7510.

11. Tu, Q., Lin, L., Cheng, L., Deng, Y. and He, Z. (2019). NCycDB: a curated integrative database for fast and accurate metagenomic profiling of nitrogen cycling genes. Bioinformatics *35*, 1040-1048.

12. Tourna, M., Stieglmeier, M., Spang, A., Könneke, M., Schintlmeister, A., Urich, T., Engel, M., Schloter, M., Wagner, M., Richter, A. et al. (2011). *Nitrososphaera viennensis*, an ammonia oxidizing archaeon from soil. Proceedings of the National Academy of Sciences *108*, 8420-8425.

13. Stuehrenberg, J., Kitzinger, K., von Arx, J.N., Graf, J.S., Lavik, G., Littmann, S., Milucka, J., Orsi, W.D., Schorn, S., Speth, D.R. et al. (2025). Urea use drives niche separation between dominant marine ammonia oxidizing archaea. Nat. Commun. *16*, 10946.

14. Kobayashi, S., Hira, D., Yoshida, K., Toyofuku, M., Shida, Y., Ogasawara, W., Yamaguchi, T., Araki, N. and Oshiki, M. (2018). Nitric oxide production from nitrite reduction and hydroxylamine oxidation by copper-containing dissimilatory nitrite reductase (NirK) from the aerobic ammonia-oxidizing archaeon, *Nitrososphaera viennensis*. Microbes and Environments *33*, 428-434.

15. Wu, L., Chen, X., Wei, W., Liu, Y., Wang, D. and Ni, B.-J. (2020). A critical review on nitrous oxide production by ammonia-oxidizing archaea. Environ. Sci. Technol. *54*, 9175-9190.

16. Rinke, C., Rubino, F., Messer, L.F., Youssef, N., Parks, D.H., Chuvochina, M., Brown, M., Jeffries, T., Tyson, G.W., Seymour, J.R. et al. (2019). A phylogenomic and ecological analysis of the globally abundant Marine Group II archaea (*Ca.* Poseidoniales ord. nov.). ISME J. *13*, 663-675.

17. Oberbeckmann, S., Bartosik, D., Huang, S.X., Werner, J., Hirschfeld, C., Wibberg, D., Heiden, S.E., Bunk, B., Overmann, J., Becher, D. et al. (2021). Genomic and proteomic profiles of biofilms on microplastics are decoupled from artificial surface properties. Environ. Microbiol. *23*, 3099-3115.

18. Pinnell, L.J. and Turner, J.W. (2019). Shotgun metagenomics reveals the benthic microbial community response to plastic and bioplastic in a coastal marine environment. Frontiers in Microbiology *10*, 13.

19. Bhagwat, G., Zhu, Q., O’Connor, W., Subashchandrabose, S., Grainge, I., Knight, R. and Palanisami, T. (2021). Exploring the composition and functions of plastic microbiome using whole-genome sequencing. Environ. Sci. Technol. *55*, 4899-4913.

20. Zhao, X., Niu, Z., Ma, Y., Zhang, Y. and Li, Y. (2023). Metagenomic insights into the potential risks of representative bio/non-degradable plastic and non-plastic debris in the upper and lower reaches of Haihe Estuary, China. Sci. Total Environ. *887*, 164026.

21. Bos, R.P., Kaul, D., Zettler, E.R., Hoffman, J.M., Dupont, C.L., Amaral-Zettler, L.A. and Mincer, T.J. (2023). Plastics select for distinct early colonizing microbial populations with reproducible traits across environmental gradients. Environ. Microbiol. *25*, 2761-2775.

22. Rubin-Blum, M., Yudkovsky, Y., Marmen, S., Raveh, O., Amrani, A., Kutuzov, I., Guy-Haim, T. and Rahav, E. (2023). Tar patties are hotspots of hydrocarbon turnover and nitrogen fixation during a nearshore pollution event in the oligotrophic southeastern Mediterranean Sea. Mar. Pollut. Bull. *197*, 115747.

23. Di Cesare, A., Sathicq, M.B., Sbaffi, T., Sabatino, R., Manca, D., Breider, F., Coudret, S., Pinnell, L.J., Turner, J.W. and Corno, G. (2024). Parity in bacterial communities and resistomes: Microplastic and natural organic particles in the Tyrrhenian Sea. Mar. Pollut. Bull. *203*, 116495.
