# Supplementary material for: Unraveling the coastal marine plastisphere archaeome
Source: Nat Commun. 2026 Aug 14;17:9780. doi: 10.1038/s41467-026-76782-z (PMC13572444; doi:10.1038/s41467-026-76782-z)
Supplement: Supplementary file 2 [file 41467_2026_76782_MOESM2_ESM.pdf]

nature portfolio

Corresponding author(s): Changchao Li; James Kar-Hei Fang; Ling N. Jin

Last updated by author(s): Jun 24, 2026

Reporting Summary

Nature Portfolio wishes to improve the reproducibility of the work that we publish. This form provides structure for consistency and transparency in reporting. For further information on Nature Portfolio policies, see our Editorial Policies and the Editorial Policy Checklist.

Statistics

For all statistical analyses, confirm that the following items are present in the figure legend, table legend, main text, or Methods section.

n/a	Confirmed	
☐	☒	The exact sample size (n) for each experimental group/condition, given as a discrete number and unit of measurement
☐	☒	A statement on whether measurements were taken from distinct samples or whether the same sample was measured repeatedly
☐	☒	The statistical test(s) used AND whether they are one- or two-sided<br>*Only common tests should be described solely by name; describe more complex techniques in the Methods section.*
☐	☒	A description of all covariates tested
☐	☒	A description of any assumptions or corrections, such as tests of normality and adjustment for multiple comparisons
☐	☒	A full description of the statistical parameters including central tendency (e.g. means) or other basic estimates (e.g. regression coefficient) AND variation (e.g. standard deviation) or associated estimates of uncertainty (e.g. confidence intervals)
☐	☒	For null hypothesis testing, the test statistic (e.g. F, t, r) with confidence intervals, effect sizes, degrees of freedom and P value noted<br>*Give P values as exact values whenever suitable.*
☒	☐	For Bayesian analysis, information on the choice of priors and Markov chain Monte Carlo settings
☒	☐	For hierarchical and complex designs, identification of the appropriate level for tests and full reporting of outcomes
☐	☒	Estimates of effect sizes (e.g. Cohen's d, Pearson's r), indicating how they were calculated

Our web collection on statistics for biologists contains articles on many of the points above.

Software and code

Policy information about availability of computer code

Data collection	No software was used.
Data analysis	Raw amplicon sequences were processed using an open-source pipeline (EasyAmplicon, https://github.com/YongxinLiu/EasyAmplicon) based on USEARCH v10.0.240/VSEARCH v2.15.0. Databases for 16S amplicon data included RDP train set v16 and UNITE database v10.0. Raw metagenomic reads were processed with the EasyMetagenome pipeline (https://github.com/YongxinLiu/EasyMetagenome) with software packages including fastp v0.23.2, MEGAHIT v1.2.9, Prodigal v2.6.3, CD-HIT v4.8.1, Salmon v1.10.1, MMseqs2, and DIAMOND v2.1.6 and databases including GTDB, CCycDB, MCycDB, NCycDB, SCycDB, and PCycDB. We used R v4.2.2 for statistical analyses and plotting with R packages including ggplot2 v3.5.1, vegan v2.6-4, picante v1.8.2, randomForest v4.7-1.1, NST v3.1.10, iCAMP v1.5.12, igraph v1.4.1, and ggClusterNet v2.0. Scripts employed in the computation analyses are available under the following link: https://github.com/Changchao-Li/plastisphere_archaea.

For manuscripts utilizing custom algorithms or software that are central to the research but not yet described in published literature, software must be made available to editors and reviewers. We strongly encourage code deposition in a community repository (e.g. GitHub). See the Nature Portfolio guidelines for submitting code & software for further information.

Data

Policy information about availability of data

All manuscripts must include a data availability statement. This statement should provide the following information, where applicable:

- Accession codes, unique identifiers, or web links for publicly available datasets
- A description of any restrictions on data availability
- For clinical datasets or third party data, please ensure that the statement adheres to our policy

Amplicon sequencing data have been deposited in NCBI under BioProject IDs PRJNA1307274 (archaea), PRJNA1307280 (bacteria), and PRJNA1307281 (fungi). Shotgun metagenomic sequences are available under BioProject ID PRJNA1307277. Source data are provided with this paper.

Research involving human participants, their data, or biological material

Policy information about studies with human participants or human data. See also policy information about sex, gender (identity/presentation), and sexual orientation and race, ethnicity and racism.

Reporting on sex and gender	n/a
Reporting on race, ethnicity, or other socially relevant groupings	n/a
Population characteristics	n/a
Recruitment	n/a
Ethics oversight	n/a

Note that full information on the approval of the study protocol must also be provided in the manuscript.

Field-specific reporting

Please select the one below that is the best fit for your research. If you are not sure, read the appropriate sections before making your selection.

☐ Life sciences ☐ Behavioural & social sciences ☒ Ecological, evolutionary & environmental sciences

For a reference copy of the document with all sections, see nature.com/documents/nr-reporting-summary-flat.pdf

Ecological, evolutionary & environmental sciences study design

All studies must disclose on these points even when the disclosure is negative.

Study description	To address the knowledge gap in archaeal plastisphere ecology, we collected paired plastic debris and adjacent surface-water samples from diverse coastal marine environments and performed both amplicon and metagenomic analyses. Our goal was to characterize archaeal community patterns and their ecological implications in the coastal plastisphere, thereby advancing understanding of archaeal ecology in this anthropogenic habitat and the potential threats posed by plastic pollution.
Research sample	We collected paired plastic debris and corresponding surface water samples from coastal marine environments. A total of 94 plastic debris samples and corresponding paired 94 bulk water samples were obtained. The plastic particles were identified using optical photothermal infrared (O-PTIR) spectroscopy. Environmental physicochemical parameters were measured. We extracted DNA from all samples and the archaeal, bacterial, fungal, and functional genes were investigated based on amplicon sequencing and metagenomics.
Sampling strategy	Floating plastic debris was collected using a Manta trawl (333 µm mesh) towed behind a boat. After approximately 30 minutes of trawling, all visible plastic pieces were picked out with sterilized tweezers from the material gathered in the net's collection cup and stored in sterile tubes. At least three replicate trawls were conducted at each site. Two liters of surface seawater were collected alongside each plastic sample as the corresponding bulk water sample. All samples were immediately stored on ice in the field, transported to the lab on the same day, and kept at 4 °C until further processing. In total, we obtained 94 plastic debris samples and corresponding paired 94 bulk water samples.
Data collection	Water temperature, pH, and salinity were measured in situ using a YSI multi-parameter probe. One liter of each water sample was filtered through a 0.45 µm membrane for subsequent analyses of nutrients and organic carbon. Dissolved organic carbon (DOC) was measured with an Elementar Acquray TOC analyzer. Nitrate and phosphate concentrations were determined with a Skalar San++ continuous flow analyzer. Nineteen types of antibiotics were measured (as indicators of human-derived chemical pollution) following established protocols.<br>For archaea, we amplified the 16S rRNA gene using archaea-specific primers Arch519F/Arch915R, which effectively capture archaeal diversity even at low abundance. For bacteria, the 16S rRNA gene (V3–V4 region) was amplified with primers 341F/806R, and for

	fungi, the ITS2 region with primers ITS3-2024F/ITS4-2409R. Sequencing libraries were quality-checked and paired-end sequenced (2 × 300 bp) on a DNBSEQ-2000 platform. For shotgun metagenomics, we constructed low-input libraries from 25–100 ng of DNA per sample and then sequenced on the DNBSEQ-2000 platform (150 bp paired-end), generating ≥ 10 Gb of data per sample.
Timing and spatial scale	We collected paired plastic debris and corresponding surface water samples along the coasts of Hong Kong and Qingdao, China, in August 2021 and February 2022.
Data exclusions	To normalize sequencing depth across samples, we rarefied the archaeal dataset to 8,000 reads per sample, the bacterial dataset to 30,000 reads, and the fungal dataset to 5,700 reads. Samples with fewer reads than these thresholds were removed. Additionally, any sample lacking its paired plastic or water counterpart was removed to maintain a 1:1 plastisphere–bulk pairing. This yielded 88 paired plastisphere and water samples for archaea (176 samples total), 92 pairs for bacteria, and 51 pairs for fungi. We excluded any metagenomes that lacked corresponding amplicon data or a paired sample. This yielded 82 paired plastisphere and water metagenomes (164 total metagenomic samples).
Reproducibility	Sampling was conducted in two seasons (dry and wet), across two regions (subtropical Hong Kong and temperate Qingdao, China), and encompassed diverse coastal habitat types within each region (beaches, urban river estuaries, mariculture sites, ports, marine reserves, and sites near wastewater outfalls), thereby capturing a wide range of environmental conditions and human impacts. For every analysis, plastisphere and bulk water samples were paired 1:1, with at least 51 replicates included in each group. These practices ensured the reproducibility and robustness of our findings.
Randomization	Randomization was not applicable to this study because it was based on field-collected environmental samples rather than experimental treatments. Sampling locations were chosen to capture diverse coastal habitat types across two geographic regions and two seasons, and replicate samples were collected at each site to ensure representativeness. All samples were processed and analyzed using standardized laboratory and computational protocols applied uniformly to all groups.
Blinding	Blinding was not applicable, as plastisphere and bulk water samples had to be identified during field collection and laboratory processing. However, all sequencing and bioinformatic analyses were conducted using standardized pipelines, which minimized potential bias due to knowledge of sample identity.

Did the study involve field work? ☒ Yes ☐ No

Field work, collection and transport

Field conditions	Sampling was carried out in coastal marine environments under natural field conditions encompassing a wide range of environmental conditions. Plastic debris and bulk seawater samples were collected in situ without manipulation, preserving authentic community composition and environmental context.
Location	Samples were collected along the coasts of Hong Kong (subtropical) and Qingdao, China (temperate), covering beaches, urban river estuaries, mariculture sites, ports, marine reserves, and areas near wastewater outfalls. Floating plastic debris and water samples were collected at the marine surface (<1m).
Access & import/export	Field sampling was conducted under institutional approval and in compliance with local regulations. Sample collection did not require special permits, and all materials remained within China; no international import/export of biological specimens was involved.
Disturbance	Sampling was non-invasive and involved only the collection of surface plastic debris and seawater. No disturbance to habitats, organisms, or protected areas occurred beyond the removal of floating plastic litter.

Reporting for specific materials, systems and methods

We require information from authors about some types of materials, experimental systems and methods used in many studies. Here, indicate whether each material, system or method listed is relevant to your study. If you are not sure if a list item applies to your research, read the appropriate section before selecting a response.

Materials & experimental systems

n/a	Involved in the study	
☒	☐	Antibodies
☒	☐	Eukaryotic cell lines
☒	☐	Palaeontology and archaeology
☒	☐	Animals and other organisms
☒	☐	Clinical data
☒	☐	Dual use research of concern
☒	☐	Plants

Methods

n/a	Involved in the study	
☒	☐	ChIP-seq
☒	☐	Flow cytometry
☒	☐	MRI-based neuroimaging

Plants

Seed stocks	n/a
Novel plant genotypes	n/a
Authentication	n/a
