# Supplementary material for: Unraveling the coastal marine plastisphere archaeome
Source: Nat Commun. 2026 Aug 14;17:9780. doi: 10.1038/s41467-026-76782-z (PMC13572444; doi:10.1038/s41467-026-76782-z)
Supplement: Supplementary file 3 [file 41467_2026_76782_MOESM3_ESM.pdf]

nature portfolio

Peer Review File

Unraveling the coastal marine plastisphere archaeome

Corresponding Author: Dr Changchao Li

This file contains all reviewer reports in order by version, followed by all author rebuttals in order by version.

Attachments originally included by the reviewers as part of their assessment can be found at the end of this file.

Version 0:

Reviewer comments:

Reviewer #1

(Remarks to the Author)
This is an interesting and well-executed study that addresses a notable knowledge gap by focusing on the plastisphere's archaeal communities. The manuscript is very well written, with a smooth logical flow and excellent figures that greatly aid understanding. The introduction nicely sets up the context of plastic pollution and the oft-overlooked archaeal domain, and the multi-faceted approach (paired sampling across regions/seasons, amplicon + metagenomics) is comprehensive and robust. The results are novel and important for microbial ecology and environmental science. I was especially intrigued by the evidence that plastisphere archaea harbor unique biogeochemical functions (e.g., methane and nitrogen cycling genes) and appear less sensitive to environmental stressors, suggesting plastic biofilms may act as a microbial refuge under environmental stress. I am also impressed that this study not only investigated archaea in depth, but also considered bacteria and fungi in parallel, revealing a unique differentiative pattern in archaeal community responses compared to the other domains.

Overall, I support publication of this work. My comments below are aimed at further enhancing the clarity and impact of the manuscript. I have outlined several major suggestions that mostly involve expanding on interpretations and ensuring clarity in a few conceptual areas, followed by some minor comments to polish the text. Many of the minor comments are relatively small phrasing or formatting suggestions to improve readability.

Major comments
1. The authors present exciting evidence that plastisphere archaea carry genes for methane, nitrogen, sulfur, and phosphorus cycling, and they posit that plastics could alter nutrient cycles and greenhouse gas fluxes. While I agree these findings have intriguing implications, I encourage the authors to clarify the scope of these interpretations. In particular, it should be made clear that the study infers potential biogeochemical impacts based on genomic capacity, rather than direct process rate measurements. For instance, the discussion of methane turnover (lines 226-235) is fascinating, but the manuscript should explicitly acknowledge that no methane production or consumption rates were measured, so any net effect on CH_4 emissions remains hypothetical at this stage. This is not to detract from the importance of the finding, rather, framing it as a hypothesis for future research would prevent overstatement. Similarly, when discussing accelerated nitrogen removal or increased N_2O generation, I suggest adding a sentence noting that the magnitude of these impacts in situ is uncertain, perhaps encouraging follow-up studies to quantify actual fluxes.

2. The finding that archaeal community assembly is more strongly stochastic in the plastisphere, compared to bacterial or fungal assembly, is very interesting. To ensure readers grasp this point, the authors should expand the explanation of their assembly analysis and its implications. Currently, the text mentions this result (and Fig. 3) but could offer more context. For example, it would help to briefly explain why archaeal communities might be governed by dispersal limitation. The authors do hint at this, noting archaea's low abundance and broad tolerance combined with the "island effect" of plastic habitats. I encourage them to elaborate just a bit more: perhaps explicitly state that each plastic fragment acts as an isolated island that is initially colonized by whatever archaea happen to disperse to it, resulting in communities that differ greatly from one fragment to another (high β-diversity) and are less shaped by deterministic selection. A brief definition of terms like "dispersal limitation" and "homogeneous selection" in lay terms would also be helpful for readers not deeply familiar with community assembly theory.

3. One of the most intriguing findings is that plastisphere archaeal communities appear less sensitive to ambient environmental variation than bulk water communities, yet the difference between plastisphere and water communities becomes greater in more polluted conditions. I would like to see a bit more exploration of the mechanism and implications

here, for example, does this reduced sensitivity mean plastisphere communities could maintain functional stability in polluted waters (for better or worse)? Why the distinctiveness of plastisphere archaeal communities and functional profiles amplified by the increasing pollution stress? This finding is a novel ecological aspect of the plastisphere and is worth elaborating.

4. Regarding the methodology, I wonder if the authors have data on the types or sizes of plastic debris and whether that had any effect on archaeal communities. The paper treats the plastisphere generally, but different polymer types or surface textures might select for different microbes. If the dataset allowed analysis of community composition by plastic type (e.g., comparing polyethylene and polypropylene pieces) and no strong differences were found, the authors could state that for completeness. If such analysis was not feasible, that's understandable – but it might be worth acknowledging that plastic heterogeneity (polymer chemistry, age, etc.) could be a factor, even if in this study the plastisphere effect overall swamped any within-plastic variation. Furthermore, the authors might consider whether the observed differences are truly unique to "plastic" as a substrate, or whether any artificial substrate might yield similar patterns. In the Discussion or Conclusion, it could strengthen the paper to briefly note that while plastics create a novel and widespread habitat, some effects (e.g., increased surface-associated diversity) could also occur on natural floating substrates (wood, glass, etc.), though plastics have special properties.

Minor comments:

Title: Consider using American spelling for consistency, e.g., "Unraveling" (one "l") instead of "Unravelling". The manuscript text predominantly uses American English, so the title might be adjusted to match, unless journal style dictates otherwise.

Line 65: "the 'island effect' of the plastisphere habitat" – this metaphor is great, but the wording could be clarified. Perhaps "the isolated 'island' nature of the plastisphere habitat" or "the plastisphere's 'island effect' (i.e., each plastic piece acting as an isolated microhabitat)". A brief explanation of "island effect" would ensure all readers understand the concept.

Line 100: "effective, adoptive management strategies" – This seems to be a small typo: it should likely read "adaptive management strategies." Adaptive management is a common term in environmental science, whereas "adoptive" is not usually used in this context.

Lines 126-133: Excellent that sampling was done across two regions and two seasons. To give the reader a better sense of the dataset up front, consider stating the total number of samples (plastisphere vs bulk water) here if possible. For example, "sampling was conducted in two seasons… yielding X plastic debris samples and X adjacent water samples in total." This would complement the mention of diverse habitats and underline how robust the sampling effort was.

Line 169: "Thaumarchaeota, Woesearchaeota, and Pacearchaeota were significantly depleted". However, according to Fig. 1d, Thaumarchaeota showed no significant difference. Please verify whether this result was statistically significant.

Lines 175-177: "capacity to harbor archaeal taxa that are either non-native to the local environment or too rare in the surrounding water to be detected". To avoid any potential misinterpretation, the authors might clarify what "non-native to the local environment" entails. Does it imply that some archaea found on the plastics may have originated from distant regions or unusual sources (e.g., terrestrial runoff or deep sea)? If the data identified any surprising taxa on plastics that one wouldn't expect in coastal water, it would be great to mention an example. If not, perhaps rephrase to "taxa that are not observed in the surrounding water" (which covers both truly allochthonous and simply rare ones).

Line 253: "Our data corroborates these trends from a coastal archaeal perspective." This should be "data corroborate" (plural verb agreement). Also, to improve the phrasing: maybe "Our data corroborate these trends from an archaeal perspective in coastal waters."

Lines 402-404: "the plastisphere's functional composition thus appears less sensitive to environmental variation". Perhaps use a term like "buffered" or "insulated" to convey the idea of reduced sensitivity. Additionally, line 419 mentions "Notably, several……to be enhanced under high levels of antibiotic pollution (Fig. 4e)." I think some text might be missing around "Notably, several…". If it's referring to several taxa or functions that only appear under high pollution, consider completing that thought.

Once again, I want to congratulate the authors on a novel and important study. These comments are mostly aimed at clarity and ensuring the manuscript's messaging is as strong as the data. I am excited about the findings and enthusiastically recommend publication after revision. The insights into the coastal plastisphere archaeome open up new questions and directions for research, and this paper will likely become a foundational reference for the topic.

Reviewer #2

(Remarks to the Author)
Dear Wang et al.,

I have attached my comments as a PDF.

Reviewer #4

(Remarks to the Author)
The present study by Wang et al. addresses an important topic: the impact of plastic contamination on microbial communities and their metabolic potentials in coastal marine systems. The authors sampled many different coastal environments and collected plastic debris along with surrounding water to compare planktonic and plastic attached microbiomes by a metagenomic approach. The authors thereby focus on Archaea rather than Bacteria. The strengths of the study lay in the generation of a comprehensive data set for coastal marine systems, although Archaea have already been investigated in marine and terrestrial systems. However, the manuscript would greatly profit from acknowledging such studies and setting the results in context with findings from such studies that previously addressed Bacteria and Archaea. Furthermore, Archaea are generally lower in abundance in the plastisphere than Bacteria. In particular when metagenomes are considered, the rather low abundance of Archaea might result in a low number of reads. I would strongly advise to add some analysis on how good the Archaeal genes were captured (representativeness) to avoid conclusions based on data noise. A discussion on the relevance of Archaea relative to Bacteria would like wise strengthen the study.
The data presented here is based on DNA analysis, not allowing for conclusions on activities. Thus, many conclusions on activities are speculative and the manuscript would profit from toning down speculative statements.

Specific comments
L58 and throughout the manuscript. Please adhere to current taxonomy. Euryarchaeota are Methanobacteriota, …..
L60 “Archeal gene pathways”; gene pathways are not defined. Such phrasing might be clearer by using e.g. “genetic potentials for methanogenesis, …..” and naming the pathways. A “pathway for nitrogen or sulfur” is likewise unclear.
L66-67 Much speculation, would avoid in the abstract.
L68 Please provide evidence for the “hidden …. Impacts of plastic pollution”
L92-100 This paragraph would profit from considering current literature from other groups and eventually also other systems (marine, freshwater, terrestrial) that addressed plastisphere effects.
L108 There is previous evidence from literature, please add.
L153 Please explain the difference between taxonomic and phylogenetic diversity here.
L160 What kind of diversity does the betadiversity refer to? Taxonomic or phylogenetic? How does this relate to findings from other plastisphere studies? Were similar or contrasting findings obtained?
L165 and throughout the manuscript. Please consider current Taxonomy.
L180 How does this relate to previous findings of plastisphere studies?
L210-211 Please specify pathways. A pathway for methane might refer to methanogenesis or methanotrophy, one for nitrogen might refer to assimilation or dissimilation, etc.
L227-235 Highly speculative. Please provide activity/process or at least gene expression data. The genetic potential alone does not allow for conclusions on activity. I would expect that the plastisphere in coastal marine systems is largely subjected to light, thus also hosting oxygenic phototrophic Bacteria and Eukarya. Methanogens and AOM are obligate anaerobes that hardly thrive under oxic conditions.
L241 Please provide information on biofilm thickness and oxygenic phototrophs. Is there evidence that biofilms are of a size to allow for oxygen depletion?
L243 Are there nitrite oxidizing Archaea producing nitrate known?
L253-258 Speculative. Are there data on activities of Archaea on plastic surfaces?
L278 Are such organisms feeding on sulfur or also other compounds? Please clarify.
L284 This statement is not supported by the data. Please tone down.
L390-409 Very interesting analyses. Please give the concentrations of antibiotics measured and the other environmental parameters in the supplement and cite here. Would there be a causative relationship? Are the antibiotics concentrations inhibitory/ effective for Archaea?
L427 Might this be due to a simple life style change, i.e. that sessile particle associated organisms are more tolerant to environmental disturbance/ stressors irrespective of the support? If yes, this wouldn’t be a specific plastisphere effect.
L506 Cells were removed from plastic particles. Was the community structure of removed cells representative of the attached microbes? In a quantitative way, was the majority of cells removed?
L565-565 What was the likelihood of a correct assignment of genes/ sequences to Archea?

Version 1:

Reviewer comments:

Reviewer #1

(Remarks to the Author)
The auhtors have thoroughly revised paper according to reviewers' comments. I am satisfied with the revisions relevant to my previous comments and the paper could be considered for publication.

Reviewer #4

(Remarks to the Author)
Thanks to the authors for the thoughtful revision of the manuscript. The authors substantially improved the manuscript and pointed out that coverage of Archaeal taxa was sufficient to delineate meaningful conclusions by 16S rRNA amplicon sequence analyses. By linking the metagenomic analyses to such amplicon analyses, the validity of the metagenomics approach becomes apparent. However, the clarity could still be increased on how the metagenomic data were linked to the

amplicon data to increase the statistical significance of the functional inference analysis.
The limitations and future research needs are highlighted now and nicely presented in the conclusion section.

Specific comments
Previous comment to L58/ L165. My concerns relative to the current taxonomy have not been addressed to sufficient detail. I would encourage the authors to (i) use the same taxonomy for MAGs and marker gene analyses (important to bring meta-genomic and amplicon data together, see comment above), and (ii) update to the current taxonomy. New taxonomy always takes some time to become evident in current literature, but this time should be kept as small as possible. Thus, I don't see a reason for not changing to the current taxonomy only because other studies havn't done so … . In case the authors would like to keep relation to the old taxonomy, the old names might be provided in parentheses.
Previous comment to L92-100/108/180 and elsewhere. Nitrososphaera were also found enriched in the plastisphere – maybe this is a general trend across different habitats. In my view, it is important to delineate some general effects (or move towards a systematic understanding) of plastic on Archaeal communities and thus increase the impact of the study. This can only be done in case previous studies on Archaea in the plastisphere are included and findings compared in a concise way. Current literature coverage on Archaea in the plastispher might be helpful. Eventually, it would be better to place such a paragraph in the discussion. This will keep a concise style.
Previous comment to L253-258 (now L273-279). Could the nirK genes stem from ammonia oxidizers reflecting the potential for nitrifier denitrification rather than standard denitrification?
Previous comment to L506. The approach utilized to detach cells from plastic is referred to as being established. Is there any evidence from other studies on how complete the detachment is? The rarefaction analyses will not really help here … . In case there is no evaluation of detachment efficiencies possible, please acknowledge. This is not a major issue, since the same method was applied to all samples.

Open Access This Peer Review File is licensed under a Creative Commons Attribution 4.0 International License, which permits use, sharing, adaptation, distribution and reproduction in any medium or format, as long as you give appropriate credit to the original author(s) and the source, provide a link to the Creative Commons license, and indicate if changes were made.
In cases where reviewers are anonymous, credit should be given to 'Anonymous Referee' and the source.
The images or other third party material in this Peer Review File are included in the article's Creative Commons license, unless indicated otherwise in a credit line to the material. If material is not included in the article's Creative Commons license and your intended use is not permitted by statutory regulation or exceeds the permitted use, you will need to obtain permission directly from the copyright holder.
To view a copy of this license, visit https://creativecommons.org/licenses/by/4.0/

Responses to reviewers' comments

Comments are shown in black;
our responses are shown in blue;
text quoted from the revised manuscript is shown in pure blue.

Reviewer #1 (Remarks to the Author):

This is an interesting and well-executed study that addresses a notable knowledge gap by focusing on the plastisphere's archaeal communities. The manuscript is very well written, with a smooth logical flow and excellent figures that greatly aid understanding. The introduction nicely sets up the context of plastic pollution and the oft-overlooked archaeal domain, and the multi-faceted approach (paired sampling across regions/seasons, amplicon + metagenomics) is comprehensive and robust. The results are novel and important for microbial ecology and environmental science. I was especially intrigued by the evidence that plastisphere archaea harbor unique biogeochemical functions (e.g., methane and nitrogen cycling genes) and appear less sensitive to environmental stressors, suggesting plastic biofilms may act as a microbial refuge under environmental stress. I am also impressed that this study not only investigated archaea in depth, but also considered bacteria and fungi in parallel, revealing a unique differentiative pattern in archaeal community responses compared to the other domains.

Overall, I support publication of this work. My comments below are aimed at further enhancing the clarity and impact of the manuscript. I have outlined several major suggestions that mostly involve expanding on interpretations and ensuring clarity in a few conceptual areas, followed by some minor comments to polish the text. Many of the minor comments are relatively small phrasing or formatting suggestions to improve readability.

Response: Thank you very much for your kind recognition and appreciation of our work. We are deeply grateful for your insightful and detailed comments and suggestions. We have carefully revised the manuscript in response to your feedback, and we believe the revised version is significantly improved. We sincerely hope it now meets your expectations.

Major comments

1. The authors present exciting evidence that plastisphere archaea carry genes for methane,

nitrogen, sulfur, and phosphorus cycling, and they posit that plastics could alter nutrient cycles and greenhouse gas fluxes. While I agree these findings have intriguing implications, I encourage the authors to clarify the scope of these interpretations. In particular, it should be made clear that the study infers potential biogeochemical impacts based on genomic capacity, rather than direct process rate measurements. For instance, the discussion of methane turnover (lines 226-235) is fascinating, but the manuscript should explicitly acknowledge that no methane production or consumption rates were measured, so any net effect on CH_4 emissions remains hypothetical at this stage. This is not to detract from the importance of the finding, rather, framing it as a hypothesis for future research would prevent overstatement. Similarly, when discussing accelerated nitrogen removal or increased N_2O generation, I suggest adding a sentence noting that the magnitude of these impacts in situ is uncertain, perhaps encouraging follow-up studies to quantify actual fluxes.

Response: We thank you for this helpful suggestion. We agree that our data support inference of biogeochemical potential based on archaeal community composition and metagenomic functional profiles, rather than direct measurements of microbial activity or in situ process rates. To avoid overstatement while maintaining readability, we have taken a balanced approach in the revision. Specifically, we have softened process-level interpretations throughout the manuscript, especially in the Results and Discussion sections where methane and nitrogen cycling are discussed, and we have added a clear statement in the Conclusions and outlook section noting that the inferred biogeochemical implications are based on genomic potential and require future validation through direct rate- and activity-based measurements. We believe this revision clarifies the scope of our interpretations without introducing unnecessary repetition throughout the manuscript.

The updated Conclusions and outlook section is as follows:

The plastisphere represents a novel human-made habitat within the ocean microbiome that may redistribute microbial taxa and alter biogeochemical functional potential in marine systems. Recognizing these shifts is important for predicting the broader environmental impacts of plastic debris beyond the well-known direct hazards to wildlife. Here, we show that plastics host a restructured archaeal community characterized by higher taxonomic diversity and greater heterogeneity than the surrounding seawater. Correspondingly, archaeal functional gene profiles differ between the plastisphere and bulk water, with enrichment of pathways related to CH_4, N,

and S cycling in the plastisphere, highlighting the potential for plastic pollution to influence biogeochemical cycling[1, 2] and climate-related processes[3] from an archaeal perspective. As plastic waste continues to accumulate globally, the plastisphere will expand accordingly, leading to greater prevalence and density of archaeal communities in marine ecosystems, and thereby amplifying their ecological impacts.

We also found that stochastic processes predominantly govern archaeal community assembly in the plastisphere, underscoring the complexity of predicting these communities and the difficulty of mitigating their impacts through environmental management alone. Therefore, effectively addressing the impacts of plastic pollution will require upstream solutions such as reducing plastic production, improving recycling and reuse[4], advancing waste treatment technologies, and actively cleaning up existing plastic waste[5]. It is also important to identify the locations and time periods that should be prioritized for waste control and management, namely those in which plastic pollution is exerting particularly strong ecological impacts. An ambitious global plastic treaty could also help mitigate these impacts[5-7]. Beyond illuminating the impacts of plastic pollution, our work broadens understanding of how microorganisms respond to the proliferation of novel anthropogenic niches in the Anthropocene[8].

Nevertheless, because our analyses are based on taxonomic composition and metagenomic functional inference, direct links to viability, in situ activity, and realized biogeochemical rates remain to be established. In addition, the ecological implications highlighted here are based on relative comparisons rather than direct measurements of impact magnitude. For example, we still do not know to what extent plastic pollution contributes to microbial redistribution and invasion in the ocean, nor what magnitude of greenhouse-gas-related effects may be associated with plastisphere archaea. Further research is needed to directly link these microbial shifts to real-world ecological and health outcomes of plastic pollution, and to quantitatively assess the full risks of the plastisphere. Addressing these questions will require cross-disciplinary collaboration among researchers in environmental microbiology, environmental impact assessment, public health, and data science. Moreover, our study only considered surface waters and significant knowledge gaps remain regarding plastisphere dynamics throughout the water column and deep ocean ecosystems. Future work should also investigate the ecological mechanisms underlying plastisphere archaeal community assembly, including the roles of biofilm succession, substrate heterogeneity, and dispersal dynamics, to advance predictive understanding of how plastic pollution reshapes microbial diversity and function in marine ecosystems.

2. The finding that archaeal community assembly is more strongly stochastic in the plastisphere, compared to bacterial or fungal assembly, is very interesting. To ensure readers grasp this point, the authors should expand the explanation of their assembly analysis and its implications. Currently, the text mentions this result (and Fig. 3) but could offer more context. For example, it would help to briefly explain why archaeal communities might be governed by dispersal limitation. The authors do hint at this, noting archaea's low abundance and broad tolerance combined with the "island effect" of plastic habitats. I encourage them to elaborate just a bit more: perhaps explicitly state that each plastic fragment acts as an isolated island that is initially colonized by whatever archaea happen to disperse to it, resulting in communities that differ greatly from one fragment to another (high β-diversity) and are less shaped by deterministic selection. A brief definition of terms like "dispersal limitation" and "homogeneous selection" in lay terms would also be helpful for readers not deeply familiar with community assembly theory.

Response: We thank the reviewer for this helpful suggestion. We agree that the ecological meaning of the assembly analysis can be made clearer for a broader readership. In the revised manuscript, we have therefore added concise explanations of key community-assembly terms immediately after their first appearance in the text, including drift, dispersal limitation, heterogeneous selection, and homogeneous selection. We have also clarified the island-like nature of plastic particles by explicitly noting that each plastic fragment can act as an isolated habitat patch colonized by whichever archaeal cells happen to arrive and establish, thereby contributing to high between-particle heterogeneity and weaker deterministic filtering. We believe these revisions improve the accessibility and interpretation of Fig. 3 without overburdening the text.

The relevant parts have been revised as follows:

Chance-driven processes such as drift (random changes in community composition) and dispersal limitation (restricted arrival of taxa to local habitats) dominated archaeal composition, while bacteria and fungi were more strongly shaped by environmental filtering and biotic interactions. Archaea generally occur at relatively low abundance in coastal marine environments, comprising ~1% of total metagenomic reads from bacteria, fungi, and archaea combined in this study (Supplementary Fig. S2), and such low-abundance taxa are more susceptible to stochastic colonization and extinction events. (Lines 335 – 341)

We found that heterogeneous selection, in which different local environments favor different taxa, was minimal for archaeal communities in both plastisphere and bulk water (Fig. 3b,c). Instead, homogeneous selection, in which similar environmental conditions repeatedly favor similar taxa, influenced the most abundant archaeal taxa (e.g., bin3, dominated by Thaumarchaeota, which accounted for >66% of relative abundance) (Fig. 3b,c). (Lines 367 – 372)

3. One of the most intriguing findings is that plastisphere archaeal communities appear less sensitive to ambient environmental variation than bulk water communities, yet the difference between plastisphere and water communities becomes greater in more polluted conditions. I would like to see a bit more exploration of the mechanism and implications here, for example, does this reduced sensitivity mean plastisphere communities could maintain functional stability in polluted waters (for better or worse)? Why the distinctiveness of plastisphere archaeal communities and functional profiles amplified by the increasing pollution stress? This finding is a novel ecological aspect of the plastisphere and is worth elaborating.

Response: Thank you very much for this insightful comment. We agree that this is a particularly intriguing ecological pattern and that its potential mechanism and implications deserved further elaboration. In the revised manuscript, we have therefore expanded the discussion of this result to clarify why plastisphere archaeal communities and functional profiles may become increasingly distinct from those in bulk water under higher pollution stress. Specifically, we now discuss the possibility that the plastisphere provides a relatively sheltered microhabitat in which resident microorganisms are buffered, to some extent, from external chemical stress, whereas bulk-water archaeal communities are more directly exposed to surrounding conditions. Under this scenario, increasing anthropogenic stress would be expected to reshape bulk-water communities more strongly than plastisphere communities, thereby amplifying the taxonomic and functional divergence between them. We also note that this reduced environmental sensitivity may imply a relatively stable, and potentially functionally redundant, metabolic repertoire in plastisphere communities, with possible consequences for ecosystem functioning under polluted conditions. We have added these interpretations to the revised manuscript as follows:

These findings indicate that under higher environmental stress, plastisphere archaeal communities deviate more strongly from those in bulk water. One plausible explanation is that the plastisphere provides a sheltered microhabitat in which resident microorganisms are buffered, to

some extent, from external chemical stress, whereas bulk-water archaeal communities are more directly exposed to surrounding conditions[9]. Under this scenario, increasing stress would be expected to reshape bulk-water communities more strongly than plastisphere communities, thereby amplifying the taxonomic and functional divergence between them. Overall, increasing anthropogenic chemical stressors appear to be associated with more distinct plastisphere archaeal assemblages and greater plastisphere–bulk-water divergence in functional potential. These results suggest that, where chemical pollution co-occurs with plastic debris, the plastisphere may become an increasingly distinct and ecologically important microbial habitat.
(Lines 467 – 477)

4. Regarding the methodology, I wonder if the authors have data on the types or sizes of plastic debris and whether that had any effect on archaeal communities. The paper treats the plastisphere generally, but different polymer types or surface textures might select for different microbes. If the dataset allowed analysis of community composition by plastic type (e.g., comparing polyethylene and polypropylene pieces) and no strong differences were found, the authors could state that for completeness. If such analysis was not feasible, that's understandable – but it might be worth acknowledging that plastic heterogeneity (polymer chemistry, age, etc.) could be a factor, even if in this study the plastisphere effect overall swamped any within-plastic variation. Furthermore, the authors might consider whether the observed differences are truly unique to "plastic" as a substrate, or whether any artificial substrate might yield similar patterns. In the Discussion or Conclusion, it could strengthen the paper to briefly note that while plastics create a novel and widespread habitat, some effects (e.g., increased surface-associated diversity) could also occur on natural floating substrates wood, glass, etc.), though plastics have special properties.

Response: Thank you for these thoughtful comments and questions. In the present study, our primary aim was to identify the generalized ecological patterns of plastisphere archaea, treating the plastisphere as a novel anthropogenic habitat and evaluating its potential ecological implications at the habitat level. For this reason, we focused on the overall plastisphere signal rather than on within-plastic heterogeneity such as polymer type, size, or surface texture. In our view, these factors may indeed contribute to variation among plastic samples, but they represent secondary sources of heterogeneity relative to the broader question of whether plastics constitute a distinct microbial habitat compared with the surrounding environment.

This distinction is important conceptually. In microbial ecology, when one seeks to understand the general ecological characteristics of a habitat, the primary comparison is typically between that habitat and other habitats, rather than among all sources of within-habitat variation. Similarly, although polymer type, weathering state, or particle size may influence plastisphere communities to some extent, these factors do not preclude the possibility that plastics as a whole represent a distinct habitat for microorganisms. Indeed, as our previous work on global plastisphere bacterial communities has shown, variation within plastic samples can exist, but the contrast between plastic and non-plastic habitats remains the dominant signal when the comparison is made at a broader ecological scale[10].

To address the reviewer's additional concern regarding whether the observed archaeal patterns might simply reflect a general surface-associated lifestyle rather than a plastic-specific effect, we performed an additional meta-analysis of published marine metagenomic datasets containing both plastisphere samples and non-plastic solid-surface samples. This allowed us to test directly whether plastisphere archaeal communities are distinct from those on other substrates.

The added analyses, methods, results, and figures have been incorporated into the revised manuscript and Supplementary Information as follows:

To assess whether these compositional patterns reflect a plastic-specific archaeal signal rather than a general consequence of surface attachment, we performed a meta-analysis of published metagenomic datasets that included both plastisphere and non-plastic solid surfaces. Across studies, plastisphere archaeal communities remained compositionally distinct from those on other solid substrates (Stouffer weighted *Z*-test: $P < 0.01$; Supplementary Notes S5 and S6 and Figs. S10a and S11a), indicating that the observed archaeal community patterns are not merely a generic biofilm effect. (Lines 199 – 205 in the main text)

Consistent with the taxonomic meta-analysis above, a parallel analysis further showed that, despite considerable heterogeneity in study design, combined evidence supported the distinctiveness of plastisphere archaeal functional gene profiles relative to non-plastic substrates (Stouffer weighted *Z*-test: $Z = 2.35$, $P < 0.01$; Supplementary Notes S5 and S6 and Figs. S10b and S11b), suggesting that these functional patterns likewise reflect substrate-specific archaeal responses rather than a general surface-associated biofilm effect. (Lines 305 – 310 in the main text)

Supplementary Note S5 Plastisphere as an archaeal habitat distinct from other solid surfaces

To determine whether the distinctive archaeal assemblages observed on plastics represent a genuine substrate-specific signal rather than a general biofilm effect, we compared plastisphere archaeal communities with those on other solid surfaces, including wood, ceramic, metal, and cardboard, across five independent studies with sufficient replication, using both taxonomic composition and functional gene profiles as response variables.

For taxonomic composition (Supplementary Fig. S10a), four of the five studies showed greater between-group than within-group Bray–Curtis dissimilarity, indicating that plastisphere archaeal communities were generally more dissimilar from other-surface communities than they were internally variable. Two studies, wos_2 and wos_134, reached individual significance, whereas the others showed positive but non-significant trends, likely owing to limited within-study sample sizes. When combined across all five studies, Stouffer's weighted Z-test yielded $Z = 2.385$, $P < 0.01$, confirming consistent compositional separation of plastisphere archaeal communities from those on other solid surfaces across independent datasets.

A parallel pattern was observed for functional gene profiles (Supplementary Fig. S10b). Again, four of the five studies showed greater between-group than within-group Bray–Curtis dissimilarity, indicating that plastisphere samples harbored archaeal functional profiles distinct from those on other solid substrates. The combined Stouffer Z-test yielded $Z = 2.351$, $P < 0.01$, indicating that the functional distinctiveness of plastisphere archaeal communities was as consistent and statistically robust as their compositional distinctiveness.

Together, these results demonstrate that plastisphere archaeal communities differ from those on other solid surfaces at both taxonomic and functional levels, providing evidence that plastic substrates constitute a genuinely distinct archaeal habitat rather than simply another attachment surface. The convergence of compositional and functional signals across independent studies with diverse reference surfaces further supports the robustness of this conclusion.

Supplementary Note S6 Archaeome dissimilarity patterns across polymer types and substrate types

To evaluate whether polymer type structures plastisphere archaeal communities, we compared three categories of Bray–Curtis dissimilarity across four independent studies that included multiple polymer types and non-plastic reference substrates with known polymer identity (wos_50, wos_85, wos_2, and wos_134): within-polymer, between-polymer, and plastic-versus-non-plastic dissimilarity. Analyses were conducted for both archaeal taxonomic composition and functional gene profiles (Supplementary Fig. S11).

Overall, results were inconsistent across studies. In some datasets, plastic-versus-non-plastic dissimilarity exceeded between-polymer dissimilarity, suggesting that substrate identity explained more variation than polymer chemistry. In others, this pattern was not observed, and similar inconsistency was found for functional gene profiles. We attribute this lack of consistency primarily to the limited size and scope of the available datasets, including low replication per polymer type, narrow polymer diversity, sparse non-plastic reference groups, and the restricted ecological representativeness of incubation-based experiments.

These results indicate that currently available public datasets are insufficient to robustly disentangle polymer-type effects from broader substrate-identity effects in plastisphere archaeal communities. In contrast, the present study was designed to resolve the broader plastisphere signal under field conditions using a much larger set of paired plastisphere and ambient seawater samples across diverse coastal environments. We therefore interpret the consistent taxonomic and functional separation observed in our dataset as evidence of a generalizable plastisphere-specific archaeal signal, while recognizing that polymer-specific effects remain an important question for future work.

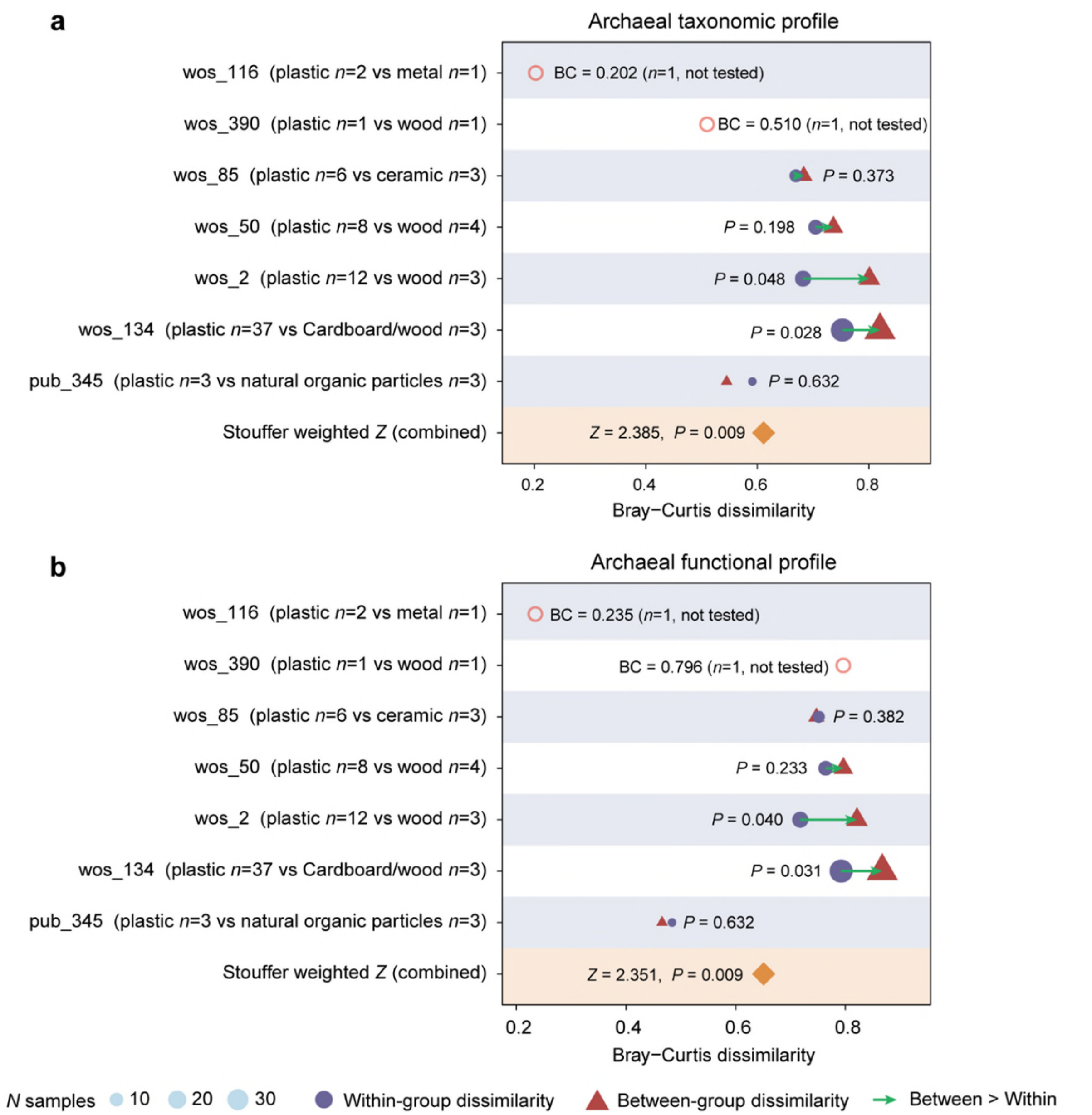

Fig. S10 Plastisphere archaeal communities are compositionally and functionally distinct from those on other solid surfaces. a, Taxonomic composition. **b,** Functional gene profiles. In both panels, red triangles indicate mean between-group Bray–Curtis dissimilarity (plastisphere versus other solid surfaces), and blue circles indicate mean within-group Bray–Curtis dissimilarity. Green arrows indicate studies in which between-group dissimilarity exceeded within-group dissimilarity. Point size is proportional to total sample size. For wos_116 and wos_390, within-group dissimilarity could not be calculated because of insufficient replication, and only between-group dissimilarity is shown descriptively (open circles). Statistical significance within each study was assessed using permutation tests, with the number of permutations limited to the total

number of possible label arrangements when this was <999. Combined evidence across the five testable studies was evaluated using Stouffer's weighted *Z*-method, weighting each study by the square root of total sample size.

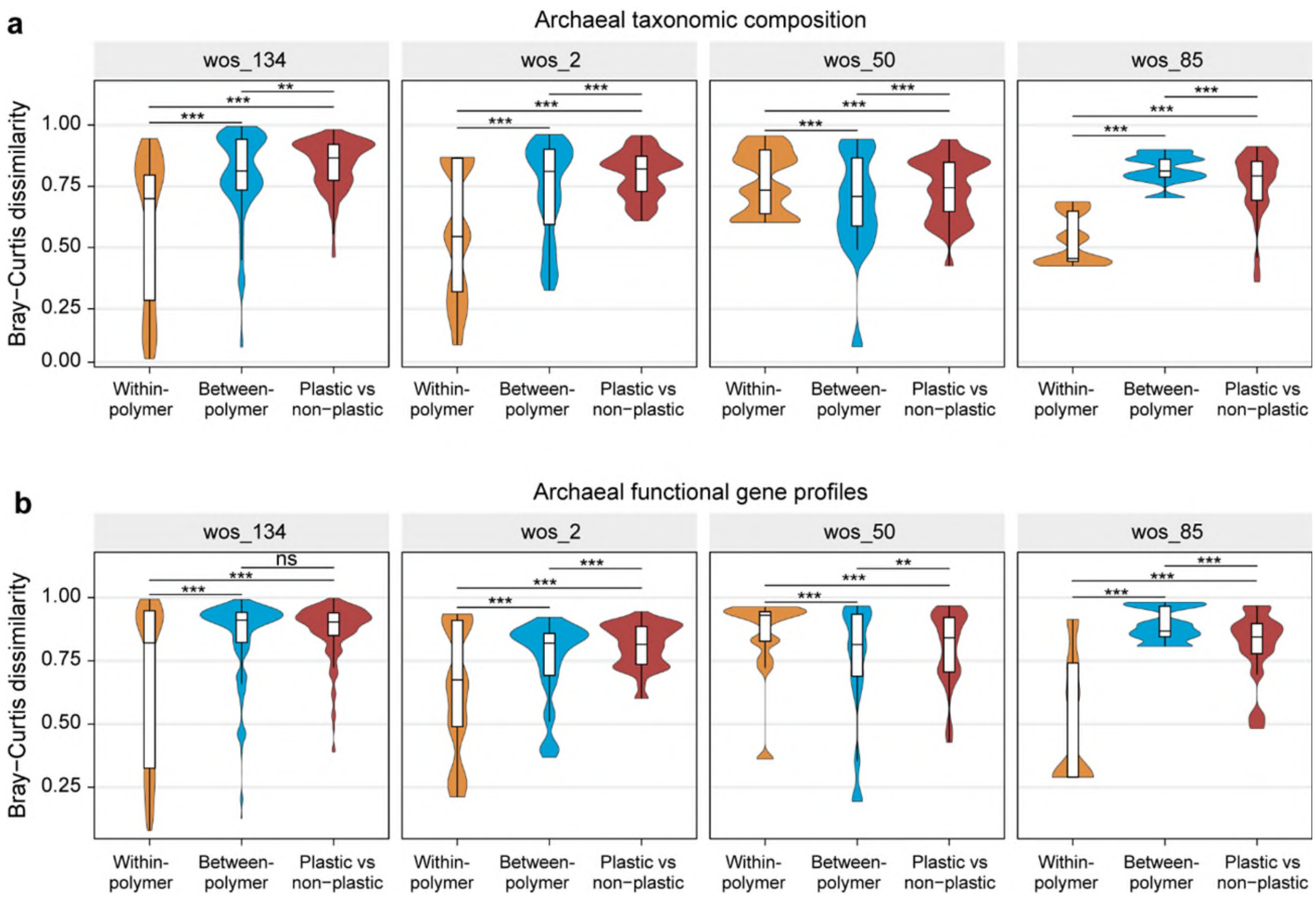

Fig. S11 Comparison of archaeal community dissimilarity across polymer types and substrate types. a, Taxonomic composition based on species-level GTDB annotation. **b,** Functional gene profiles based on combined archaeal carbon, nitrogen, phosphorus, sulfur, and methane cycling genes. In each panel, three categories of pairwise Bray–Curtis dissimilarity are shown across four independent studies: within-polymer, between-polymer, and plastic-versus-non-plastic. Each distribution comprises 999 bootstrap-sampled pairwise distances to ensure equal sample sizes across categories despite group imbalance. The horizontal line within each box indicates the median. Statistical significance was assessed using Wilcoxon rank-sum tests (*** $P < 0.001$, ** $P < 0.01$, ns = non-significant).

Minor comments:

Title: Consider using American spelling for consistency, e.g., "Unraveling" (one "l") instead of

“Unravelling”. The manuscript text predominantly uses American English, so the title might be adjusted to match, unless journal style dictates otherwise.

Response: Thank you for this helpful suggestion. We have revised the title to “Unraveling” in the revised manuscript to ensure consistency with American English usage.

Line 65: “the ‘island effect’ of the plastisphere habitat” – this metaphor is great, but the wording could be clarified. Perhaps “the isolated ‘island’ nature of the plastisphere habitat” or “the plastisphere’s ‘island effect’ (i.e., each plastic piece acting as an isolated microhabitat)”. A brief explanation of “island effect” would ensure all readers understand the concept.

Response: Thank you for this suggestion. Because this wording was speculative rather than directly supported by evidence generated in the present study, and in light of a related comment from Reviewer #4, we have removed this sentence from the Abstract.

Line 100: “effective, adoptive management strategies” – This seems to be a small typo: it should likely read “adaptive management strategies.” Adaptive management is a common term in environmental science, whereas “adoptive” is not usually used in this context.

Response: Thank you for catching this typo. We have corrected “adoptive” to “adaptive” in the revised manuscript.

Lines 126-133: Excellent that sampling was done across two regions and two seasons. To give the reader a better sense of the dataset up front, consider stating the total number of samples (plastisphere vs bulk water) here if possible. For example, “sampling was conducted in two seasons… yielding X plastic debris samples and X adjacent water samples in total.” This would complement the mention of diverse habitats and underline how robust the sampling effort was.

Response: Thanks for this suggestion. We have therefore added this information to the revised manuscript as “yielding a total of 94 sample pairs (plastisphere versus bulk water).”

Line 169: “Thaumarchaeota, Woesearchaeota, and Pacearchaeota were significantly depleted”. However, according to Fig. 1d, Thaumarchaeota showed no significant difference. Please verify whether this result was statistically significant.

Response: Thank you for this helpful reminder. We agree that the previous wording was not sufficiently precise. The *P* value for *Thaumarchaeota* was 0.087 and therefore did not reach statistical significance. To avoid ambiguity, we have revised the text as follows: "Euryarchaeota and Crenarchaeota were enriched in the plastisphere ($P < 0.01$), whereas Thaumarchaeota ($P = 0.087$), Woesearchaeota ($P < 0.05$), and Pacearchaeota ($P < 0.001$) were depleted." (Lines 182 – 184 in the main text)

Lines 175-177: "capacity to harbor archaeal taxa that are either non-native to the local environment or too rare in the surrounding water to be detected". To avoid any potential misinterpretation, the authors might clarify what "non-native to the local environment" entails. Does it imply that some archaea found on the plastics may have originated from distant regions or unusual sources (e.g., terrestrial runoff or deep sea)? If the data identified any surprising taxa on plastics that one wouldn't expect in coastal water, it would be great to mention an example. If not, perhaps rephrase to "taxa that are not observed in the surrounding water" (which covers both truly allochthonous and simply rare ones).

Response: Thank you for this helpful suggestion. We agree that the phrase "non-native to the local environment" could be over-interpreted, as our data do not allow us to distinguish confidently between truly allochthonous taxa and taxa that are simply present at very low abundance in the surrounding water. We have therefore revised the sentence to a more precise and neutral wording as follows:

Some ASVs were detected only in plastisphere samples and not in bulk water (Fig. 1e), highlighting the plastisphere's capacity to harbor archaeal taxa that are not observed in the surrounding water or occur there at abundances below detection. (Lines 186 – 189 in the main text)

Line 253: "Our data corroborates these trends from a coastal archaeal perspective." This should be "data corroborate" (plural verb agreement). Also, to improve the phrasing: maybe "Our data corroborate these trends from an archaeal perspective in coastal waters."

Response: Thank you for this helpful reminder. During the revision process, this sentence was removed from the manuscript in response to other comments, and the issue of verb agreement

is therefore no longer relevant in the revised version.

Lines 402-404: “the plastisphere’s functional composition thus appears less sensitive to environmental variation”. Perhaps use a term like “buffered” or “insulated” to convey the idea of reduced sensitivity. Additionally, line 419 mentions “Notably, several……to be enhanced under high levels of antibiotic pollution (Fig. 4e).” I think some text might be missing around “Notably, several…”. If it’s referring to several taxa or functions that only appear under high pollution, consider completing that thought.

Response: Thank you for these helpful suggestions. We have incorporated the term “buffering” into the revised text and now state: The plastisphere’s functional composition thus appears less sensitive to environmental variation, suggesting a relatively stable, and potentially functionally redundant, metabolic repertoire within plastisphere communities, as well as a possible buffering effect of the plastisphere microenvironment for microorganisms under harsh and fluctuating environmental conditions. (Lines 444 – 447 in the main text)

Regarding the text around line 419, we agree that the original wording was unclear. Our intention was not to suggest that these taxa or functions appear only under high-pollution conditions, but rather that they become more strongly enriched under higher levels of pollution. We have revised the relevant wording accordingly in the manuscript to make this point clearer.

Once again, I want to congratulate the authors on a novel and important study. These comments are mostly aimed at clarity and ensuring the manuscript’s messaging is as strong as the data. I am excited about the findings and enthusiastically recommend publication after revision. The insights into the coastal plastisphere archaeome open up new questions and directions for research, and this paper will likely become a foundational reference for the topic.

Response: We are deeply grateful for your support, encouragement, and thoughtful evaluation of our work. We greatly appreciate your positive comments on the novelty and importance of the study, and we are very pleased that you find the work promising and potentially foundational for this topic. Your feedback has been extremely helpful in improving the clarity and overall strength of the manuscript.

Reviewer #2 (Remarks to the Author):

I am for constructive and fair feedback and hope the authors can appreciate it as such. Altogether, the negligible relative abundance of archaea on plastic marine debris diminishes the scope and impact of this study, which I regard as being methodologically sound and thoughtful – inadvertently misleading though because the relative abundance data are not displayed and may very well verify that archaeal abundance is negligible.

Response: We thank the reviewer for this important concern. We appreciate your acknowledgment that the study is methodologically sound and thoughtful, and we have carefully addressed the concern regarding archaeal relative abundance.

We agree that reporting cross-domain relative abundance data is essential for properly contextualizing our findings. In the revised manuscript, we have therefore added a quantitative comparison of the relative abundances of Bacteria, Archaea, and Fungi across all samples based on shotgun metagenomic sequencing, with the detailed methods and results now provided in Supplementary Note S1. As expected, Bacteria dominated both seawater (~98.96%) and plastisphere (~98.66%) communities, whereas Archaea accounted for ~0.94% in seawater and ~1.03% in the plastisphere, and Fungi for ~0.10% and ~0.31%, respectively. These data are now cited in the Results section, where the low relative abundance of Archaea is explicitly acknowledged and placed into context. We have therefore been transparent throughout the revised manuscript about the quantitatively minor status of archaeal reads.

At the same time, we respectfully maintain that low relative abundance does not necessarily imply ecological negligibility. The concept of the “rare biosphere” is well established in microbial ecology, recognizing that low-abundance taxa can contribute disproportionately to key ecosystem functions and resilience. Marine Thaumarchaeota, for example, are globally important ammonia oxidizers despite constituting only a minor fraction of total prokaryotic communities. In addition, the relatively low archaeal proportions in shotgun metagenomes may partly reflect the still less comprehensive representation of archaeal genomes in current reference databases, a known systematic limitation that our GTDB-based custom database was specifically intended to reduce.

The purpose of this study is therefore not to argue that Archaea numerically rival Bacteria in the plastisphere, but rather to determine how this third domain of life assembles in this novel

anthropogenic habitat and what functional potential is associated with these communities. This archaeal perspective has remained largely absent from plastisphere research, and we believe that providing a dedicated archaeal-focused assessment represents a meaningful and timely contribution to both archaeal ecology and plastisphere science.

The revised related text is as follows:

Metagenomic read classification revealed that, across all samples, bacteria dominated the coastal marine microbiome, accounting for ~98–99% of the combined bacterial, archaeal, and fungal reads, whereas archaea and fungi each constituted only ~1% and ~0.2%, respectively (Supplementary Notes S1 and S2 and Figs. S2 and S3). The relatively low archaeal abundance may partly reflect underrepresentation of archaeal genomes in current reference databases, which can lead to underestimation in shotgun-based classifications. Nevertheless, low relative abundance does not necessarily imply low ecological relevance[11-13]. We therefore further examined whether plastisphere-associated archaea exhibit distinct taxonomic, assembly, and functional patterns. (Lines 138 – 145 in the main text)

Supplementary Note S1 Relative abundance of archaea, bacteria, and fungi in the plastisphere

To contextualize the ecological significance of archaea within the broader microbial community, we quantified the relative abundances of the three domains—archaea, bacteria, and fungi—across all plastisphere and seawater samples using a dual-database Kraken2 classification strategy. For bacterial and archaeal classification, a custom database was constructed using GTDB release 207 representative genomes, comprising 62,291 bacterial and 3,412 archaeal species-representative genomes selected from 311,480 bacterial and 6,062 archaeal total genomes. For fungal classification, the Kraken2 PlusPF database, which incorporates RefSeq fungal reference genomes, was used. All metagenomic reads were classified independently against both databases in paired-end mode, and reads assigned to non-target groups were discarded.

Domain-level relative abundances were calculated as follows. For each sample, bacterial and archaeal read counts were extracted from the GTDB-based Kraken2 report at the domain rank, whereas fungal read counts were extracted from the PlusPF-based report at the kingdom rank. To account for differences in total read counts between the two databases, the proportion of each

domain relative to the total reads in its respective database was first calculated, and the three proportions were then normalized to sum to 100%.

As expected, bacteria dominated both seawater and plastisphere communities, accounting for 98.96% (96.32%–99.91%) and 98.66% (89.21%–99.94%) of the combined three-domain reads, respectively (Supplementary Fig. S2). Archaea and fungi each represented minor but consistent fractions of the total community (Supplementary Fig. S2). Archaea comprised 0.94% (0.07%–3.61%) of reads in bulk water and 1.03% (0.03%–10.08%) in plastisphere communities, whereas fungi accounted for 0.10% (0.02%–0.56%) and 0.31% (0.02%–2.70%), respectively (Supplementary Fig. S2). The relatively low archaeal read proportions may also partly reflect the still less complete representation of archaeal genomes in current reference databases compared with bacteria, which can lead to systematic underestimation of archaeal reads in shotgun-based classification. The use of a GTDB-based database, which provides substantially broader archaeal genome coverage than standard NCBI RefSeq-based Kraken2 databases (3,412 versus approximately 800–1,000 archaeal representative genomes), was intended to mitigate this bias and improve archaeal abundance estimates.

These data confirm that, although archaea constitute a numerically minor fraction of the total microbial community, they are consistently present across all samples and habitats. In microbial ecology, low relative abundance does not necessarily imply low ecological importance. For example, marine ammonia-oxidizing Thaumarchaeota drive a substantial fraction of global oceanic nitrification despite comprising only a minor fraction of total prokaryotic cells[14, 15], and the “rare biosphere” concept recognizes that low-abundance taxa can maintain important ecosystem functions and resilience[12, 13, 16, 17]. The domain-level abundance data are therefore provided here as contextual background rather than as a direct measure of ecological importance. The primary analyses in the main text, including archaeal community structure, diversity, assembly, and functional potential, are based on archaeal-targeted amplicon sequencing and archaeal-specific metagenomic analyses designed to resolve archaeal ecology at appropriate resolution.

Supplementary Note S2 Recovery of archaeal reads from bacterial-targeted 16S amplicon data

To further illustrate that the limited representation of archaea in the plastisphere literature may partly reflect methodological constraints rather than true ecological absence, we re-examined a previously published dataset from our group[18] in which 16S rRNA gene amplicon sequencing had

been performed using the 515F/806R primer pair with a bacterial focus. When archaeal reads were retained rather than discarded during taxonomic assignment, archaeal sequences were recovered at detectable proportions across all samples.

Notably, the plastisphere group showed a higher archaeal proportion (~3.7%) than the seawater group (Supplementary Fig. S3), consistent with the view that plastic surfaces can selectively concentrate archaeal taxa. These results are presented here only as a methodological illustration and do not constitute new findings of the present study. Instead, they support the interpretation that the apparent scarcity of archaea in many 16S-based plastisphere surveys may partly reflect under-detection arising from primer bias and downstream analytical choices, rather than true ecological absence. By contrast, archaeal reads were not recovered from the bacterial-targeted 341F/806R amplicon dataset generated in the present study, which is also consistent with the known primer-dependent variation in archaeal coverage.

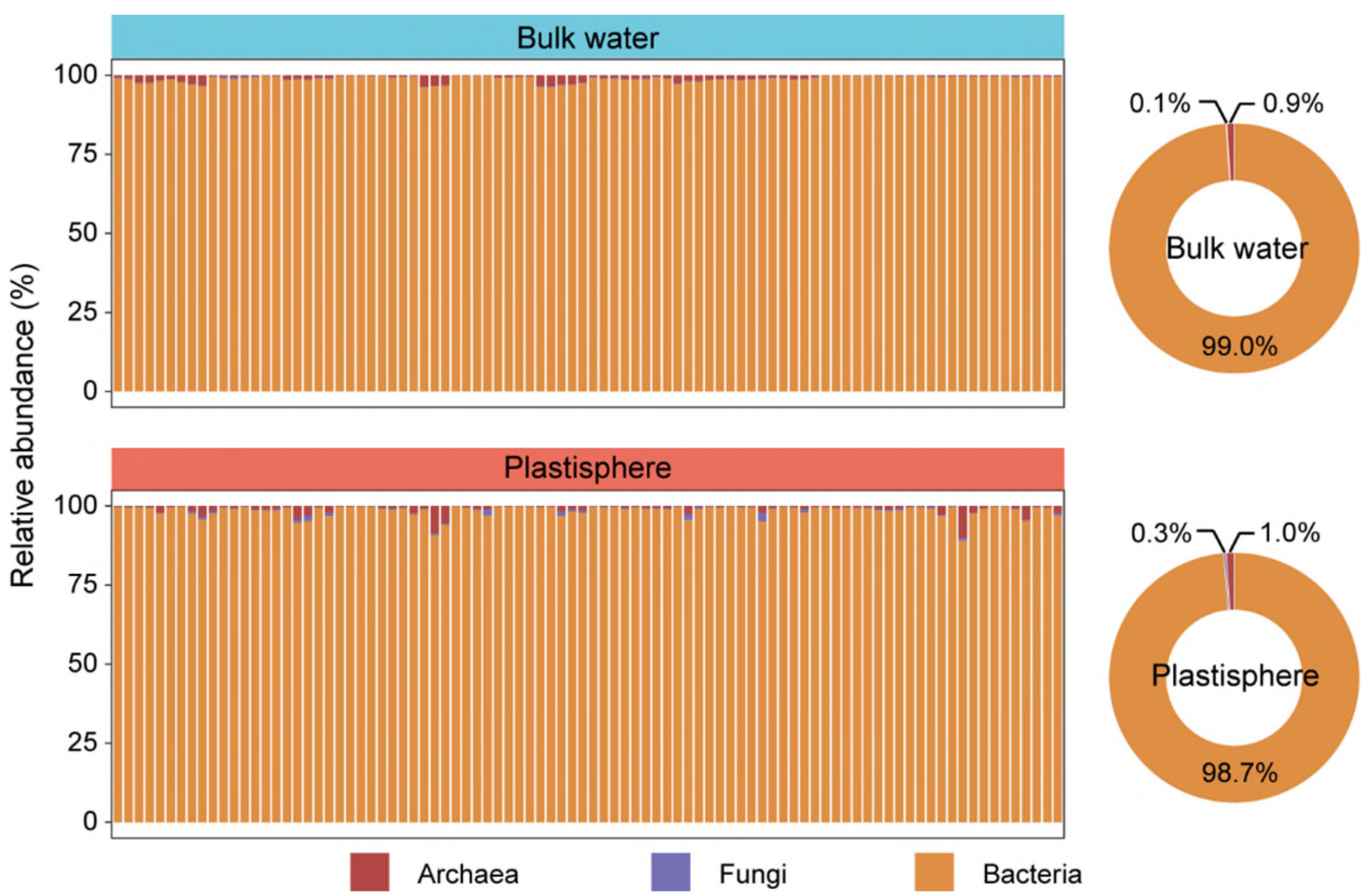

Fig. S2 Domain-level relative abundances of archaea, bacteria, and fungi in plastisphere and bulk-water communities. Stacked bar plots and pie charts show the relative proportions of archaea, bacteria, and fungi in plastisphere and seawater samples. Details of the classification strategy and normalization procedure are provided in Supplementary Note S1. *n* = 82 per group.

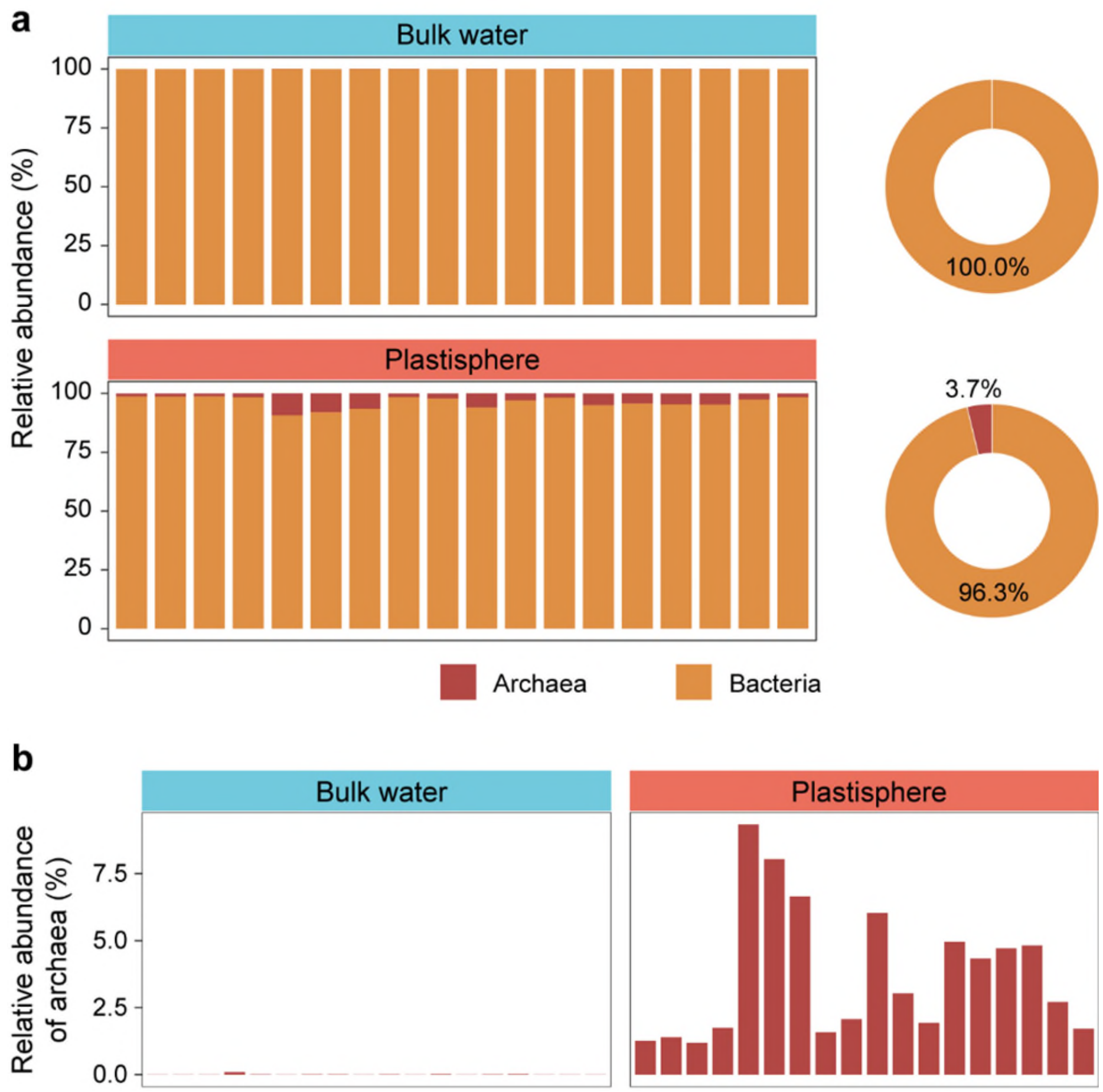

Fig. S3 Recovery of archaeal reads from a bacterial-targeted 16S amplicon dataset. a, Stacked bar plots and pie charts showing the relative proportions of archaea and bacteria recovered from a previously published 16S amplicon dataset generated with the 515F/806R primer pair[18] when archaeal reads were retained during taxonomic assignment. **b,** Relative abundance of archaeal reads in plastisphere and seawater samples from the same dataset. These data are provided as a methodological illustration showing that archaeal sequences can be recovered from bacterial-targeted amplicon surveys at detectable levels, particularly in plastisphere communities, and do not represent new results of the present study. *n* = 18 per group.

The manuscript investigates archaeal communities colonizing plastic marine debris and those subsisting in surrounding seawaters. The authors implemented a granular taxonomic annotation scheme (i.e., taxonomy based on 16S and whole-genome-shotgun sequencing) followed by an analysis of functional metabolic potential, including biochemically proven metabolic pathways. The manuscript is refreshingly well-written and the bioinformatic approach is mainly sound

(although I have some questions regarding assembly, DIAMOND vs. BLAST for annotating "biogeochemical cycling genes", and whether or not metagenome-assembled genomes could be binned.) Furthermore, the authors have taken particular care to demonstrate that collected particles were indeed plastic via O-PTIR spectroscopy, which I appreciate.

Response: Thank you very much for your positive evaluation of our manuscript, including both the writing and the methodology. We are grateful for your recognition of the care taken in the study design, bioinformatic analysis, and verification of plastic identity.

Following your comments, we further performed genome-resolved metagenomic binning and recovered 23 archaeal metagenome-assembled genomes (MAGs), of which 5 were derived from plastisphere samples. Notably, when binning was performed using plastisphere samples alone, the same five plastisphere-derived archaeal MAGs were still recovered. These MAGs and their functional annotations are now described in the revised Methods section and presented in the Supplementary Information. We have also added the relevant results and associated methods to the revised manuscript as follows:

Genome-resolved metagenomic analysis further supported this pattern: among 23 recovered archaeal metagenome-assembled genome (MAGs), all five plastisphere-derived MAGs were affiliated with Nitrososphaerales, whereas Poseidoniales MAGs were recovered exclusively from bulk water (Supplementary Note S4 and Figs. S8 and S9). (Lines 193 – 197 in the main text)

Our genome-resolved analyses further supported this pattern: Nitrososphaerales MAGs were enriched in the plastisphere and carried key nitrogen-cycling genes, including complete *amoABC*, *ureABC*, and *nirK*, consistent with the potential for ammonia oxidation and related nitrogen transformations in plastisphere biofilms (Supplementary Note S4 and Figs. S8 and S9). (Lines 273 – 276 in the main text)

Notably, *Nitrososphaera* emerged as the single most important biomarker, consistent with the genome-resolved metagenomic results showing enrichment of Nitrososphaerales MAGs in the plastisphere (Supplementary Figs. S8 and S9). Together, these results indicate that ammonia-oxidizing Nitrososphaerales-related archaea are a prominent and recurrent signature of the plastisphere archaeome. (in Supplementary Note S3)

Supplementary Note S4 Recovery and characterization of archaeal metagenome-assembled genomes

Using genome-resolved metagenomic binning, we reconstructed 23 non-redundant archaeal metagenome-assembled genomes (MAGs) that met at least medium-quality draft criteria (completeness ≥50%, contamination ≤10%; Supplementary Fig. S8)[19]. Taxonomic classification within the GTDB framework indicated two dominant archaeal lineages: Nitrososphaerales (*n* = 11; phylum Thermoproteota, class Nitrososphaeria) and Poseidoniales (*n* = 12; phylum Thermoplasmatota, class Poseidoniia). Notably, all five plastisphere-derived MAGs were affiliated with Nitrososphaerales, whereas all Poseidoniales MAGs were recovered exclusively from bulk-water metagenomes (Supplementary Fig. S8). The plastisphere signal was robust to dereplication strategy, as dereplication restricted to plastisphere-derived bins recovered the same five Nitrososphaerales MAGs. Despite comparable sequencing depth across habitats, fewer archaeal MAGs were recovered from plastisphere samples, consistent with the greater complexity and strain heterogeneity expected in surface-attached biofilms, which can reduce assembly contiguity and genome recovery[20]. This pattern is also consistent with the higher archaeal taxonomic diversity observed in the amplicon data (Fig. 1 in the main text).

Because Nitrososphaerales/Nitrososphaeria comprise ammonia-oxidizing archaea (AOA), typically characterized by genes encoding ammonia monooxygenase[21], we evaluated the nitrogen-cycling potential of recovered MAGs by annotating predicted proteins against NCycDB[22]. Among the five plastisphere-derived Nitrososphaerales MAGs, two encoded the complete ammonia monooxygenase gene set (*amoA*, *amoB*, *amoC*), supporting genomic potential for archaeal ammonia oxidation (Supplementary Fig. S8). One of these MAGs additionally contained a complete urease operon (*ureA*, *ureB*, *ureC*) (Supplementary Fig. S8), suggesting the potential to hydrolyze urea and thereby generate ammonia for nitrification[23]. Given that urea is widely available in marine systems and can support AOA growth and activity[24], this genomic configuration is consistent with the possibility that plastisphere-associated AOA exploit dissolved organic nitrogen substrates within the biofilm microenvironment.

We further detected *nirK* (copper-containing nitrite reductase) in most plastisphere Nitrososphaerales MAGs (Supplementary Fig. S8). In AOA, *nirK* has been shown to generate nitric oxide (NO) from nitrite and/or hydroxylamine-derived intermediates and is considered central to archaeal nitrogen metabolism[25]. NO-related reactions in AOA have also been linked to nitrous oxide (N_2O) formation under low-oxygen conditions[26]. Although gene presence alone does

not demonstrate in situ activity, these marker genes suggest that plastisphere-associated AOA may contribute to localized nitrification and NO/N_2O-related transformations within redox-stratified plastisphere biofilms.

In contrast, bulk-water Poseidoniales MAGs lacked *amo* genes (Supplementary Fig. S8), consistent with genome-resolved studies describing Poseidoniales (Marine Group II archaea) as predominantly aerobic (photo)heterotrophs involved in dissolved and particulate organic matter turnover, with additional lineage-specific traits including transport and degradation of organic substrates and, in some taxa, nitrate-reduction capacity[27].

Finally, metagenomic read recruitment to MAG-predicted genes showed that Nitrososphaerales MAGs were significantly more abundant in plastisphere samples than in bulk water, whereas Poseidoniales MAGs showed the opposite pattern (Wilcoxon rank-sum tests, $P < 0.001$; Supplementary Fig. S9).

Collectively, the selective recovery and enrichment of AOA-affiliated MAGs in the plastisphere, together with their encoded potential for ammonia oxidation, urea utilization, and NO-related nitrite reduction, provide genome-resolved support for our gene-centric inference that the plastisphere may act as a localized hotspot for archaeal nitrogen transformations in coastal marine ecosystems.

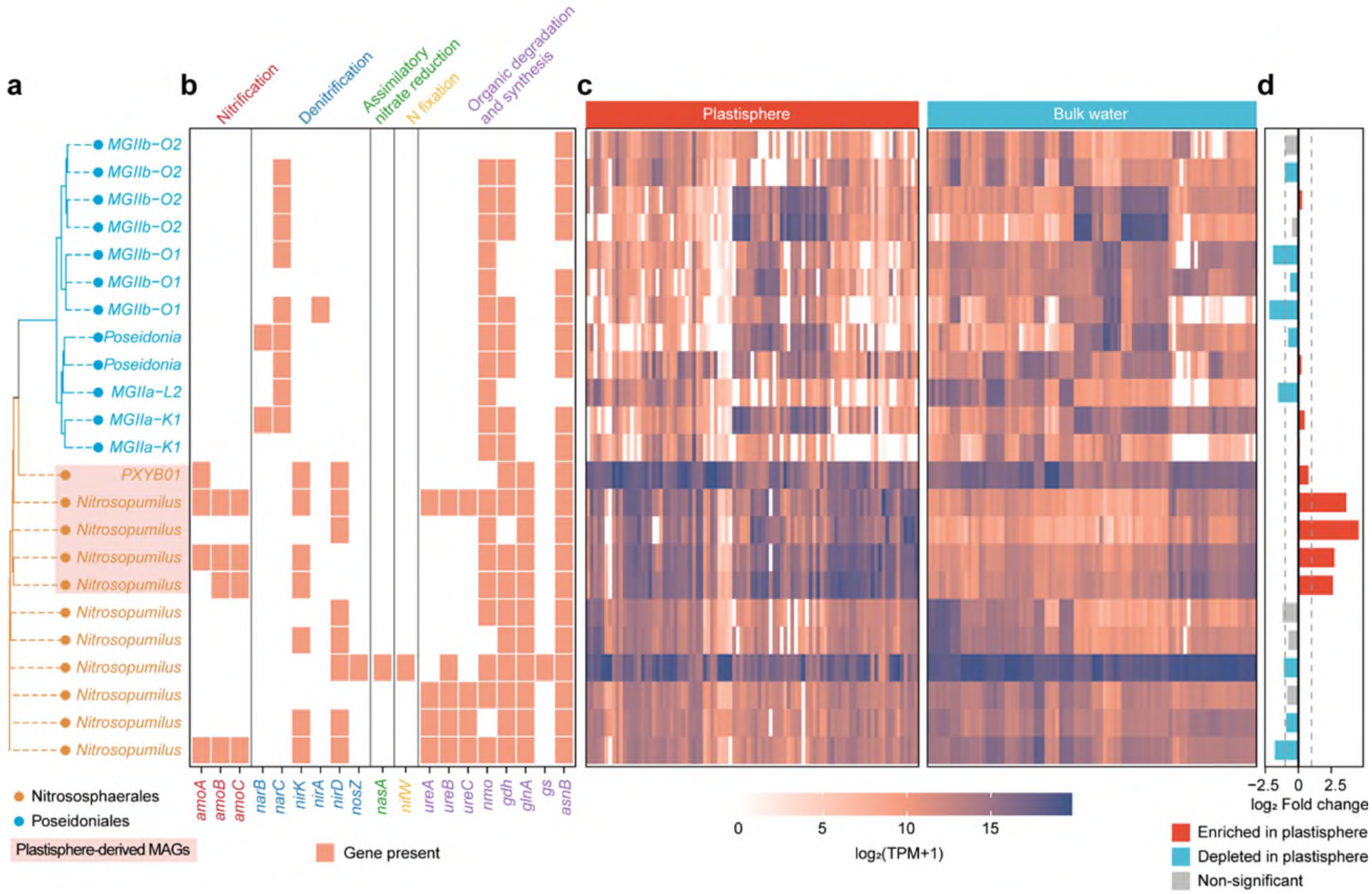

Fig. S8 Genome-resolved characterization of archaeal MAGs recovered from plastisphere and bulk-water communities. a, Maximum-likelihood phylogenetic tree of 23 archaeal MAGs inferred from ar53 marker genes. Tips are coloured by taxonomic order (orange, Nitrososphaerales; blue, Poseidoniales); plastisphere-derived MAGs are highlighted with a pink background. **b,** Presence of nitrogen-cycling genes across all 23 MAGs, grouped by functional process according to NCycDB classification. **c,** Heat map of MAG relative abundance (log_2-transformed TPM) across plastisphere and bulk water samples. **d,** Differential abundance of each MAG between plastisphere and bulk water. Red and blue bars indicate significant enrichment in plastisphere and bulk water, respectively; grey bars indicate no significant difference (Wilcoxon rank-sum test, $P < 0.05$).

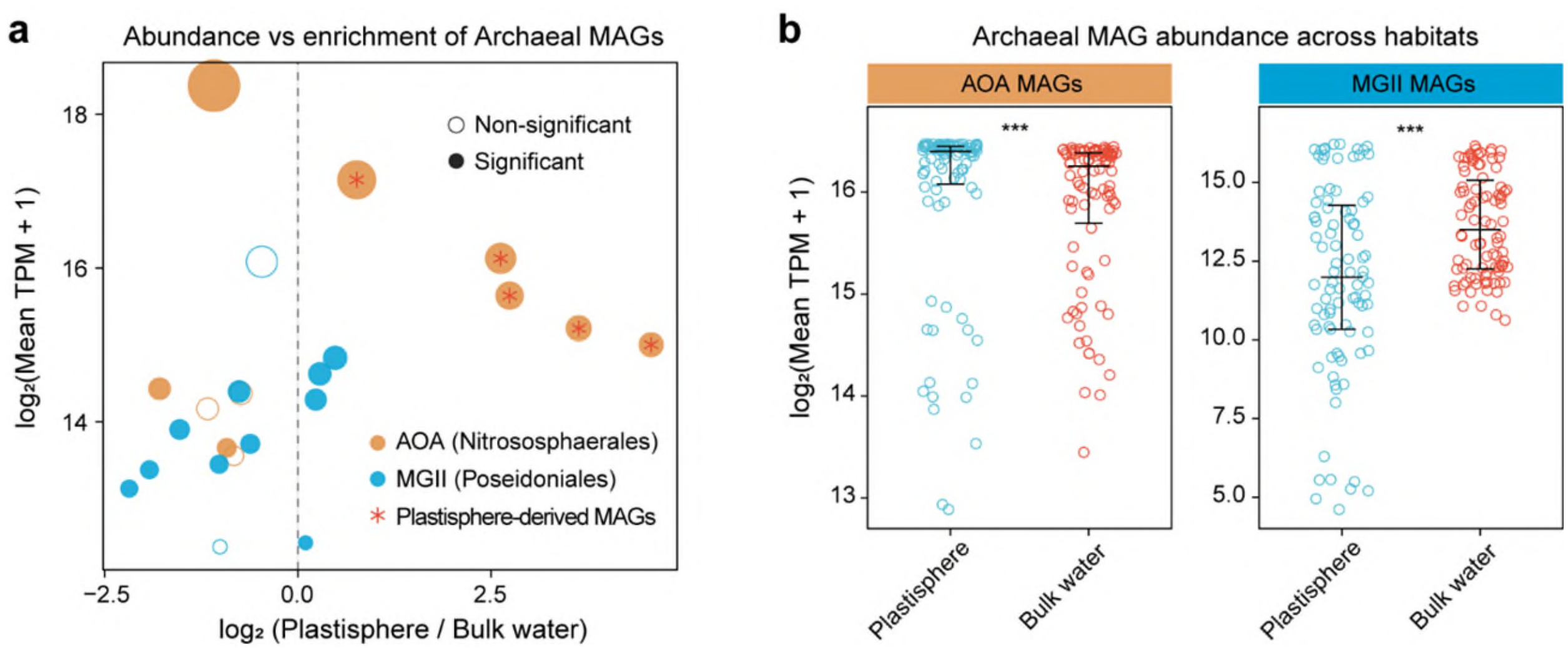

Fig. S9 Differential abundance of archaeal MAGs between plastisphere and bulk water. a, Bubble plot showing the relationship between differential abundance and mean abundance for each MAG. Bubble size represents mean total TPM, and colour indicates taxonomic affiliation (orange, Nitrososphaerales; blue, Poseidoniales). Plastisphere-derived MAGs are marked with asterisks. Filled and open symbols indicate statistically significant and non-significant differences, respectively (Wilcoxon rank-sum test, $P < 0.05$). **b,** Mean abundance of Nitrososphaerales and Poseidoniales MAGs in plastisphere and bulk-water samples. Significance was assessed using Wilcoxon rank-sum tests ($n = 82$ per group; *** $P < 0.001$).

The authors have astutely pointed out that archaea make important biogeochemical contributions in various systems and that coastal habitats are laden with plastic pollution, which microbes respond to writ large – a promising area of inquiry to understand how microbes adapt to plastic surfaces. The former statements need to be contextualized though because of archaea's low abundance on plastic – this is being oversold (discussed more below.) Archaeal community assembly (and frankly a description of archaea in the plastisphere) is poorly understood and not well-represented in the literature. This may be in part because of stochastic influences on archaea, as the authors empirically demonstrate herein. It may also be because archaea are not as effective colonizers of surfaces and are generally more specialized for a distinct niche. The lack of data on archaea in the plastisphere may also be directly related to archaea's low relative abundance in the plastisphere (I believe this is likely the main reason why there are few studies on archaea in the plastisphere.) In other words, most studies of the plastisphere, which predominantly employ 16S sequencing, do not show hardly any presence of archaea. There are caveats to that generalization, but our current body of knowledge suggests that archaea are a negligible component of epiplastic communities, both early and mature biofilms.

Response: We thank the reviewer for this thoughtful perspective. We agree that archaea generally occur at much lower relative abundance than bacteria in most environmental compartments, and we have revised the manuscript throughout to avoid overstating either their abundance or their ecological impact. At the same time, we respectfully suggest that the limited representation of archaea in the plastisphere literature should not be attributed solely to ecological insignificance. Although archaeal research is a younger field than bacterial and fungal research, archaea are nevertheless recognized as an ecologically and evolutionarily important domain of life, often contributing disproportionately to key processes such as elemental cycling, particularly in specialized or understudied environments.

We also suggest that the relative scarcity of plastisphere archaeal studies reflects, at least in part, methodological factors. In many 16S amplicon-based plastisphere studies, archaeal sequences may in fact be generated, but because the studies are typically designed around bacterial-focused primers and analytical pipelines, archaeal reads are often not retained or examined in detail. To illustrate this point, we re-examined a previous dataset from our own earlier study[18] generated with the 515F/806R primer pair and originally analyzed with a bacterial focus. When reads not assigned as bacteria were retained rather than discarded, archaeal sequences were recovered at detectable levels, including a proportion of ~3.7% in plastisphere samples, which was notably higher than in seawater (Supplementary Note S2 and Fig. S3). By contrast, archaeal reads were not recovered in the 341F/806R amplicon dataset generated in the present study, which is consistent with the stronger bacterial bias of that primer set. We present this comparison only as a methodological illustration in the response, rather than as a new result of the present study. Together, these observations suggest that the apparent scarcity of archaea in many plastisphere surveys may partly reflect under-detection, in addition to their low relative abundance.

Accordingly, our aim in this study was not to argue that archaea are dominant members of the plastisphere, but rather to resolve plastisphere archaeal diversity specifically using archaeal-targeted primers and to assess how this third domain of life assembles in the plastisphere and what functional potential and ecological implications may be associated with it. This archaeal perspective represents a previously overlooked dimension of plastisphere research and of plastic pollution impacts more broadly.

More generally, lower abundance than bacteria does not necessarily imply lesser importance. As

we now clarify in the revised main text and Supplementary Note S1, archaea can play disproportionate roles in biogeochemical processes, and in many systems important ecosystem functions are governed by only a small fraction of community members.

The revised related parts are as follows:

Metagenomic read classification revealed that, across all samples, bacteria dominated the coastal marine microbiome, accounting for ~98–99% of the combined bacterial, archaeal, and fungal reads, whereas archaea and fungi each constituted only ~1% and ~0.2%, respectively (Supplementary Notes S1 and S2 and Figs. S2 and S3). The relatively low archaeal abundance may partly reflect underrepresentation of archaeal genomes in current reference databases, which can lead to underestimation in shotgun-based classifications. Nevertheless, low relative abundance does not necessarily imply low ecological relevance[11-13]. We therefore further examined whether plastisphere-associated archaea exhibit distinct taxonomic, assembly, and functional patterns. (Lines 138 – 145 in the main text)

Supplementary Note S1 Relative abundance of archaea, bacteria, and fungi in the plastisphere

To contextualize the ecological significance of archaea within the broader microbial community, we quantified the relative abundances of the three domains—archaea, bacteria, and fungi—across all plastisphere and seawater samples using a dual-database Kraken2 classification strategy. For bacterial and archaeal classification, a custom database was constructed using GTDB release 207 representative genomes, comprising 62,291 bacterial and 3,412 archaeal species-representative genomes selected from 311,480 bacterial and 6,062 archaeal total genomes. For fungal classification, the Kraken2 PlusPF database, which incorporates RefSeq fungal reference genomes, was used. All metagenomic reads were classified independently against both databases in paired-end mode, and reads assigned to non-target groups were discarded.

Domain-level relative abundances were calculated as follows. For each sample, bacterial and archaeal read counts were extracted from the GTDB-based Kraken2 report at the domain rank, whereas fungal read counts were extracted from the PlusPF-based report at the kingdom rank. To account for differences in total read counts between the two databases, the proportion of each

domain relative to the total reads in its respective database was first calculated, and the three proportions were then normalized to sum to 100%.

As expected, bacteria dominated both seawater and plastisphere communities, accounting for 98.96% (96.32%–99.91%) and 98.66% (89.21%–99.94%) of the combined three-domain reads, respectively (Supplementary Fig. S2). Archaea and fungi each represented minor but consistent fractions of the total community (Supplementary Fig. S2). Archaea comprised 0.94% (0.07%–3.61%) of reads in bulk water and 1.03% (0.03%–10.08%) in plastisphere communities, whereas fungi accounted for 0.10% (0.02%–0.56%) and 0.31% (0.02%–2.70%), respectively (Supplementary Fig. S2). The relatively low archaeal read proportions may also partly reflect the still less complete representation of archaeal genomes in current reference databases compared with bacteria, which can lead to systematic underestimation of archaeal reads in shotgun-based classification. The use of a GTDB-based database, which provides substantially broader archaeal genome coverage than standard NCBI RefSeq-based Kraken2 databases (3,412 versus approximately 800–1,000 archaeal representative genomes), was intended to mitigate this bias and improve archaeal abundance estimates.

These data confirm that, although archaea constitute a numerically minor fraction of the total microbial community, they are consistently present across all samples and habitats. In microbial ecology, low relative abundance does not necessarily imply low ecological importance. For example, marine ammonia-oxidizing Thaumarchaeota drive a substantial fraction of global oceanic nitrification despite comprising only a minor fraction of total prokaryotic cells[14, 15], and the “rare biosphere” concept recognizes that low-abundance taxa can maintain important ecosystem functions and resilience[12, 13, 16, 17]. The domain-level abundance data are therefore provided here as contextual background rather than as a direct measure of ecological importance. The primary analyses in the main text, including archaeal community structure, diversity, assembly, and functional potential, are based on archaeal-targeted amplicon sequencing and archaeal-specific metagenomic analyses designed to resolve archaeal ecology at appropriate resolution.

Supplementary Note S2 Recovery of archaeal reads from bacterial-targeted 16S amplicon data

To further illustrate that the limited representation of archaea in the plastisphere literature may partly reflect methodological constraints rather than true ecological absence, we re-examined a previously published dataset from our group[18] in which 16S rRNA gene amplicon sequencing had

been performed using the 515F/806R primer pair with a bacterial focus. When archaeal reads were retained rather than discarded during taxonomic assignment, archaeal sequences were recovered at detectable proportions across all samples.

Notably, the plastisphere group showed a higher archaeal proportion (~3.7%) than the seawater group (Supplementary Fig. S3), consistent with the view that plastic surfaces can selectively concentrate archaeal taxa. These results are presented here only as a methodological illustration and do not constitute new findings of the present study. Instead, they support the interpretation that the apparent scarcity of archaea in many 16S-based plastisphere surveys may partly reflect under-detection arising from primer bias and downstream analytical choices, rather than true ecological absence. By contrast, archaeal reads were not recovered from the bacterial-targeted 341F/806R amplicon dataset generated in the present study, which is also consistent with the known primer-dependent variation in archaeal coverage.

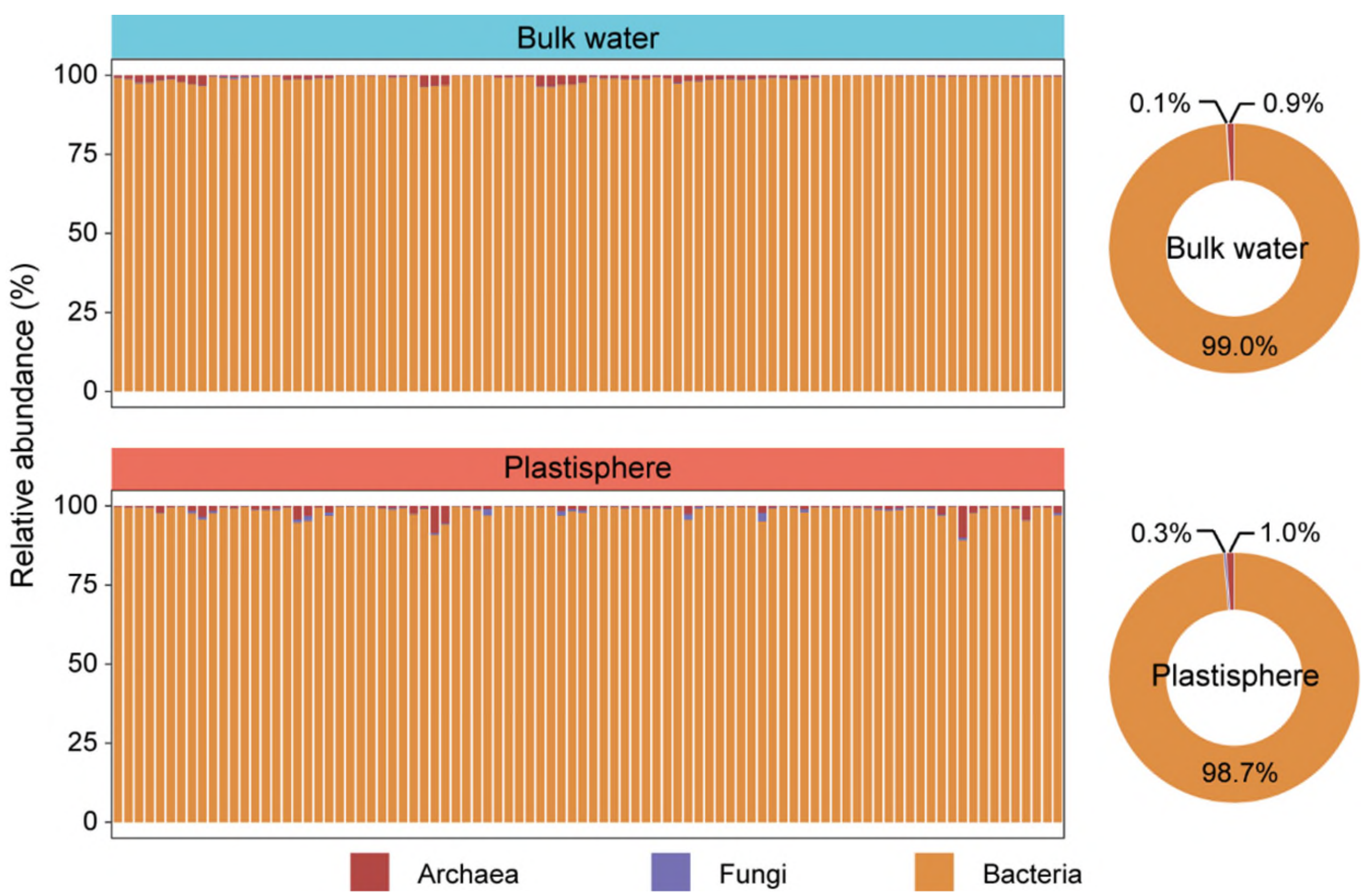

Fig. S2 Domain-level relative abundances of archaea, bacteria, and fungi in plastisphere and bulk-water communities. Stacked bar plots and pie charts show the relative proportions of archaea, bacteria, and fungi in plastisphere and seawater samples. Details of the classification strategy and normalization procedure are provided in Supplementary Note S1. n = 82 per group.

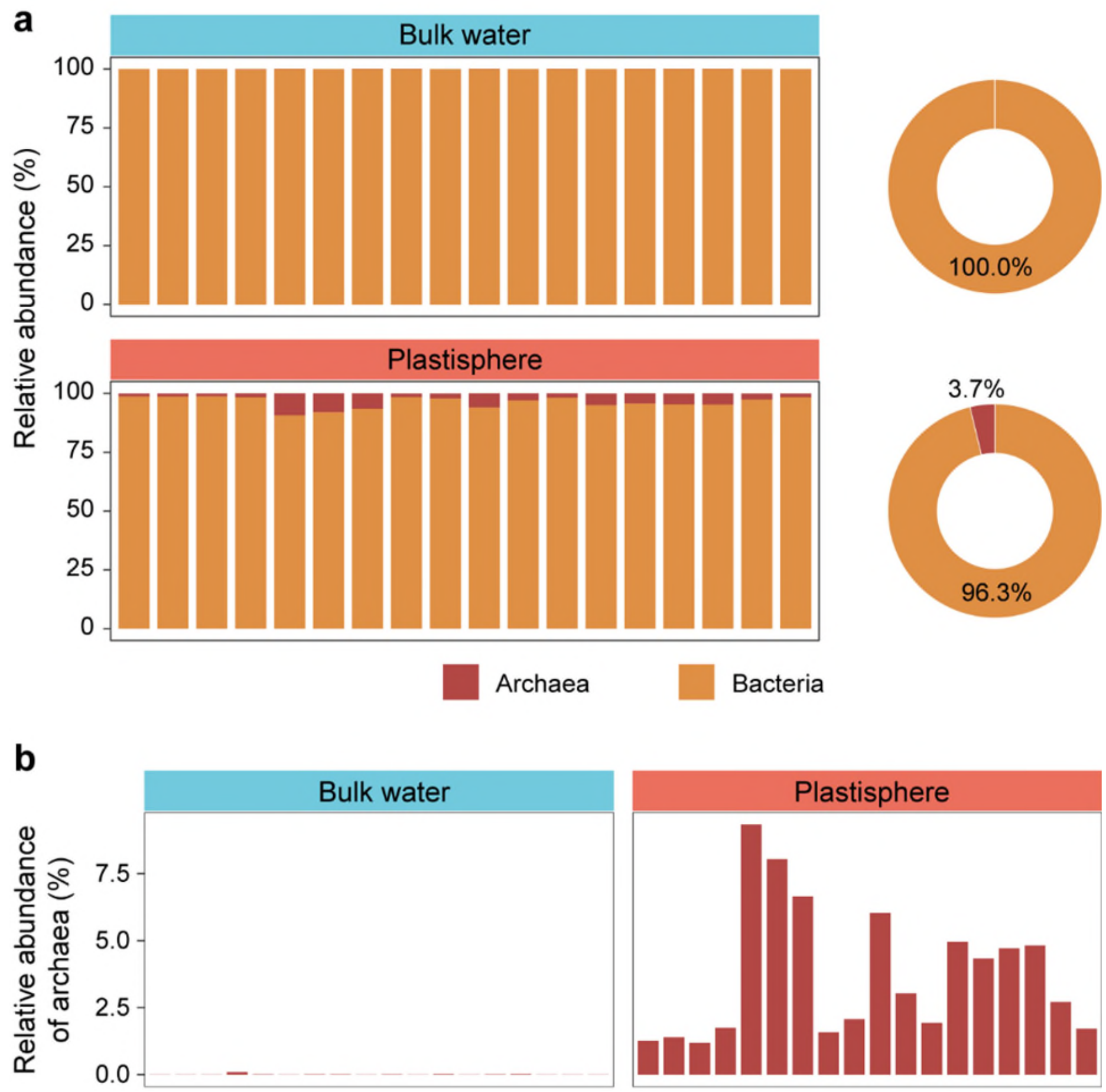

Fig. S3 Recovery of archaeal reads from a bacterial-targeted 16S amplicon dataset. a, Stacked bar plots and pie charts showing the relative proportions of archaea and bacteria recovered from a previously published 16S amplicon dataset generated with the 515F/806R primer pair[18] when archaeal reads were retained during taxonomic assignment. **b,** Relative abundance of archaeal reads in plastisphere and seawater samples from the same dataset. These data are provided as a methodological illustration showing that archaeal sequences can be recovered from bacterial-targeted amplicon surveys at detectable levels, particularly in plastisphere communities, and do not represent new results of the present study. *n* = 18 per group.

With regards to the present study, for 16S sequencing (and recognizing that microbes have different copy numbers of the 16S, 'preferential' amplification, etc), I did not see a taxonomic comparison of the relative abundance of archaea and bacteria (Figure 3 appears to have relative abundance data, but I suspect it can be interpreted as Thaumarchaeota are >60% of the relative

abundance of all archaea in this study.) For WGS data, I did not see a taxonomic comparison of the relative abundance of prokaryotes and eukaryotes, etc. Do archaea exhibit a higher-than-expected relative abundance in this study (compared to the other domains?) If so, these data need to be front and center to demonstrate what proportion of the community we are looking at. I suspect that we are looking at <0.5-1% of the total community abundance (probably even less, but I don't want to make the assumption – I would like the authors to communicate that should this manuscript proceed to a revision.) This is not to say that archaea do not play some role in microbial community assembly or have a biogeochemical influence – they just exist in such small relative proportions compared to bacteria, for example, and consequently have a lesser influence.

Response: Thank you for this important comment. As noted above, we agree that cross-domain relative abundance data are essential for properly contextualizing our findings. In the revised manuscript, we have therefore added a quantitative comparison of the relative abundances of Bacteria, Archaea, and Fungi across all samples based on shotgun metagenomic sequencing, with the detailed methods and results now provided in Supplementary Notes S1 and S2. These data show that Bacteria dominated both seawater and plastisphere communities, whereas Archaea accounted for ~0.94% in seawater and ~1.03% in the plastisphere, with Fungi representing ~0.10% and ~0.31%, respectively. These results are now cited in the Results and discussion section, and we have been explicit throughout the revised manuscript that archaea represent a quantitatively minor component of the total microbial community.

For the 16S amplicon data, cross-domain abundance comparison is not appropriate because the archaeal community analyses in this study were based on archaeal-targeted primers rather than universal primers. Our objective was therefore not to estimate the relative abundance of Archaea versus other domains from the amplicon data, but to resolve archaeal community structure specifically. More broadly, while we agree that archaea are numerically minor relative to bacteria, we do not consider low relative abundance alone to imply ecological negligibility. The purpose of this study is not to argue that Archaea numerically rival Bacteria in the plastisphere, but rather to characterize how this third domain of life assembles in this novel anthropogenic habitat and what functional potential is associated with these communities.

L54-L57: Yes, coastal marine ecosystems are of high ecological and social importance, and archaea are found there, but what is their abundance in epiplastic communities in early and mature stages? I recognize that the authors captured free-drifting plastics rather than incubating

polymers.

Response: We thank the reviewer for raising this important point. Our study was based on free-drifting plastic debris collected directly from the field, and therefore likely included particles spanning different degrees of weathering and biofilm development, rather than polymers incubated under defined early or mature colonization stages. This field-based design was chosen deliberately to capture authentic in situ plastisphere patterns under real environmental conditions. We agree that a systematic comparison of archaeal abundance and community dynamics across biofilm developmental stages would be highly valuable, but this question is beyond the scope of the present study and will require dedicated stage-resolved sampling or incubation experiments. We have therefore acknowledged this as an important direction for future work in the revised Conclusions and outlook section, where we now note that future studies should investigate plastisphere succession and other ecological mechanisms underlying archaeal community assembly. The revised part in the Conclusions and outlook section is as follows:

Nevertheless, because our analyses are based on taxonomic composition and metagenomic functional inference, direct links to viability, in situ activity, and realized biogeochemical rates remain to be established. In addition, the ecological implications highlighted here are based on relative comparisons rather than direct measurements of impact magnitude. For example, we still do not know to what extent plastic pollution contributes to microbial redistribution and invasion in the ocean, nor what magnitude of greenhouse-gas-related effects may be associated with plastisphere archaea. Further research is needed to directly link these microbial shifts to real-world ecological and health outcomes of plastic pollution, and to quantitatively assess the full risks of the plastisphere. Addressing these questions will require cross-disciplinary collaboration among researchers in environmental microbiology, environmental impact assessment, public health, and data science. Moreover, our study only considered surface waters and significant knowledge gaps remain regarding plastisphere dynamics throughout the water column and deep ocean ecosystems. Future work should also investigate the ecological mechanisms underlying plastisphere archaeal community assembly, including the roles of biofilm succession, substrate heterogeneity, and dispersal dynamics, to advance predictive understanding of how plastic pollution reshapes microbial diversity and function in marine ecosystems. (Lines 519 – 534)

L60-L63: That is cool that some pathways were enriched on plastic compared to seawater, but suggesting that it could alter nutrient cycles just with archaea is a real stretch.

Response: Thank you for this comment. We agree that the original wording could be interpreted too strongly. In the revised manuscript, we have therefore added "potentially" to make the statement more appropriately cautious and to avoid overstatement.

L72-L133: This is a terrific, succinct discussion.

Response: Thank you very much for this positive comment. We greatly appreciate your recognition of the writing and discussion in the manuscript.

L137-L138: OK, but how does the relative abundance compare across plastic samples and in seawater? I would not compare 16S vs. WGS relative abundance data, but there needs to be data for both 16S and WGS relative abundances calculated separately.

Response: Thank you for this important comment. For the 16S amplicon data, cross-domain abundance comparison is not appropriate because we used archaeal-targeted primers (Arch519F/Arch915R), and the resulting dataset is therefore inherently restricted to the archaeal community. By contrast, for the shotgun metagenomic data, cross-domain relative abundances have now been quantified and reported, as described above, thereby providing the domain-level context requested by the reviewer.

L165-L167: 97% of what percentage of all sequences from phylogenetically distinct microbes?

Response: Thank you for this comment. The 97% value refers to the proportion of all sequences identified as archaea, that is, the cumulative relative abundances of all archaeal ASVs in the dataset. We have clarified this point in the revised manuscript (Lines 180 – 181).

L215-L218: I agree with this statement if it is about bacteria, though the cell count numbers for microbes on surface plastics is on the order of 10^{21}, which is incredibly small compared to what's in the water column at the same depths. Moreover, the data from other studies suggest archaea have low relative abundance in the plastisphere and the data from this study suggests that stochastic processes drive their presence.

Response: Thank you for this important comment. We agree that, in absolute terms, the microbial

biomass associated with marine plastic debris is small relative to that of the surrounding ocean water column. As estimated by Zhao et al[28], the plastisphere holds microbial cells corresponding to approximately 1% of the microbial cells in the ocean surface microlayer. Because a substantial fraction of marine plastic debris is concentrated in the oligotrophic central gyres, these plastics may have unquantified consequences for the surrounding water-column microbial diversity, community function, and biogeochemical cycles[28]. The upper-bound estimate of plastisphere-associated carbon (1.1 $\times 10^4$ metric tons) in Zhao et al's study approaches the estimated cellular carbon mass in global rivers (2.4 $\times$ 10^4 metric tons) and in both polar regions (8 $\times$ 10^4 metric tons).

These are substantial quantities that could have meaningful ecological impacts and therefore warrant attention. Moreover, plastics represent a pervasive, human-introduced habitat; the ocean and other environmental compartments, and the natural microbial world, would not be exposed to these perturbations in the absence of anthropogenic plastic pollution. With continuing increases in plastic production, waste emission, and environmental persistence, the microbial load associated with the plastisphere, and its potential ecological consequences, can be expected to rise over time.

For the second point, our results indicate that it is the assembly and community structure of plastisphere archaea, rather than their overall occurrence, that are more strongly governed by stochastic processes. In other words, the arrival and establishment of particular archaeal taxa on individual plastic particles may be relatively stochastic, but once plastic enters the environment it is rapidly colonized by microorganisms. From this broader perspective, the occurrence of archaea on plastic surfaces is not accidental, but a predictable consequence of plastic pollution as a novel habitat. Indeed, archaea were detected consistently across all plastisphere samples in our study.

More broadly, all of these microorganisms and their associated potential impacts are ultimately induced by the introduction of plastic into the environment, which is itself a deterministic anthropogenic factor. Without plastic pollution, these plastisphere-specific microbial assemblages and the associated ecological effects would not exist.

L220-L235: I recognize the logic behind this notion and appreciate it. Might other sources of methane (e.g., livestock; hydrothermal vent fluid) be more important contributors? We are likely talking about small relative proportions of archaea in the plastisphere contributing to methane cycling.

Response: Thank you for this important comment. We agree that other methane sources, such as livestock systems or hydrothermal environments, are likely to be far more important contributors to global methane fluxes than plastisphere-associated archaea. Our intention was not to imply that plastisphere archaea represent a dominant methane source, but rather to highlight a previously overlooked dimension of plastic pollution: namely, that plastic-associated archaeal communities may carry methane-cycling potential and could therefore contribute to local methane dynamics in ways that remain largely unquantified.

From our perspective, the key point is that plastic is a novel, anthropogenic habitat that would not exist in natural systems in the absence of plastic pollution. Accordingly, any plastisphere-associated methane-cycling potential and its possible ecological implications are also anthropogenically introduced. Given the continued growth, persistence, and fragmentation of plastic waste in the environment, the abundance of plastisphere-associated microorganisms and their potential ecological effects may also increase over time. Although the magnitude of this contribution is currently unknown, identifying the direction and potential of such impacts is an important first step toward more comprehensive risk assessment of plastic pollution.

To make this limitation explicit, we have revised the manuscript to avoid overstatement and added the following wording to the ending paragraph:

Nevertheless, because our analyses are based on taxonomic composition and metagenomic functional inference, direct links to viability, in situ activity, and realized biogeochemical rates remain to be established. In addition, the ecological implications highlighted here are based on relative comparisons rather than direct measurements of impact magnitude. For example, we still do not know to what extent plastic pollution contributes to microbial redistribution and invasion in the ocean, nor what magnitude of greenhouse-gas-related effects may be associated with plastisphere archaea. Further research is needed to directly link these microbial shifts to real-world ecological and health outcomes of plastic pollution, and to quantitatively assess the full risks of the plastisphere. Addressing these questions will require cross-disciplinary collaboration among researchers in environmental microbiology, environmental impact assessment, public health, and data science. Moreover, our study only considered surface waters and significant knowledge gaps remain regarding plastisphere dynamics throughout the water column and deep ocean ecosystems. Future work should also investigate the ecological mechanisms underlying

plastisphere archaeal community assembly, including the roles of biofilm succession, substrate heterogeneity, and dispersal dynamics, to advance predictive understanding of how plastic pollution reshapes microbial diversity and function in marine ecosystems. (Lines 519 – 534)

L237-L258: This is a really cool idea when paired with the notion that microbes adapt to plastic and can "perturb" nutrient cycles. I suspect that this would be more pertinent to the bacteria living in epiplastic biofilms though not archaea.

Response: Thank you for this comment. We agree that bacteria, being much more abundant than archaea in the plastisphere, are likely to have a larger influence on nutrient cycling, consistent with our previous global plastisphere bacterial study (https://doi.org/10.1016/j.xinn.2023.100543; recognized as Best Paper, Most Cited, and Most Popular Paper of the journal) and other publications. Our study, by contrast, focuses on archaea—the third domain of life, which has important ecological and evolutionary significance—to examine how plastic pollution may affect archaeal diversity, distribution, and potential functional impacts. By investigating these patterns, we aim to provide a more comprehensive understanding of the ecological consequences of plastic pollution. Such understanding is essential for informing effective management strategies and for identifying the key drivers that shape microbial assembly and responses in anthropogenically altered habitats.

L284: there are no metatranscriptomic nor respiration data in this study. How can you be so certain that archaeal communities are "highly" active in biogeochemical processes in the plastisphere? I would revise this to reflect having amplicon and metagenomic data. Are there any pathways that are enriched on different polymer types?

Response: Thank you for this important comment. We agree that our amplicon and metagenomic data support inference of archaeal community composition and functional potential, but not direct activity. We have therefore revised the manuscript throughout to avoid overstating activity and now describe plastisphere archaeal communities as exhibiting enhanced biogeochemical functional potential rather than being highly active.

Regarding polymer-specific effects, we agree that polymer type could, in principle, influence microbial community composition and associated functional signatures within the plastisphere. However, polymer-specific analysis was not the primary aim of the present study. Our objective

was to identify the generalized archaeal ecological patterns associated with the plastisphere as a novel anthropogenic habitat under real field conditions, using a broad paired design rather than incubation experiments based on predefined polymer types. In this context, we focused on the overall plastisphere signal rather than on within-plastic heterogeneity.

To address this question more directly, we performed an additional analysis during revision using published marine metagenomic datasets that included multiple polymer types together with non-plastic reference substrates. Across four independent studies, we compared within-polymer, between-polymer, and plastic-versus-non-plastic Bray–Curtis dissimilarities for both archaeal taxonomic composition and functional gene profiles. The results were inconsistent across studies and did not support a robust general conclusion regarding the relative importance of polymer type versus substrate identity (Supplementary Note S6 and Fig. S11). We attribute this inconsistency primarily to the limited scope of the currently available datasets, including low replication per polymer type, narrow polymer diversity, sparse non-plastic reference groups, and the restricted ecological representativeness of incubation-based experiments. In contrast, our own study was designed to resolve the broader plastisphere signal under field conditions using a much larger set of paired plastisphere and ambient seawater samples across diverse coastal environments. We therefore interpret the consistent taxonomic and functional separation observed in our dataset as evidence of a robust and generalizable plastisphere-specific archaeal signal, while recognizing that polymer-specific effects remain an important question for future work.

The related analyses, methods, results, and figures have now been incorporated into the revised manuscript and Supplementary Information.

Supplementary Note S6 Archaeome dissimilarity patterns across polymer types and substrate types

To evaluate whether polymer type structures plastisphere archaeal communities, we compared three categories of Bray–Curtis dissimilarity across four independent studies that included multiple polymer types and non-plastic reference substrates with known polymer identity (wos_50, wos_85, wos_2, and wos_134): within-polymer, between-polymer, and plastic-versus-non-plastic dissimilarity. Analyses were conducted for both archaeal taxonomic composition and functional gene profiles (Supplementary Fig. S11).

Overall, results were inconsistent across studies. In some datasets, plastic-versus-non-plastic dissimilarity exceeded between-polymer dissimilarity, suggesting that substrate identity explained more variation than polymer chemistry. In others, this pattern was not observed, and similar inconsistency was found for functional gene profiles. We attribute this lack of consistency primarily to the limited size and scope of the available datasets, including low replication per polymer type, narrow polymer diversity, sparse non-plastic reference groups, and the restricted ecological representativeness of incubation-based experiments.

These results indicate that currently available public datasets are insufficient to robustly disentangle polymer-type effects from broader substrate-identity effects in plastisphere archaeal communities. In contrast, the present study was designed to resolve the broader plastisphere signal under field conditions using a much larger set of paired plastisphere and ambient seawater samples across diverse coastal environments. We therefore interpret the consistent taxonomic and functional separation observed in our dataset as evidence of a generalizable plastisphere-specific archaeal signal, while recognizing that polymer-specific effects remain an important question for future work.

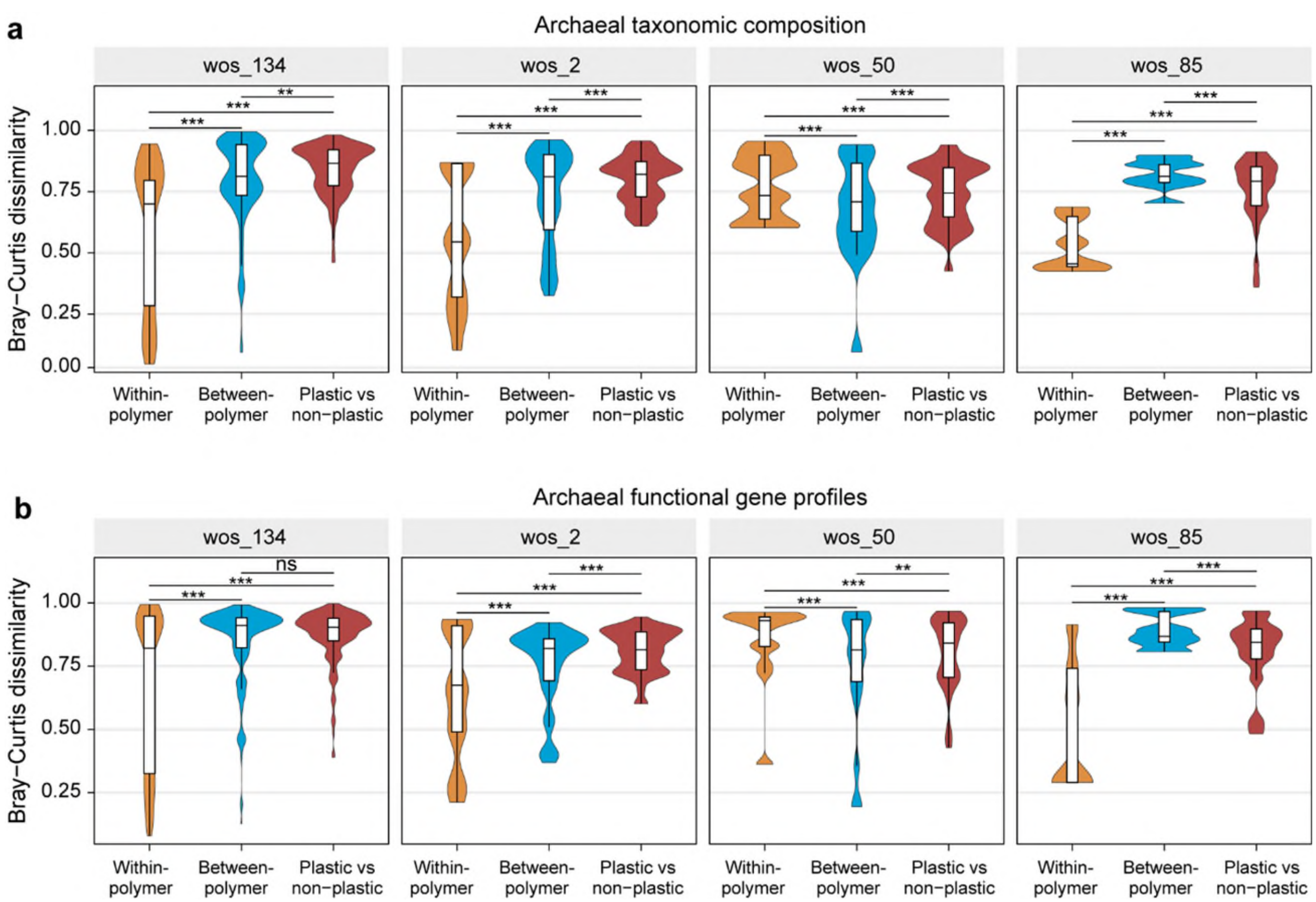

Fig. S11 Comparison of archaeal community dissimilarity across polymer types and substrate types. a, Taxonomic composition based on species-level GTDB annotation. **b,** Functional gene profiles based on combined archaeal carbon, nitrogen, phosphorus, sulfur, and methane cycling genes. In each panel, three categories of pairwise Bray–Curtis dissimilarity are shown across four independent studies: within-polymer, between-polymer, and plastic-versus-non-plastic. Each distribution comprises 999 bootstrap-sampled pairwise distances to ensure equal sample sizes across categories despite group imbalance. The horizontal line within each box indicates the median. Statistical significance was assessed using Wilcoxon rank-sum tests (*** $P < 0.001$, ** $P < 0.01$, ns = non-significant).

L286-L288: Scaling up is a cool idea, but the context matters here. If we posit that Zhao et al. 2020's microbial carrying capacity numbers are conservative (i.e. 10^{21} microbes on surface plastics), and that 0.5% of these microbes may be archaea (i.e. $5x10^{18}$), archaeal numbers are miniscule compared to the rest of the plastisphere and what's in the surrounding water column. I agree with your statement "Moreover, our study only considered surface waters and significant knowledge gaps remain regarding plastisphere dynamics throughout the water column and deep ocean ecosystems" on L472-L473. However, even considering microbial biomass on plastics in the ocean's interior, which tend to be significantly smaller pieces of plastic than what's at the surface, there's going to be limited space for microbes to colonize.

Response: Thank you for this comment. We agree that absolute archaeal abundance on plastics is low relative to the total microbial biomass in the ocean. Estimates from Zhao et al. [28] suggest that surface plastic debris may carry on the order of 2.1×10^{21} to 3.4×10^{21} microbial cells, which is considered conservative. Assuming archaea comprise approximately 0.5% of these cells, their numerical contribution remains small compared with the overall plastisphere community and surrounding seawater. Another estimate from the team of L. A. Amaral-Zettler (Nature Reviews Microbiology)[29] indicates that microbial biomass associated with plastics corresponds to roughly 1% of oceanic microbial cells, although the true value may be higher. Notably, one gram of plastic can support microbial abundances exceeding that in hundreds of liters of surrounding water[29], highlighting that plastics act as microbial hotspots, including for archaea, with potential localized impacts on community structure and biogeochemical processes.

Moreover, as plastic production and environmental accumulation continue to increase, and as

plastics persist and fragment into smaller pieces with higher surface-area-to-volume ratios, the total microbial biomass associated with marine plastics and its potential ecological impacts are expected to grow over time. Plastics, as entirely anthropogenic entities, introduce novel habitats and microbial assemblages that would not exist in the environment otherwise; thus, the associated microbial impacts are also anthropogenically added.

Importantly, low abundance does not imply low ecological significance. For example, pathogenic bacterial species represent less than 1% of total bacteria but cause millions of deaths annually. Similarly, rare species can drive critical ecosystem functions. Microalgae, often present at low abundance relative to bacteria, can nevertheless induce substantial ecological consequences, including harmful algal blooms and toxin production that affect wildlife, human health, and economic activities.

Finally, regarding plastics in the ocean interior, while smaller particles may carry fewer cells individually, fragmentation of larger plastic debris increases the total number of particles and total available surface area, potentially enhancing their capacity to harbor microbes. Additionally, plastic particles enriched with organic matter may increase in bulk density and settle, expanding the potential volume of plastisphere habitat in the water column. Taken together, these factors indicate that even relatively low-abundance archaeal populations on plastics could contribute meaningfully to localized microbial processes and ecosystem impacts.

L496-L409: what about relative abundance of archaea and bacteria? I would include those data in Figure 1.

Response: Thank you for this helpful suggestion. As noted previously, we agree that cross-domain relative abundance data are important for contextualizing archaeal results. In the revised manuscript, we have therefore added a quantitative comparison of the relative abundances of Bacteria, Archaea, and Fungi across all metagenomic samples, with the methods and results now provided in Supplementary Note S1 and Supplementary Fig. S2. These data are also referenced in the opening paragraph of the Results section.

Because Figure 1 focuses specifically on archaeal community characteristics inferred from archaeal-targeted amplicon sequencing, we chose not to integrate cross-domain metagenomic abundance data directly into that figure. Doing so would mix datasets generated for different

analytical purposes and could reduce figure clarity. Instead, we present the domain-level context in the Supplementary Information and explicitly cite it in the main text. We believe this approach provides readers with a clear understanding of archaeal relative abundance while keeping the main text and figures focused and easy to follow.

L424-L432: I see the train of thought here, but how can you come to this conclusion when stochastic processes.

Response: We thank the reviewer for this important comment. We agree that the dominance of stochastic processes requires careful interpretation when considering subsequent environmental analyses. In our view, however, these analyses address different ecological questions and are therefore not contradictory. The stochasticity ratio and iCAMP analyses were used to infer the overall balance of community assembly processes, showing that archaeal assembly is primarily governed by stochastic mechanisms. By contrast, the later analyses were intended to identify environmental correlates of archaeal taxonomic and functional variation, rather than to suggest that deterministic environmental filtering overrides the overall stochastic assembly regime.

In other words, stochastic dominance does not imply that environmental conditions have no influence; rather, it indicates that the total variation explained by measured deterministic factors is smaller than that attributable to stochastic processes such as dispersal limitation and drift. We have revised the manuscript to clarify that the environmental variables are interpreted as correlates or modulators of archaeal variation within a predominantly stochastic assembly framework, rather than as dominant deterministic drivers. This interpretation aligns with previous archaeal ecology studies showing that communities can remain largely stochastic in assembly while still exhibiting significant associations with environmental conditions and ecosystem functioning[30].

To improve clarity, we also modified the title of this section and added explanatory text at the beginning as follows:

Environmental correlates of plastisphere archaeal variation and ecological implications

Although stochastic processes dominated plastisphere archaeal community assembly, this does not preclude environmental variables from being associated with archaeal taxonomic and functional variation. We therefore examined which measured environmental factors were

correlated with archaeal community composition and functional profiles, with the aim of identifying environmental correlates and potential modulators of archaeal variation. (Lines 426 – 431)

L449-L450: “everything is everywhere, but the environment selects.” Presence does not mean activity nor processes occur nor viability.

Response: We thank the reviewer for this important clarification. We fully agree that the presence of taxa or functional genes does not demonstrate viability, in situ activity, or realized process rates. Our study therefore supports inference about community structure and functional potential, rather than direct demonstration of active methane, nitrogen, or sulfur transformation. In the revised manuscript, we have carefully toned down process-level language throughout and added a clearer statement in the Conclusions and outlook section emphasizing that direct links to viability, activity, and realized biogeochemical rates remain to be established. The revised parts are as follows:

The plastisphere represents a novel human-made habitat within the ocean microbiome that may redistribute microbial taxa and alter biogeochemical functional potential in marine systems. Recognizing these shifts is important for predicting the broader environmental impacts of plastic debris beyond the well-known direct hazards to wildlife. Here, we show that plastics host a restructured archaeal community characterized by higher taxonomic diversity and greater heterogeneity than the surrounding seawater. Correspondingly, archaeal functional gene profiles differ between the plastisphere and bulk water, with enrichment of pathways related to CH_4, N, and S cycling in the plastisphere, highlighting the potential for plastic pollution to influence biogeochemical cycling[1, 2] and climate-related processes[3] from an archaeal perspective. As plastic waste continues to accumulate globally, the plastisphere will expand accordingly, leading to greater prevalence and density of archaeal communities in marine ecosystems, and thereby amplifying their ecological impacts.

Nevertheless, because our analyses are based on taxonomic composition and metagenomic functional inference, direct links to viability, in situ activity, and realized biogeochemical rates remain to be established. In addition, the ecological implications highlighted here are based on relative comparisons rather than direct measurements of impact magnitude. For example, we still do not know to what extent plastic pollution contributes to microbial redistribution and invasion in the ocean, nor what magnitude of greenhouse-gas-related effects may be associated with

plastisphere archaea. Further research is needed to directly link these microbial shifts to real-world ecological and health outcomes of plastic pollution, and to quantitatively assess the full risks of the plastisphere. Addressing these questions will require cross-disciplinary collaboration among researchers in environmental microbiology, environmental impact assessment, public health, and data science. Moreover, our study only considered surface waters and significant knowledge gaps remain regarding plastisphere dynamics throughout the water column and deep ocean ecosystems. Future work should also investigate the ecological mechanisms underlying plastisphere archaeal community assembly, including the roles of biofilm succession, substrate heterogeneity, and dispersal dynamics, to advance predictive understanding of how plastic pollution reshapes microbial diversity and function in marine ecosystems.

L544-L549: Was there an opportunity to bin archaeal metagenome-assembled genomes from the dataset? What about with a coassembly? I suppose the sequencing depth is not deep enough and low abundance members are challenging to get MAGs for.

Response: Thank you for this helpful suggestion. As noted above, we followed up on this point by conducting genome-resolved metagenomic binning and recovered 23 non-redundant archaeal MAGs, including 5 derived from plastisphere samples. Notably, all plastisphere-derived MAGs were affiliated with Nitrososphaerales, whereas Poseidoniales MAGs were recovered exclusively from bulk-water metagenomes. These MAGs are now described in the revised Methods and Supplementary Note S4, and the main manuscript now includes a brief summary of the genome-resolved results in both the community-composition and nitrogen-cycling sections. We agree that low abundance and the complexity of biofilm-associated communities likely constrain archaeal MAG recovery, but the genomes recovered here nevertheless provide valuable genome-resolved support for the major gene-centric functional patterns identified in this study. The revised related parts are as follows:

Genome-resolved metagenomic analysis further supported this pattern: among 23 recovered archaeal metagenome-assembled genome (MAGs), all five plastisphere-derived MAGs were affiliated with Nitrososphaerales, whereas Poseidoniales MAGs were recovered exclusively from bulk water (Supplementary Note S4 and Figs. S8 and S9). (Lines 193 – 197 in the main text)

Our genome-resolved analyses further supported this pattern: Nitrososphaerales MAGs were enriched in the plastisphere and carried key nitrogen-cycling genes, including complete *amoABC*,

ureABC, and *nirK*, consistent with the potential for ammonia oxidation and related nitrogen transformations in plastisphere biofilms (Supplementary Note S4 and Figs. S8 and S9). (Lines 273 – 276 in the main text)

Notably, *Nitrososphaera* emerged as the single most important biomarker, consistent with the genome-resolved metagenomic results showing enrichment of Nitrososphaerales MAGs in the plastisphere (Supplementary Figs. S8 and S9). Together, these results indicate that ammonia-oxidizing Nitrososphaerales-related archaea are a prominent and recurrent signature of the plastisphere archaeome. (in Supplementary Note S3)

Supplementary Note S4 Recovery and characterization of archaeal metagenome-assembled genomes

Using genome-resolved metagenomic binning, we reconstructed 23 non-redundant archaeal metagenome-assembled genomes (MAGs) that met at least medium-quality draft criteria (completeness ≥50%, contamination ≤10%; Supplementary Fig. S8)[19]. Taxonomic classification within the GTDB framework indicated two dominant archaeal lineages: Nitrososphaerales (*n* = 11; phylum Thermoproteota, class Nitrososphaeria) and Poseidoniales (*n* = 12; phylum Thermoplasmatota, class Poseidoniia). Notably, all five plastisphere-derived MAGs were affiliated with Nitrososphaerales, whereas all Poseidoniales MAGs were recovered exclusively from bulk-water metagenomes (Supplementary Fig. S8). The plastisphere signal was robust to dereplication strategy, as dereplication restricted to plastisphere-derived bins recovered the same five Nitrososphaerales MAGs. Despite comparable sequencing depth across habitats, fewer archaeal MAGs were recovered from plastisphere samples, consistent with the greater complexity and strain heterogeneity expected in surface-attached biofilms, which can reduce assembly contiguity and genome recovery[20]. This pattern is also consistent with the higher archaeal taxonomic diversity observed in the amplicon data (Fig. 1 in the main text).

Because Nitrososphaerales/Nitrososphaeria comprise ammonia-oxidizing archaea (AOA), typically characterized by genes encoding ammonia monooxygenase[21], we evaluated the nitrogen-cycling potential of recovered MAGs by annotating predicted proteins against NCycDB[22]. Among the five plastisphere-derived Nitrososphaerales MAGs, two encoded the complete ammonia monooxygenase gene set (*amoA*, *amoB*, *amoC*), supporting genomic potential for archaeal ammonia oxidation (Supplementary Fig. S8). One of these MAGs additionally contained a complete urease operon (*ureA*, *ureB*, *ureC*) (Supplementary Fig. S8), suggesting the potential

to hydrolyze urea and thereby generate ammonia for nitrification[23]. Given that urea is widely available in marine systems and can support AOA growth and activity[24], this genomic configuration is consistent with the possibility that plastisphere-associated AOA exploit dissolved organic nitrogen substrates within the biofilm microenvironment.

We further detected *nirK* (copper-containing nitrite reductase) in most plastisphere Nitrososphaerales MAGs (Supplementary Fig. S8). In AOA, *nirK* has been shown to generate nitric oxide (NO) from nitrite and/or hydroxylamine-derived intermediates and is considered central to archaeal nitrogen metabolism[25]. NO-related reactions in AOA have also been linked to nitrous oxide (N_2O) formation under low-oxygen conditions[26]. Although gene presence alone does not demonstrate in situ activity, these marker genes suggest that plastisphere-associated AOA may contribute to localized nitrification and NO/N_2O-related transformations within redox-stratified plastisphere biofilms.

In contrast, bulk-water Poseidoniales MAGs lacked *amo* genes (Supplementary Fig. S8), consistent with genome-resolved studies describing Poseidoniales (Marine Group II archaea) as predominantly aerobic (photo)heterotrophs involved in dissolved and particulate organic matter turnover, with additional lineage-specific traits including transport and degradation of organic substrates and, in some taxa, nitrate-reduction capacity[27].

Finally, metagenomic read recruitment to MAG-predicted genes showed that Nitrososphaerales MAGs were significantly more abundant in plastisphere samples than in bulk water, whereas Poseidoniales MAGs showed the opposite pattern (Wilcoxon rank-sum tests, $P < 0.001$; Supplementary Fig. S9).

Collectively, the selective recovery and enrichment of AOA-affiliated MAGs in the plastisphere, together with their encoded potential for ammonia oxidation, urea utilization, and NO-related nitrite reduction, provide genome-resolved support for our gene-centric inference that the plastisphere may act as a localized hotspot for archaeal nitrogen transformations in coastal marine ecosystems.

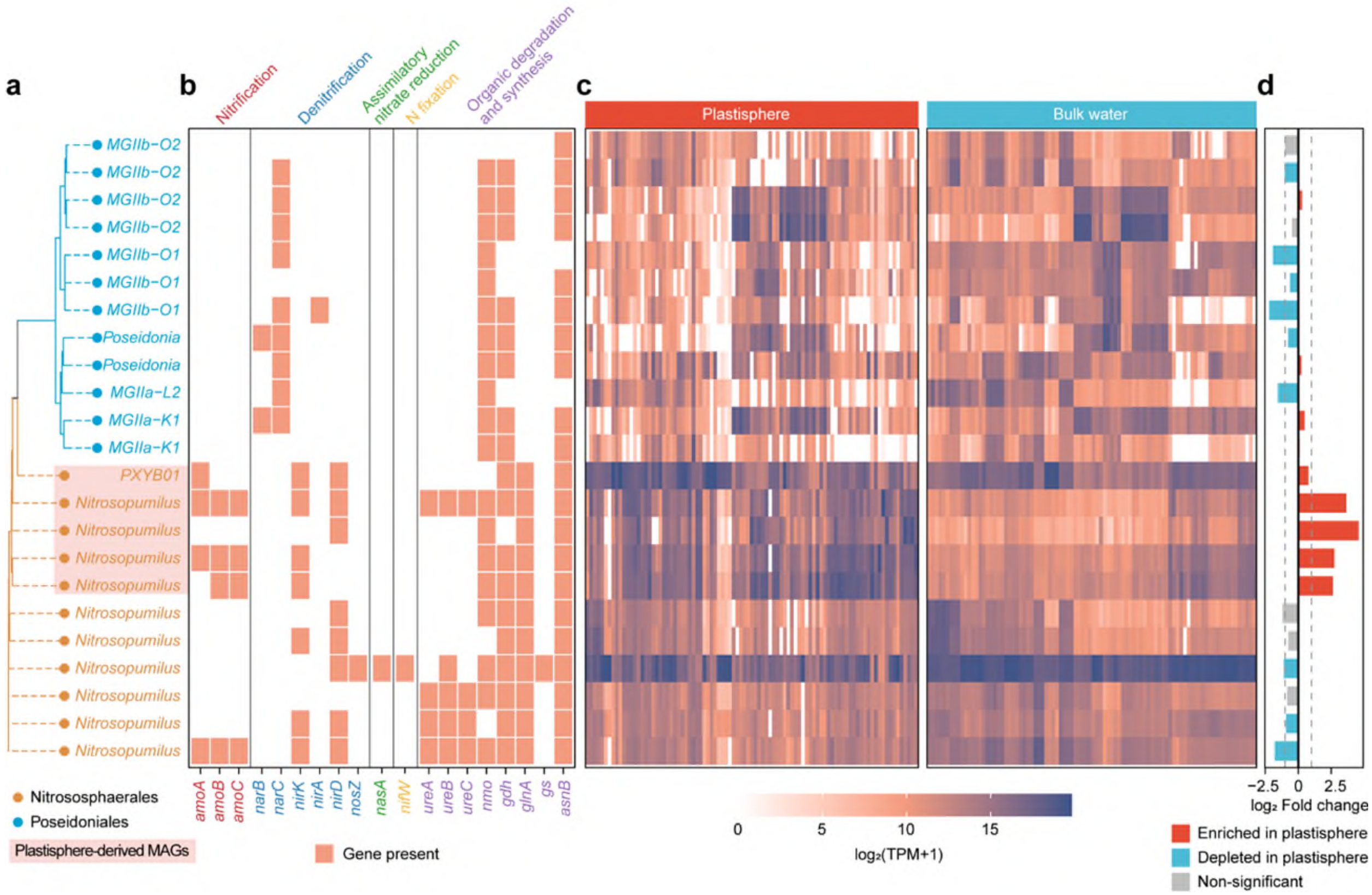

Fig. S8 Genome-resolved characterization of archaeal MAGs recovered from plastisphere and bulk-water communities. a, Maximum-likelihood phylogenetic tree of 23 archaeal MAGs inferred from ar53 marker genes. Tips are coloured by taxonomic order (orange, Nitrososphaerales; blue, Poseidoniales); plastisphere-derived MAGs are highlighted with a pink background. **b,** Presence of nitrogen-cycling genes across all 23 MAGs, grouped by functional process according to NCycDB classification. **c,** Heat map of MAG relative abundance ($\log_2$-transformed TPM) across plastisphere and bulk water samples. **d,** Differential abundance of each MAG between plastisphere and bulk water. Red and blue bars indicate significant enrichment in plastisphere and bulk water, respectively; grey bars indicate no significant difference (Wilcoxon rank-sum test, $P < 0.05$).

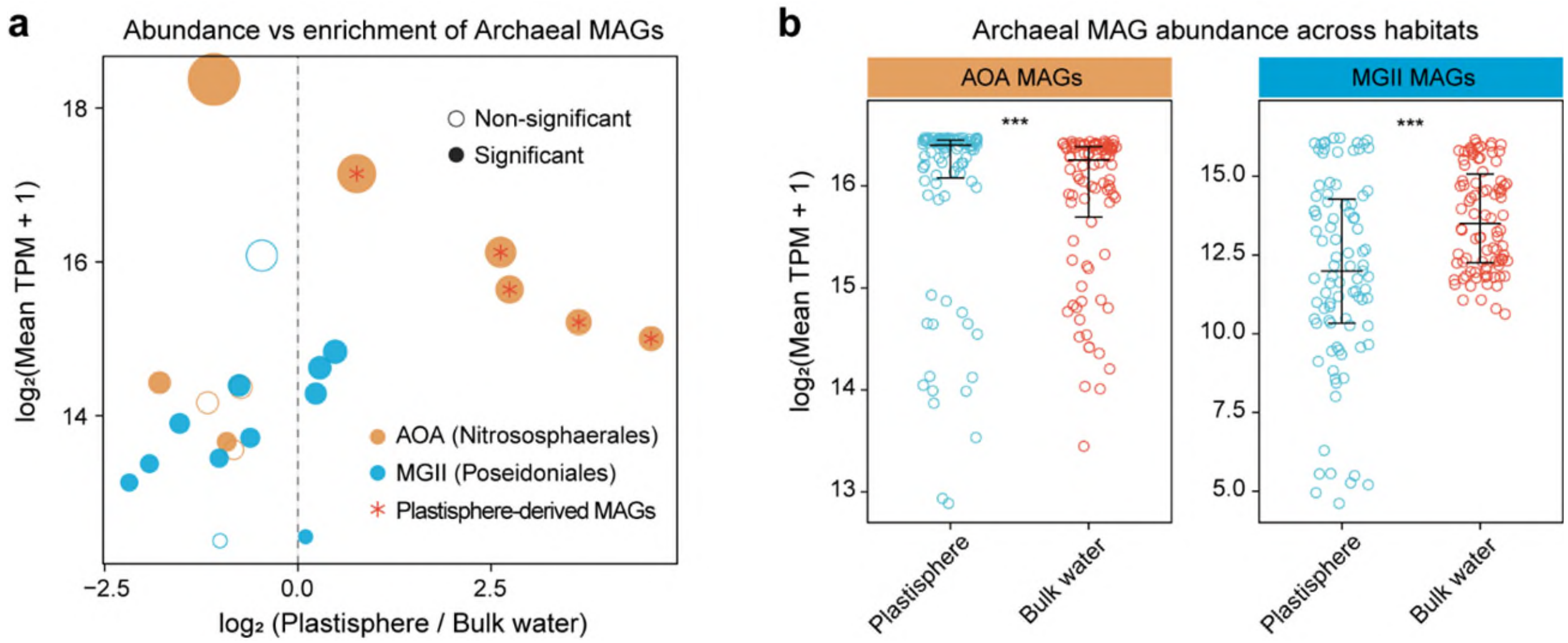

Fig. S9 Differential abundance of archaeal MAGs between plastisphere and bulk water. a, Bubble plot showing the relationship between differential abundance and mean abundance for each MAG. Bubble size represents mean total TPM, and colour indicates taxonomic affiliation (orange, Nitrososphaerales; blue, Poseidoniales). Plastisphere-derived MAGs are marked with asterisks. Filled and open symbols indicate statistically significant and non-significant differences, respectively (Wilcoxon rank-sum test, $P < 0.05$). **b,** Mean abundance of Nitrososphaerales and Poseidoniales MAGs in plastisphere and bulk-water samples. Significance was assessed using Wilcoxon rank-sum tests ($n = 82$ per group; *** $P < 0.001$).

Reviewer #4 (Remarks to the Author):

The present study addresses an important topic: the impact of plastic contamination on microbial communities and their metabolic potentials in coastal marine systems. The authors sampled many different coastal environments and collected plastic debris along with surrounding water to compare planktonic and plastic attached microbiomes by a metagenomic approach. The authors thereby focus on Archaea rather than Bacteria. The strengths of the study lay in the generation of a comprehensive data set for coastal marine systems, although Archaea have already been investigated in marine and terrestrial systems. However, the manuscript would greatly profit from acknowledging such studies and setting the results in context with findings from such studies that previously addressed Bacteria and Archaea. Furthermore, Archaea are generally lower in abundance in the plastisphere than Bacteria. In particular when metagenomes are considered, the rather low abundance of Archaea might result in a low number of reads. I would strongly advise to add some analysis on how good the Archaeal genes were captured (representativeness) to avoid conclusions based on data noise. A discussion on the relevance of Archaea relative to Bacteria would like wise strengthen the study.

Response: We thank the reviewer for these helpful comments. We agree that Archaea are numerically lower than Bacteria in plastisphere communities, and for this reason, archaeal community-level analyses—including composition, diversity, and assembly mechanisms—were performed using archaeal-targeted amplicon sequencing with the Arch519F/Arch915R primer pair. Rarefaction curve analyses indicate that our sequencing depth and sample number were sufficient to capture the dominant archaeal diversity patterns in both plastisphere and seawater samples (Supplementary Fig. S22), supporting the robustness of these community-level analyses.

For functional analyses, we used metagenomic data to characterize archaeal gene potential. To ensure accurate archaeal assignment, we employed a GTDB-based database containing an expanded set of archaeal genomes, reducing underrepresentation relative to standard databases. Taxonomic annotation was first used to extract archaeal-affiliated genes from the full metagenomic catalog, establishing an archaeal-only gene catalog and abundance table. Functional annotation was then performed specifically on this archaeal gene set. This two-step workflow—taxonomy-first, function-second—together with the comprehensive reference database, represents a widely used and best-practice approach in metagenomic archaeal functional analysis.

Additionally, we successfully recovered 23 archaeal MAGs, including 5 from plastisphere samples, providing genome-resolved support that is consistent with the gene-centric functional patterns. Together, these approaches ensure that both community-level and functional analyses of plastisphere archaea are reliable and robust, providing comprehensive insight into archaeal diversity and potential functional capacity in this novel anthropogenic habitat.

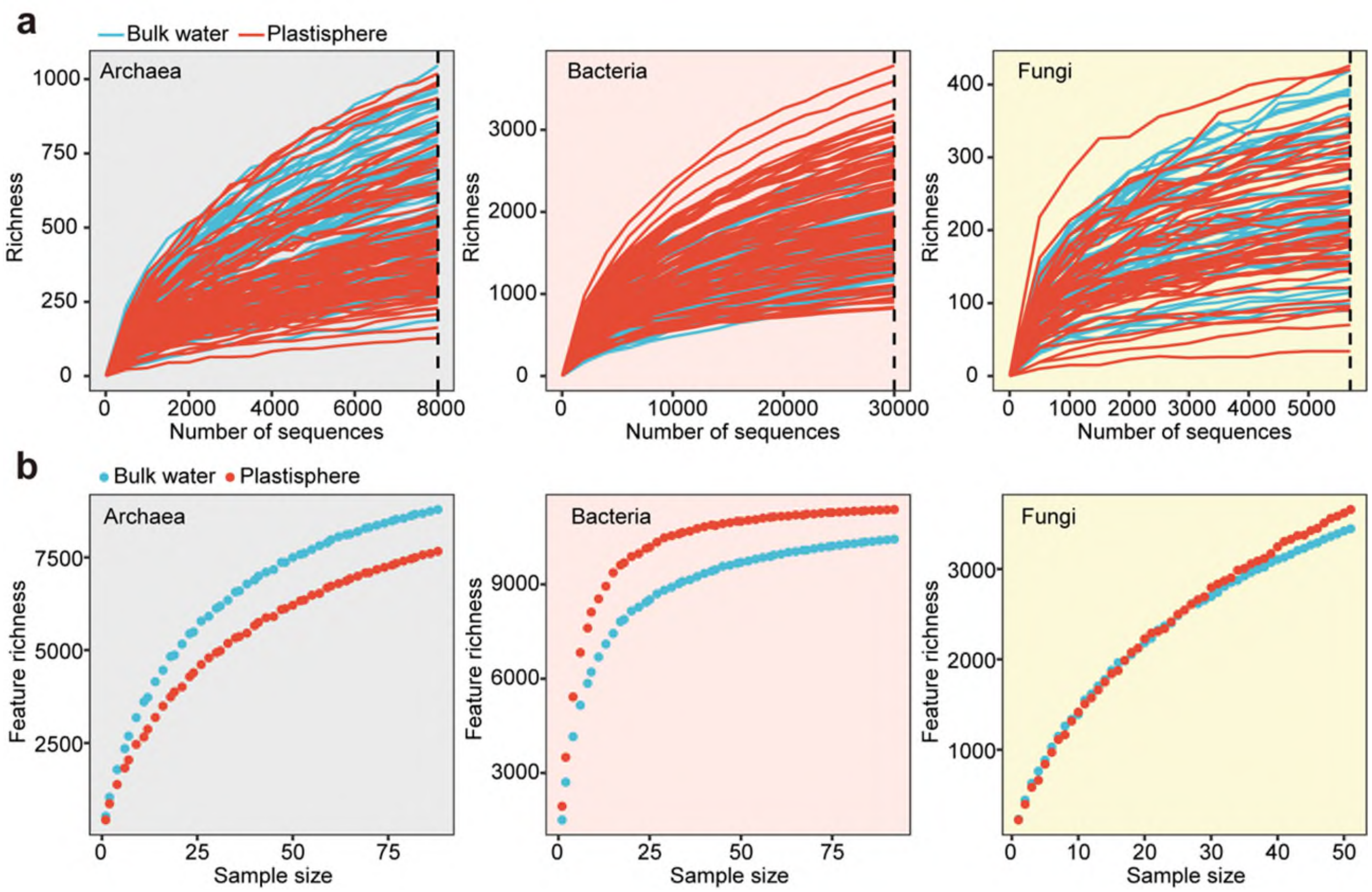

Fig. S22 Rarefaction curves. a, The number of archaeal, bacterial and fungal ASVs with the increasing number of sequences (the sequencing depth). **b,** Accumulation curves of the number of archaeal, bacterial and fungal ASVs with an increasing number of samples (the sample size). These curves flatten out at the final sequence depth (8,000 for archaea, 30,000 for bacteria, and 5,700 for fungi) and sample size (n = 88 per archaeal group, n = 92 per bacterial group and n = 51 per fungal group) chosen for this study, indicating that our samples could capture most of microbial members from the plastisphere and bulk water and thus were sufficient for the comparative analyses.

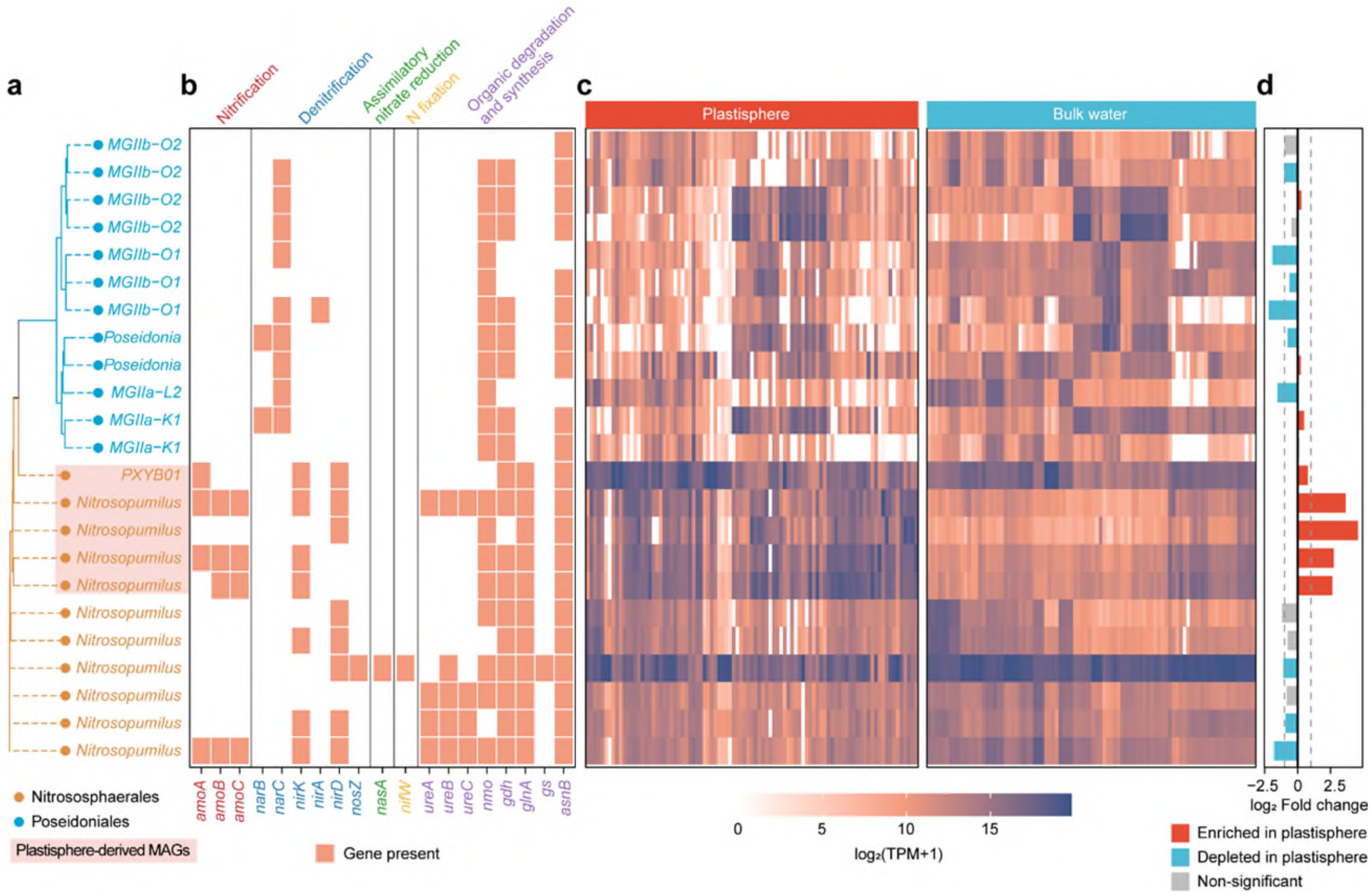

Fig. S8 Genome-resolved characterization of archaeal MAGs recovered from plastisphere and bulk-water communities. a, Maximum-likelihood phylogenetic tree of 23 archaeal MAGs inferred from ar53 marker genes. Tips are coloured by taxonomic order (orange, Nitrososphaerales; blue, Poseidoniales); plastisphere-derived MAGs are highlighted with a pink background. **b,** Presence of nitrogen-cycling genes across all 23 MAGs, grouped by functional process according to NCycDB classification. **c,** Heat map of MAG relative abundance (log_2-transformed TPM) across plastisphere and bulk water samples. **d,** Differential abundance of each MAG between plastisphere and bulk water. Red and blue bars indicate significant enrichment in plastisphere and bulk water, respectively; grey bars indicate no significant difference (Wilcoxon rank-sum test, $P < 0.05$).

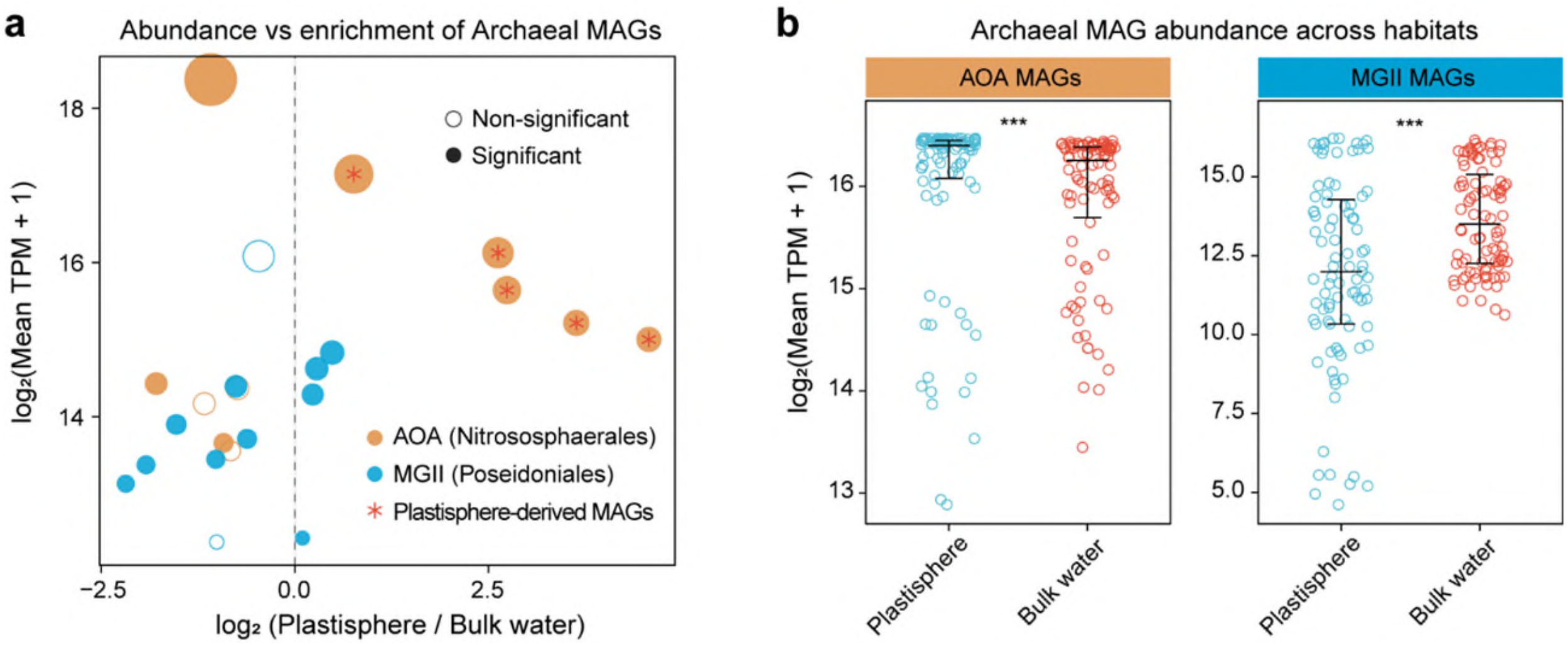

Fig. S9 Differential abundance of archaeal MAGs between plastisphere and bulk water. a, Bubble plot showing the relationship between differential abundance and mean abundance for each MAG. Bubble size represents mean total TPM, and colour indicates taxonomic affiliation (orange, Nitrososphaerales; blue, Poseidoniales). Plastisphere-derived MAGs are marked with asterisks. Filled and open symbols indicate statistically significant and non-significant differences, respectively (Wilcoxon rank-sum test, $P < 0.05$). **b,** Mean abundance of Nitrososphaerales and Poseidoniales MAGs in plastisphere and bulk-water samples. Significance was assessed using Wilcoxon rank-sum tests ($n = 82$ per group; *** $P < 0.001$).

The data presented here is based on DNA analysis, not allowing for conclusions on activities. Thus, many conclusions on activities are speculative and the manuscript would profit from toning down speculative statements.

Response: Thank you for this important comment. We have revised the manuscript throughout to tone down statements implying direct microbial activity and to clarify that our inferences are based on DNA-level analyses, including metagenomic and archaeal-targeted amplicon sequencing. We also explicitly acknowledge these methodological limitations and the need for future work to link taxonomic and functional potential to actual in situ activity, viability, and process rates in the Conclusions and outlook section, as follows:

Nevertheless, because our analyses are based on taxonomic composition and metagenomic functional inference, direct links to viability, in situ activity, and realized biogeochemical rates remain to be established. In addition, the ecological implications highlighted here are based on

relative comparisons rather than direct measurements of impact magnitude. For example, we still do not know to what extent plastic pollution contributes to microbial redistribution and invasion in the ocean, nor what magnitude of greenhouse-gas-related effects may be associated with plastisphere archaea. Further research is needed to directly link these microbial shifts to real-world ecological and health outcomes of plastic pollution, and to quantitatively assess the full risks of the plastisphere. Addressing these questions will require cross-disciplinary collaboration among researchers in environmental microbiology, environmental impact assessment, public health, and data science. Moreover, our study only considered surface waters and significant knowledge gaps remain regarding plastisphere dynamics throughout the water column and deep ocean ecosystems. Future work should also investigate the ecological mechanisms underlying plastisphere archaeal community assembly, including the roles of biofilm succession, substrate heterogeneity, and dispersal dynamics, to advance predictive understanding of how plastic pollution reshapes microbial diversity and function in marine ecosystems. (Lines 519 – 534)

Specific comments

L58 and throughout the manuscript. Please adhere to current taxonomy. Euryarchaeota are Methanobacteriota, …..

Response: Thank you for this suggestion. Archaeal taxonomy in this study was derived primarily from archaeal-targeted 16S rRNA gene amplicon data using the RDP classifier with the SILVA reference database, which follows the classical archaeal taxonomy (for example, *Euryarchaeota*) and is still commonly used in archaeal ecology studies, including recent high-impact work (for example, *Nature Climate Change* 13: 561–569[30]). Because these analyses are based on amplicon sequences rather than genome-resolved taxonomy, direct reassignment to recent genome-based phylum names under frameworks such as GTDB is not straightforward. We therefore consider the use of a consistent SILVA-based taxonomy to be the most appropriate approach for the present amplicon-based community analyses. To make this clearer in the manuscript, we have now added the following statement in the Methods section: "For internal consistency with the marker-gene analysis, archaeal taxonomy from amplicon data is reported using the SILVA/RDP framework, whereas genome-based GTDB taxonomy is used separately for MAG analyses." (lines 594 – 596)

L60 "Archeal gene pathways"; gene pathways are not defined. Such phrasing might be clearer by

using e.g. “genetic potentials for methanogenesis, …..” and naming the pathways. A “pathway for nitrogen or sulfur” is likewise unclear.

Response: Thank you for this helpful suggestion. We agree that the phrase “archaeal gene pathways” was imprecise and that “pathways for methane, nitrogen, or sulfur” was vague. In the revised Abstract, we have replaced this wording with a clearer and more accurate description of the metagenomic result. The sentence now reads: “Archaeal genes involved in methane, nitrogen, and sulfur cycling were also enriched in plastisphere biofilms” (Lines 58 – 59). This revision avoids the undefined expression “gene pathways” while keeping the Abstract concise and more consistent with the scope of our gene-based functional inference.

L66-67 Much speculation, would avoid in the abstract.

Response: Thanks for your suggestion, and we have removed the statement in the revised manuscript to make the abstract only containing solid findings and enhance the solidness and conciseness.

L68 Please provide evidence for the “hidden …. Impacts of plastic pollution”

Response: Thank you for this comment. We agree that the previous wording was insufficiently supported and could be interpreted as too broad. In the revised Abstract, we have therefore replaced the phrase with a more precise and evidence-based statement that is directly aligned with the scope of our findings. The sentence now reads:

Our study reveals a previously overlooked yet potentially important dimension of plastic pollution impacts from an archaeal perspective, broadening our understanding of microbiology in the Anthropocene. (Lines 63 – 65)

This revised wording avoids the phrase “hidden impacts” while more accurately reflecting that our study identifies an archaeal dimension of plastisphere ecology that has been largely overlooked in previous research.

L92-100 This paragraph would profit from considering current literature from other groups and eventually also other systems (marine, freshwater, terrestrial) that addressed plastisphere effects.

Response: Thank you for this helpful suggestion. We agree that this paragraph should better situate our study within the broader plastisphere literature beyond our own previous work. In the revised manuscript, we have therefore added representative findings from other groups and from multiple systems, including studies showing that the plastisphere can support viral persistence, facilitate the dissemination of antimicrobial resistance, and, through bacteria–virus interactions, influence ecosystem carbon sequestration. At the same time, we have kept this paragraph relatively concise in order to preserve the succinct style of the Introduction, which Reviewer #2 also positively noted. We therefore chose to incorporate a brief synthesis of key findings from other studies rather than expanding this section extensively. The revised paragraph now reads as follows:

Understanding the microbiology of this novel habitat is crucial to further understand how human activities are altering the natural microbial world, and what these changes mean for ecosystems and human well-being[7, 8, 31, 32]. Research in this emerging field has intensified in recent years, yielding foundational knowledge. For example, our previous studies revealed that the plastisphere selectively assembles distinct bacterial and fungal communities, altering their diversity and functional profiles, and enriching pathogens, harmful genes, and microbes involved in organic compound degradation[10, 18]. Other studies further suggest that the plastisphere can support viral persistence[33], facilitate the dissemination of antimicrobial resistance[32, 34-36], and, through bacteria–virus interactions, affect ecosystem carbon sequestration[37]. Together, these findings highlight the potentially severe impacts of the plastisphere microbiome on ecosystem and human health[2, 7, 32]. We therefore emphasize the importance of recognizing indirect and hidden microbiological impacts of plastic pollution in order to develop effective, adaptive management strategies[7, 9, 31]. (Lines 88 – 99)

L108 There is previous evidence from literature, please add.

Response: Thank you for this suggestion. We agree that some previous plastisphere studies have reported archaeal sequences, and we have now acknowledged this explicitly in the revised Introduction. At the same time, we maintain that these previous observations do not yet provide a systematic understanding of plastisphere archaeal ecology. Most earlier studies were not designed to investigate archaea specifically, and therefore did not resolve archaeal diversity, assembly patterns, or functional potential in a dedicated way.

The present study offers several advantages that, in our view, address this gap more comprehensively. First, we used archaeal-targeted amplicon sequencing, which allowed us to specifically resolve archaeal community composition, diversity, and assembly patterns, rather than relying on bacterial-oriented surveys in which archaeal signals are often underrepresented. Second, we complemented this with archaeal-focused metagenomic functional analyses, enabling direct assessment of archaeal functional potential across multiple elemental cycles. Third, our study was based on a paired plastisphere-versus-bulk-water design, which allowed us to directly quantify how archaeal communities and functions differ between plastic-associated and surrounding free-living habitats. Fourth, the dataset spans multiple coastal habitat types, two geographic regions, and two seasons, providing broader ecological representativeness than most currently available studies. Finally, we further strengthened the study through genome-resolved MAG analyses and cross-study meta-analysis, which provided additional support for the plastisphere-specific archaeal patterns identified in our dataset.

To reflect both the existence of previous evidence and the still-limited state of archaeal-focused understanding, we revised the Introduction as follows:

Although some previous plastisphere studies have detected archaea[38, 39], a systematic understanding is still lacking regarding which archaeal taxa colonize the plastisphere, how archaeal communities respond to the plastisphere's unique conditions, and what ecological impacts may be associated with these changes. (Lines 106 – 110)

L153 Please explain the difference between taxonomic and phylogenetic diversity here.

Response: Thank you for this helpful suggestion. In the revised manuscript, we now clarify that taxonomic diversity refers to the number and relative distribution of taxa in the community, whereas phylogenetic diversity reflects the evolutionary breadth of the lineages represented. The sentence now reads:

Although taxonomic diversity (reflecting the number and relative distribution of taxa) increased, archaeal phylogenetic diversity (reflecting the evolutionary breadth of the community) decreased in the plastisphere, with a similar pattern also observed for fungi. (Lines 163 – 165)

L160 What kind of diversity does the beta-diversity refer to? Taxonomic or phylogenetic? How does this relate to findings from other plastisphere studies? Were similar or contrasting findings obtained?

Response: Thank you for this comment. Here, β-diversity refers to taxonomic β-diversity, quantified using Bray–Curtis dissimilarity and reflecting community compositional heterogeneity, rather than phylogenetic β-diversity. We have clarified this explicitly in the revised manuscript by revising the sentence to: "Likewise, archaeal taxonomic β-diversity, based on Bray–Curtis dissimilarity and reflecting community compositional heterogeneity, was greater in the plastisphere." (Lines 170 – 172)

Regarding comparison with previous plastisphere studies, we respectfully note that such comparison is not always necessary or especially meaningful for every individual pattern. Most currently available studies differ substantially from the present work in taxonomic focus, sample size, sampling design, and the use of field versus incubation approaches. In contrast, our study was designed to provide a broad, archaeal-focused, field-based assessment using paired plastisphere and bulk-water samples across multiple coastal habitat types, two regions, and two seasons. Moreover, the ecological interpretation of the elevated β-diversity is explored in detail in the community assembly section, where we show that high microscale habitat heterogeneity and strong dispersal limitation among isolated plastic "islands" likely contribute to the observed pattern. We therefore considered it more informative to clarify the metric itself here and to provide a mechanistic explanation later in the manuscript, rather than forcing repeated comparison with studies that are not directly comparable.

L165 and throughout the manuscript. Please consider current Taxonomy.

Response: Thank you for this suggestion. As explained above, archaeal community composition in this study was inferred from archaeal-targeted 16S rRNA gene amplicon data using the RDP classifier with the SILVA reference database. We therefore retained the classical SILVA-based taxonomy consistently throughout the amplicon-based community analyses, rather than reassigning taxa to recent genome-based classifications such as GTDB. We consider this to be the most appropriate and internally consistent taxonomy framework for the present dataset. To make this clearer in the manuscript, we have now added the following statement in the Methods section: "For internal consistency with the marker-gene analysis, archaeal taxonomy from

amplicon data is reported using the SILVA/RDP framework, whereas genome-based GTDB taxonomy is used separately for MAG analyses." (lines 594 – 596)

L180 How does this relate to previous findings of plastisphere studies?

Response: Thank you for this comment. We respectfully note that direct comparison with previous plastisphere studies is not always especially informative for every individual archaeal taxon identified in our dataset, particularly because archaeal-focused plastisphere studies remain very limited and most previous work was not designed to resolve archaeal biomarkers specifically. Here, our main purpose was to report a robust, data-driven result from the random-forest model, namely that *Nitrososphaera* emerged as the top archaeal biomarker distinguishing plastisphere from bulk-water communities.

Importantly, this result is also independently supported by our genome-resolved metagenomic analysis. We recovered plastisphere-derived ammonia-oxidizing archaeal (AOA)-affiliated MAGs assigned to Nitrososphaerales, and these MAGs were significantly enriched in the plastisphere relative to bulk water. Together with the nitrogen-cycling gene enrichments, this provides consistent support that AOA-related archaeal lineages are a prominent feature of the plastisphere archaeome in our dataset. More broadly, because the present study was designed as a comprehensive, archaeal-focused, field-based investigation with multiple complementary lines of evidence, we considered it more informative to emphasize the internal consistency among our community, functional, and genome-resolved results than to force point-by-point comparison with a still limited and not directly comparable literature. We performed substantial additional analyses and revisions to strengthen the manuscript; however, we chose not to add comparisons that we considered insufficiently informative or not directly comparable, in order to preserve the clarity, focus, and flow of the manuscript. Thanks for your understanding.

Accordingly, we revised the main text as follows:

Notably, the ammonia-oxidizing archaeal genus *Nitrososphaera* (phylum Crenarchaeota)[40, 41] emerged as the top biomarker, contributing ~16% of the model's predictive power and showing strong enrichment in the plastisphere (Supplementary Fig. S7). Genome-resolved metagenomic analysis further supported this pattern: among 23 recovered archaeal metagenome-assembled genome (MAGs), all five plastisphere-derived MAGs were affiliated with Nitrososphaerales,

whereas Poseidoniales MAGs were recovered exclusively from bulk water (Supplementary Note S4 and Figs. S8 and S9). (Lines 191 – 197)

The corresponding revisions in Supplementary Information are as follows:

Supplementary Note S3 Archaeal biomarkers of the plastisphere

To identify archaeal features that best distinguish the plastisphere from bulk water, we applied random-forest machine-learning models to archaeal community data at multiple taxonomic levels, including phylum, class, order, family, and genus, using the randomForest package in R.

Among these models, the genus-level model showed the highest predictive performance, explaining 85% of the variation between plastisphere and bulk-water archaeal communities (Supplementary Table S5), further supporting the marked compositional differentiation of the plastisphere from the surrounding natural habitat. Model performance and feature importance were evaluated using five-fold cross-validation with five repeated runs. We then selected the 20 archaeal taxa that contributed most strongly to model accuracy as plastisphere biomarkers (Supplementary Fig. S7a).

These biomarker taxa belonged to five phyla. Of the 20 biomarkers, 11 were enriched in the plastisphere, namely *Nitrososphaera*, *Halococcus*, *Fervidicoccus*, *Halogranum*, *Methanolobus*, *Methanogenium*, *Halovivax*, *Halomarina*, *Natrialba*, *Halostagnicola*, and *Natrinema*, whereas 9 were depleted, namely Woesearchaeota Incertae Sedis AR18, Pacearchaeota Incertae Sedis AR13, *Nitrosopumilus*, *Methanospirillum*, Woesearchaeota Incertae Sedis AR15, *Methanocorpusculum*, Woesearchaeota Incertae Sedis AR20, Woesearchaeota Incertae Sedis AR16, and *Methanoplanus* (Supplementary Fig. S7b).

Notably, *Nitrososphaera* emerged as the single most important biomarker, consistent with the genome-resolved metagenomic results showing enrichment of Nitrososphaerales MAGs in the plastisphere (Supplementary Figs. S8 and S9). Together, these results indicate that ammonia-oxidizing Nitrososphaerales-related archaea are a prominent and recurrent signature of the plastisphere archaeome.

Supplementary Note S4 Recovery and characterization of archaeal metagenome-assembled genomes

Using genome-resolved metagenomic binning, we reconstructed 23 non-redundant archaeal metagenome-assembled genomes (MAGs) that met at least medium-quality draft criteria (completeness ≥50%, contamination ≤10%; Supplementary Fig. S8)[19]. Taxonomic classification within the GTDB framework indicated two dominant archaeal lineages: Nitrososphaerales (*n* = 11; phylum Thermoproteota, class Nitrososphaeria) and Poseidoniales (*n* = 12; phylum Thermoplasmatota, class Poseidoniia). Notably, all five plastisphere-derived MAGs were affiliated with Nitrososphaerales, whereas all Poseidoniales MAGs were recovered exclusively from bulk-water metagenomes (Supplementary Fig. S8). The plastisphere signal was robust to dereplication strategy, as dereplication restricted to plastisphere-derived bins recovered the same five Nitrososphaerales MAGs. Despite comparable sequencing depth across habitats, fewer archaeal MAGs were recovered from plastisphere samples, consistent with the greater complexity and strain heterogeneity expected in surface-attached biofilms, which can reduce assembly contiguity and genome recovery[20]. This pattern is also consistent with the higher archaeal taxonomic diversity observed in the amplicon data (Fig. 1 in the main text).

Because Nitrososphaerales/Nitrososphaeria comprise ammonia-oxidizing archaea (AOA), typically characterized by genes encoding ammonia monooxygenase[21], we evaluated the nitrogen-cycling potential of recovered MAGs by annotating predicted proteins against NCycDB[22]. Among the five plastisphere-derived Nitrososphaerales MAGs, two encoded the complete ammonia monooxygenase gene set (*amoA*, *amoB*, *amoC*), supporting genomic potential for archaeal ammonia oxidation (Supplementary Fig. S8). One of these MAGs additionally contained a complete urease operon (*ureA*, *ureB*, *ureC*) (Supplementary Fig. S8), suggesting the potential to hydrolyze urea and thereby generate ammonia for nitrification[23]. Given that urea is widely available in marine systems and can support AOA growth and activity[24], this genomic configuration is consistent with the possibility that plastisphere-associated AOA exploit dissolved organic nitrogen substrates within the biofilm microenvironment.

We further detected *nirK* (copper-containing nitrite reductase) in most plastisphere Nitrososphaerales MAGs (Supplementary Fig. S8). In AOA, *nirK* has been shown to generate nitric oxide (NO) from nitrite and/or hydroxylamine-derived intermediates and is considered central to archaeal nitrogen metabolism[25]. NO-related reactions in AOA have also been linked to nitrous oxide (N_2O) formation under low-oxygen conditions[26]. Although gene presence alone does not demonstrate in situ activity, these marker genes suggest that plastisphere-associated AOA

may contribute to localized nitrification and NO/N_2O-related transformations within redox-stratified plastisphere biofilms.

In contrast, bulk-water Poseidoniales MAGs lacked *amo* genes (Supplementary Fig. S8), consistent with genome-resolved studies describing Poseidoniales (Marine Group II archaea) as predominantly aerobic (photo)heterotrophs involved in dissolved and particulate organic matter turnover, with additional lineage-specific traits including transport and degradation of organic substrates and, in some taxa, nitrate-reduction capacity[27].

Finally, metagenomic read recruitment to MAG-predicted genes showed that Nitrososphaerales MAGs were significantly more abundant in plastisphere samples than in bulk water, whereas Poseidoniales MAGs showed the opposite pattern (Wilcoxon rank-sum tests, $P < 0.001$; Supplementary Fig. S9).

Collectively, the selective recovery and enrichment of AOA-affiliated MAGs in the plastisphere, together with their encoded potential for ammonia oxidation, urea utilization, and NO-related nitrite reduction, provide genome-resolved support for our gene-centric inference that the plastisphere may act as a localized hotspot for archaeal nitrogen transformations in coastal marine ecosystems.

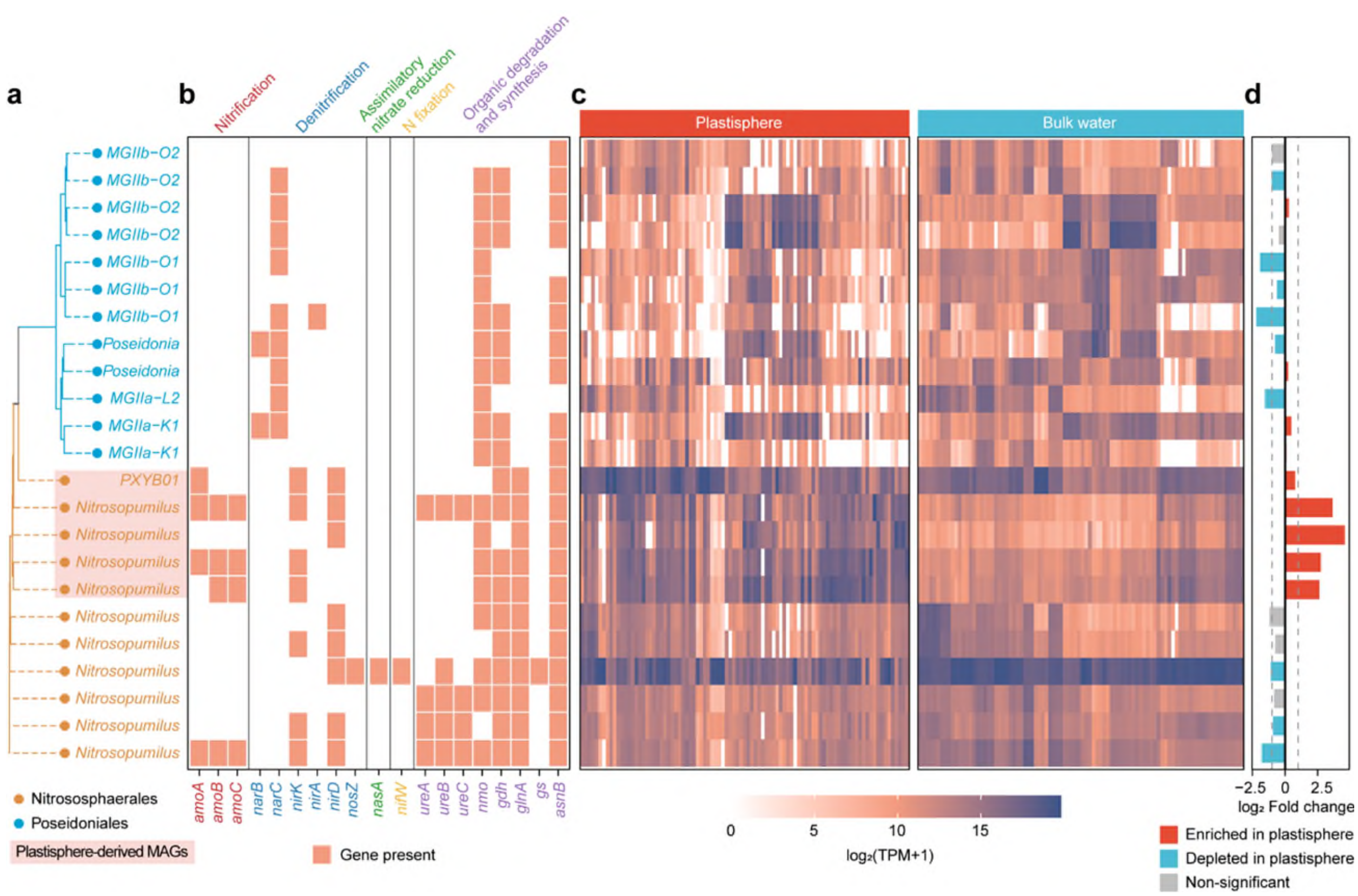

Fig. S8 Genome-resolved characterization of archaeal MAGs recovered from plastisphere and bulk-water communities. a, Maximum-likelihood phylogenetic tree of 23 archaeal MAGs inferred from ar53 marker genes. Tips are coloured by taxonomic order (orange, Nitrososphaerales; blue, Poseidoniales); plastisphere-derived MAGs are highlighted with a pink background. **b,** Presence of nitrogen-cycling genes across all 23 MAGs, grouped by functional process according to NCycDB classification. **c,** Heat map of MAG relative abundance (log_2-transformed TPM) across plastisphere and bulk water samples. **d,** Differential abundance of each MAG between plastisphere and bulk water. Red and blue bars indicate significant enrichment in plastisphere and bulk water, respectively; grey bars indicate no significant difference (Wilcoxon rank-sum test, $P < 0.05$).

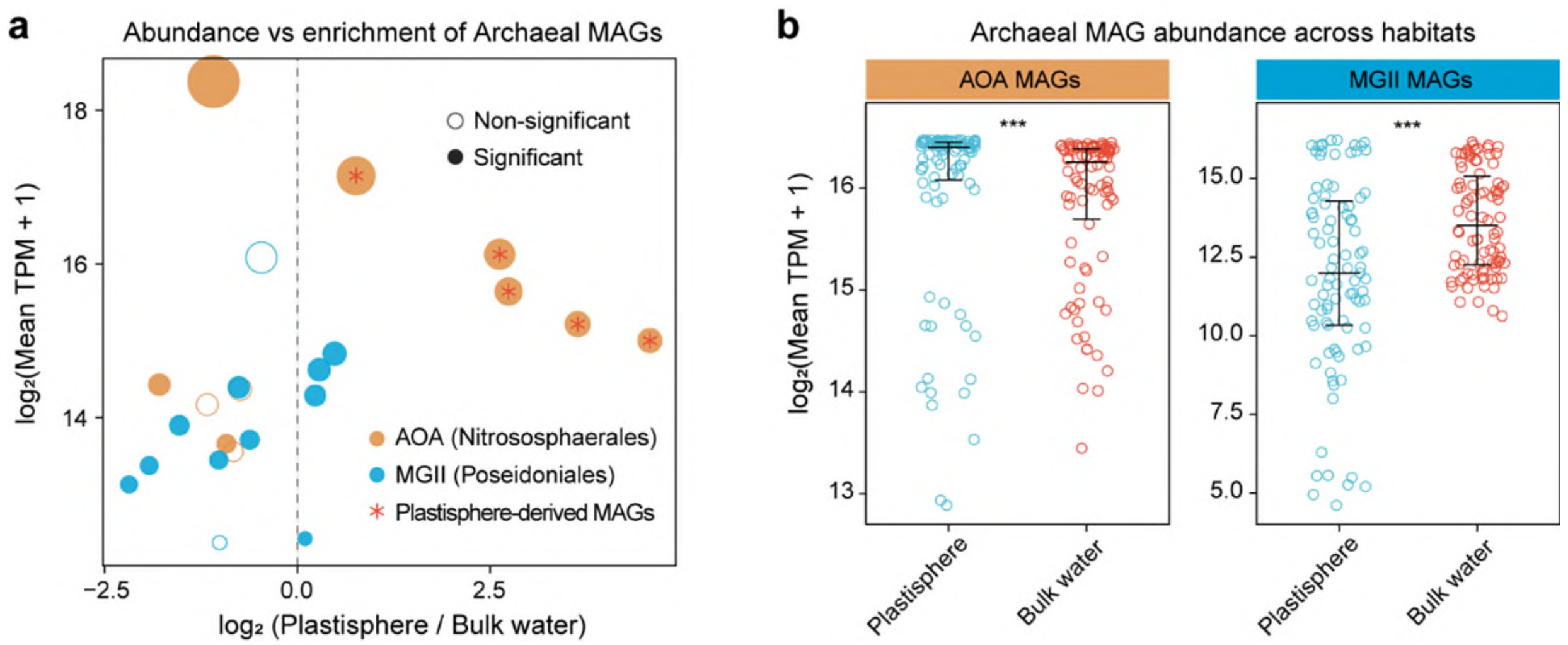

Fig. S9 Differential abundance of archaeal MAGs between plastisphere and bulk water. a, Bubble plot showing the relationship between differential abundance and mean abundance for each MAG. Bubble size represents mean total TPM, and colour indicates taxonomic affiliation (orange, Nitrososphaerales; blue, Poseidoniales). Plastisphere-derived MAGs are marked with asterisks. Filled and open symbols indicate statistically significant and non-significant differences, respectively (Wilcoxon rank-sum test, $P < 0.05$). **b,** Mean abundance of Nitrososphaerales and Poseidoniales MAGs in plastisphere and bulk-water samples. Significance was assessed using Wilcoxon rank-sum tests ($n = 82$ per group; *** $P < 0.001$).

L210-211 Please specify pathways. A pathway for methane might refer to methanogenesis or methanotrophy, one for nitrogen might refer to assimilation or dissimilation, etc.

Response: Thank you for this helpful suggestion. We agree that the original wording was too

broad, as "pathways for methane, nitrogen, sulfur, and phosphorus cycling" could refer to multiple distinct processes. In the revised manuscript, we therefore replaced this phrase with a more precise description. The sentence now reads: "The archaeal functional gene profile was significantly altered in the plastisphere relative to bulk water, with distinct differences in functional genes related to methane (CH_4), nitrogen (N), sulfur (S), and phosphorus (P) cycling (Fig. 2a; Supplementary Figs. S12 to S17)." The specific pathways are then described in the following paragraphs and corresponding Supplementary Figures.

L227-235 Highly speculative. Please provide activity/process or at least gene expression data. The genetic potential alone does not allow for conclusions on activity. I would expect that the plastisphere in coastal marine systems is largely subjected to light, thus also hosting oxygenic phototrophic Bacteria and Eukarya. Methanogens and AOM are obligate anaerobes that hardly thrive under oxic conditions.

Response: Thank you for this important comment. We agree that our metagenomic data support inference of functional potential, rather than direct evidence of activity, gene expression, or realized methane production and oxidation rates. We have therefore revised this section to avoid overinterpreting gene enrichment as evidence of active methanogenesis or AOM in situ.

At the same time, because this is a comparative study between the plastisphere and the paired surrounding bulk water, our interpretation is based on the idea that plastisphere biofilms may provide relatively oxygen-depleted microenvironments compared with the surrounding water column. This is supported by previous studies showing that plastisphere biofilms can create conditions favorable for low-oxygen processes. We now present this as a plausible mechanism rather than a demonstrated process in our dataset, and we explicitly note that the magnitude and direction of any net effect on CH_4 dynamics remain uncertain.

The revised text now reads:

A likely mechanism is that plastisphere biofilms create localized low-oxygen microenvironments relative to the surrounding water column[42, 43]. Such conditions could allow methanogenic archaea and anaerobic methanotrophs performing AOM to coexist in close proximity within the biofilm. Accordingly, the concurrent enrichment of genes associated with methanogenesis and AOM suggests the potential for coupled methane production and oxidation in plastisphere biofilms, with

possible implications for local CH_4 dynamics. Ecologically, dispersed plastic debris may therefore represent hotspots of methane turnover in coastal waters, although the magnitude and direction of any net effect remain uncertain. Related studies in soils have linked plastics to elevated CH_4 emissions via microbial shifts[44, 45], highlighting the need to test whether similar outcomes occur in marine plastisphere systems and to quantify the contribution of the plastisphere archaeome. (Lines 250 – 259)

L241 Please provide information on biofilm thickness and oxygenic phototrophs. Is there evidence that biofilms are of a size to allow for oxygen depletion?

Response: Thanks for your comment. We agree that our study did not directly measure biofilm thickness, oxygen profiles, or the abundance of oxygenic phototrophs on plastic surfaces. Our intention here was therefore not to present oxygen depletion as a direct observation from our dataset, but rather to discuss a plausible mechanism, grounded in previous plastisphere studies, that may help explain the nitrogen-cycling patterns observed in our paired plastisphere-versus-bulk-water comparison.

Importantly, this is a comparative study in which plastisphere communities are interpreted relative to the paired surrounding water column. In this context, our discussion is based on the idea that plastisphere biofilms may provide relatively oxygen-depleted microenvironments compared with the surrounding bulk water. This interpretation is supported by previous work. One *Nature Communications* study reported that biofilm formation in the plastisphere provides anoxic conditions favoring denitrifiers and that, compared with surrounding bulk water, the plastisphere showed higher denitrifying activity and N_2O production[42]. Another *Nature Communications* study further showed that plastisphere biofilms can generate steep “oxic–microoxic–hypoxic” oxygen gradients within the biofilm microenvironment[43].

We have therefore kept this discussion as a literature-based mechanistic interpretation rather than a direct result of the present study, and we have revised the wording to avoid overstatement while retaining its relevance for explaining the enrichment of nitrification- and denitrification-related genes in our plastisphere samples. The revised text now reads:

Plastisphere biofilms likely develop steep oxygen and nutrient gradients[43], allowing ammonia-oxidizing archaea to inhabit oxygenated outer layers of the biofilm, where they convert ammonia

to nitrite[26]. These may then diffuse into deeper low-O_2 zones that support denitrification[42]. In addition, plastics tend to adsorb organic matter from surrounding media[46-48], providing abundant substrate for nitrogen-transforming microorganisms. Weathered plastic surfaces also acquire negative charges that attract positively charged ions like NH_4^+ [49], potentially concentrating ammonia and further benefiting nitrifiers. These features are consistent with previous reports that the plastisphere can function as a nitrification hotspot and may also favor denitrification and N_2O production[41-43, 50]. Our genome-resolved analyses further supported this pattern: Nitrososphaerales MAGs were enriched in the plastisphere and carried key nitrogen-cycling genes, including complete *amoABC*, *ureABC*, and *nirK*, consistent with the potential for ammonia oxidation and related nitrogen transformations in plastisphere biofilms (Supplementary Note S4 and Figs. S8 and S9). Overall, the enrichment of both nitrification- and denitrification-related genes suggests that floating debris may harbor enhanced potential for nitrogen transformations, with possible implications for nitrogen removal, N_2O-related processes, water quality, the global nitrogen balance, and climate change[2] (Lines 265 – 279)

L243 Are there nitrite oxidizing Archaea producing nitrate known?

Response: Thank you for this important correction. We agree that ammonia-oxidizing archaea are associated with the oxidation of ammonia to nitrite, not nitrate. We have therefore revised the text accordingly. The sentence now reads: "Plastisphere biofilms likely develop steep oxygen and nutrient gradients[43], allowing ammonia-oxidizing archaea to inhabit oxygenated outer layers of the biofilm, where they convert ammonia to nitrite[26]." (Lines 265 – 267)

L253-258 Speculative. Are there data on activities of Archaea on plastic surfaces?

Response: Thanks for your comment. We agree that our data do not provide direct evidence of archaeal activity on plastic surfaces. Our analyses are based on community composition and metagenomic functional inference, and therefore support interpretation of functional potential, rather than direct activity, viability, or realized nitrogen transformation rates. In response, we substantially revised throughout the manuscript to avoid overstatement at the process level.

In this section, we removed the previous wording implying accelerated nitrogen removal and N_2O generation as direct outcomes, and replaced it with a more cautious interpretation centered on the enrichment of nitrification- and denitrification-related genes and genome-resolved support

from plastisphere-enriched Nitrososphaerales MAGs. The revised text now reads:

Our genome-resolved analyses further supported this pattern: Nitrososphaerales MAGs were enriched in the plastisphere and carried key nitrogen-cycling genes, including complete *amoABC*, *ureABC*, and *nirK*, consistent with the potential for ammonia oxidation and related nitrogen transformations in plastisphere biofilms (Supplementary Note S4 and Figs. S8 and S9). Overall, the enrichment of both nitrification- and denitrification-related genes suggests that floating debris may harbor enhanced potential for nitrogen transformations, with possible implications for nitrogen removal, N_2O-related processes, water quality, the global nitrogen balance, and climate change[2]. (Lines 273 – 279)

In addition, to make this limitation explicit at the manuscript level, we have added the following statement in the Conclusions and outlook section:

Nevertheless, because our analyses are based on taxonomic composition and metagenomic functional inference, direct links to viability, in situ activity, and realized biogeochemical rates remain to be established. In addition, the ecological implications highlighted here are based on relative comparisons rather than direct measurements of impact magnitude. For example, we still do not know to what extent plastic pollution contributes to microbial redistribution and invasion in the ocean, nor what magnitude of greenhouse-gas-related effects may be associated with plastisphere archaea. Further research is needed to directly link these microbial shifts to real-world ecological and health outcomes of plastic pollution, and to quantitatively assess the full risks of the plastisphere. Addressing these questions will require cross-disciplinary collaboration among researchers in environmental microbiology, environmental impact assessment, public health, and data science. Moreover, our study only considered surface waters and significant knowledge gaps remain regarding plastisphere dynamics throughout the water column and deep ocean ecosystems. Future work should also investigate the ecological mechanisms underlying plastisphere archaeal community assembly, including the roles of biofilm succession, substrate heterogeneity, and dispersal dynamics, to advance predictive understanding of how plastic pollution reshapes microbial diversity and function in marine ecosystems. (Lines 519 – 534)

This revision, in our view, better aligns the interpretation with the actual scope of the data while preserving the ecological relevance of the observed nitrogen-cycling patterns.

L278 Are such organisms feeding on sulfur or also other compounds? Please clarify.

Response: Thank you for this helpful comment. We agree that the phrase "sulfur-feeding organisms" was imprecise, as our intention was not to imply that these organisms feed specifically on sulfur compounds, but rather that they can be attracted by DMS as an infochemical. We have therefore revised the sentence for clarity as follows:

Additionally, DMS associated with plastic biofilms can act as an infochemical: organisms such as seabirds and copepods are attracted by the DMS signal to plastic debris, increasing exposure through mistaken ingestion and thereby potentially generating broader ecosystem impacts[51, 52]. (Lines 298 – 300)

L284 This statement is not supported by the data. Please tone down.

Response: Thank you for this comment. We agree that the original wording was too strong and have therefore toned down the statement accordingly. The sentence now reads: "Overall, plastisphere archaeal communities exhibit enhanced biogeochemical functional potential." (Line 312)

L390-409 Very interesting analyses. Please give the concentrations of antibiotics measured and the other environmental parameters in the supplement and cite here. Would there be a causative relationship? Are the antibiotics concentrations inhibitory/ effective for Archaea?

Response: Thank you for these helpful comments. All key data supporting the analyses, including antibiotic concentrations and environmental parameters, have previously been uploaded to GitHub (https://github.com/Changchao-Li/plastisphere_archaea), and are indicated in the manuscript. Following your suggestion, we have now added the measured environmental parameters to the Supplementary Information as Supplementary Table S2 and cited in the main text.

Regarding causality, we agree that our dataset is based on field observations and therefore does not support direct causal inference. We have accordingly interpreted these relationships as environmental associations or correlates, rather than as evidence that antibiotics directly caused the observed archaeal patterns. In the revised manuscript, we discuss a plausible ecological

explanation for these associations: One plausible explanation is that the plastisphere provides a sheltered microhabitat in which resident microorganisms are buffered, to some extent, from external chemical stress, whereas bulk-water archaeal communities are more directly exposed to surrounding conditions[9]. Under this scenario, increasing stress would be expected to reshape bulk-water communities more strongly than plastisphere communities, thereby amplifying the taxonomic and functional divergence between them. (Lines 468 – 473)

This interpretation is also consistent with previous studies showing that plastic surfaces can adsorb organic matter, support biofilm formation, and provide microhabitats that may buffer microorganisms from harsh environmental conditions. Microplastics, as a type of inert carbon[53], readily adsorb organic matter onto their surfaces and thus can create a relatively nutrient-rich, benign microenvironment for microbial colonization[29, 46, 48, 54]. Holes and cracks in the surface of microplastics provide shelter for microorganisms, and reduce their exposure to external stressors[18]. The rough, organic surface of microplastics facilitates attachment of microbial secretions such as extracellular polysaccharides, and biofilm formation[55]. These biofilms, in turn, help protect microorganisms from harsh environmental conditions[56]. Therefore, microorganisms can rapidly colonize plastic surfaces in the environment, which may enhance their survival and growth[57]. In harsh or nutrient-poor environments, such as open-oceanic systems, the plastisphere often acts as a nutrient island and thus harbors significantly higher microbial biomass compared to the surrounding environment[29, 58]. The plastisphere may also help to buffer environmental stress on microorganisms, for example, its demonstrated sheltering role during the transition from freshwater to seawater[18, 59]. The plastisphere's ability to prolong the survival and infectivity of microorganisms, including pathogens and viruses, is also well documented[7, 33, 58, 60, 61].

With respect to whether the measured antibiotic concentrations are inhibitory or effective specifically for archaea, we did not intend to make such a claim, and this question is beyond the scope of the present field-based study. Here, antibiotics are treated primarily as representative indicators of anthropogenic chemical pollution and environmental stress, rather than as archaeal-specific inhibitory agents. We have clarified this interpretation in the revised manuscript.

L427 Might this be due to a simple life style change, i.e. that sessile particle associated organisms are more tolerant to environmental disturbance/ stressors irrespective of the support? If yes, this wouldn't be a specific plastisphere effect.

Response: Thank you for this important comment. We agree that, in principle, some degree of stress buffering could be associated with a surface-attached lifestyle more generally. However, from our perspective, this does not diminish the ecological relevance of the plastisphere as a specific habitat. Plastic is a novel, anthropogenic substrate imposed on natural ecosystems, and the plastisphere is therefore not simply another naturally occurring attachment surface, but a human-created habitat that would otherwise not exist. Accordingly, all associated microbial restructuring and potential ecological impacts are also anthropogenically added to the natural environment.

This interpretation is also consistent with our previous global study of plastisphere bacterial communities, which showed that, although variation exists within plastic samples, the plastisphere nevertheless represents a distinct microbial habitat with characteristic ecological patterns and potential implications[10]. In other words, even if some mechanisms are shared more broadly by surface-attached lifestyles, plastic still creates a special and ecologically meaningful habitat signal at the community level.

To address this concern directly in the present study, we performed an additional meta-analysis of published marine metagenomic datasets containing both plastisphere samples and non-plastic solid-surface samples. The results showed that plastisphere archaeal communities were consistently distinct from those on other solid substrates at both taxonomic and functional levels. We therefore interpret the archaeal patterns observed here not as a generic consequence of sessile particle-associated growth alone, but as evidence that plastic represents a distinct habitat for archaeal communities.

At the same time, we agree that some mechanisms, such as surface-associated buffering from environmental stress, may overlap with broader biofilm-associated lifestyles. Our point is not that every individual feature of the plastisphere is exclusive to plastics, but that plastic introduces a widespread, persistent, and anthropogenic habitat whose combined properties generate a distinct archaeal ecological signal and associated potential impacts that should not exist in natural systems in the absence of plastic pollution.

The added analyses, methods, results, and figures have been incorporated into the revised manuscript and Supplementary Information as follows:

To assess whether these compositional patterns reflect a plastic-specific archaeal signal rather than a general consequence of surface attachment, we performed a meta-analysis of published metagenomic datasets that included both plastisphere and non-plastic solid surfaces. Across studies, plastisphere archaeal communities remained compositionally distinct from those on other solid substrates (Stouffer weighted *Z*-test: $P < 0.01$; Supplementary Notes S5 and S6 and Figs. S10a and S11a), indicating that the observed archaeal community patterns are not merely a generic biofilm effect. (Lines 199 – 205 in the main text)

Consistent with the taxonomic meta-analysis above, a parallel analysis further showed that, despite considerable heterogeneity in study design, combined evidence supported the distinctiveness of plastisphere archaeal functional gene profiles relative to non-plastic substrates (Stouffer weighted *Z*-test: $Z = 2.35$, $P < 0.01$; Supplementary Notes S5 and S6 and Figs. S10b and S11b), suggesting that these functional patterns likewise reflect substrate-specific archaeal responses rather than a general surface-associated biofilm effect. (Lines 305 – 310 in the main text)

Supplementary Note S5 Plastisphere as an archaeal habitat distinct from other solid surfaces

To determine whether the distinctive archaeal assemblages observed on plastics represent a genuine substrate-specific signal rather than a general biofilm effect, we compared plastisphere archaeal communities with those on other solid surfaces, including wood, ceramic, metal, and cardboard, across five independent studies with sufficient replication, using both taxonomic composition and functional gene profiles as response variables.

For taxonomic composition (Supplementary Fig. S10a), four of the five studies showed greater between-group than within-group Bray–Curtis dissimilarity, indicating that plastisphere archaeal communities were generally more dissimilar from other-surface communities than they were internally variable. Two studies, wos_2 and wos_134, reached individual significance, whereas the others showed positive but non-significant trends, likely owing to limited within-study sample sizes. When combined across all five studies, Stouffer's weighted *Z*-test yielded $Z = 2.385$, $P < 0.01$, confirming consistent compositional separation of plastisphere archaeal communities from those on other solid surfaces across independent datasets.

A parallel pattern was observed for functional gene profiles (Supplementary Fig. S10b). Again, four of the five studies showed greater between-group than within-group Bray–Curtis dissimilarity, indicating that plastisphere samples harbored archaeal functional profiles distinct from those on other solid substrates. The combined Stouffer Z-test yielded $Z = 2.351$, $P < 0.01$, indicating that the functional distinctiveness of plastisphere archaeal communities was as consistent and statistically robust as their compositional distinctiveness.

Together, these results demonstrate that plastisphere archaeal communities differ from those on other solid surfaces at both taxonomic and functional levels, providing evidence that plastic substrates constitute a genuinely distinct archaeal habitat rather than simply another attachment surface. The convergence of compositional and functional signals across independent studies with diverse reference surfaces further supports the robustness of this conclusion.

Supplementary Note S6 Archaeome dissimilarity patterns across polymer types and substrate types

To evaluate whether polymer type structures plastisphere archaeal communities, we compared three categories of Bray–Curtis dissimilarity across four independent studies that included multiple polymer types and non-plastic reference substrates with known polymer identity (wos_50, wos_85, wos_2, and wos_134): within-polymer, between-polymer, and plastic-versus-non-plastic dissimilarity. Analyses were conducted for both archaeal taxonomic composition and functional gene profiles (Supplementary Fig. S11).

Overall, results were inconsistent across studies. In some datasets, plastic-versus-non-plastic dissimilarity exceeded between-polymer dissimilarity, suggesting that substrate identity explained more variation than polymer chemistry. In others, this pattern was not observed, and similar inconsistency was found for functional gene profiles. We attribute this lack of consistency primarily to the limited size and scope of the available datasets, including low replication per polymer type, narrow polymer diversity, sparse non-plastic reference groups, and the restricted ecological representativeness of incubation-based experiments.

These results indicate that currently available public datasets are insufficient to robustly disentangle polymer-type effects from broader substrate-identity effects in plastisphere archaeal communities. In contrast, the present study was designed to resolve the broader plastisphere signal under field conditions using a much larger set of paired plastisphere and ambient seawater

samples across diverse coastal environments. We therefore interpret the consistent taxonomic and functional separation observed in our dataset as evidence of a generalizable plastisphere-specific archaeal signal, while recognizing that polymer-specific effects remain an important question for future work.

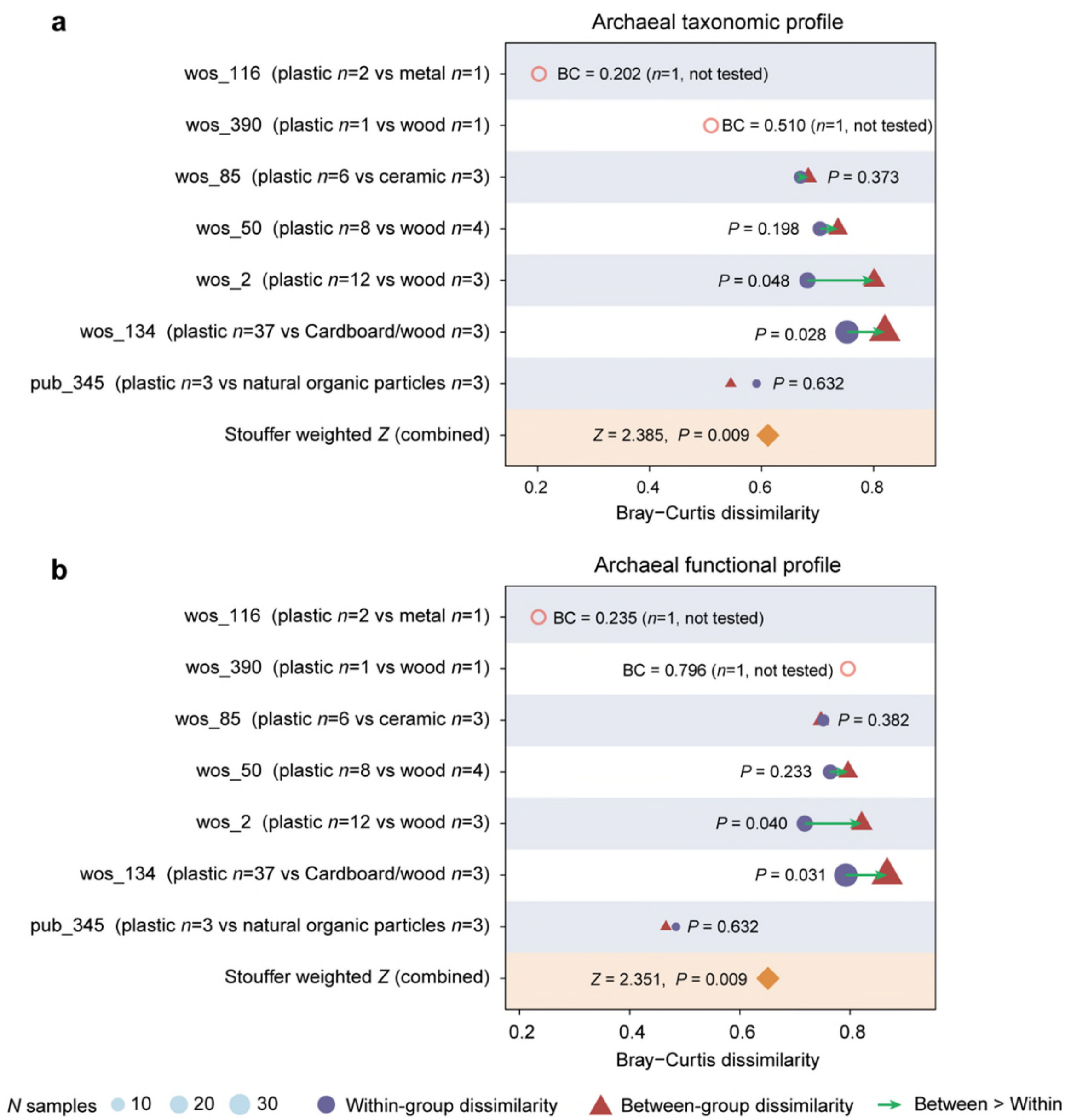

Fig. S10 Plastisphere archaeal communities are compositionally and functionally distinct from those on other solid surfaces. a, Taxonomic composition. **b,** Functional gene profiles. In both panels, red triangles indicate mean between-group Bray–Curtis dissimilarity (plastisphere versus other solid surfaces), and blue circles indicate mean within-group Bray–Curtis dissimilarity. Green arrows indicate studies in which between-group dissimilarity exceeded within-group

dissimilarity. Point size is proportional to total sample size. For wos_116 and wos_390, within-group dissimilarity could not be calculated because of insufficient replication, and only between-group dissimilarity is shown descriptively (open circles). Statistical significance within each study was assessed using permutation tests, with the number of permutations limited to the total number of possible label arrangements when this was <999. Combined evidence across the five testable studies was evaluated using Stouffer's weighted *Z*-method, weighting each study by the square root of total sample size.

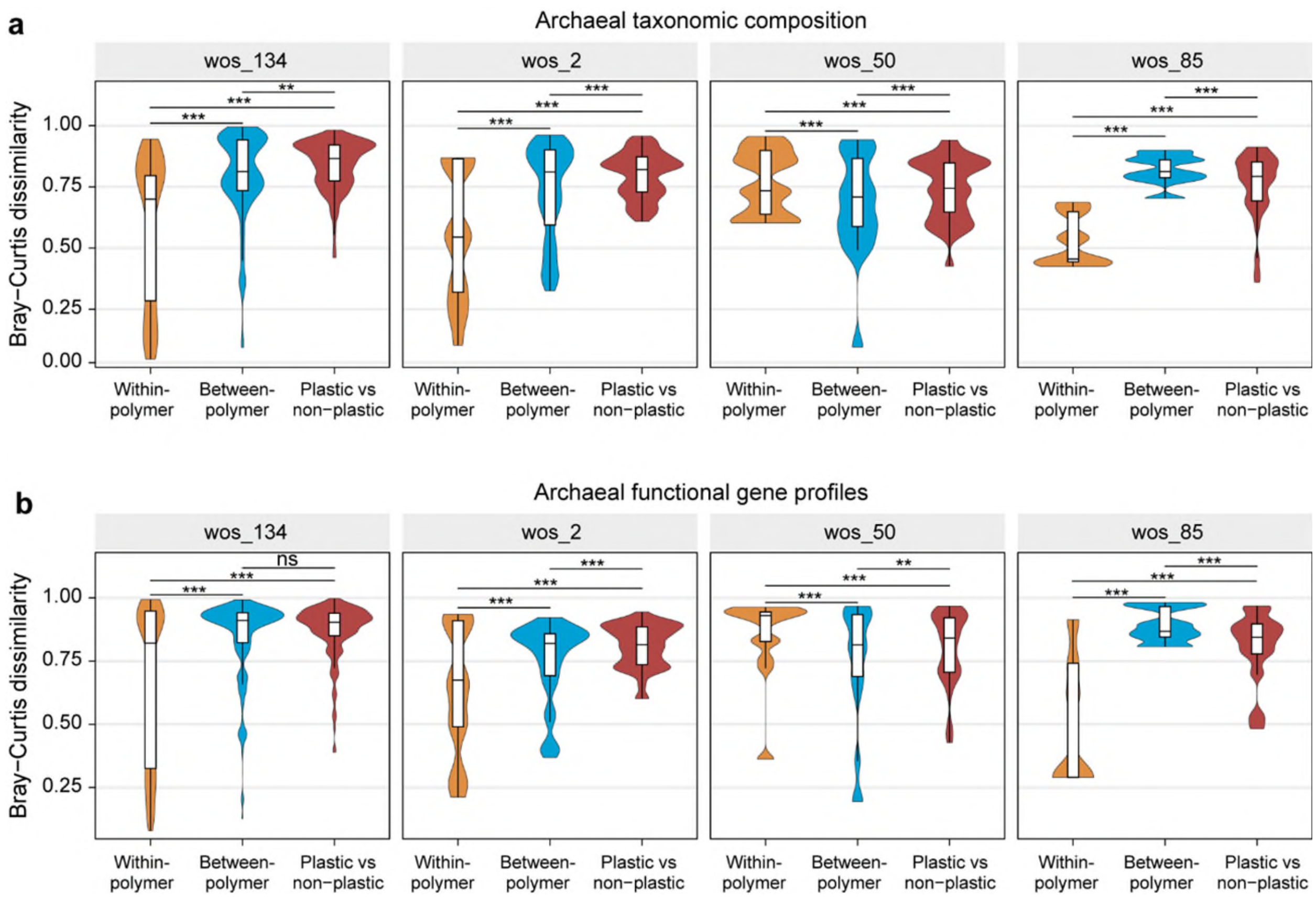

Fig. S11 Comparison of archaeal community dissimilarity across polymer types and substrate types. a, Taxonomic composition based on species-level GTDB annotation. **b,** Functional gene profiles based on combined archaeal carbon, nitrogen, phosphorus, sulfur, and methane cycling genes. In each panel, three categories of pairwise Bray–Curtis dissimilarity are shown across four independent studies: within-polymer, between-polymer, and plastic-versus-non-plastic. Each distribution comprises 999 bootstrap-sampled pairwise distances to ensure equal sample sizes across categories despite group imbalance. The horizontal line within each box indicates the median. Statistical significance was assessed using Wilcoxon rank-sum tests (*** $P < 0.001$, ** $P < 0.01$, ns = non-significant).

L506 Cells were removed from plastic particles. Was the community structure of removed cells representative of the attached microbes? In a quantitative way, was the majority of cells removed?

Response: Thank you for this important methodological question. We used a membrane-filtration-based DNA extraction workflow in which each plastic debris sample was sonicated multiple times in sterile 1× PBS on ice to detach microorganisms from particle surfaces and pores, and the PBS wash was then filtered through a 0.22 μm membrane to collect the cells[62]. This is an established approach for recovering surface-attached microbial communities from plastic particles.

Although we did not directly quantify detachment efficiency and therefore cannot state whether the majority of attached cells were removed, rarefaction analyses showed that the sequencing depth and sample size were sufficient to capture the dominant microbial diversity patterns in both plastisphere and bulk-water samples (Supplementary Fig. S22). Additional sequencing depth or sample number did not substantially increase observed richness. We therefore consider the recovered communities adequate for the comparative analyses performed in this study.

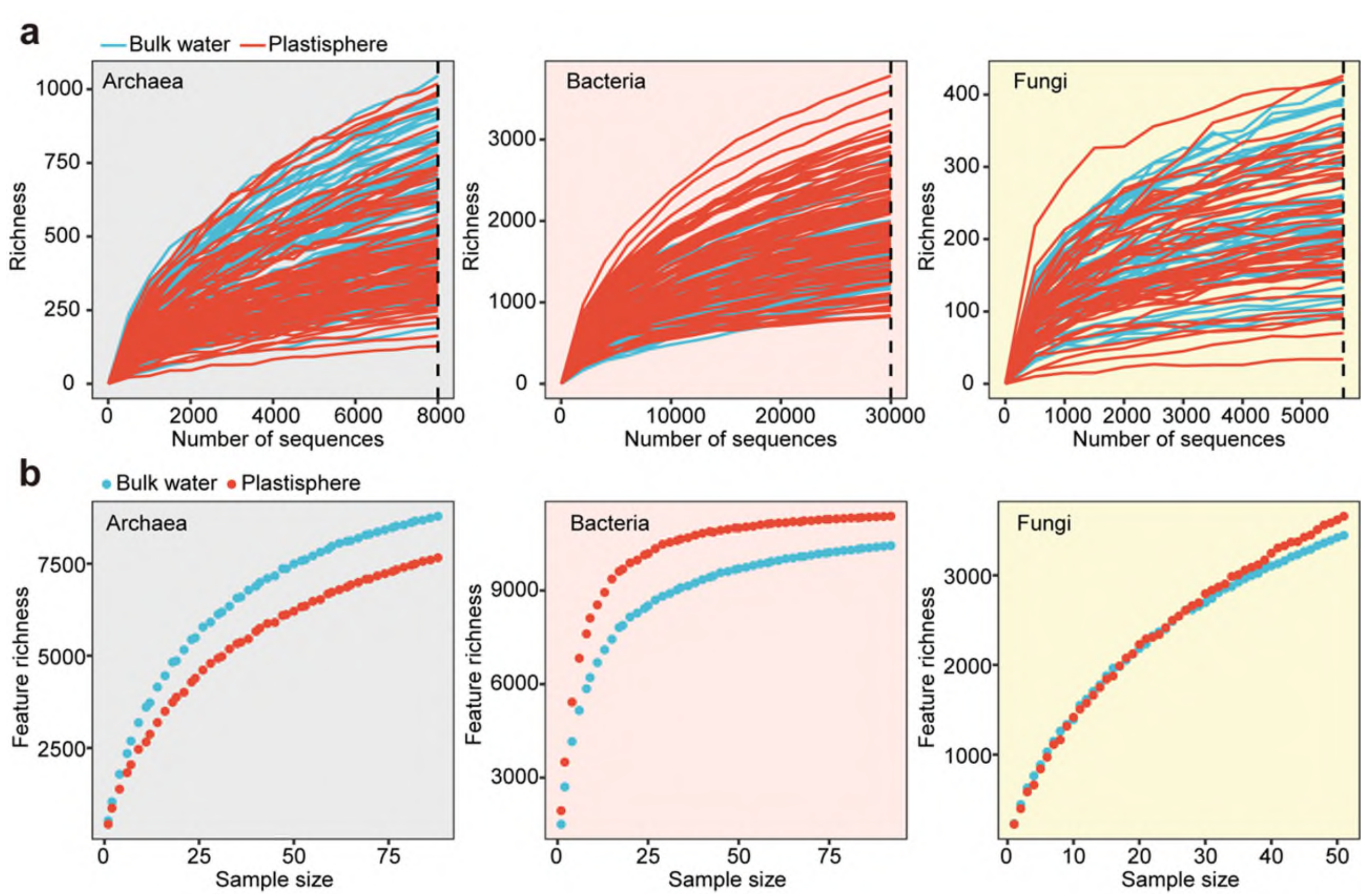

Fig. S22 Rarefaction curves. a, The number of archaeal, bacterial and fungal ASVs with the increasing number of sequences (the sequencing depth). **b,** Accumulation curves of the number of archaeal, bacterial and fungal ASVs with an increasing number of samples (the sample size). These curves flatten out at the final sequence depth (8,000 for archaea, 30,000 for bacteria, and 5,700 for fungi) and sample size (n = 88 per archaeal group, n = 92 per bacterial group and n = 51 per fungal group) chosen for this study, indicating that our samples could capture most of microbial members from the plastisphere and bulk water and thus were sufficient for the comparative analyses.

L565-565 What was the likelihood of a correct assignment of genes/ sequences to Archaea?

Response: Thank you for this important question. In our functional metagenomic analysis, archaeal assignment was performed before biogeochemical gene annotation. Specifically, taxonomic annotation for each gene in the non-redundant metagenomic gene catalog was first determined against GTDB. We then extracted only the genes annotated as archaeal-affiliated, together with their corresponding abundance values, to generate an archaeal-only gene catalog and abundance table. The subsequent annotation against CCycDB, MCycDB, NCycDB, SCycDB, and PCycDB was performed only on this archaeal-only gene set, rather than on the full metagenomic catalog. This taxonomy-first, function-second workflow is a widely used and reasonable strategy in metagenomic functional analysis for reducing the risk of assigning non-archaeal genes to the archaeal dataset. In addition, the parameter settings used for functional gene matching follow commonly adopted standards in metagenomic functional analyses. We therefore consider both the workflow and the annotation criteria appropriate for comparative analysis of archaeal functional potential.

More importantly, our study does not rely on the interpretation of any single gene or single taxon in isolation. Rather, we focus on comparative differences in the overall functional potential of plastisphere versus bulk-water archaeal communities. In this comparative context, the key objective is to identify robust shifts in broader functional profiles, rather than to claim absolute certainty for the exact taxonomic identity or exact functional assignment of every individual sequence. This is also a general feature of metagenomic analysis: it provides broad, cultivation-independent insight into community composition and functional potential, but it does not guarantee with complete certainty that every inferred taxon is truly present or that every inferred function is realized in situ. We have therefore interpreted these results at the level of comparative

functional potential and have explicitly acknowledged these limitations in the manuscript. The related revised part in the Conclusion and outlook section is as follows:

Nevertheless, because our analyses are based on taxonomic composition and metagenomic functional inference, direct links to viability, in situ activity, and realized biogeochemical rates remain to be established. In addition, the ecological implications highlighted here are based on relative comparisons rather than direct measurements of impact magnitude. For example, we still do not know to what extent plastic pollution contributes to microbial redistribution and invasion in the ocean, nor what magnitude of greenhouse-gas-related effects may be associated with plastisphere archaea. Further research is needed to directly link these microbial shifts to real-world ecological and health outcomes of plastic pollution, and to quantitatively assess the full risks of the plastisphere. Addressing these questions will require cross-disciplinary collaboration among researchers in environmental microbiology, environmental impact assessment, public health, and data science. Moreover, our study only considered surface waters and significant knowledge gaps remain regarding plastisphere dynamics throughout the water column and deep ocean ecosystems. Future work should also investigate the ecological mechanisms underlying plastisphere archaeal community assembly, including the roles of biofilm succession, substrate heterogeneity, and dispersal dynamics, to advance predictive understanding of how plastic pollution reshapes microbial diversity and function in marine ecosystems. (Lines 519 – 534)

References

1. Huang, W.; Xia, X., Element cycling with micro(nano)plastics. *Science* **2024,** *385*, (6712), 933-935.
2. Liu, X.; Wei, W.; Chen, Z.; Wu, L.; Duan, H.; Zheng, M.; Wang, D.; Ni, B.-J., The threats of micro- and nanoplastics to aquatic ecosystems and water health. *Nature Water* **2025,** *3*, (7), 764-781.
3. Zhang, L.; Wang, C.; Xie, B., Microplastics and soil greenhouse gas emissions: A critical reflection on meta-analyses. *Environ. Sci. Technol.* **2025**.
4. Bergmann, M.; Arp, H. P. H.; Carney Almroth, B.; Cowger, W.; Eriksen, M.; Dey, T.; Gündoğdu, S.; Helm, R. R.; Krieger, A.; Syberg, K.; Tekman, M. B.; Thompson, R. C.; Villarrubia-Gómez, P.; Warrier, A. K.; Farrelly, T., Moving from symptom management to upstream plastics prevention: The fallacy of plastic cleanup technology. *One Earth* **2023,** *6*, (11), 1439-1442.
5. Pottinger, A. S.; Geyer, R.; Biyani, N.; Martinez, C. C.; Nathan, N.; Morse, M. R.; Liu, C.; Hu, S.; de Bruyn, M.; Boettiger, C.; Baker, E.; McCauley, D. J., Pathways to reduce global plastic waste mismanagement and greenhouse gas emissions by 2050. *Science* **2024,** *386*, (6726), 1168-1173.
6. Papp, A.; Oremus, K. L., Plastic bag bans and fees reduce harmful bag litter on shorelines. *Science 388*, (6753), eadp9274.
7. Li, C.; Liu, J.; Rillig, M. C.; Bank, M. S.; Fantke, P.; Zhu, D.; Zhu, Y.-G.; Jin, L. N., What harmful microbes are lurking in the world's 7 billion tonnes of plastic waste? *Nature* **2024,** *634*, (8032), 30-32.
8. Haney, J.; Rochman, C. M., Plastic pollution has the potential to alter ecological and evolutionary processes in aquatic ecosystems. *Nature Ecology & Evolution* **2025,** *9*, (5), 762-768.
9. Li, C.; Jin, L. N.; Bank, M. S.; Fan, C.; Gillings, M. R.; Zhao, T.; Han, Y.; Chen, T.; Gao, M.; Zhu, D.; Chen, Q.; Zhu, G.; Wang, J.; Wang, L.; Liu, J.; Yuan, X.; Huang, Q.; Wang, X.; Jahnke, A.; Brahney, J.; Allen, S.; Arp, H. P. H.; Oberbeckmann, S.; Bergmann, M.; Pointing, S. B.; Zhang, D.; Rillig, M. C., Potential planetary health impacts of the airborne plastisphere. *One Earth* **2025,** *8*, (10), 101446.
10. Li, C.; Gillings, M. R.; Zhang, C.; Chen, Q.; Zhu, D.; Wang, J.; Zhao, K.; Xu, Q.; Leung, P. H.; Li, X.; Liu, J.; Jin, L., Ecology and risks of the global plastisphere as a newly expanding microbial habitat. *The Innovation* **2024,** *5*, (1), 100543.

11. Zhang, X.; Zhang, C.; Liu, Y.; Zhang, R.; Li, M., Non-negligible roles of archaea in coastal carbon biogeochemical cycling. *Trends Microbiol.* **2023,** *31*, (6), 586-600.
12. Mouillot, D.; Bellwood, D. R.; Baraloto, C.; Chave, J.; Galzin, R.; Harmelin-Vivien, M.; Kulbicki, M.; Lavergne, S.; Lavorel, S.; Mouquet, N.; Paine, C. E. T.; Renaud, J.; Thuiller, W., Rare species support vulnerable functions in high-diversity ecosystems. *PLoS Biol.* **2013,** *11*, (5), e1001569.
13. Lynch, M. D. J.; Neufeld, J. D., Ecology and exploration of the rare biosphere. *Nat. Rev. Microbiol.* **2015,** *13*, (4), 217-229.
14. Wang, B.; Qin, W.; Ren, Y.; Zhou, X.; Jung, M.-Y.; Han, P.; Eloe-Fadrosh, E. A.; Li, M.; Zheng, Y.; Lu, L.; Yan, X.; Ji, J.; Liu, Y.; Liu, L.; Heiner, C.; Hall, R.; Martens-Habbena, W.; Herbold, C. W.; Rhee, S.-k.; Bartlett, D. H.; Huang, L.; Ingalls, A. E.; Wagner, M.; Stahl, D. A.; Jia, Z., Expansion of Thaumarchaeota habitat range is correlated with horizontal transfer of ATPase operons. *ISME J.* **2019,** *13*, (12), 3067-3079.
15. Borrel, G.; Brugère, J.-F.; Gribaldo, S.; Schmitz, R. A.; Moissl-Eichinger, C., The host-associated archaeome. *Nat. Rev. Microbiol.* **2020,** *18*, (11), 622-636.
16. Lennon, J. T.; Jones, S. E., Microbial seed banks: the ecological and evolutionary implications of dormancy. *Nat. Rev. Microbiol.* **2011,** *9*, (2), 119-130.
17. Li, C.; Jin, L.; Zhang, C.; Li, S.; Zhou, T.; Hua, Z.; Wang, L.; Ji, S.; Wang, Y.; Gan, Y.; Liu, J., Destabilized microbial networks with distinct performances of abundant and rare biospheres in maintaining networks under increasing salinity stress. *iMeta* **2023,** *2*, (1), e79.
18. Li, C.; Wang, L.; Ji, S.; Chang, M.; Wang, L.; Gan, Y.; Liu, J., The ecology of the plastisphere: Microbial composition, function, assembly, and network in the freshwater and seawater ecosystems. *Water Research* **2021,** *202*, 117428.
19. Bowers, R. M.; Kyrpides, N. C.; Stepanauskas, R.; Harmon-Smith, M.; Doud, D.; Reddy, T. B. K.; Schulz, F.; Jarett, J.; Rivers, A. R.; Eloe-Fadrosh, E. A.; Tringe, S. G.; Ivanova, N. N.; Copeland, A.; Clum, A.; Becraft, E. D.; Malmstrom, R. R.; Birren, B.; Podar, M.; Bork, P.; Weinstock, G. M.; Garrity, G. M.; Dodsworth, J. A.; Yooseph, S.; Sutton, G.; Glöckner, F. O.; Gilbert, J. A.; Nelson, W. C.; Hallam, S. J.; Jungbluth, S. P.; Ettema, T. J. G.; Tighe, S.; Konstantinidis, K. T.; Liu, W.-T.; Baker, B. J.; Rattei, T.; Eisen, J. A.; Hedlund, B.; McMahon, K. D.; Fierer, N.; Knight, R.; Finn, R.; Cochrane, G.; Karsch-Mizrachi, I.; Tyson, G. W.; Rinke, C.; Kyrpides, N. C.; Schriml, L.; Garrity, G. M.; Hugenholtz, P.; Sutton, G.; Yilmaz, P.; Meyer, F.; Glöckner, F. O.; Gilbert, J. A.; Knight, R.; Finn, R.; Cochrane, G.; Karsch-Mizrachi, I.; Lapidus, A.; Meyer, F.; Yilmaz, P.; Parks, D. H.; Murat Eren, A.; Schriml, L.; Banfield, J. F.; Hugenholtz, P.; Woyke, T.; The Genome Standards, C., Minimum information about a single

amplified genome (MISAG) and a metagenome-assembled genome (MIMAG) of bacteria and archaea. *Nature Biotechnology* **2017,** *35*, (8), 725-731.

20. Rocha, U.; Kasmanas, J. C.; Toscan, R.; Sanches, D. S.; Magnusdottir, S.; Saraiva, J. P., Simulation of 69 microbial communities indicates sequencing depth and false positives are major drivers of bias in prokaryotic metagenome-assembled genome recovery. *PLoS Comput. Biol.* **2024,** *20*, (10), e1012530.
21. Hatzenpichler, R., Diversity, physiology, and niche differentiation of ammonia-oxidizing archaea. *Appl. Environ. Microbiol.* **2012,** *78*, (21), 7501-7510.
22. Tu, Q.; Lin, L.; Cheng, L.; Deng, Y.; He, Z., NCycDB: a curated integrative database for fast and accurate metagenomic profiling of nitrogen cycling genes. *Bioinformatics* **2019,** *35*, (6), 1040-1048.
23. Tourna, M.; Stieglmeier, M.; Spang, A.; Könneke, M.; Schintlmeister, A.; Urich, T.; Engel, M.; Schloter, M.; Wagner, M.; Richter, A.; Schleper, C., *Nitrososphaera viennensis*, an ammonia oxidizing archaeon from soil. *Proceedings of the National Academy of Sciences* **2011,** *108*, (20), 8420-8425.
24. Stuehrenberg, J.; Kitzinger, K.; von Arx, J. N.; Graf, J. S.; Lavik, G.; Littmann, S.; Milucka, J.; Orsi, W. D.; Schorn, S.; Speth, D. R.; Vuillemin, A.; Wu, S.; Marchant, H. K.; Kuypers, M. M. M., Urea use drives niche separation between dominant marine ammonia oxidizing archaea. *Nat. Commun.* **2025,** *16*, (1), 10946.
25. Kobayashi, S.; Hira, D.; Yoshida, K.; Toyofuku, M.; Shida, Y.; Ogasawara, W.; Yamaguchi, T.; Araki, N.; Oshiki, M., Nitric oxide production from nitrite reduction and hydroxylamine oxidation by copper-containing dissimilatory nitrite reductase (NirK) from the aerobic ammonia-oxidizing archaeon, *Nitrososphaera viennensis*. *Microbes and Environments* **2018,** *33*, (4), 428-434.
26. Wu, L.; Chen, X.; Wei, W.; Liu, Y.; Wang, D.; Ni, B.-J., A critical review on nitrous oxide production by ammonia-oxidizing archaea. *Environ. Sci. Technol.* **2020,** *54*, (15), 9175-9190.
27. Rinke, C.; Rubino, F.; Messer, L. F.; Youssef, N.; Parks, D. H.; Chuvochina, M.; Brown, M.; Jeffries, T.; Tyson, G. W.; Seymour, J. R.; Hugenholtz, P., A phylogenomic and ecological analysis of the globally abundant Marine Group II archaea (*Ca.* Poseidoniales ord. nov.). *ISME J.* **2019,** *13*, (3), 663-675.
28. Zhao, S.; Zettler, E. R.; Amaral-Zettler, L. A.; Mincer, T. J., Microbial carrying capacity and carbon biomass of plastic marine debris. *ISME J.* **2021,** *15*, (1), 67-77.
29. Amaral-Zettler, L. A.; Zettler, E. R.; Mincer, T. J., Ecology of the plastisphere. *Nat. Rev. Microbiol.* **2020,** *18*, (3), 139-151.

30. Zhang, Y.; Ning, D.; Wu, L.; Yuan, M. M.; Zhou, X.; Guo, X.; Hu, Y.; Jian, S.; Yang, Z.; Han, S.; Feng, J.; Kuang, J.; Cornell, C. R.; Bates, C. T.; Fan, Y.; Michael, J. P.; Ouyang, Y.; Guo, J.; Gao, Z.; Shi, Z.; Xiao, N.; Fu, Y.; Zhou, A.; Wu, L.; Liu, X.; Yang, Y.; Tiedje, J. M.; Zhou, J., Experimental warming leads to convergent succession of grassland archaeal community. *Nature Climate Change* **2023,** *13*, (6), 561-569.
31. Rillig, M. C.; Li, C.; Jin, L. N.; Kim, S. W., Understanding the soil plastisphere and its environmental impacts. *One Earth* **2024,** *7*, (12), 2095-2098.
32. Ormsby, M. J.; Quilliam, R. S., The aquatic plastisphere: ecology, pathogen dissemination and antimicrobial resistance. *Nat. Rev. Microbiol.* **2026**.
33. Lu, J.; Yu, Z.; Ngiam, L.; Guo, J., Microplastics as potential carriers of viruses could prolong virus survival and infectivity. *Water Research* **2022,** *225*, 119115.
34. Zhu, D.; Ma, J.; Li, G.; Rillig, M. C.; Zhu, Y.-G., Soil plastispheres as hotspots of antibiotic resistance genes and potential pathogens. *ISME J.* **2022,** *16*, (2), 521-532.
35. Bank, M. S.; Ok, Y. S.; Swarzenski, P. W., Microplastic's role in antibiotic resistance. *Science* **2020,** *369*, (6509), 1315-1315.
36. Zadjelovic, V.; Wright, R. J.; Borsetto, C.; Quartey, J.; Cairns, T. N.; Langille, M. G. I.; Wellington, E. M. H.; Christie-Oleza, J. A., Microbial hitchhikers harbouring antimicrobial-resistance genes in the riverine plastisphere. *Microbiome* **2023,** *11*, (1), 225.
37. Wang, L.; Lin, D.; Xiao, K.-Q.; Ma, L.-J.; Fu, Y.-M.; Huo, Y.-X.; Liu, Y.; Ye, M.; Sun, M.-M.; Zhu, D.; Rillig, M. C.; Zhu, Y.-G., Soil viral–host interactions regulate microplastic-dependent carbon storage. *Proceedings of the National Academy of Sciences* **2024,** *121*, (45), e2413245121.
38. Agostini, L.; Moreira, J. C. F.; Bendia, A. G.; Kmit, M. C. P.; Waters, L. G.; Santana, M. F. M.; Sumida, P. Y. G.; Turra, A.; Pellizari, V. H., Deep-sea plastisphere: Long-term colonization by plastic-associated bacterial and archaeal communities in the Southwest Atlantic Ocean. *Sci. Total Environ.* **2021,** *793*, 148335.
39. Wang, Q.; Zheng, G.; Ni, L.; Wang, H.; Li, W.; Guo, P.; Wang, Y.; Zheng, D.; Wu, J.; Zhang, D., Colonization characteristics and dynamic transition of archaea communities on polyethylene and polypropylene microplastics in the sediments of mangrove ecosystems. *Journal of Hazardous Materials* **2024,** *471*, 134343.
40. Ren, M.; Wang, J., Phylogenetic divergence and adaptation of *Nitrososphaeria* across lake depths and freshwater ecosystems. *ISME J.* **2022,** *16*, (6), 1491-1501.

41. Yang, Q.; Zhong, Y.; Feng, S.-w.; Wen, P.; Wang, H.; Wu, J.; Yang, S.; Liang, J.-L.; Li, D.; Yang, Q.; Tam, N. F. Y.; Peng, P. a., Temporal enrichment of comammox *Nitrospira* and *Ca.* Nitrosocosmicus in a coastal plastisphere. *ISME J.* **2024,** *18*, (1), wrae186.

42. Su, X.; Yang, L.; Yang, K.; Tang, Y.; Wen, T.; Wang, Y.; Rillig, M. C.; Rohe, L.; Pan, J.; Li, H.; Zhu, Y.-g., Estuarine plastisphere as an overlooked source of N_2O production. *Nat. Commun.* **2022,** *13*, (1), 3884.

43. Su, X.; Huang, X.; Zhang, Y.; Yang, L.; Wen, T.; Yang, X.; Zhu, G.; Zhang, J.; Tang, Y.; Li, Z.; Ding, J.; Li, R.; Pan, J.; Chen, X.; Huang, F.; Rillig, M. C.; Zhu, Y.-g., Nitrifying niche in estuaries is expanded by the plastisphere. *Nat. Commun.* **2024,** *15*, (1), 5866.

44. He, Z.; Hou, Y.; Li, Y.; Bei, Q.; Li, X.; Zhu, Y.-G.; Liesack, W.; Rillig, M. C.; Peng, J., Increased methane production associated with community shifts towards *Methanocella* in paddy soils with the presence of nanoplastics. *Microbiome* **2024,** *12*, (1), 259.

45. Su, P.; Bu, N.; Liu, X.; Sun, Q.; Wang, J.; Zhang, X.; Xiang, T.; Chu, K.; Zhang, Z.; Cao, X.; Li, Z., Stimulated soil CO_2 and CH_4 emissions by microplastics: A hierarchical perspective. *Soil Biology and Biochemistry* **2024,** *194*, 109425.

46. Wang, J.; Tao, J.; Ji, J.; Wu, M.; Sun, Y.; Li, J.; Gan, J., Use of a dual-labeled bioaccumulation method to quantify microplastic vector effects for hydrophobic organic contaminants in soil. *ACS Environmental Au* **2023,** *3*, (4), 233-241.

47. Wang, L.-C.; Lin, J. C.-T.; Ye, J.-A.; Lim, Y. C.; Chen, C.-W.; Dong, C.-D.; Liu, T.-K., Enrichment of persistent organic pollutants in microplastics from coastal waters. *Environ. Sci. Technol.* **2024,** *58*, (50), 22391-22404.

48. Sheridan, E. A.; Fonvielle, J. A.; Cottingham, S.; Zhang, Y.; Dittmar, T.; Aldridge, D. C.; Tanentzap, A. J., Plastic pollution fosters more microbial growth in lakes than natural organic matter. *Nat. Commun.* **2022,** *13*, (1), 4175.

49. Yang, X.; Zhang, L.; Chen, Y.; He, Q.; Liu, T.; Zhang, G.; Yuan, L.; Peng, H.; Wang, H.; Ju, F., Micro(nano)plastic size and concentration co-differentiate nitrogen transformation, microbiota dynamics, and assembly patterns in constructed wetlands. *Water Research* **2022,** *220*, 118636.

50. Huang, X.; Yang, L.; Zhou, S.; Zhong, L.; Xu, G.; Bi, M.; Yang, X.; Su, X.; Rillig, M. C., Plastic biofilms as hotspots of nitrogen cycling in estuarine ecosystems: Comparative ecological, genomic, and transcriptomic analysis across substrates. *Global Change Biology* **2025,** *31*, (7), e70329.

51. Savoca, M. S.; Wohlfeil, M. E.; Ebeler, S. E.; Nevitt, G. A., Marine plastic debris emits a keystone infochemical for olfactory foraging seabirds. *Science Advances 2*, (11), e1600395.

52. Procter, J.; Hopkins, F. E.; Fileman, E. S.; Lindeque, P. K., Smells good enough to eat: Dimethyl sulfide (DMS) enhances copepod ingestion of microplastics. *Mar. Pollut. Bull.* **2019,** *138*, 1-6.

53. Rillig, M. C.; Leifheit, E.; Lehmann, J., Microplastic effects on carbon cycling processes in soils. *PLoS Biol.* **2021,** *19*, (3), e3001130.

54. Chen, X.; Yu, X.; Zhang, L.; Zhao, W.; Sui, Q., Organic pollutants adsorbed on microplastics: Potential indicators for source appointment of microplastics. *Journal of Hazardous Materials* **2024,** *465*, 133225.

55. Rillig, M. C.; Kim, S. W.; Zhu, Y.-G., The soil plastisphere. *Nat. Rev. Microbiol.* **2024,** *22*, 64–74.

56. Hall-Stoodley, L.; Costerton, J. W.; Stoodley, P., Bacterial biofilms: from the Natural environment to infectious diseases. *Nat. Rev. Microbiol.* **2004,** *2*, (2), 95-108.

57. Mincer, T. J.; Zettler, E. R.; Amaral-Zettler, L. A., Biofilms on plastic debris and their influence on marine nutrient cycling, productivity, and hazardous chemical mobility. In *Hazardous Chemicals Associated with Plastics in the Marine Environment*, Takada, H.; Karapanagioti, H. K., Eds. Springer International Publishing: Cham, 2016; pp 221-233.

58. Bowley, J.; Baker-Austin, C.; Porter, A.; Hartnell, R.; Lewis, C., Oceanic hitchhikers – Assessing pathogen risks from marine microplastic. *Trends Microbiol.* **2021,** *29*, (2), 107-116.

59. Song, J.; Beule, L.; Jongmans-Hochschulz, E.; Wichels, A.; Gerdts, G., The travelling particles: community dynamics of biofilms on microplastics transferred along a salinity gradient. *ISME Communications* **2022,** *2*, (1), 35.

60. Beloe, C. J.; Browne, M. A.; Johnston, E. L., Plastic debris as a vector for bacterial disease: An interdisciplinary systematic review. *Environ. Sci. Technol.* **2022,** *56*, (5), 2950-2958.

61. Gkoutselis, G.; Rohrbach, S.; Harjes, J.; Brachmann, A.; Horn, M. A.; Rambold, G., Plastiphily is linked to generic virulence traits of important human pathogenic fungi. *Communications Earth & Environment* **2024,** *5*, (1), 51.

62. Jiang, W.; Liang, P.; Wang, B.; Fang, J.; Lang, J.; Tian, G.; Jiang, J.; Zhu, T. F., Optimized DNA extraction and metagenomic sequencing of airborne microbial communities. *Nature Protocols* **2015,** *10*, (5), 768-779.

Reviewer #4 (Remarks to the Author):

Thanks to the authors for the thoughtful revision of the manuscript. The authors substantially improved the manuscript and pointed out that coverage of Archaeal taxa was sufficient to delineate meaningful conclusions by 16S rRNA amplicon sequence analyses. By linking the metagenomic analyses to such amplicon analyses, the validity of the metagenomics approach becomes apparent. However, the clarity could still be increased on how the metagenomic data were linked to the amplicon data to increase the statistical significance of the functional inference analysis. The limitations and future research needs are highlighted now and nicely presented in the conclusion section.

Response: We thank the reviewer for the positive evaluation of our revision and for recognizing the improvements made to the manuscript. We clarify that, for each sample with both datasets, archaeal amplicon sequencing and shotgun metagenomic sequencing were performed using the same DNA extract. Thus, the two datasets are directly linked at the sample level. In our design, archaeal 16S rRNA amplicon sequencing was used to resolve archaeal community composition, diversity, assembly processes, and environmental associations with higher sensitivity, whereas shotgun metagenomics was used to characterize archaeal functional gene profiles and recover MAGs where possible. The metagenomic analysis was therefore not statistically merged with the amplicon analysis to increase significance; rather, the two approaches provide complementary taxonomic, functional, and genome-resolved evidence from the same environmental DNA samples, supporting a consistent interpretation of plastisphere archaeal community shifts and functional potential.

Specific comments

Comment 1. Previous comment to L58/ L165. My concerns relative to the current taxonomy have not been addressed to sufficient detail. I would encourage the authors to (i) use the same taxonomy for MAGs and marker gene analyses (important to bring meta-genomic and amplicon data together, see comment above), and (ii) update to the current taxonomy. New taxonomy always takes some time to become evident in current literature, but this time should be kept as small as possible. Thus, I don't see a reason for not changing to the current taxonomy only because other studies havn't done so … . In case the authors would like to keep relation to the old taxonomy, the old names might be provided in parentheses.

Response: We thank the reviewer for this important concern and agree that taxonomic consistency and currency are important for clarity. We have carefully considered both aspects of the reviewer's suggestion: unifying the taxonomy between amplicon and MAG analyses, and updating the terminology to current genome-based taxonomy. We agree that this issue required clearer explanation, and we have therefore added a clarifying statement in the Methods section to describe how the two taxonomic frameworks relate to each other and why a simple global name replacement would not be appropriate.

The amplicon and MAG datasets were analyzed using different, method-specific taxonomic frameworks that are not directly interchangeable. For 16S rRNA amplicon data, taxonomy was assigned using the RDP classifier under an rRNA-based reference framework. For MAGs, taxonomy was assigned using GTDB-Tk against GTDB r207, a standardized genome-based archaeal taxonomy framework used consistently for our genome-resolved analyses. Because these frameworks differ in both classification boundaries and nomenclature, several classical archaeal higher-level names used in rRNA-based taxonomy do not correspond one-to-one to genome-based GTDB taxa. For example, many lineages historically assigned to Thaumarchaeota in rRNA-based frameworks correspond broadly to Nitrososphaeria within Thermoproteota in

GTDB; Crenarchaeota-related rRNA lineages are also reorganized within Thermoproteota-related genome-based taxonomy; traditional Euryarchaeota sensu lato has been split across multiple GTDB phyla/lineages, including Thermoplasmatota/Poseidoniales (Marine Group II) and other groups; and Woesearchaeota and Pacearchaeota correspond to DPANN/Nanobdellati-related lineages with revised boundaries in genome-based taxonomy. Therefore, directly replacing rRNA-based names in the amplicon-based ASV analyses with GTDB names would risk introducing misleading taxonomic equivalences.

For this reason, we retained the rRNA-based names for amplicon-derived ASV analyses and used GTDB names for genome-resolved MAG analyses, while explicitly cross-referencing the two nomenclatures where relevant. We believe this approach best preserves the accuracy and internal consistency of each analysis while helping readers connect the amplicon and MAG results.

The added clarification is as below (Lines 530 – 544):

For internal consistency with the marker-gene analysis, archaeal taxonomy from amplicon data is reported using the SILVA/RDP framework, whereas genome-based GTDB taxonomy is used separately for MAG analyses. These two frameworks use different classification boundaries and nomenclature, reflecting distinct underlying methodologies: rRNA-based marker-gene classification versus genome phylogeny-based taxonomy. Consequently, some archaeal higher-level names differ between the amplicon and MAG analyses and cannot be reconciled by simple one-to-one name replacement. For example, many lineages historically assigned to Thaumarchaeota in rRNA-based frameworks correspond broadly to Nitrososphaeria within Thermoproteota in GTDB; Crenarchaeota-related rRNA lineages are also reorganized within Thermoproteota-related genome-based taxonomy; traditional Euryarchaeota *sensu lato* has been split across multiple GTDB phyla/lineages, including Thermoplasmatota/Poseidoniales (Marine Group II) and other groups; and Woesearchaeota and Pacearchaeota correspond to DPANN/Nanobdellati-related lineages with revised boundaries in genome-based taxonomy. We therefore retained SILVA/RDP names for amplicon-derived ASV analyses and used GTDB names for genome-resolved MAG analyses, while providing key cross-references between the two frameworks where relevant.

Comment 2. Previous comment to L92-100/108/180 and elsewhere. Nitrososphaera were also found enriched in the plastisphere – maybe this is a general trend across different habitats. In my view, it is important to delineate some general effects (or move towards a systematic understanding) of plastic on Archaeal communities and thus increase the impact of the study. This can only be done in case previous studies on Archaea in the plastisphere are included and findings compared in a concise way. Current literature coverage on Archaea in the plastisphere might be helpful. Eventually, it would be better to place such a paragraph in the discussion. This will keep a concise style.

Response: We thank the reviewer for this useful suggestion. We have added a concise comparison with previous plastisphere studies to place the enrichment of Nitrososphaera/Nitrososphaerales-related archaea in a broader context.

The added text is as follows (Lines 194 – 199):

The enrichment of Nitrososphaerales in the plastisphere is consistent with observations from other systems, where Candidatus Nitrosocosmicus progressively increased on coastal plastic biofilms during a 39-month colonization experiment[61] and Nitrososphaeraceae were enriched in

the benthic plastisphere of aquaculture ponds[62], suggesting that the plastisphere may recurrently favor AOA across aquatic systems.

Comment 3. Previous comment to L253-258 (now L273-279). Could the *nirK* genes stem from ammonia oxidizers reflecting the potential for nitrifier denitrification rather than standard denitrification?

Response: Thanks to the reviewer for the question. The reviewer is correct that *nirK* detected in ammonia-oxidizing archaeal MAGs may reflect AOA-associated nitrite reduction rather than standard denitrification. This interpretation is consistent with our original wording, where we described the presence of *amoABC*, *ureABC*, and *nirK* in Nitrososphaerales MAGs as evidence for "ammonia oxidation and related nitrogen transformations" rather than as direct evidence of standard denitrification.

To make this point clearer, we have revised the relevant sentence as follows: "Our genome-resolved analyses further supported this pattern: Nitrososphaerales MAGs were enriched in the plastisphere and carried key nitrogen-cycling genes, including complete *amoABC*, *ureABC*, and *nirK*, consistent with the potential for ammonia oxidation and related nitrogen transformations, including AOA-associated nitrite reduction, in plastisphere biofilms (Supplementary Note S4 and Figs. S8 and S9)."

Comment 4. Previous comment to L506. The approach utilized to detach cells from plastic is referred to as being established. Is there any evidence from other studies on how complete the detachment is? The rarefaction analyses will not really help here … . In case there is no evaluation of detachment efficiencies possible, please acknowledge. This is not a major issue, since the same method was applied to all samples.

Response: Thanks to the reviewer for this question. We did not intend to claim that cell detachment from plastic debris was quantitatively complete. To our knowledge, there is currently no standardized method for determining absolute cell or DNA detachment efficiency from field-collected environmental plastic debris, especially given the heterogeneity of environmental plastic particles in size, shape, polymer type, surface roughness, weathering status, and biofilm structure. In this study, we used an adapted membrane-filtration DNA extraction workflow based on a published Nature Protocols method widely used in environmental microbiome studies. Rather than extracting DNA from only a few individual particles or using swab-based surface collection, we pooled the plastic debris collected within each sample, repeatedly washed/sonicated them in sterile 1× PBS, filtered the entire wash through a 0.22 μm membrane, and extracted DNA from the membrane. This approach was designed to maximize recovery of plastisphere-associated microbial material and reduce stochastic effects from incomplete detachment of individual particles. Although the absolute detachment efficiency cannot be quantified, all plastisphere samples were processed using the same protocol, and our conclusions are based on relative community composition and functional-potential comparisons between plastisphere and bulk-water samples, rather than on quantitative estimates of total cells detached from plastic debris.

I am for constructive and fair feedback and hope the authors can appreciate it as such. Altogether, the negligible relative abundance of archaea on plastic marine debris diminishes the scope and impact of this study, which I regard as being methodologically sound and thoughtful – inadvertently misleading though because the relative abundance data are not displayed and may very well verify that archaeal abundance is negligible.

The manuscript by Wang et al. investigates archaeal communities colonizing plastic marine debris and those subsisting in surrounding seawaters. The authors implemented a granular taxonomic annotation scheme (i.e., taxonomy based on 16S and whole-genome-shotgun sequencing) followed by an analysis of functional metabolic potential, including biochemically proven metabolic pathways. The manuscript is refreshingly well-written and the bioinformatic approach is mainly sound (although I have some questions regarding assembly, DIAMOND vs. BLAST for annotating "biogeochemical cycling genes", and whether or not metagenome-assembled genomes could be binned.) Furthermore, the authors have taken particular care to demonstrate that collected particles were indeed plastic via O-PTIR spectroscopy, which I appreciate.

The authors have astutely pointed out that archaea make important biogeochemical contributions in various systems and that coastal habitats are laden with plastic pollution, which microbes respond to writ large – a promising area of inquiry to understand how microbes adapt to plastic surfaces. The former statements need to be contextualized though because of archaea's low abundance on plastic – this is being oversold (discussed more below.) Archaeal community assembly (and frankly a description of archaea in the plastisphere) is poorly understood and not well-represented in the literature. This may be in part because of stochastic influences on archaea, as the authors empirically demonstrate herein. It may also be because archaea are not as effective colonizers of surfaces and are generally more specialized for a distinct niche. The lack of data on archaea in the plastisphere may also be directly related to archaea's low relative abundance in the plastisphere (I believe this is likely the main reason why there are few studies on archaea in the plastisphere.) In other words, most studies of the plastisphere, which predominantly employ 16S sequencing, do not show hardly any presence of archaea. There are caveats to that generalization, but our current body of knowledge suggests that archaea are a negligible component of epiplastic communities, both early and mature biofilms.

With regards to the present study, for 16S sequencing (and recognizing that microbes have different copy numbers of the 16S, 'preferential' amplification, etc), I did not see a taxonomic comparison of the relative abundance of archaea and bacteria (Figure 3 appears to have relative abundance data, but I suspect it can be interpreted as Thaumarchaeota are >60% of the relative abundance of all archaea in this study.) For WGS data, I did not see a taxonomic

comparison of the relative abundance of prokaryotes and eukaryotes, etc. Do archaea exhibit a higher-than-expected relative abundance in this study (compared to the other domains?) If so, these data need to be front and center to demonstrate what proportion of the community we are looking at. I suspect that we are looking at <0.5-1% of the total community abundance (probably even less, but I don't want to make the assumption – I would like the authors to communicate that should this manuscript proceed to a revision.) This is not to say that archaea do not play some role in microbial community assembly or have a biogeochemical influence – they just exist in such small relative proportions compared to bacteria, for example, and consequently have a lesser influence.

L54-L57: Yes, coastal marine ecosystems are of high ecological and social importance, and archaea are found there, but what is their abundance in epiplastic communities in early and mature stages? I recognize that the authors captured free-drifting plastics rather than incubating polymers.

L60-L63: That is cool that some pathways were enriched on plastic compared to seawater, but suggesting that it could alter nutrient cycles just with archaea is a real stretch.

L72-L133: This is a terrific, succinct discussion.

L137-L138: OK, but how does the relative abundance compare across plastic samples and in seawater? I would not compare 16S vs. WGS relative abundance data, but there needs to be data for both 16S and WGS relative abundances calculated separately.

L165-L167: 97% of what percentage of all sequences from phylogenetically distinct microbes?

L215-L218: I agree with this statement if it is about bacteria, though the cell count numbers for microbes on surface plastics is on the order of 10^{21}, which is incredibly small compared to what's in the water column at the same depths. Moreover, the data from other studies suggest archaea have low relative abundance in the plastisphere and the data from this study suggests that stochastic processes drive their presence.

L220-L235: I recognize the logic behind this notion and appreciate it. Might other sources of methane (e.g., livestock; hydrothermal vent fluid) be more important contributors? We are likely talking about small relative proportions of archaea in the plastisphere contributing to methane cycling.

L237-L258: This is a really cool idea when paired with the notion that microbes adapt to plastic and can "perturb" nutrient cycles. I suspect that this would be more pertinent to the bacteria living in epiplastic biofilms though not archaea.

L284: there are no metatranscriptomic nor respiration data in this study. How can you be so certain that archaeal communities are “highly” active in biogeochemical processes in the plastisphere? I would revise this to reflect having amplicon and metagenomic data. Are there any pathways that are enriched on different polymer types?

L286-L288: Scaling up is a cool idea, but the context matters here. If we posit that Zhao et al. 2020’s microbial carrying capacity numbers are conservative (i.e. 10^{21} microbes on surface plastics), and that 0.5% of these microbes may be archaea (i.e. $5x10^{18}$), archaeal numbers are miniscule compared to the rest of the plastisphere and what’s in the surrounding water column. I agree with your statement “Moreover, our study only considered surface waters and significant knowledge gaps remain regarding plastisphere dynamics throughout the water column and deep ocean ecosystems” on L472-L473. However, even considering microbial biomass on plastics in the ocean’s interior, which tend to be significantly smaller pieces of plastic than what’s at the surface, there’s going to be limited space for microbes to colonize.

L496-L409: what about relative abundance of archaea and bacteria? I would include those data in Figure 1.

L424-L432: I see the train of thought here, but how can you come to this conclusion when stochastic processes

L449-L450: “everything is everywhere, but the environment selects.” Presence does not mean activity nor processes occur nor viability.

L544-L549: Was there an opportunity to bin archaeal metagenome-assembled genomes from the dataset? What about with a coassembly? I suppose the sequencing depth is not deep enough and low abundance members are challenging to get MAGs for.
